# Microwave‐Enhanced Synthesis of 2‐Styrylquinoline‐4‐Carboxamides With Promising Anti‐Lymphoma Activity

**DOI:** 10.1002/ardp.70148

**Published:** 2025-11-24

**Authors:** Ignazio Sardo, Lorenzo Manfreda, Giulia Maria Titone, Marilia Barreca, Roberta Bivacqua, Virginia Spanò, Sara Amata, Arianna Zanolli, Roberta Bortolozzi, Maria Valeria Raimondi, Giampietro Viola, Paola Barraja, Alessandra Montalbano

**Affiliations:** ^1^ Department of Biological, Chemical and Pharmaceutical Sciences and Technologies (STEBICEF) University of Palermo Palermo Italy; ^2^ Department of Woman's and Child's Health University of Padova Padova Italy; ^3^ Istituto di Ricerca Pediatrica IRP Fondazione Città della Speranza Padova Italy; ^4^ Department of Pharmaceutical and Pharmacological Sciences University of Padova Padova Italy

**Keywords:** 2‐styrylquinoline‐4‐carboxamides, diffuse large B‐cell lymphoma, DNA synthesis inhibition, microwave‐assisted organic synthesis, mitochondria‐dependent apoptosis

## Abstract

Diffuse large B‐cell lymphoma (DLBCL) is the most common subtype of non‐Hodgkin lymphoma, characterized by significant clinical and molecular heterogeneity. Here, we report the design and synthesis of a novel series of 2‐styrylquinoline‐4‐carboxamides via an efficient microwave‐assisted organic synthesis (MAOS) approach. This strategy enabled the rapid and high‐yielding isolation of derivatives **4a–z** and **4aa–ah** in three steps from commercially available isatin. Among the 34 compounds synthesized, 24 showed antiproliferative activity in vitro, with compound **4i** displaying sub‐micromolar IC₅₀ values across multiple lymphoma cell lines, including SU‐DHL‐8 and TOLEDO. Mechanism of action studies demonstrated that **4i** was able to induce G₂/M cell‐cycle arrest and DNA synthesis suppression, coupled with mitochondrial membrane depolarization and reactive oxygen species (ROS) accumulation, suggesting activation of the intrinsic apoptotic pathway. Importantly, active derivatives were nontoxic to healthy peripheral blood mononuclear cells (PBMCs), indicating a favorable therapeutic window. These results validate the quinoline scaffold as a promising chemotype, highlighting the utility of MAOS for the sustainable synthesis of bioactive heterocycles.

## Graphical abstract

A microwave‐assisted synthesis enabled the efficient preparation of novel 2‐styrylquinoline‐4‐carboxamides with potent antiproliferative activity against lymphoma cell lines. Among them, compound **4i** induced G₂/M arrest and mitochondria‐mediated apoptosis, showing selectivity for tumor over healthy cells.

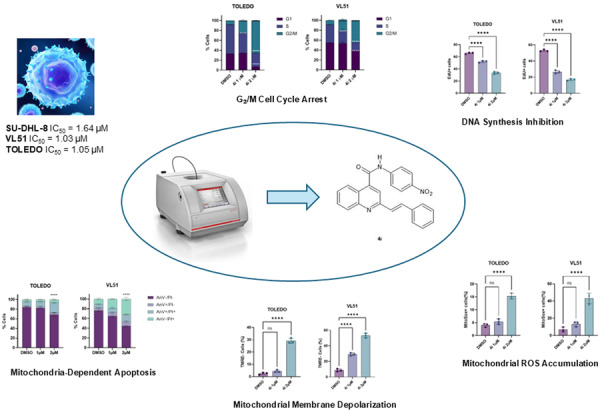

## 1 Introduction

Non‐Hodgkin lymphoma (NHL) represents an extremely heterogeneous group of lymphoid cancers arising from the malignant transformation of B and T lymphocytes, and, less frequently, natural killer (NK) cells. A wide array of subtypes exists, distinguished by unique molecular, cellular, and immunological features impacting their pathogenesis, clinical presentation, and treatment response [1, 2]. Among these, diffuse large B‐cell lymphoma (DLBCL) stands out as the most common and clinically relevant subtype, accounting for 30%–40% of all NHL cases worldwide [3].

DLBCL is an aggressive and rapidly proliferating B‐cell malignancy characterized by its significant clinical variability. The disease generally affects adults, with the average age at diagnosis of about 60 years, though younger patients can also be impacted; men are slightly more frequently affected than women [3, 4]. Malignant cells arise either from the germinal center or post‐germinal center stages, and recent advancements in genomic profiling have allowed further sub‐classification into molecularly distinct subtypes [5]. These include the germinal center B‐cell‐like (GCB) subtype, which tends to have a more favorable prognosis, and the activated B‐cell‐like (ABC) subtype, which is typically associated with a poorer clinical outcome. Additional genomic studies have even refined the categorization into five clusters based on mutational landscapes, offering significant implications for patient stratification and the development of targeted therapies [6].

The pathogenesis of DLBCL is strongly associated with a spectrum of genetic alterations. Chromosomal translocations, such as those involving BCL‐2 and MYC, are pivotal events that lead to the overexpression of oncogenes, thereby driving uncontrolled cell proliferation and evasion of apoptosis [7]. Moreover, point mutations, epigenetic modifications, and dysregulated signaling pathways, including NF‐κB, JAK‐STAT, and PI3K‐AKT, further contribute to the disease progression. These molecular disruptions not only affect the biological behavior of DLBCL but also influence its response to therapy [8].

Clinically, DLBCL typically presents a rapid enlargement of lymph nodes, most commonly in the cervical, axillary, or inguinal regions. About 40% of cases exhibit extranodal involvement, affecting organs such as the gastrointestinal tract, central nervous system (CNS), bone marrow, and skin. Patients often present systemic “B symptoms” including fever, night sweats, and weight loss. In cases where the CNS is involved, the prognosis is notably worse, necessitating specialized diagnostic and treatment approaches [9]. The diagnosis of DLBCL is confirmed by histopathological examination, supported by immunohistochemical staining and fluorescence in situ hybridization (FISH) to detect specific genetic rearrangements, which are critical for distinguishing it from other B‐cell lymphomas [10].

The standard treatment protocol for DLBCL involves R‐CHOP chemo‐immunotherapy—a combination of rituximab (R), cyclophosphamide (C), doxorubicin (H), vincristine (O), and prednisone (P)—which achieves complete remission in approximately 60%–70% of patients [11]. However, a significant proportion of patients either relapse or present refractory disease, underscoring the need for novel therapeutic strategies [12]. In recent years, advances in targeted therapies, including Bruton's tyrosine kinase inhibitors, BCL2 inhibitors, and innovative immunotherapeutic approaches such as chimeric antigen receptor (CAR) T‐cell therapies and antibody‐drug conjugates such as Brentuximab Vedotin, have provided promising alternatives. These emerging treatments, alongside ongoing refinements in molecular profiling and precision oncology, are reshaping the therapeutic landscape and hold promises for overcoming the challenges posed by DLBCL's heterogeneous biology [13].

Despite the progress achieved, the treatment of refractory or relapsed DLBCL still represents an unmet clinical need, highlighting the urgency of novel and more effective therapeutic options [14]. The improved knowledge of the bio‐molecular pathways behind the pathogenesis of DLBCL has fostered the development of novel small molecule inhibitors of key players in tumorigenesis. The quinoline core has been widely investigated in drug design. It represents a frequent structural motif not only in antibacterial, antifungal, antiviral, and anti‐neurodegenerative agents, but also in antiproliferative compounds [15, 16]. Moreover, the styryl moiety is recurrent in compounds endowed with antiproliferative activity, and styryl‐heterocyclic hybrids have been developed as a strategy to improve resveratrol's anticancer properties [17]. In particular, 2‐styrylquinolines have been described for their antiproliferative activity against solid tumor cell lines (HTC116, HepG2, and MDA‐MB468 with IC_50_ values at micromolar level) [17] as well as on leukemia and lymphoma cell lines [18].

**Figure 1:**
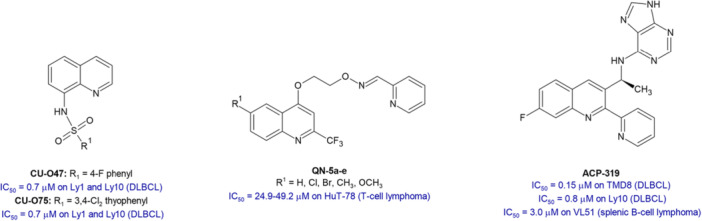
Quinoline‐based compounds with cytotoxic activity against lymphoma.

Since quinoline‐4‐carboxamides derivatives have been investigated for their antiproliferative activity [19] and quinolines have recently emerged as valuable candidates in the treatment of lymphoma [20, 21, 22, 23, 24, 25, 26] (Figure 1), we continued our studies on nitrogen heterocyclic systems as anti‐lymphoma agents [27, 28, 29, 30] approaching a versatile microwave‐assisted synthesis [31, 32] leading to a novel series of 2‐styrylquinoline‐4‐carboxamides **4a–z** and **4aa–ah**. The new compounds were assessed for their antiproliferative effect against the SU‐DHL‐8 DLBCL cell line, showing in some cases good potency with IC_50_ values in the sub‐micromolar range.

## 2 Results and Discussion

### 2.1 Chemistry

**Scheme 1:**
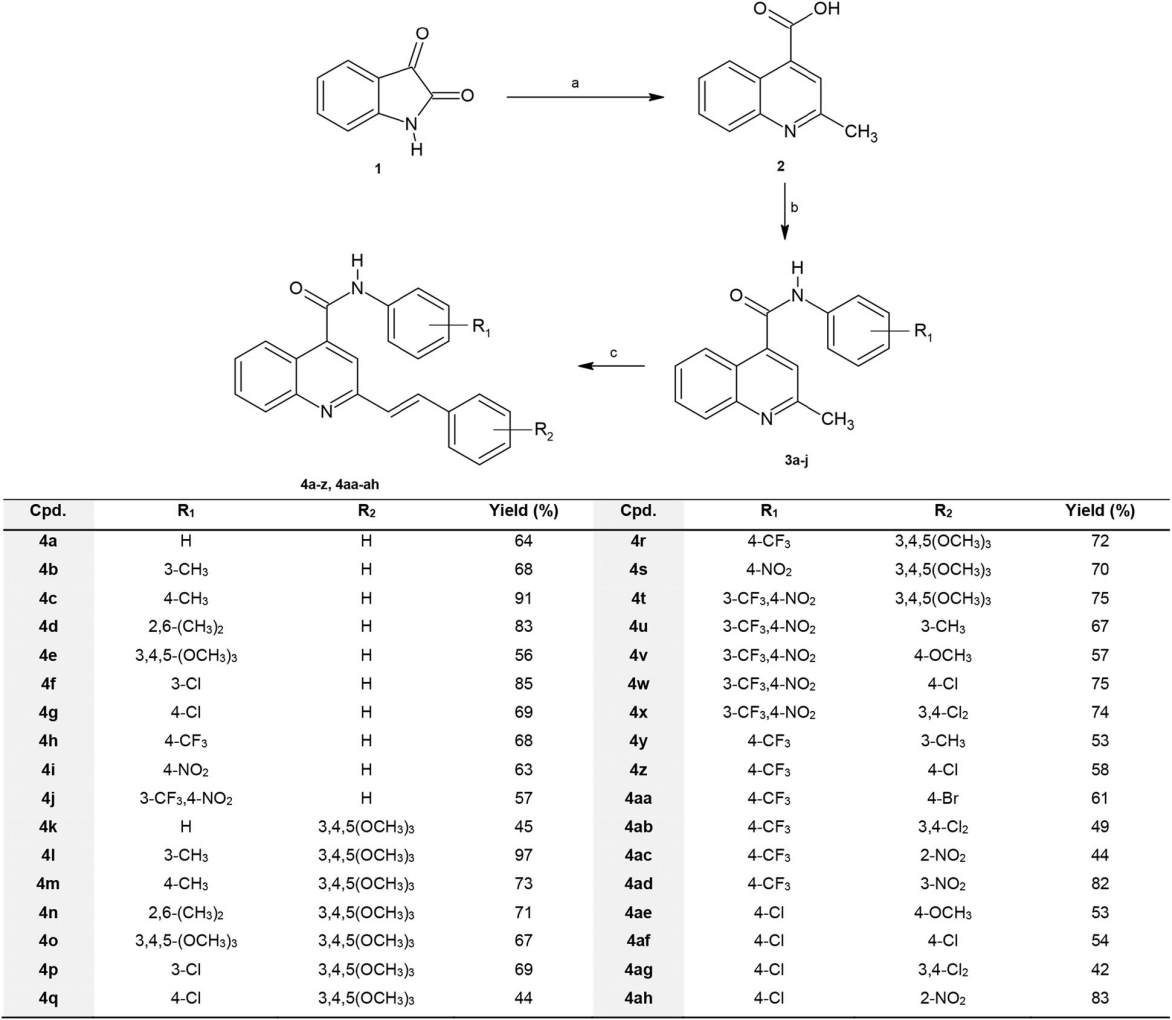
Reagents and conditions. (a) 1. KOH 10%, (CH₃)₂CO; MW: 150°C, 15 min, 100 W (2 cycles); 2. HCl 37%, pH < 6.5; b) 1. EDC·HCl (2 eq), HOBt (0.2 eq), DMAP (2 eq), ACN; 2. Substituted anilines (R₁) (1–1.5 eq); MW: 50°C, 20 min, 60 W (2 cycles); c) Substituted aldehydes (R₂) (1–2 eq), glacial AcOH; MW: 150°C, 20 min, 200 W (2 cycles). A cooldown process has been applied between each cycle of microwave irradiation.

The quinoline derivatives **4a–z** and **4aa–ah** were obtained in good yields through an efficient three‐steps MAOS procedure (Scheme 1). Considering that, to the best of our knowledge, no examples of microwave‐assisted syntheses of 2‐styrylquinoline‐4‐carboxamides have been reported in the literature, we explored this approach as an efficient strategy to obtain high yields of the desired compounds while reducing the reaction time.

The synthetic route starts from the commercially available isatin (**1**), which upon Pfitzinger reaction with acetone under basic conditions, has been converted into the intermediate 2‐methylquinoline‐4‐carboxylic acid (**2**). By applying microwave‐assisted organic synthesis (MAOS) under closed‐vessel conditions using a monomodal microwave reactor, intermediate (**2**) was obtained in 93% yield after 30 min (two 15‐min cycles) at 100 W and 150°C. This procedure efficiently improved the yields (48%) and reduced the reaction time (7 h) in comparison with the conventional heating method reported in the literature [33]. The subsequent amidation reaction was conducted using *N*‐ethyl‐*N’*‐(3‐dimethylaminopropyl)carbodiimide hydrochloride (EDC·HCl), hydroxybenzotriazole (HOBt) as activating agents and 4‐dimethylaminopyridine (DMAP) as catalyst, under stirring at room temperature. When amidations were performed using the conventional method, reaction times and yields were strongly influenced by the electronic properties of the substituent. Indeed, electron‐withdrawing substituted anilines required longer reaction times (72 h) leading to the desired compounds of type **3** in lower yields (13%–57%). On the other hand, the 2‐methyl‐*N*‐phenylquinoline‐4‐carboxamides (**3a–j**) were efficiently obtained in higher yields (45‐83%) using microwave heating for 40 min (two 20‐min cycles) at 60 W and 50°C (Table 1). Reaction conditions for the amidation, leading to derivatives **3a–j**, were optimized by monitoring the reaction leading to intermediate **3c**. After the first cycle at 50°C, for 20 min, 60 W, some starting materials were still detectable in the reaction mixture. Therefore, another cycle was applied, which allowed us to isolate the desired compound **3c** in good yields (68%). Further prolongation of the reaction time of each cycle led to a decrease in yield due to the formation of by‐products in the reaction mixture. The synthetic procedure optimized for **3c** proved to be efficient also in isolating those intermediates obtained by reaction of compound **2** with electron‐withdrawing substituted anilines. Therefore, this procedure has been subsequently applied to isolate all desired compounds (**3a–j**).

**Table 1:** Comparison of experimental reaction conditions for compounds 3‐j.

		Conventional method		Microwave	
Cpd.	R_1_	Reaction time	Yield (%)	Reaction time	Yield (%)
**3a**	H	4 h 30′, r.t.	78	50°C, 20 min, 60 W (2 cycles)	83
**3b**	3‐CH_3_	24 h, r.t.	49	50°C, 20 min, 60 W (2 cycles)	81
**3c**	4‐CH_3_	24 h, r.t.	47	50°C, 20 min, 60 W (2 cycles)	68
**3d**	2,6‐(CH_3_)_2_	48 h, r.t.	54	50°C, 20 min, 60 W (2 cycles)	65
**3e**	3,4,5‐(OCH_3_)_3_	48 h, r.t.	59	50°C, 20 min, 60 W (2 cycles)	65
**3f**	3‐Cl	72 h, r.t.	54	50°C, 20 min, 60 W (2 cycles)	69
**3g**	4‐Cl	72 h, r.t.	57	50°C, 20 min, 60 W (2 cycles)	65
**3h**	4‐CF_3_	72 h, r.t.	40	50°C, 20 min, 60 W (2 cycles)	69
**3i**	4‐NO_2_	72 h, r.t.	31	50°C, 20 min, 60 W (2 cycles)	55
**3j**	3‐CF_3_,4‐NO_2_	72 h, r.t.	13	50°C, 20 min, 60 W (2 cycles)	45

The final reaction step exploited the imine–enamine tautomerism of the 2‐methylquinoline core, enabling the formation of a reactive exocyclic methylene group susceptible to nucleophilic attack by the suitable substituted aldehyde under acidic conditions. By microwave irradiation for 40 min at 150°C and 200 W, the intermediate **3c** was successfully converted into the final product **4c** in very high yields (91%). Therefore, the same reaction conditions have been applied in the synthesis of all desired compounds, **4a–z** and **4aa–ah**, that have been isolated in yields ranging from 42% to 97%.

With the exception of a few compounds (**4b,c,f,g,h,l,m,o,p,q,ad,ah**) which were purified by crystallization from ethanol, medium‐pressure liquid chromatography (MPLC) has been used as an efficient purification method to isolate substituted *N*‐phenyl‐2‐[(*E*)‐2‐phenylethen‐1‐yl]quinoline‐4‐carboxamides. Compared with lower pressure chromatographic methods, MPLC offers faster and more efficient separations, thus reducing both solvent and chemical waste disposal costs [34, 35]. This is in line with our efforts in employing synthetic methods and purification procedures with maximal environmental compatibility. As a matter of fact, MAOS provides significant added value by combining reduced solvents consumption, higher yields, and solutions to complex syntheses, with higher reaction rates leading to considerable energy savings, making the overall synthetic procedure optimized by us, eco‐friendly and compliant with European environmental regulations [36]. Our versatile method allowed the isolation of 34 new derivatives belonging to three different groups according to the R_2_ substituent at the styryl moiety: compounds **4a–j** (R_2_ = H), **4k–4t** (R_2_ = 3,4,5‐trimethoxy), and **4u–4ah** (R_2_ = alkyl, alkoxy, halogen, nitro).

### 2.2 Biology

#### 2.2.1 Cell Growth Inhibitory Effects

The synthesized quinoline derivatives **4a–z** and **4aa–ah** were evaluated in vitro for their antiproliferative activity against a panel of five tumor cell lines (HD‐MB03, medulloblastoma; RPMI‐8402, T‐lymphoblastic leukemia; SU‐DHL‐8, B lymphocyte, large cell lymphoma; A549, non‐small cell lung cancer; MDA‐MB‐231, triple‐negative breast cancer) (Table 2). Within the class of 34 tested compounds, 24 displayed antiproliferative activity, with IC_50_ values ranging from 0.46 to 9.34 µM. By comparing the series of unsubstituted styryl compounds with the 3,4,5‐trimethoxy substituted ones, the latter displayed increased cytotoxicity especially on RPMI‐840 (compare **4b** and **4l**, **4d** and **4n**, **4e** and **4o**, **4f,** and **4p**). Similarly, compounds **4l**, **4o,** and **4p** showed higher antiproliferative activity on MDA‐MB‐231 cell line than the corresponding R_2_‐unsubstituted derivatives (**4b**, **4e**, **4f**). Only compound **4d**, belonging to the unsubstituted styryl series, showed cytotoxic activity against MDA‐MB‐231 cells. Notably, SU‐DHL‐8 B lymphocyte cell line proved to be the most sensitive one (24 active compounds, IC_50_: 0.46–8.27 µM) followed by RPMI‐8402 T‐lymphoblastic leukemia cell line (12 active compounds, IC_50_: 0.79–9.06 M). From data reported in Table 2, **4n** emerged as the most effective of the series, reaching sub‐micromolar activity against both tumor cell lines, SU‐DHL‐8 (0.46 µM) and RPMI‐8402 (0.79 µM). Furthermore, derivative **4ah** (R_1_ = 4‐Cl, R_2_ = 2‐NO₂) showed activity against four tumor cell lines, with IC_50_ values between 1.33 and 6.92 µM. Replacing the 4‐Cl group with a trifluoromethyl group (**4ac**) reduced antiproliferative activity. Except for derivatives **4h**, **4j**, **4w**, **4y**, **4z**, and **4ab** (IC_50_ range = 3.2–8.97 µM), generally, the presence of the trifluoromethyl group resulted in the loss or reduction of biological activity. Interestingly, the most active compounds on SU‐DHL‐8 cell line were **4i** and **4s** (1.64 and 1.37 µM, respectively) characterised by the presence of a nitro group at position 4 on the benzamide moiety.

**Table 2:** In vitro antiproliferative activity (IC₅₀, µM) of quinoline derivatives 4a–z and 4aa–ah against five human cancer cell lines.

Cpd [µM]	R_1_	R_2_	HD‐MB03	RPMI‐8402	SU‐DHL‐8	A549	MDA‐MB‐231
**4a**	H	H	> 10	> 10	> 10	> 10	> 10
**4b**	3‐CH_3_	H	> 10	> 10	5.05 ± 0.58	> 10	> 10
**4c**	4‐CH_3_	H	> 10	> 10	> 10	> 10	> 10
**4d**	2,6‐(CH_3_)_2_	H	> 10	> 10	6.53 ± 1.49	> 10	5.60 ± 0.79
**4e**	3,4,5‐(OCH_3_)_3_	H	7.10 ± 1.15	> 10	6.61 ± 0.76	> 10	> 10
**4f**	2‐Cl	H	> 10	> 10	8.27 ± 0.72	> 10	> 10
**4g**	4‐Cl	H	> 10	6.31 ± 0.36	7.80 ± 1.30	> 10	> 10
**4h**	4‐CF_3_	H	> 10	8.97 ± 0.75	7.09 ± 0.25	> 10	> 10
**4i**	4‐NO_2_	H	> 10	1.14 ± 0.12	1.64 ± 0.25	> 10	> 10
**4j**	3‐CF_3_,4‐NO_2_	H	8.23 ± 0.47	> 10	3.86 ± 0.44	> 10	> 10
**4k**	H	3,4,5‐(OCH_3_)_3_	> 10	> 10	5.33 ± 0.52	> 10	> 10
**4l**	3‐CH_3_	3,4,5‐(OCH_3_)_3_	> 10	3.75 ± 0.18	2.94 ± 0.08	9.34 ± 0.18	5.95 ± 0.66
**4m**	4‐CH_3_	3,4,5‐(OCH_3_)_3_	> 10	> 10	> 10	> 10	> 10
**4n**	2,6‐(CH_3_)_2_	3,4,5‐(OCH_3_)_3_	> 10	0.79 ± 0.07	0.46 ± 0.05	> 10	> 10
**4o**	3,4,5‐(OCH_3_)_3_	3,4,5‐(OCH_3_)_3_	> 10	4.55 ± 0.42	2.09 ± 0.14	> 10	5.95 ± 0.89
**4p**	2‐Cl	3,4,5‐(OCH_3_)_3_	8.31 ± 0.64	9.06 ± 0.59	4.46 ± 0.37	> 10	5.70 ± 0.10
**4q**	4‐Cl	3,4,5‐(OCH_3_)_3_	> 10	> 10	4.61 ± 0.84	> 10	> 10
**4r**	4‐CF_3_	3,4,5‐(OCH_3_)_3_	> 10	> 10	2.13 ± 0.08	> 10	> 10
**4s**	4‐NO_2_	3,4,5‐(OCH_3_)_3_	> 10	> 10	1.37 ± 0.14	> 10	> 10
**4t**	3‐CF_3_,4‐NO_2_	3,4,5‐(OCH_3_)_3_	> 10	> 10	> 10	> 10	> 10
**4u**	3‐CF_3_,4‐NO_2_	3‐CH_3_	> 10	> 10	> 10	> 10	> 10
**4v**	3‐CF_3_,4‐NO_2_	4‐OCH_3_	> 10	> 10	> 10	> 10	> 10
**4w**	3‐CF_3_,4‐NO_2_	4‐Cl	> 10	3.56 ± 0.44	8.25 ± 0.67	> 10	> 10
**4x**	3‐CF_3_,4‐NO_2_	3,4‐Cl_2_	> 10	> 10	> 10	> 10	> 10
**4y**	4‐CF_3_	3‐CH_3_	3.84 ± 0.22	3.20 ± 0.19	3.25 ± 0.23	8.73 ± 0.74	7.84 ± 0.65
**4z**	4‐CF_3_	4‐Cl	6.73 ± 1.15	> 10	4.77 ± 0.32	> 10	> 10
**4aa**	4‐CF_3_	4‐Br	> 10	> 10	> 10	> 10	> 10
**4ab**	4‐CF_3_	3,4‐Cl_2_	7.83 ± 0.98	> 10	6.54 ± 0.44	> 10	> 10
**4ac**	4‐CF_3_	2‐NO_2_	> 10	> 10	> 10	> 10	> 10
**4ad**	4‐CF_3_	3‐NO_2_	> 10	> 10	> 10	> 10	> 10
**4ae**	4‐Cl	4‐OCH_3_	> 10	6.41 ± 1.16	4.67 ± 0.14	> 10	> 10
**4af**	4‐Cl	4‐Cl	> 10	4.61 ± 0.24	4.45 ± 0.53	> 10	> 10
**4ag**	4‐Cl	3,4‐Cl_2_	6.93 ± 0.06	6.42 ± 0.43	3.49 ± 0.11	7.71 ± 0.56	5.52 ± 0.22
**4ah**	4‐Cl	2‐NO_2_	> 10	1.33 ± 0.02	2.15 ± 0.45	6.92 ± 0.68	4.99 ± 0.35

*Note:* Cell viability was evaluated after 72 h of treatment. Data are represented as mean +/− SEM of at least three independent experiments.

Based on these interesting results on hematological malignancies‐derived cell lines, styrylquinolines **4i, 4n, 4s** and **4ah** were further tested for their antiproliferative activity on a panel of five lymphoma cell lines (VL51, splenic B‐cell lymphoma; TOLEDO, diffuse large B‐cell lymphoma, non‐Hodgkin lymphoma; SU‐DHL‐18, lymph node lymphoma; SU‐DHL‐1, histiocytic large cell lymphoma; KM‐H2, Hodgkin lymphoma) (Table 3). In this case, a different behaviour was observed. Unexpectedly, **4n** and **4s** were devoid of antiproliferative activity, while **4i** confirmed its interesting activity, displaying notable activity across all lymphoma cell lines with IC_50_ values ranging from 1.033 to 7.6 µM. Moreover, **4ah** exhibited slightly lower activity (3.79 ≤ IC₅₀ ≤ 6.74 µM) compared with **4i** on TOLEDO, SU‐DHL‐18, SU‐DHL‐1, and KM‐H2 cells, while showing no activity against the VL51 cell line (IC₅₀ > 10 µM).

**Table 3:** IC₅₀ values (µM) of selected quinoline derivatives (4i, 4n, 4 s, 4ah) against lymphoma‐derived cell lines.

Cpd [µM]	R_1_	R_2_	VL51	TOLEDO	SU‐DHL‐18	SU‐DHL‐1	KM‐H2
4i	4‐NO_2_	H	1.03 ± 0.02	1.05 ± 0.09	7.60 ± 2.80	2.32 ± 1.46	2.80 ± 0.21
4n	2,6‐(CH_3_)_2_	3,4,5‐(OCH_3_)_3_	> 10	> 10	> 10	> 10	> 10
4s	4‐NO_2_	3,4,5‐(OCH_3_)_3_	> 10	> 10	> 10	> 10	> 10
4ah	4‐Cl	2‐NO_2_	> 10	4.93 ± 0.62	3.79 ± 0.51	4.90 ± 0.31	6.74 ± 2.12

*Note:* Cell viability was evaluated after 72 h of treatment. Data are represented as mean +/− SEM of at least three independent experiments.

To assess the potential toxicity of the most promising quinoline derivatives **4i, 4n, 4s,** and **4ah**, an antiproliferative assay was performed, using peripheral blood mononuclear cells (PBMCs) isolated from a healthy donor. All compounds were found to be non‐cytotoxic (IC₅₀ > 10 µM) in both resting and phytohemagglutinin‐stimulated PBMCs, indicating a favorable selectivity profile for tumor cells over normal lymphocytes.

#### 2.2.2 Compound 4i Induces G₂/M Cell‐Cycle Arrest and Inhibits DNA Synthesis in Lymphoma Cell Lines

**Figure 2:**
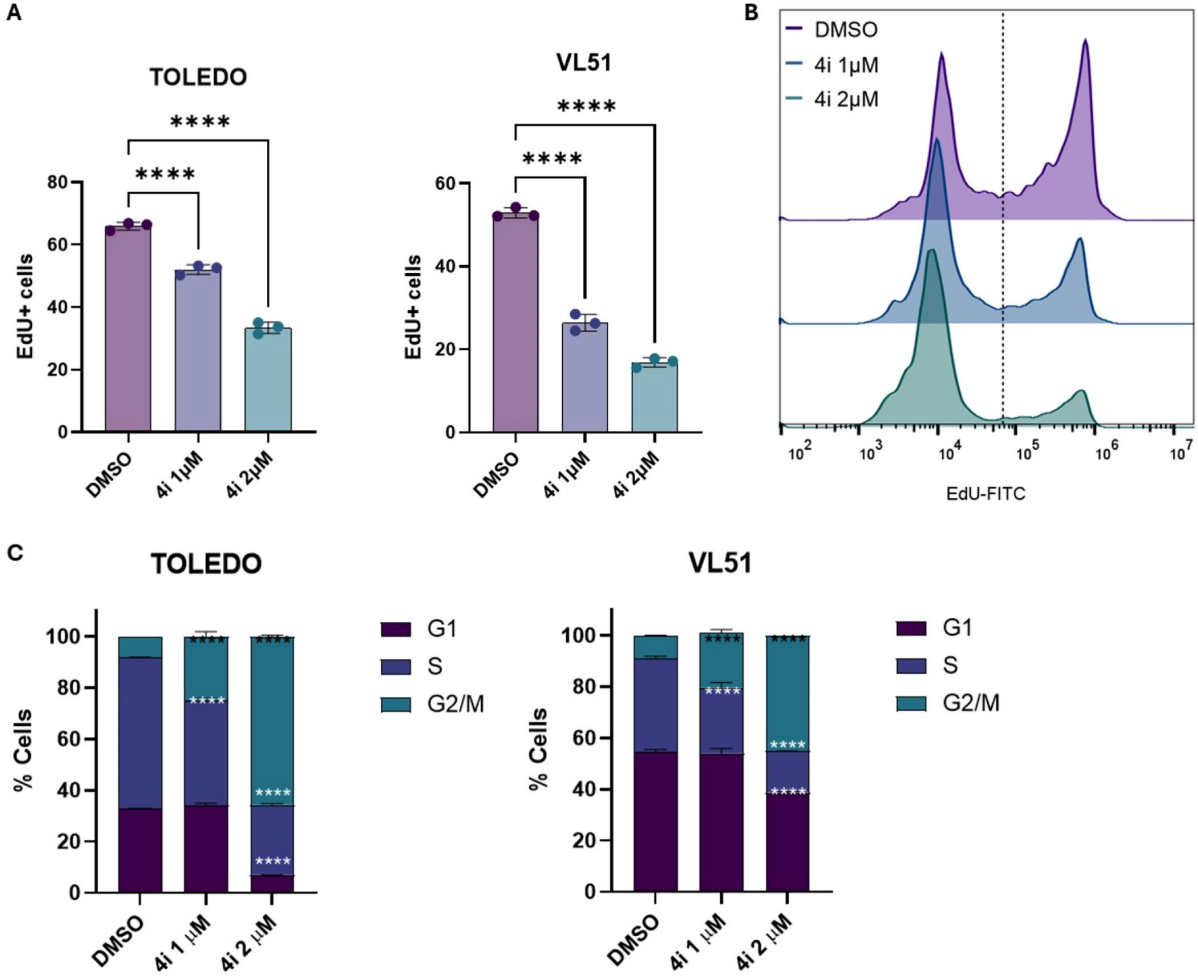
Compound **4i** inhibits cell proliferation and induces G_2_/M cell‐cycle arrest in lymphoma cell lines. (A) EdU incorporation assay showing dose‐dependent reduction in DNA synthesis in TOLEDO and VL51 cell lines after 48 h of treatment with compound **4i** at 1 and 2 μM, respectively. DMSO was used as a vehicle control. Data are presented as mean ± SEM of three independent experiments. **** indicates *p* < 0.0001. (B) Representative flow cytometry histograms of EdU‐FITC fluorescence in TOLEDO cells treated with DMSO (purple), **4i** at the indicated concentrations for 48 h. (C) Cell‐cycle analysis of TOLEDO and VL51 cells treated with DMSO, 1 μM, or 2 μM of compound **4i** for 48 h. Stacked bar graphs show the percentage of cells in G_1_, S, and G_2_/M phases. Data are presented as mean percentages ± SEM of three independent experiments. **** indicates *p* < 0.0001 compared with DMSO control.

Given its potent antiproliferative activity, further insight to assess the mechanism of compound **4i** was conducted in TOLEDO and VL51 lymphoma cell lines. Cell‐cycle analysis after 48 h of exposure to **4i** at 2 µM concentration revealed a significant accumulation of cells in the G₂/M phase, suggesting a dose‐ and time‐dependent cell‐cycle arrest (Figure 2, Panel C). These findings were corroborated by the EdU incorporation assay, which demonstrated a marked, dose‐dependent reduction in DNA synthesis, confirming inhibition of cell proliferation (Figure 2, Panels A and B).

#### 2.2.3 Compound 4i Induces Mitochondria‐Dependent Apoptosis in Lymphoma Cells via Membrane Depolarization and ROS Accumulation

**Figure 3:**
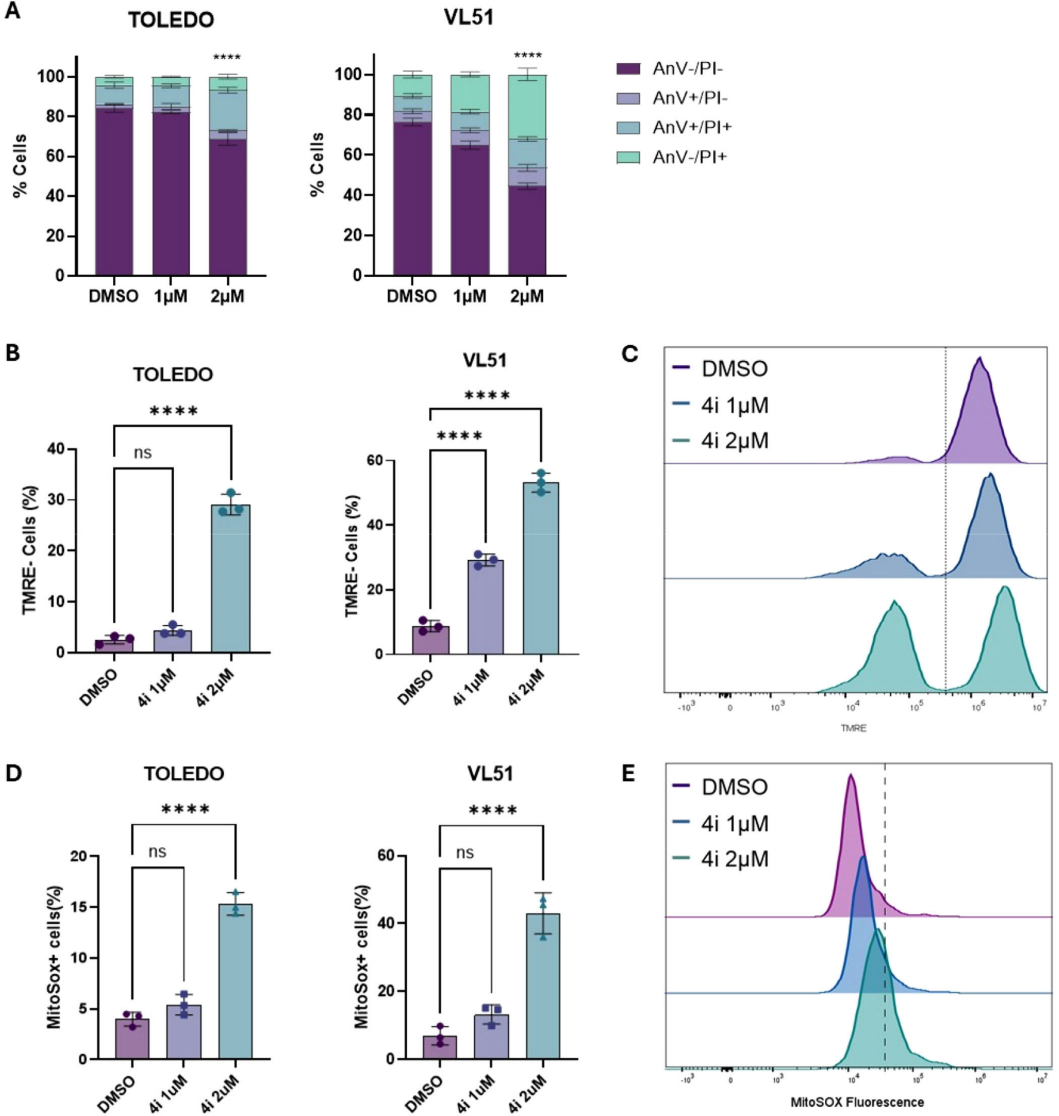
Compound 4i induces apoptosis and mitochondrial dysfunction in lymphoma cell lines. (A) Annexin V/PI staining analysis of TOLEDO and VL51 cells treated with DMSO, 1 μM, or 2 μM of compound 4i for 48 h. Stacked bar graphs show the percentage of cells in different stages of apoptosis. (B) Quantification of TMRE‐negative cells in TOLEDO and VL51 lines after 24 h of treatment with DMSO, and the indicated concentrations of **4i**, indicating mitochondrial membrane depolarization. (C) Representative flow cytometry histograms of TMRE fluorescence in TOLEDO cells treated with DMSO and the indicated concentrations of 4i for 24 h. (D) Percentage of MitoSOX‐positive cells in TOLEDO and VL51 lines after 24 h of treatment with DMSO, 100 nM, or 2 μM of compound **4i**, showing mitochondrial ROS accumulation. (E) Representative flow cytometry histograms of MitoSOX fluorescence in TOLEDO cells treated with DMSO (purple), 1 μM 4i (blue), or 2 μM 4i (green) for 24 h. Data are presented as mean ± SD of three independent experiments. **** indicates *p* < 0.0001, ns indicates not significant compared to DMSO control.

To characterize the mode of cell death induced by **4i,** a cytofluorimetric analysis was performed at 1 and 2 µM using Annexin‐V and propidium iodide (PI) staining on TOLEDO and VL51 cell lines. Annexin V identifies cells undergoing early apoptosis, while double‐positive cells (Annexin V and PI) may represent either necrotic or late apoptotic populations. As shown in the corresponding graphs (Figure 3, Panel A), **4i** induced late apoptosis and necrosis after 48 h at 2 µM. Since the loss of mitochondrial membrane potential (ΔΨm) is a hallmark of intrinsic apoptosis, we analyzed its decrease after 24 h of treatment. Our results show an increased percentage of TMRE‐negative cells (Figure 3, Panel B), indicating early mitochondrial membrane depolarization. As a consequence, a marked dose‐dependent increase of mitochondrial ROS is seen after 24 h of treatment (Figure 3, Panel C). Collectively, these results indicate that compound **4i** induces apoptosis in lymphoma cells through the intrinsic, mitochondria‐dependent pathway, as evidenced by mitochondrial depolarization and ROS accumulation.

## 3 Conclusion

The development of quinoline‐based compounds still represents a promising approach in the search for effective treatments for hematologic malignancies, including diffuse large B‐cell lymphoma (DLBCL). In this study, we synthesized a novel series of 2‐styrylquinoline‐4‐carboxamides using a microwave‐assisted strategy, aiming to improve synthetic efficiency and generate structurally diverse derivatives with potent antiproliferative activity. The in vitro evaluation of these compounds revealed that several derivatives exerted significant cytotoxic activity, particularly against the SU‐DHL‐8 DLBCL cell line, with IC₅₀ values in the sub‐micromolar range. Among them, **4n** emerged as the most effective of the series; compounds **4i** and **4ah** demonstrated broader cytotoxic profiles across multiple lymphoma cell lines in the sub‐micromolar range. These findings suggest a promising therapeutic potential, especially for compound **4i**, which retained efficacy in both germinal center‐derived and activated B‐cell‐like DLBCL models. Structure–activity relationship (SAR) analysis of quinoline derivatives **4a–z** and **4aa–ah** showed that specific aromatic substitutions can have a significant impact on antiproliferative activity. The presence of a nitro group at the 4‐position of the benzamide ring, as observed in compounds **4i** and **4s,** was found to enhance activity, particularly against the SU‐DHL‐8 cell line. Derivative **4ah**, bearing a 4‐chloro and a 2‐nitro group on the benzamide and styryl moieties, respectively, exhibited broad activity against multiple tumour cell lines. However, replacing the 4‐chloro group with a trifluoromethyl group, as in compound **4ac**, led to a marked decrease in activity, indicating the detrimental effect of CF₃ substitution. Generally, trifluoromethyl‐substituted derivatives exhibited reduced or no activity. These findings emphasise the significance of the nature and position of aromatic substituents in modulating the antiproliferative effects of styrylquinoline derivatives. Importantly, the most active derivatives did not exhibit cytotoxicity against normal peripheral blood mononuclear cells (PBMCs), indicating a favorable selectivity profile. Although the precise molecular target(s) of the synthesized quinoline derivatives remain to be identified, our functional data suggest potential interference with cell‐cycle regulatory proteins and mitochondrial homeostasis. The induction of G₂/M arrest, mitochondrial depolarization, and ROS accumulation observed in compound **4i**‐treated lymphoma cells point toward a mitochondria‐dependent apoptotic pathway, which may involve disruption of microtubule dynamics, DNA damage response, or oxidative stress pathways. In conclusion, the biological results reported here not only validate the quinoline scaffold as a valuable pharmacophore for lymphoma therapy but also emphasize the utility of microwave‐assisted organic synthesis (MAOS) for rapid, high‐yield access to complex heterocyclic molecules. Future efforts should also focus on understanding the molecular targets and resistance mechanisms involved, potentially through proteomic or transcriptomic profiling, to refine compound optimization and enhance the potential for clinical translation.

## 4 Experimental

### 4.1 Chemistry

#### 4.1.1 General

All reagents and solvents were purchased from Sigma‐Aldrich, TCI, Fluka, Merck, and Across and used without further purification. Microwave reactions were performed with an Anton Paar GmbH—Monowave 300. All reactions were monitored by thin‐layer chromatography (TLC) on precoated TLC plates (0.2 mm Kieselgel 60 G F254, Merck). Developed plates were air‐dried and visualized by exposure to UV light (λ = 254 and 365 nm). Products were purified by crystallization from EtOH or by medium pressure liquid chromatography (MPLC). MPLC was performed using a CombiFlash RF200 (TeleDyne Isco) and prepacked silica gel RediSep cartridge (35–75 µm). All compounds were named following IUPAC rules as applied by ChemDraw Professional 16.0. All compounds were fully characterized by spectroscopic data. Melting points (m.p) were determined on a Stuart SMP30 melting point apparatus and are uncorrected. Elemental analyses (C, H, N) were within ±0.4% of theoretical values and were performed with a VARIO EL III elemental analyzer. Nuclear magnetic resonance spectra were recorded on a Bruker AC 300 (^1^H: 300 MHz, ^13^C: 75 MHz), a Bruker Avance II 400 (^1^H: 400 MHz, ^13^C: 100 MHz), and a Bruker Avance III HD 600 (^1^H: 600 MHz, ^13^C: 151 MHz), as detailed in Section 4 for each compound, at room temperature using DMSO‐d_6_ as solvent and tetramethylsilane (TMS) as internal standard. Chemical shifts (δ) for ^1^H and ^13^C spectra were acquired in parts per million (ppm). Data are reported as follows: chemical shift (ppm), multiplicity (indicated as: s, singlet; b s, broad singlet; d, doublet; t, triplet; q, d t, doublet of triplets, quartet; b q, broad quartet; m, multiplet), integrated intensity, assignments. The characterization data of compounds **4a–z** and **4aa–ah** are reported in the Supporting Information. The purity and the exact mass of all tested compounds (**4a–z**, **4aa–ah**) were determined as follows. The HPLC system consisted of an Agilent 1260 Infinity II series connected to an Agilent 6540 UHD accurate‐mass quadrupole time‐of‐flight (Q‐TOF) spectrometer equipped with a Dual AJS ESI source and an UV detector (Agilent Technologies, Santa Clara, CA, USA). Separation was performed on a reversed‐phase C18 column ZORBAX Extended‐C18 (2.1 × 50 mm, 1.8 µm) coupled with an Agilent ZORBAX Extended‐C18 security guard column (2.1 × 5 mm, 1.8 µm) supplied from Agilent Technologies, Santa Clara, California, USA. The flow rate was 0.4 mL/min, and the column temperature was set to 30°C. The eluents were formic acid–water (0.1:99.9, v/v) (phase A) and formic acid–acetonitrile (0.1:99.9, v/v) (phase B). The following gradient was employed: 0–10 min, linear gradient from 5% to 95% B; 10–15 min, washing and reconditioning of the column to 5% B. Injection volume was 10 µL. Water and acetonitrile were of HPLC grade, while formic acid was of analytical quality. Both the solvents and the additive were purchased from CARLO ERBA Reagents srl (Milan, Italy). The eluate was monitored through UV absorption at 250 nm and via mass spectrometry total ion count (TIC). N₂ served as the desolvation gas at 300°C with a flow rate of 9 L/min. The nebulizer was calibrated to 45 psi. The sheath gas temperature was established at 350°C with a flow rate of 12 L/min. A capillary potential of 3.5 kV was employed for the positive ion mode. The fragmentor was adjusted to 175 volts. Mass spectra were obtained within the 50 to 1500 *m/z* range.

#### 4.1.2 Synthesis of 2‐Methylquinoline‐4‐Carboxylic Acid (2)

In a 30 mL microwave reaction tube equipped with a magnetic stir bar, isatin (1) (200 mg, 1.36 mmol) was dissolved in 4 mL of aqueous KOH (10%), followed by the addition of 0.3 mL acetone RPE. The mixture was heated at 150°C under microwave irradiation (100 W) for 15 min and vigorous magnetic stirring (600 rpm). After cooling to room temperature, a second cycle was carried out under the same conditions. The reaction mixture was then allowed to cool to room temperature and then acidified to pH < 6.5 using aqueous HCl (37%), affording the title compound 2 as a white solid. The resulting solid was collected by vacuum filtration, washed with cold water (10 mL) and dried in a desiccator over silica gel.

White solid. Yield: 93%; m.p.: 245°C–247°C. ^1^H NMR (300 MHz, DMSO‐d_6_) *δ*: 3.00 (s, 3H, CH₃), 7.90 (t, 1H, *J* = *7.5 Hz*, 6‐CH_quin_), 8.08 (t, 1H, *J* = *7.5 Hz*, 7‐CH_quin_), 8.21 (s, 1H, 3‐CH_quin_), 8.50 (d, 1H, *J* = *8.4 Hz*, 8‐CH_quin_), 8.66 (d, 1H, *J* = *8.4 Hz*, 5‐CH_quin_), 14.20 (b s, 1H, OH_carboxylic acid_); ^13^C APT NMR (75 MHz, DMSO‐d_6_) *δ*: 21.46 (CH₃), 122.49 (CH‐3_quin_), 123.84 (C‐4a_quin_), 124.57 (CH‐6_quin_), 126.65 (CH‐5_quin_), 129.80 (CH‐8_quin_), 133.66 (CH‐7_quin_), 140.59 (C‐4_quin_), 143.09 (C‐8a_quin_), 159.02 (C‐2_quin_), 166.40 (HO–C═O). Anal calcd for C_11_H_9_NO_2_: C, 70.58; H, 4.85; N, 7.48. Found: C, 70.50; H, 4.68; N, 7.35.

#### 4.1.3 General Procedure for the Synthesis of Substituted 2‐Methyl‐*N*‐Phenylquinoline‐4‐Carboxamide (3a–j)

The synthesis of substituted 2‐methyl‐*N*‐phenylquinoline‐4‐carboxamide (**3a–j**) was performed by direct amidation between 2‐methylquinoline‐4‐carboxylic acid (2) (1.0 equiv) and the suitable substituted aniline (1.1 equiv) in anhydrous acetonitrile (1 mL/mmol), using *N*‐ethyl‐*N*‐(3‐dimethylaminopropyl)carbodiimide hydrochloride (EDC·HCl) (1.1 equiv), hydroxybenzotriazole (HOBt) (0.2 equiv), and 4‐dimethylaminopyridine (DMAP) (2.0 equiv). After complete depletion of the starting material (Table 2), water was added to the reaction mixture causing the formation of a precipitate that was vacuum‐filtered, washed with cold water (10 mL), and dried in a stove. The crude substituted 2‐methyl‐*N*‐phenylquinoline‐4‐carboxamide (**3a–j**) were purified by crystallization from ethanol giving yields ranging from 12.6% to 78.3% (Table 2).

The same reaction was performed in a 30 mL microwave reaction tube equipped with a magnetic stir bar, where 2‐methylquinoline‐4‐carboxylic acid (2) (1.0 equiv) was dissolved in anhydrous acetonitrile (1 mL/mmol), then EDC·HCl (2.0 equiv), HOBt (0.2 equiv), DMAP (2.0 equiv) and the appropriately substituted aniline (1.1 equiv) were added. The tube was sealed and heated at 50°C under microwave irradiation (60 W) for 20 min and vigorous magnetic stirring (600 rpm). After cooling to room temperature, a second cycle was carried out under the same conditions. Upon completion, the reaction mixture was allowed to cool at room temperature, and water was added. The resulting precipitate was collected by vacuum filtration, washed with cold water (10 mL) and dried in a stove. Crystallization from ethanol resulted in the substituted 2‐methyl‐*N*‐phenylquinoline‐4‐carboxamides (**3a–j**) in yields ranging from 45.2% to 83.4% (Table 2).

Yields and characterizations reported below are related to microwave‐assisted synthesis.

##### 4.1.3.1 2‐Methyl‐*N*‐Phenylquinoline‐4‐Carboxamide (3a)

White solid; yield: 83%; m.p: 197°C–198°C. ^1^H NMR (300 MHz, DMSO‐d_6_) *δ*: 2.74 (s, 3H, CH₃_quin_), 7.17 (t, 1H, *J* **=** *6.9 Hz*, 4′‐CH_phenyl_), 7.41 (t, 2H, *J* **=** *7.4 Hz*, 3′/5′‐CH_phenyl_), 7.59–7.64 (m, 2H, 2′/6′‐CH_phenyl_), 7.79–7.82 (m, 3H, 3/6/7‐CH_quinl_), 8.02–8.11 (m, 2H, 5/8‐CH_quinl_), 10.78 (s, 1H, NH–C**═**O); ^13^C APT NMR (75 MHz, DMSO‐d_6_) *δ*: 25.28 (CH₃_quin_), 120.37 (CH‐2′/6′_phenyl_), 122.86 (C‐4a_quin_), 124.59 (CH‐3_quin_), 125.45 (CH‐5_quin_), 127.05 (CH‐4′_phenyl_), 129.19 (CH‐6_quin_), 129.31 (CH‐3′/5′_phenyl_, CH‐8_quin_), 130.29 (CH‐7_quin_), 139.25 (C‐1′_phenyl_), 142.60 (C‐4_quin_), 148.11 (C‐8a_quin_), 159.08 (C−2_quin_), 165.84 (HN–C**═**O). Anal calcd for C_17_H_14_N_2_O: C, 77.84; H, 5.38; N, 10.68. Found: C, 77.74; H, 5.27; N, 10.79.

##### 4.1.3.2 2‐Methyl‐*N*‐(3‐Methylphenyl)Quinoline‐4‐Carboxamide (3b)

White solid; yield: 81%; m.p: 174°C–175°C. ^1^H NMR (300 MHz, DMSO‐d_6_) *δ*: 2.33 (s, 3H, 3′‐CH₃_phenyl_), 2.75 (s, 3H, CH₃_quin_), 6.99 (d, 1H, *J* **=** *7.2 Hz*, 4′‐CH_phenyl_), 7.28 (t, 1H, *J* **=** *7.7 Hz*, 5′‐CH_phenyl_), 7.56 (d, 1H, *J* **=** *8.1 Hz*, 6′‐CH_phenyl_), 7.61–7.66 (m, 3H, 3/6‐CH_quin_, 2′‐CH_phenyl_), 7.81 (t, 1H, *J* **=** *7.4 Hz*, 7‐CH_quin_), 8.03–8.10 (m, 2H, 5/8‐CH_quin_), 10.69 (s, 1H, NH–C**═**O); ^13^C APT NMR (75 MHz, DMSO‐d_6_) *δ*: 21.67 (3′‐CH₃_phenyl_), 25.01 (CH₃_quin_), 117.64 (CH‐6′_phenyl_), 120.41 (CH‐2′_phenyl_), 120.94 (CH‐3_quin_), 122.94 (C‐4a_quin_), 125.31 (CH‐5_quin_), 125.52 (CH‐6_quin_), 127.22 (CH‐4′_phenyl_), 128.68 (CH‐8_quin_), 129.12 (CH‐5′_phenyl_), 130.54 (CH‐7_quin_), 138.52 (C‐1′_phenyl_), 139.12 (C‐3′_phenyl_), 143.11 (C‐4_quin_), 147.50 (C‐8a_quin_), 159.04 (C‐2_quin_), 165.62 (HN‐C** =** O). Anal calcd for C_18_H_16_N_2_O: C, 78.24; H, 5.84; N, 10.14. Found: C, 78.09; H, 5.71; N, 10.21.

##### 4.1.3.3 2‐Methyl‐*N*‐(4‐Methylphenyl)Quinoline‐4‐Carboxamide (3c)

White solid; yield: 68%; m.p: 172°C–173°C. ¹H NMR (300 MHz, DMSO‐d₆) *δ*: 2.29 (s, 3H, 4′‐CH₃_phenyl_), 2.72 (s, 3H, CH₃_quin_), 7.21–8.08 (m, 5H_quin_, 4H_phenyl_), 10.70 (s, 1H, NH–C**═**O); ¹³C APT NMR (75 MHz, DMSO‐d₆) δ: 20.95 (4′‐CH₃_phenyl_), 24.97 (CH₃_quin_), 120.33 (CH‐3_quin_), 120.49 (CH‐2′/6′_phenyl_), 122.88 (C‐4a_quin_), 125.41 (CH‐5_quin_), 127.09 (CH‐6_quin_), 129.03 (CH‐8_quin_), 129.70 (CH‐3′/5′_phenyl_), 130.37 (CH‐7_quin_), 133.81 (C‐4′_phenyl_), 136.59 (C‐1′_phenyl_), 142.65 (C‐4_quin_), 147.97 (C‐8a_quin_), 159.19 (C‐2_quin_), 165.70 (HN–C**═**O). Anal calcd for C_18_H_16_N_2_O: C, 78.24; H, 5.84; N, 10.14. Found: C, 78.38; H, 5.99; N, 10.01.

##### 4.1.3.4 *N*‐(2,6‐Dimethylphenyl)‐2‐Methylquinoline‐4‐Carboxamide (3d)

White solid; yield: 65%; m.p: 181°C–182°C. ¹H NMR (300 MHz, DMSO‐d₆) *δ*: 2.33 (s, 6H, 2′/6′‐CH₃_phenyl_), 2.79 (s, 3H, CH₃_quin_), 7.17 (b s, 3H, 3′/4′/5′‐CH_phenyl_), 7.66–7.72 (m, 2H, 3/6‐CH_quin_), 7.84 (t, 1H, *J* **=** *7.5 Hz*, 7‐CH_quin_), 8.07 (d, 1H, *J* **=** *8.4 Hz*, 8‐CH_quin_), 8.20 (d, 1H, *J* **=** *8.4 Hz*, 5‐CH_quin_), 10.20 (s, 1H, NH–C**═**O). ¹³C APT NMR (75 MHz, DMSO‐d₆) δ: 18.73 (2′/6′‐CH₃_phenyl_), 24.95 (CH₃_quin_), 120.50 (CH‐3_quin_), 123.20 (C‐4a_quin_), 125.56 (CH‐5_quin_), 127.39 (CH‐6_quin_), 127.49 (CH‐4′_phenyl_), 127.49 (CH‐8_quin_), 128.38 (CH‐3′/5′_phenyl_), 130.77 (CH‐7_quin_), 134.82 (C‐1′_phenyl_), 135.85 (C‐2′/6′_phenyl_), 143.55 (C‐4_quin_), 147.15 (C‐8a_quin_), 159.05 (C‐2_quin_), 165.66 (HN–C**═**O). Anal calcd for C_19_H_18_N_2_O: C, 78.59; H, 6.25; N, 9.65. Found: C, 78.45; H, 6.37; N, 9.73.

##### 4.1.3.5 2‐Methyl‐*N*‐(3,4,5‐Trimethoxyphenyl)Quinoline‐4‐Carboxamide (3e)

White solid; yield: 59%; m.p: 223°C–224°C. ¹H NMR (300 MHz, DMSO‐d₆) *δ*: 2.74 (s, 3H, CH₃_quin_), 3.67 (s, 3H, 4′‐OCH₃_phenyl_), 3.79 (s, 6H, 3′/5′‐OCH₃_phenyl_), 7.22 (s, 2H, 2′/6′‐CH_phenyl_), 7.62 (b s, 2H, 3/6‐CH_quin_), 7.80 (m, 1H, 7‐CH_quin_), 8.01–8.12 (m, 2H, 5/8‐CH_quin_), 10.70 (s, 1H, NH–C**═**O); ¹³C APT NMR (75 MHz, DMSO‐d₆) *δ*: 25.27 (CH₃_quin_), 56.22 (3′/5′‐OCH₃_phenyl_), 60.62 (4′‐OCH₃_phenyl_), 98.11 (CH‐2′/6′_phenyl_), 120.32 (CH‐3_quin_), 122.83 (C‐4a_quin_), 125.48 (CH‐5_quin_), 127.05 (CH‐6_quin_), 129.17 (CH‐8_quin_), 130.30 (CH‐7_quin_), 134.50 (C‐1′_phenyl_), 135.40 (C‐4′_phenyl_), 142.48 (C‐4_quin_), 148.10 (C‐8a_quin_), 153.24 (C‐3′/5′_phenyl_), 159.04 (C‐2_quin_), 165.65 (HN–C**═**O). Anal calcd for C_20_H_20_N_2_O_4_: C, 68.17; H, 5.72; N, 7.95. Found: C, 68.31; H, 5.88; N, 7.80.

##### 4.1.3.6 *N*‐(3‐Chlorophenyl)‐2‐Methylquinoline‐4‐Carboxamide (3f)

White solid; yield: 69%; m.p: 218°C–219°C. ¹H NMR (300 MHz, DMSO‐d₆) *δ*: 2.78 (s, 3H, CH₃_quin_), 7.24 (d, 1H, *J* **=** *7.8 Hz*, 4′‐CH_phenyl_), 7.44 (t, 1H, *J* **=** *8.1 Hz*, 5′‐CH_phenyl_), 7.66–7.74 (m, 3H, 2′/6′‐CH_pheny_, 6‐CH_quin_), 7.85 (t, 1H, *J* **=** *7.5 Hz*, 7‐CH_quin_), 8.00–8.13 (m, 3H, 3,5,8‐CH_quin_), 11.00 (s, 1H, NH–C**═**O); ¹³C APT NMR (75 MHz, DMSO‐d₆) δ: 24.71 (CH₃_quin_), 118.87 (CH‐6′_phenyl_), 119.96 (CH‐2′_phenyl_), 120.71 (CH‐3_quin_), 122.86 (C‐4a_quin_), 124.43 (CH‐5_quin_), 125.58 (CH‐6_quin_), 127.60 (CH‐4′_phenyl_), 128.11 (CH‐8_quin_), 131.01 (CH‐7_quin_), 131.05 (CH‐5′_phenyl_), 133.62 (C‐3′_phenyl_), 140.55 (C‐1′_phenyl_), 143.11 (C‐4_quin_), 146.80 (C‐8a_quin_), 159.05 (C‐2_quin_), 165.76 (HN–C**═**O). Anal calcd for C_17_H_13_ClN_2_O: C, 68.81; H, 4.42; N, 9.44. Found: C, 68.96; H, 4.56; N, 9.29.

##### 4.1.3.7 *N*‐(4‐Chlorophenyl)‐2‐Methylquinoline‐4‐Carboxamide (3g)

White solid; yield: 65%; m.p: 221°C–222°C. ¹H NMR (300 MHz, DMSO‐d₆) δ: 2.73 (s, 3H, CH₃_quin_), 7.48 (d, 2H, *J* **=** *8.4 Hz*, 3′/5′‐CH_phenyl_), 7.59–7.66 (m, 2H, 2′/6′‐CH_phenyl_), 7.77–7.85 (m, 3H, 3,6,7‐CH_quin_), 8.02–8.09 (m, 2H, 5,8‐CH_quin_), 10.92 (s, 1H, NH–C**═**O); ¹³C APT NMR (75 MHz, DMSO‐d₆) *δ*: 25.27 (CH₃_quin_), 120.41 (CH‐3_quin_), 121.92 (CH‐2′/6′_phenyl_), 122.76 (C‐4a_quin_), 125.40 (CH‐5_quin_), 127.12 (CH‐6_quin_), 129.24 (CH‐8_quin_, CH‐3′/5′_phenyl_), 128.20 (C‐4′_phenyl_), 130.34 (CH‐7_quin_), 138.20 (C‐1′_phenyl_), 142.27 (C‐4_quin_), 148.10 (C‐8a_quin_), 159.07 (C‐2_quin_), 165.92 (HN–C**═**O). Anal calcd for C_17_H_13_ClN_2_O: C, 68.81; H, 4.42; N, 9.44. Found: C, 68.98; H, 4.27; N, 9.59.

##### 4.1.3.8 2‐Methyl‐*N*‐[4‐(Trifluoromethyl)Phenyl]Quinoline‐4‐Carboxamide (3 h)

White solid; yield: 69%; m.p: 246°C–247°C. ¹H NMR (300 MHz, DMSO‐d₆) *δ*: 2.74 (s, 3H, CH₃_quin_), 7.59–7.82 (m, 5H, 2′/6′‐CH_phenyl_, 3,6,7‐CH_quin_), 8.00–8.10 (m, 4H, 3′/5′‐CH_phenyl_, 5,8‐CH_quin_), 11.11 (s, 1H, NH–C**═**O); ¹³C APT NMR (75 MHz, DMSO‐d₆) *δ*: 25.27 (CH₃_quin_), 120.36–120.46 (CH‐2′/6′_phenyl_, CH‐3_quin_), 121.18 (q, *J* **=** *270.0 Hz*, CF_3_), 122.69 (C‐4a_quin_), 124.61 (q, *J* **=** *31.6 Hz*, 4′‐C_phenyl_‐CF_3_), 125.33 (CH‐5_quin_), 126.63 (b q, CH‐3′/5′_phenyl_), 127.18 (CH‐6_quin_), 129.24 (CH‐8_quin_), 130.36 (CH‐7_quin_), 142.03 (C‐1′_phenyl_), 142.78 (C‐4_quin_), 148.12 (C‐8a_quin_), 159.06 (C‐2_quin_), 166.34 (HN–C**═**O). Anal calcd for C_18_H_13_F_3_N_2_O: C, 65.45; H, 3.97; N, 8.48. Found: C, 65.61; H, 4.11; N, 8.65.

##### 4.1.3.9 2‐Metyl‐*N*‐(4‐Nitrophenyl)Quinoline‐4‐Carboxamide (3i)

White solid; yield: 55%; m.p: 280°C–281°C. ¹H NMR (300 MHz, DMSO‐d₆) *δ*: 2.74 (s, 3H, CH₃_quin_), 7.63 (t, 1H, *J* **=** *7.7 Hz*, 6‐CH_quin_), 7.71 (s, 1H, 3‐CH_quin_), 7.81 (t, 1H, *J* **=** *7.5 Hz*, 7‐CH_quin_), 8.03–8.10 (m, 4H, 2′/6′‐CH_phenyl_, 3′/5′‐CH_phenyl_), 8.30–8.33 (m, 2H, 5,8‐CH_quin_), 11.34 (s, 1H, NH–C**═**O); ¹³C APT NMR (75 MHz, DMSO‐d₆) *δ*: 25.27 (CH₃_quin_), 120.23 (CH‐2′/6′_phenyl_), 120.57 (CH‐3_quin_), 122.59 (C‐4a_quin_), 125.27 (CH‐5_quin_), 125.44 (CH‐3′/5′_phenyl_), 127.28 (CH‐6_quin_), 129.26 (CH‐8_quin_), 130.43 (CH‐7_quin_), 141.71 (C‐4_quin_), 143.38 (C‐1′_phenyl_), 145.29 (C‐4′_phenyl_), 148.11 (C‐8a_quin_), 159.07 (C‐2_quin_), 166.55 (HN–C**═**O). Anal calcd for C_17_H_13_N_3_O_3_: C, 66.44; H, 4.26; N, 13.67. Found: C, 66.28; H, 4.11; N, 13.81.

##### 4.1.3.10 2‐Methyl‐*N*‐[4‐Nitro‐3‐(Trifluoromethyl)Phenyl]Quinoline‐4‐Carboxamide (3j)

Yellow solid; yield: 45%; m.p: 257°C–259°C. ¹H NMR (300 MHz, DMSO‐d₆) *δ*: 2.75 (s, 3H, CH₃_quin_), 7.63 (t, 1H, *J* **=** *7.5 Hz*, 6‐CH_quin_), 7.74 (s, 1H, 3‐CH_quin_), 7.81 (t, 1H, *J* **=** *7.7 Hz*, 7‐CH_quin_), 8.05 (d, 1H, *J* **=** *8.1 Hz*, 8‐CH_quin_), 8.15 (d, 1H, *J* **=** *8.1 Hz*, 5‐CH_quin_), 8.26–8.29 (m, 2H, 5′/6′‐CH_phenyl_), 8.47 (s, 1H, 2′‐CH_phenyl_), 11.53 (s, 1H, NH–C**═**O); ¹³C APT NMR (75 MHz, DMSO‐d₆) *δ*: 25.28 (CH₃_quin_), 118.68 (q, *J* **=** *5.2 Hz*, 2′‐CH_phenyl_), 120.69 (6′‐CH_phenyl_), 122.50 (C‐4a_quin_), 122.51 (q, *J* **=** *271.4 Hz*, CF_3_), 123.36 (q, *J* **=** *32.8 Hz*, 3′‐C_phenyl_‐CF_3_), 123.75 (CH‐3_quin_), 125.34 (CH‐5_quin_), 127.31 (CH‐7_quin_), 128.11 (CH‐5′_phenyl_), 129.28 (CH‐8_quin_), 130.47 (CH‐7_quin_), 141.19 (C‐4_quin_), 142.54 (C‐4′_phenyl_), 143.78 (C‐1′_phenyl_), 148.17 (C‐8a_quin_), 159.02 (C‐2_quin_), 166.75 (HN–C**═**O). Anal calcd for C_18_H_12_F_3_N_3_O_3_: C, 57.61; H, 3.22; N, 11.20. Found: C, 57.76; H, 3.06; N, 11.12.

#### 4.1.4 General Procedure for the Synthesis of Substituted *N*‐Phenyl‐2‐[(*E*)‐2‐Phenylethen‐1‐yl]Quinoline‐4‐Carboxamides (4a–z and 4aa‐ah)

In a 30 mL microwave reaction tube equipped with a magnetic stir bar, the appropriate substituted 2‐methyl‐*N*‐phenylquinoline‐4‐carboxamide (**3a–j**) (1.0 equiv) was dissolved in glacial acetic acid (1.5 mL/mmol). An appropriately substituted benzaldehyde (1.0 equiv) or 3,4,5‐trimethoxybenzaldehyde (2.0 equiv) was then added to the solution. The tube was sealed and heated at 150°C under microwave irradiation (200 W) for 20 min and vigorous magnetic stirring (600 rpm). After cooling to room temperature, a second cycle was carried out under the same conditions. Upon completion, the reaction mixture was allowed to cool at room temperature, and water was added. The resulting precipitate was collected by vacuum filtration, washed with cold water (10 mL) and dried in a stove. Crude substituted *(E)‐N‐*phenyl‐2‐styrylquinoline‐4‐carboxamides (**4a–z** and **4aa–ah**) were purified by crystallization from ethanol or by MPLC.

##### 4.1.4.1 *(E)‐N*‐Phenyl‐2‐Styrylquinoline‐4‐Carboxamide (4a)

Purified by MPLC (ethyl acetate/cyclohexane, 1:1). White solid; yield: 64%; m.p.: 230°C–231°C. ¹H NMR (300 MHz, DMSO‐d₆) *δ*: 7.19 (t, 1H, *J* = *6.9 Hz*, 4′‐CH_phenyl_), 7.39‐7.47 (m, 5H, 2″/3″/4″/5″/6″‐CH_styryl_), 7.56–7.67 (m, 2H, CH═CH‐Ph, 6‐CH_quin_), 7.78–7.87 (m, 5H, 2′/3′/5′/6′‐CH_phenyl_, 7‐CH_quinl_), 8.00 (d, 1H, *J* = *16.5 Hz*, CH=CH‐Ph), 8.11–8.14 (m, 3H, 3/5/8‐CH_quin_), 10.86 (s, 1H, NH–C═O); ¹³C APT NMR (75 MHz, DMSO‐d₆) *δ*: 118.05 (CH‐5_quin_), 120.44 (2′/6′‐CH_phenyl_), 123.66 (C‐4a_quin_), 124.63 (CH‐3_quin_), 125.54 (4′‐CH_phenyl_), 127.57 (CH‐6_quin_), 127.82 (2″/6″‐CH_styryl_), 128.75 (CH═CH‐Ph), 129.33–129.40 (CH‐7_quin_, 3′/5′‐CH_phenyl_, 3″/5″‐CH_styryl_), 129.67 (CH‐8_quin_), 130.69 (4″‐CH_styryl_), 135.50 (CH═CH‐Ph), 136.60 (C‐1″_styryl_), 139.31 (C‐1′_phenyl_), 143.09 (C‐4_quin_), 148.49 (C‐8a_quin_), 155.97 (C‐2_quin_), 165.85 (NH–C═O). Anal calcd for C_24_H_18_N_2_O: C, 82.26; H, 5.18; N, 7.99. Found: C, 82.41; H, 5.03; N, 8.11. ESI‐MS analysis for [C_24_H_18_N_2_O + H^+^]: Calc.: 351.1492 *m*/*z*, exp.: 351.1494 *m*/*z*. Purity: 99.58%.

##### 4.1.4.2 *(E)‐*2‐Styryl‐*N*‐(m‐Tolyl)Quinoline‐4‐Carboxamide (4b)

Purified by crystallization from ethanol. White solid; yield: 68%; m.p.: 197°C–198°C. ¹H NMR (300 MHz, DMSO‐d₆) *δ*: 2.35 (s, 3H, 3′‐CH₃_phenyl_), 7.00 (d, 1H, *J* = *7.5 Hz*, 4′‐CH_phenyl_), 7.27–7.49 (m, 4H, 2′‐CH_phenyl_, 3″/4″/5″‐CH_styryl_), 7.54–7.69 (m, 4H, CH**═**CH‐Ph, 5′‐CH_phenyl_, 2″/6″‐CH_styryl_), 7.76–7.85 (m, 3H, 6′‐CH_phenyl_, 6/7‐CH_quin_), 7.97 (d, 1H, *J* = *16.5 Hz*, CH**═**CH‐Ph), 8.08–8.11 (m, 3H, 3/5/8‐CH_quin_), 10.75 (s, 1H, NH–C**═**O); ¹³C APT NMR (75 MHz, DMSO‐d₆) *δ*: 21.69 (3′‐CH₃_phenyl_), 117.64 (6′‐CH_phenyl_), 117.98 (2′‐CH_phenyl_), 120.94 (CH‐5_quin_), 123.65 (C‐4a_quin_), 125.31 (CH‐3_quin_), 125.53 (CH‐6_quin_), 127.55 (4′‐CH_phenyl_), 127.81 (2″/6″‐CH_styryl_), 128.73 (CH**═**CH‐Ph), 129.14 (CH‐7_quin_), 129.39 (CH‐8_quin_, 3″/5″‐CH_styryl_), 129.64 (5′‐CH_phenyl_), 130.68 (4″‐CH_styryl_), 135.47 (CH**═**CH‐Ph), 136.59 (C‐1″_styryl_), 138.54 (C‐1′_phenyl_), 139.20 (C‐3′_phenyl_), 143.13 (C‐4_quin_), 148.45 (C‐8a_quin_), 155.94 (C‐2_quin_), 165.78 (NH–C**═**O). Anal calcd for C_25_H_20_N_2_O: C, 82.39; H, 5.53; N, 7.69. Found: C, 82.52; H, 5.68; N, 7.51. ESI‐MS analysis for [C_25_H_20_N_2_O + Na^+^]: Calc.: 387.146 *m*/*z*, exp.: 387.1467 *m*/*z*. Purity: 98.21%.

##### 4.1.4.3 *(E)‐*2‐Styryl‐*N*‐(p‐Tolyl)Quinoline‐4‐Carboxamide (4c)

Purified by crystallization from ethanol. White solid; yield: 91%; m.p.: 196°C–197°C. ¹H NMR (300 MHz, DMSO‐d₆) *δ*: 2.32 (s, 3H, 4′‐CH₃_phenyl_), 7.23–7.24 (m, 2H, 3′/5′‐CH_phenyl_), 7.39–7.80 (m, 10H, 2′6′‐CH_phenyl_, 2″/3″/4″/5″/6″‐CH_styryl_, CH**═**CH‐Ph, 6/7‐CH_quin_), 8.00 (d, 1H, *J* **=** *16.2 Hz*, CH**═**CH‐Ph), 8.10–8.13 (m, 3H, 3/5/8‐CH_quin_), 10.79 (s, 1H, NH–C**═**O); ¹³C APT NMR (75 MHz, DMSO‐d₆) *δ*: 21.02 (4′‐CH₃_phenyl_), 118.00 (CH‐5_quin_), 120.38 (2′/6′‐CH_phenyl_), 123.67 (C‐4a_quin_), 125.57 (CH‐3_quin_), 127.54 (CH‐6_quin_), 127.82 (2″/6″‐CH_styryl_), 128.74 (CH**═**CH‐Ph), 129.40 3″/5″‐CH_styryl_), 129.69 (CH‐8_quin_, 3′/5′‐CH_phenyl_), 130.68 (4″‐CH_styryl_), 133.61 (C‐4′_phenyl_), 135.46 (CH**═**CH‐Ph), 136.59 (C‐1″_styryl_), 136.82 (C‐1′_phenyl_), 143.16 (C‐4_quin_), 148.47 (C‐8a_quin_), 155.96 (C‐2_quin_), 165.62 (NH–C**═**O). Anal calcd for C_25_H_20_N_2_O: C, 82.39; H, 5.53; N, 7.69. Found: C, 82.54; H, 5.40; N, 7.53. ESI‐MS analysis for [C_25_H_20_N_2_O** +** H^+^]: Calc.: 365.1648 *m*/*z*, exp.: 365.1647 *m*/*z*. Purity: 99.17%.

##### 4.1.4.4 *(E)‐N*‐(2,6‐dimethylphenyl)‐2‐styrylquinoline‐4‐carboxamide (4d)

Purified by MPLC (ethyl acetate/cyclohexane, 1:1). White solid; yield: 83%; m.p.: 226°C–227°C. ¹H NMR (300 MHz, DMSO‐d₆) *δ*: 2.39 (s, 6H, 2′/6′‐CH₃_phenyl_), 7.19 (b s, 3H, 3′/4′/5′‐CH_phenyl_), 7.37–7.50 (m, 3H, 3″/4″/5″‐CH_styryl_), 7.63–7.88 (m, 5H, 2″/6″‐CH_styryl_, CH**═**CH‐Ph, 6/7‐CH_quin_), 8.01 (d, 1H, *J* **=** *16.5 Hz*, CH**═**CH‐Ph), 8.11–8.24 (m, 3H, 3/5/8‐CH_quin_), 10.27 (s, 1H, NH–C**═**O); ¹³C APT NMR (75 MHz, DMSO‐d₆) *δ*: 18.84 (2′/6′‐CH₃_phenyl_), 118.55 (CH‐5_quin_), 123.96 (C‐4a_quin_), 125.61 (CH‐3_quin_), 127.48 (CH‐6_quin_), 127.70 (4′‐CH_phenyl_), 127.93 (2′/6′‐C_phenyl_), 128.18 (CH**═**CH‐Ph), 128.41 (3′/5′‐CH_phenyl_), 129.31 (CH‐7_quin_), 129.41 (3″/5″‐CH_styryl_), 129.50 (CH‐8_quin_), 130.94 (4″‐CH_styryl_), 134.88 (C‐1″_styryl_), 135.73 (CH**═**CH‐Ph), 135.87 (2″/6″‐CH_styryl_), 136.51 (C‐1′_phenyl_), 143.61 (C‐4_quin_), 148.03 (C‐8a_quin_), 155.59 (C‐2_quin_), 165.76 (NH–C**═**O). Anal calcd for C_26_H_22_N_2_O: C, 82.51; H, 5.86; N, 7.40. Found: C, 82.69; H, 5.98; N, 7.54. ESI‐MS analysis for [C_26_H_22_N_2_O** +** H^+^]: Calc.: 379.1805 *m*/*z*, exp.: 379.1805 *m*/*z*. Purity: 98.35%.

##### 4.1.4.5 *(E)‐*2‐Styryl‐*N*‐(3,4,5‐Trimethoxyphenyl)Quinoline‐4‐Carboxamide (4e)

Purified by MPLC (ethyl acetate/cyclohexane, 1:1). White solid; yield: 56%; m.p.: 216°C–217°C. ¹H NMR (300 MHz, DMSO‐d₆) *δ*: 3.69 (s, 3H, 4′‐OCH₃_phenyl_), 3.80 (s, 6H, 3′/5′‐OCH₃_phenyl_), 7.25–7.66 (m, 7H, 2′/6′‐CH_phenyl_, 2″/3″/4″/5″/6″‐CH_styryl_), 7.77–7.85 (m, 3H, CH**═**CH‐Ph, 6/7‐CH_quin_), 7.98 (d, 1H, *J* **=** *16.2 Hz*, CH**═**CH‐Ph), 8.09–8.14 (m, 3H, 3/5/8‐CH_quin_), 10.75 (s, 1H, NH–C**═**O); ¹³C APT NMR (75 MHz, DMSO‐d₆) *δ*: 56.30 (3′/5′‐OCH₃_phenyl_), 60.66 (4′‐OCH₃_phenyl_), 98.31 (2′/6′‐CH_phenyl_), 118.05 (CH‐5_quin_), 123.62 (C‐4a_quin_), 125.57 (CH‐3_quin_), 127.56 (CH‐6_quin_), 127.82 (2″/6″‐C_styryl_), 128.72 (CH**═**CH‐Ph), 129.40 (3′/5′‐CH_phenyl_, CH‐7_quinl_), 129.66 (CH‐8_quin_), 130.70 (4″‐CH_styryl_), 134.69 (C‐1′_phenyl_), 135.42 (C‐1″_styryl_), 135.49 (CH**═**CH‐Ph), 136.58 (4′‐C_phenyl_), 142.99 (C‐4_quin_), 148.49 (C‐8a_quin_), 153.29 (3′/5′‐CH_phenyl_), 155.92 (C‐2_quin_), 165.65 (NH–C**═**O). Anal calcd for C_27_H_24_N_2_O_4_: C, 73.62; H, 5.49; N, 6.36. Found: C, 73.77; H, 5.63; N, 6.22. ESI‐MS analysis for [C_27_H_24_N_2_O_4_ **+** Na^+^]: Calc.: 463.1623 *m*/*z*, exp.: *m*/*z;* 463.1622 *m*/*z*. Purity: 99.74%.

##### 4.1.4.6 *(E)‐N*‐(3‐Chlorophenyl)‐2‐Styrylquinoline‐4‐Carboxamide (4f)

Purified by MPLC (ethyl acetate/cyclohexane, 1:1). White solid; yield: 85%; m.p.: 237°C–238°C. ¹H NMR (300 MHz, DMSO‐d₆) δ: 7.24–7.26 (m, 1H, 4′‐CH_phenyl_), 7.36‐7.48 (m, 4H, 6′‐CH_phenyl_, 3″/4″/5″‐CH_styryl_), 7.54–7.73 (m, 3H, 5′‐CH_phenyl_, 2″/6″‐CH_styryl_), 7.76–7.86 (m, 3H, CH**═**CH‐Ph, 6/7‐CH_quin_), 7.98 (d, 1H, *J* **=** *16.5 Hz*, CH**═**CH‐Ph), 8.04 (b s, 1H, 2′‐CH_phenyl_), 8.09–8.15 (m, 3H, 3/5/8‐CH_quin_), 11.02 (s, 1H, NH–C**═**O); ¹³C APT NMR (75 MHz, DMSO‐d₆) *δ*: 118.09 (6′‐CH_phenyl_), 118.84 (2′‐CH_phenyl_), 119.92 (CH‐5_quin_), 123.48 (C‐4a_quin_), 124.37 (CH‐3_quin_), 125.48 (4′‐CH_phenyl_), 127.67 (CH‐6_quin_), 127.82 (2″/6″‐CH_styryl_), 128.69 (CH**═**CH‐Ph), 129.41 (3″/5″‐C_styryl_, CH‐8_quin_), 129.68 (CH‐7_quin_), 130.77 (4″‐CH_styryl_), 131.06 (5′‐CH_phenyl_), 133.65 (3′‐C_phenyl_), 135.59 (CH**═**CH‐Ph), 136.56 (1″‐C_styryl_), 140.68 (1′‐C_phenyl_), 142.59 (C‐4_quin_), 148.47 (C‐8a_quin_), 155.96 (C‐2_quin_), 166.11 (NH–C**═**O). Anal calcd for C_24_H_17_N_2_O: C, 74.90; H, 4.45; N, 7.28. Found: C, 75.02; H, 4.31; N, 7.33. ESI‐MS analysis for [C_24_H_17_ClN_2_O** +** H^+^]: Calc.: 385.1102 *m*/*z*, exp.: 385.1102 *m*/*z*. Purity: 98.49%.

##### 4.1.4.7 *(E)‐N*‐(4‐Chlorophenyl)‐2‐Styrylquinoline‐4‐Carboxamide (4g)

Purified by flash column chromatography (EtOAc/Cyclohexane, 1:1). White solid; yield: 69%; m.p.: 239°C–240°C. ¹H NMR (300 MHz, DMSO‐d₆) δ: 7.35–7.40 (m, 1H, 4″‐CH_styryl_), 7.44–7.50 (m, 4H, 2″/3″/5″/6″‐CH_styryl_), 7.54–7.66 (m, 2H, 3′/5′‐CH_phenyl_), 7.76–7.88 (m, 5H, 2′/6′‐CH_phenyl_, CH**═**CH‐Ph, 6/7‐CH_quin_), 7.98 (d, 1H, *J* **=** *16.5 Hz*, CH**═**CH‐Ph), 8.08–8.14 (m, 3H, 3/5/8‐CH_quin_), 10.97 (s, 1H, NH–C**═**O); ¹³C APT NMR (75 MHz, DMSO‐d₆) *δ*: 118.07 (CH‐5_quin_), 121.98 (2′/6′‐CH_phenyl_), 123.53 (C‐4a_quin_), 125.48 (CH‐3_quin_), 127.63 (CH‐6_quin_), 127.81 (2″/6″‐CH_styryl_), 128.26 (4′‐C_phenyl_), 128.70 (CH**═**CH‐Ph), 129.26 (3′/5′‐CH_phenyl_), 129.40 (3″/5″‐CH_styryl_, CH‐7_quin_), 129.67 (CH‐8_quin_), 130.74 (4″‐CH_styryl_), 135.56 (CH**═**CH‐Ph), 136.57 (1″‐C_styryl_), 138.23 (1′‐C_phenyl_), 142.75 (C‐4_quin_), 148.47 (C‐8a_quin_), 155.96 (C‐2_quin_), 165.92 (NH–C**═**O). Anal calcd for C_24_H_17_N_2_O: C, 74.90; H, 4.45; N, 7.28. Found: C, 74.76; H, 4.29; N, 7.41. ESI‐MS analysis for [C_24_H_17_ClN_2_O** +** H^+^]: Calc.: 385.1102 *m*/*z*, exp.: 385.1102 *m*/*z*. Purity: 98.32%.

##### 4.1.4.8 *(E)‐*2‐Styryl‐*N*‐(4‐(Trifluoromethyl)Phenyl)Quinoline‐4‐Carboxamide (4h)

Purified by crystallization from ethanol. White solid; yield: 68%; m.p.: 248°C–249°C. ¹H NMR (300 MHz, DMSO‐d₆) *δ*: 7.36–7.41 (m, 1H, 4″‐CH_styryl_), 7.44–7.49 (m, 2H, 3″/5″‐CH_styryl_), 7.54–7.67 (m, 2H, 2″/6″‐CH_styryl_), 7.76–7.86 (m, 5H, 2′/6′‐CH_phenyl_, CH**═**CH‐Ph, 6/7‐CH_quin_), 7.96–8.12 (m, 5H, 3′/5′‐CH_phenyl_, CH**═**CH‐Ph, 5/8‐CH_quin_), 8.18 (s, 1H, 3‐CH_quin_), 11.25 (s, 1H, NH–C**═**O); ¹³C APT NMR (75 MHz, DMSO‐d₆) *δ*: 118.17 (CH‐5_quin_), 120.41 (2′/6′‐CH_phenyl_), 121.20 (q, *J* **=** *270.3 Hz*, CF_3_), 123.45 (C‐4a_quin_), 124.63 (q, *J* **=** *31.6 Hz*, 4′‐C_phenyl_‐CF_3_), 125.43 (CH‐3_quin_), 126.65 (b q, CH‐3′/5′_phenyl_), 127.70 (CH‐6_quin_), 127.82 (2″/6″‐CH_styryl_), 128.68 (CH**═**CH‐Ph), 129.41 (3″/5″‐CH_styryl_, CH‐7_quin_), 129.70 (CH‐8_quin_), 130.78 (4″‐CH_styryl_), 135.61 (CH** =** CH‐Ph), 136.56 (1″‐C_styryl_), 142.50 (1′‐C_phenyl_), 142.85 (C‐4_quin_), 148.47 (C‐8a_quin_), 155.97 (C‐2_quin_), 166.35 (NH–C**═**O). Anal calcd for C_25_H_17_F_3_N_2_O: C, 71.76; H, 4.10; N, 6.70. Found: C, 71.90; H, 4.28; N, 6.55. ESI‐MS analysis for [C_25_H_17_F_3_N_2_O** +** H^+^]: Calc.: 419.1366 *m*/*z*, exp.: *m*/*z;* 419.1366 *m*/*z*. Purity: 98.78%.

##### 4.1.4.9 *(E)‐N*‐(4‐Nitrophenyl)‐2‐Styrylquinoline‐4‐Carboxamide (4i)

Purified by crystallization from ethanol. White solid; yield: 63%; m.p.: 305°C–306°C. ¹H NMR (300 MHz, DMSO‐d₆) δ: 7.39–7.68 (m, 5H, 2″/3″/4″/5″/6″‐CH_styryl_), 7.77–7.87 (m, 3H, CH**═**CH‐Ph, 6/7‐CH_quin_), 7.96–8.13 (m, 5H, 2′/6′‐CH_phenyl_, CH**═**CH‐Ph, 3/8‐CH_quin_), 8.21 (b s, 1H, 5‐CH_quin_), 8.35 (d, 2H, *J* **=** *8.7 Hz*, 3′/5′‐CH_phenyl_), 11.44 (s, 1H, NH–C**═**O); ¹³C APT NMR (75 MHz, DMSO‐d₆) *δ*: 118.24 (CH‐5_quin_), 120.27 (2′/6′‐CH_phenyl_), 123.33 (C‐4a_quin_), 125.37 (CH‐3_quin_), 125.47 (CH‐3′/5′_phenyl_), 127.82 (CH‐6_quin_, 2″/6″‐CH_styryl_), 128.65 (CH**═**CH‐Ph), 129.41 (CH‐7_quin_, 3″/5″‐CH_styryl_), 129.73 (CH‐8_quin_), 130.85 (4″‐CH_styryl_), 135.70 (CH**═**CH‐Ph), 136.54 (1″‐C_styryl_), 142.16 (C‐4_quin_), 143.42 (1′‐C_phenyl_), 145.32 (4′‐C_phenyl_), 148.48 (C‐8a_quin_), 155.97 (C‐2_quin_), 166.56 (NH–C**═**O). Anal calcd for C_24_H_17_N_3_O_3_: C, 72.90; H, 4.33; N, 10.63. Found: C, 72.75; H, 4.18; N, 10.78. ESI‐MS analysis for [C_24_H_17_N_3_O_3_ **+** H^+^]: Calc.: 396.1343 *m*/*z*, exp.: 396.1343 *m*/*z*. Purity: 98.41%.

##### 4.1.4.10 *(E)‐N‐*(4‐Nitro‐3‐(Trifluoromethyl)Phenyl)‐2‐Styrylquinoline‐4‐Carboxamide (4j)

Purified by flash column chromatography (ethyl acetate/cyclohexane, 1:1). Yellow solid; yield: 57%; m.p.: 310°C–311°C. ¹H NMR (300 MHz, DMSO‐d₆) *δ*: 7.39–7.90 (m, 8H, 2″/3″/4″/5″/6″‐CH_styryl_, CH**═**CH‐Ph, 6′‐CH_phenyl_, 6‐CH_quin_), 7.99 (d, 1H, *J* **=** *16.5 Hz*, CH**═**CH‐Ph), 8.13–8.35 (m, 5H, 3/7/8‐CH_quin_, CH‐2′/5′_phenyl_), 8.52 (b s, 1H, 5‐CH_quin_), 11.63 (s, 1H, NH–C**═**O); ¹³C APT NMR (75 MHz, DMSO‐d₆) δ: 118.38 (CH‐5_quin_), 118.72 (b q, CH‐2′_phenyl_), 122.53 (q, *J* **=** *271.7 Hz*, CF_3_), 123.23 (C‐4a_quin_), 123.44 (q, *J* **=** *30.5 Hz*, 3′‐C_phenyl_‐CF_3_), 123.83 (6′‐CH_phenyl_), 125.45 (CH‐3_quin_), 127.85 (CH‐6_quin_, 2″/6″‐CH_styryl_), 128.20 (5′‐CH_phenyl_), 128.62 (CH**═**CH‐Ph), 129.44 (CH‐7_quin_, 3″/5″‐CH_styryl_), 129.75 (CH‐8_quin_), 130.94 (4″‐CH_styryl_), 135.77 (CH**═**CH‐Ph), 136.51 (1″‐C_styryl_), 141.68 (C‐4_quin_), 142.55 (4′‐C_phenyl_), 143.81 (1′‐C_phenyl_), 148.52 (C‐8a_quin_), 155.96 (C‐2_quin_), 166.78 (NH–C**═**O). Anal calcd for C_25_H_16_F_3_N_3_O_3_: C, 64.80; H, 3.48; N, 9.07. Found: C, 64.91; H, 3.53; N, 9.00. ESI‐MS analysis for [C_25_H_16_F_3_N_3_O_3_ **+** Na^+^]: Calc.: 486.1036 *m*/*z*, exp.: 486.1034 *m*/*z*. Purity: 98.85%.

##### 4.1.4.11 *(E)‐N*‐Phenyl‐2‐(3,4,5‐Trimethoxystyryl)Quinoline‐4‐Carboxamide (4k)

Purified by crystallization from ethanol. White solid; yield: 45%; m.p.: 237°C–238°C. ¹H NMR (300 MHz, DMSO‐d₆) *δ*: 3.71 (s, 3H, 4″‐OCH₃_styryl_), 3.87 (s, 6H, 3″/5″‐OCH₃_styryl_), 7.12–7.20 (m, 3H, 2″/6″‐CH_styryl_, 4′‐CH_phenyl_), 7.42 (t, 2H, *J* **=** *7.8 Hz*, 3′/5′‐CH_phenyl_), 7.54–7.65 (m, 2H, CH**═**CH‐Ph, 6‐CH_quin_), 7.76–7.84 (m, 3H, 2′/6′‐CH_phenyl_, 7‐CH_quin_), 7.91 (d, 1H, *J* **=** *16.2 Hz*, CH**═**CH‐Ph), 8.05–8.12 (m, 3H, 3/5/8‐CH_quin_), 10.84 (s, 1H, NH–C**═**O); ¹³C APT NMR (75 MHz, DMSO‐d₆) *δ*: 56.43 (3″/5″‐OCH₃_styryl_), 60.57 (4″‐OCH₃_styryl_), 105.31 (2″/6″‐CH_styryl_), 117.91 (CH‐5_quin_), 120.41 (2′/6′‐CH_phenyl_), 123.55 (C‐4a_quin_), 124.62 (CH‐3_quin_), 125.55 (CH‐4′_phenyl_), 127.45 (CH‐6_quin_), 128.10 (CH‐7_quin_), 129.31 (CH‐3′/5′_phenyl_), 129.54 (CH**═**CH‐Ph), 130.69 (CH‐8_quin_), 132.23 (1″‐C_styryl_), 135.72 (CH**═**CH‐Ph), 138.84 (1′‐C_phenyl_), 139.28 (4″‐C_styryl_), 142.93 (C‐4_quin_), 148.53 (C‐8a_quin_), 153.63 (3″/5″‐C_styryl_), 156.12 (C‐2_quin_), 165.85 (NH–C**═**O). Anal calcd for C_27_H_24_N_2_O_4_: C, 73.62; H, 5.49; N, 6.36. Found: C, 73.77; H, 5.60; N, 6.21. ESI‐MS analysis for [C_27_H_24_N_2_O_4_ **+** Na^+^]: Calc.: 463.1628 *m*/*z*, exp.: 463.1632 *m*/*z*. Purity: 98.03%.

##### 4.1.4.12 *(E)‐N*‐(m‐Tolyl)‐2‐(3,4,5‐Trimethoxystyryl)Quinoline‐4‐Carboxamide (4l)

Purified by flash column chromatography (ethyl acetate/cyclohexane, 1:1). Yellow solid; yield: 97%; m.p.: 237°C–238°C. ¹H NMR (300 MHz, DMSO‐d₆) *δ*: 2.35 (s, 3H, 3′‐CH₃_phenyl_), 3.72 (s, 3H, 4″‐OCH₃_styryl_), 3.88 (s, 6H, 3″/5″‐OCH₃_styryl_), 6.99–7.01 (d, 1H, *J* **=** *7.5 Hz*, 4′‐CH_phenyl_), 7.12 (b s, 2H, 2″/6″‐CH_styryl_), 7.30 (t, 1H, *J* **=** *8.0 Hz*, 5′‐CH_phenyl_), 7.54–7.69 (m, 4H, 2′/6′‐CH_phenyl_, CH**═**CH‐Ph, 6‐CH_quin_), 7.82 (t, 1H, *J* **=** *7.7 Hz*, 7‐CH_quin_), 7.91 (d, 1H, *J* **=** *16.2 Hz*, CH**═**CH‐Ph), 8.05–8.12 (m, 3H, 3/5/8‐CH_quin_), 10.75 (s, 1H, NH–C**═**O); ¹³C APT NMR (75 MHz, DMSO‐d₆) *δ:* 21.68 (3′‐CH₃_phenyl_), 56.43 (3″/5″‐OCH₃_styryl_), 60.48 (4″‐OCH₃_styryl_), 105.32 (2″/6″‐CH_styryl_), 117.64 (6′‐CH_phenyl_), 117.88 (2′‐CH_phenyl_), 120.94 (CH‐5_quin_), 123.58 (C‐4a_quin_), 125.31 (CH‐3_quin_), 125.55 (CH‐6_quin_), 127.43 (CH‐4′_phenyl_), 128.11 (CH‐7_quin_), 129.14 (CH‐5′_phenyl_), 129.53 (CH**═**CH‐Ph), 130.67 (CH‐8_quin_), 132.24 (1″‐C_styryl_), 135.69 (CH**═**CH‐Ph), 138.54 (1′‐C_phenyl_), 138.85 (3′‐C_phenyl_), 139.20 (4″‐C_styryl_), 142.99 (C‐4_quin_), 148.53 (C‐8a_quin_), 153.63 (3″/5″‐C_styryl_), 156.10 (C‐2_quin_), 165.80 (NH–C**═**O). Anal calcd for C_28_H_26_N_2_O_4_: C, 73.99; H, 5.77; N, 6.16. Found: C, 74.11; H, 5.63; N, 6.31. ESI‐MS analysis for C_28_H_26_N_2_O_4_ [2 M+Na^+^]: Calc.: 931.3677 *m*/*z*, exp.: 931.3676 *m*/*z*. Purity: 98.87%.

##### 4.1.4.13 *(E)‐N*‐(p‐Tolyl)‐2‐(3,4,5‐Trimethoxystyryl)Quinoline‐4‐Carboxamide (4m)

Purified by crystallization from ethanol. Yellow solid; yield: 73%; m.p.: 238°C–239°C. ¹H NMR (300 MHz, DMSO‐d₆) *δ*: 2.32 (s, 3H, 4′‐CH₃_phenyl_), 3.71 (s, 3H, 4″‐OCH₃_styryl_), 3.87 (s, 6H, 3″/5″‐OCH₃_styryl_), 7.12 (s, 2H, 2″/6″‐CH_styryl_), 7.22 (d, 2H, *J* = *8.1 Hz*, 2′/6′‐CH_phenyl_), 7.54–7.72 (m, 4H, 2′/6′‐CH_phenyl_, CH**═**CH‐Ph, 6‐CH_quin_), 7.82 (t, 1H, *J* = *7.7 Hz*, 7‐CH_quin_), 7.91 (d, 1H, *J* = *16.2 Hz*, CH**═**CH‐Ph), 8.05–8.11 (m, 3H, 3/5/8‐CH_quin_), 10.74 (s, 1H, NH–C**═**O); ¹³C APT NMR (75 MHz, DMSO‐d₆) *δ*: 21.00 (4′‐CH₃_phenyl_), 56.42 (3″/5″‐OCH₃_styryl_), 60.57 (4″‐OCH₃_styryl_), 105.30 (2″/6″‐CH_styryl_), 117.88 (CH‐5_quin_), 120.41 (2′/6′‐CH_phenyl_), 123.60 (C‐4a_quin_), 125.57 (CH‐3_quin_), 127.41 (CH‐6_quin_), 128.11 (CH‐7_quin_), 129.52 (CH‐8_quin_), 129.67 (CH‐3′/5′_phenyl_), 130.66 (CH**═**CH‐Ph), 132.24 (1″‐C_styryl_), 133.62 (C‐4′_phenyl_), 135.68 (CH**═**CH‐Ph), 136.79 (1′‐C_phenyl_), 138.83 (4″‐C_styryl_), 143.02 (C‐4_quin_), 148.53 (C‐8a_quin_), 153.63 (3″/5″‐C_styryl_), 156.11 (C‐2_quin_), 165.63 (NH–C**═**O). Anal calcd for C_28_H_26_N_2_O_4_: C, 73.99; H, 5.77; N, 6.16. Found: C, 74.09; H, 5.85; N, 6.08. ESI‐MS analysis for [C_28_H_26_N_2_O_4_ + H^+^]: Calc.: 455.1965 *m*/*z*, exp.: 455.1965 *m*/*z*. Purity: 98.00%.

##### 4.1.4.14 *(E)‐N*‐(2,6‐Dimethylphenyl)‐2‐(3,4,5‐Trimethoxystyryl)Quinoline‐4‐Carboxamide (4n)

Purified by flash column chromatography (ethyl acetate/cyclohexane, 1:1). Yellow solid; yield: 71%; m.p.: 231°C–232°C. ¹H NMR (300 MHz, DMSO‐d₆) *δ*: 2.37 (s, 6H, 2′/6′‐CH₃_phenyl_), 3.72 (s, 3H, 4″‐OCH₃_styryl_), 3.89 (s, 6H, 3″/5″‐OCH₃_styryl_), 7.04–7.26 (m, 5H, 2″/6″‐CH_styryl_, 3′/4′/5′‐CH_phenyl_), 7.59–7.69 (m, 2H, CH**═**CH‐Ph, 6‐CH_quin_), 7.81–7.94 (m, 2H, CH**═**CH‐Ph, CH‐7_quin_), 8.01 (s, 1H, 3‐CH_quin_), 8.09 (d, 1H, *J* **=** *8.1 Hz*, 8‐CH_quin_), 8.20 (d, 1H, *J* **=** *8.1 Hz*, 5‐CH_quin_), 10.22 (s, 1H, NH–C**═**O); ¹³C APT NMR (75 MHz, DMSO‐d₆) *δ*: 18.85 (2′/6′‐CH₃_phenyl_), 56.49 (3″/5″‐OCH₃_styryl_), 60.58 (4″‐OCH₃_styryl_), 105.40 (2″/6″‐CH_styryl_), 118.38 (CH‐5_quin_), 123.85 (C‐4a_quin_), 125.55 (CH‐3_quin_), 127.45 (4′‐CH_phenyl_, CH‐6_quin_), 127.96 (CH‐7_quin_), 128.39 (CH‐3′/5′_phenyl_), 129.60 (CH‐8_quin_), 130.73 (CH**═**CH‐Ph), 132.23 (1″‐C_styryl_), 134.92 (1′‐C_phenyl_), 135.41 (CH**═**CH‐Ph), 135.84 (2′/6′‐CH_phenyl_), 138.86 (4″‐C_styryl_), 143.27 (C‐4_quin_), 148.56 (C‐8a_quin_), 153.64 (3″/5″‐C_styryl_), 155.83 (C‐2_quin_), 165.88 (NH–C**═**O). Anal calcd for C_29_H_28_N_2_O_4_: C, 74.34; H, 6.02; N, 5.98. Found: C, 74.48; H, 6.15; N, 5.89. ESI‐MS analysis for [C_29_H_28_N_2_O_4_ **+** H^+^]: Calc.: 469.2122 *m*/*z*, exp.: 469.2123 *m*/*z*. Purity: 99.75%.

##### 4.1.4.15 *(E)‐N*‐(3,4,5‐Trimethoxyphenyl)‐2‐(3,4,5‐Trimethoxystyryl)Quinoline‐4‐Carboxamide (4o)

Purified by crystallization from ethanol. Yellow solid; yield: 67%; m.p.: 213°C–214°C. ¹H NMR (300 MHz, DMSO‐d₆) *δ*: 3.68 (s, 3H, 4′‐OCH₃_phenyl_), 3.71 (s, 3H, 4″‐OCH₃_styryl_), 3.80 (s, 6H, 3′/5′‐OCH₃_phenyl_), 3.88 (s, 6H, 3″/5″‐OCH₃_styryl_), 7.12 (s, 2H, 2″/6″‐CH_styryl_), 7.24 (s, 2H, 2′/6′‐CH_phenyl_), 7.54–7.66 (m, 2H, CH**═**CH‐Ph, 6‐CH_quin_), 7.80–7.93 (m, 2H, CH**═**CH‐Ph, CH‐7_quin_), 8.06–8.14 (m, 3H, 3/5/8‐CH_quin_), 10.74 (s, 1H, NH–C**═**O); ¹³C APT NMR (75 MHz, DMSO‐d₆) *δ*: 56.29 (3″/5″‐OCH₃_phenyl_), 56.44 (3″/5″‐OCH₃_styryl_), 60.57 (4″‐OCH₃_styryl_), 60.64 (4′‐OCH₃_phenyl_), 98.25 (CH‐2′/6′_phenyl_), 105.32 (2″/6″‐CH_styryl_), 117.92 (CH‐5_quin_), 123.52 (C‐4a_quin_), 125.58 (CH‐3_quin_), 127.45 (CH‐6_quin_), 128.08 (CH‐7_quin_), 129.54 (CH‐8_quin_), 130.70 (CH**═**CH‐Ph), 132.22 (1″‐C_styryl_), 135.42 (1′‐C_phenyl_), 135.70 (CH**═**CH‐Ph), 138.45 (4′‐CH_phenyl_), 141.72 (4″‐C_styryl_), 142.84 (C‐4_quin_), 148.53 (C‐8a_quin_), 153.27 (3″/5″‐C_styryl_), 153.63 (3″/5″‐C_styryl_), 156.07 (C‐2_quin_), 165.65 (NH–C**═**O). Anal calcd for C_30_H_30_N_2_O_7_: C, 67.91; H, 5.70; N, 5.28. Found: C, 68.01; H, 5.79; N, 5.17. ESI‐MS analysis for [C_30_H_30_N_2_O_7_ **+** H^+^]: Calc.: 531.2126 *m/z*, exp.: 531.2126 *m/z*. Purity: 98.18%.

##### 4.1.4.16 *(E)‐N*‐(3‐Chlorophenyl)‐2‐(3,4,5‐Trimethoxystyryl)Quinoline‐4‐Carboxamide (4p)

Purified by flash column chromatography (ethyl acetate/cyclohexane, 1:1). Yellow solid; yield: 69%; m.p.: 256°C–257°C. ¹H NMR (300 MHz, DMSO‐d₆) *δ*: 3.72 (s, 3H, 4″‐OCH₃_styryl_), 3.88 (s, 6H, 3″/5″‐OCH₃_styryl_), 7.12 (s, 2H, 2″/6″‐CH_styryl_), 7.25 (b d, 1H, *J* **=** *8.1 Hz*, 6′‐CH_phenyl_), 7.46 (t, 1H, *J* **=** *7.8 Hz*, 4′‐CH_pheny_), 7.55–7.73 (m, 3H, 2′/5′‐CH_phenyl_, CH**═**CH‐Ph), 7.81–7.95 (m, 2H, CH**═**CH‐Ph, 6‐CH_quin_), 8.05–8.12 (m, 4H, 3/5/7/8‐CH_quin_), 11.03 (s, 1H, NH–C**═**O); ¹³C APT NMR (75 MHz, DMSO‐d₆) *δ*: 56.43 (3″/5″‐OCH₃_styryl_), 60.57 (4″‐OCH₃_styryl_), 105.31 (2″/6″‐CH_styryl_), 118.01 (CH‐6′_phenyl_), 118.84 (CH‐2′_phenyl_), 119.92 (CH‐5_quin_), 123.41 (C‐4a_quin_), 124.38 (CH‐3_quin_), 125.51 (4′‐CH_phenyl_), 127.56 (CH‐6_quin_), 128.06 (CH‐7_quin_), 129.57 (CH‐8_quin_), 130.77 (CH**═**CH‐Ph), 131.05 (5′‐CH_phenyl_), 132.21 (1″‐C_styryl_), 133.66 (3′‐CH_phenyl_), 135.82 (CH**═**CH‐Ph), 138.88 (1′‐C_phenyl_), 140.68 (4″‐C_styryl_), 142.45 (C‐4_quin_), 148.55 (C‐8a_quin_), 153.64 (3″/5″‐C_styryl_), 156.12 (C‐2_quin_), 166.13 (NH–C**═**O). Anal calcd for C_27_H_23_ClN_2_O_4_: C, 68.28; H, 4.88; N, 5.90. Found: C, 68.37; H, 4.97; N, 5.81. ESI‐MS analysis for C_27_H_23_ClN_2_O_4_ [M** +** H^+^]: Calc.: 475.1419 *m*/*z*, exp.: 475.1419 *m*/*z*. Purity: 99.25%.

##### 4.1.4.17 *(E)‐N*‐(4‐Chlorophenyl)‐2‐(3,4,5‐Trimethoxystyryl)Quinoline‐4‐Carboxamide (4q)

Purified by flash column chromatography (ethyl acetate/cyclohexane, 1:1). Yellow solid; yield: 44%; m.p.: 247°C–248°C. ¹H NMR (300 MHz, DMSO‐d₆) *δ*: 3.72 (s, 3H, 4″‐OCH₃_styryl_), 3.88 (s, 6H, 3″/5″‐OCH₃_styryl_), 7.12 (s, 2H, 2″/6″‐CH_styryl_), 7.47–7.65 (m, 4H, 3′/5′‐CH_phenyl_, 6‐CH_quin_, CH**═**CH‐Ph), 7.80–7.94 (m, 4H, 2′/6′‐CH_phenyl_, CH**═**CH‐Ph, 7‐CH_quin_), 8.06–8.12 (m, 3H, 3/5/8‐CH_quin_), 10.97 (s, 1H, NH–C**═**O); ¹³C APT NMR (75 MHz, DMSO‐d₆) *δ*: 56.43 (3″/5″‐OCH₃_styryl_), 60.57 (4″‐OCH₃_styryl_), 105.33 (2″/6″‐CH_styryl_), 117.98 (CH‐5_quin_), 121.99 (2′/6′‐CH_phenyl_), 123.46 (C‐4a_quin_), 125.50 (CH‐3_quin_), 127.51 (CH‐6_quin_), 128.07 (CH‐7_quin_), 128.28 (4′‐C_phenyl_), 129.25 (3′/5′‐CH_phenyl_), 129.56 (CH‐8_quin_), 130.73 (CH**═**CH‐Ph), 132.22 (1″‐C_styryl_), 135.79 (CH**═**CH‐Ph), 138.22 (1′‐C_phenyl_), 138.88 (4″‐C_styryl_), 142.61 (C‐4_quin_), 148.54 (C‐8a_quin_), 153.64 (3″/5″‐C_styryl_), 156.12 (C‐2_quin_), 165.94 (NH–C**═**O). Anal calcd for C_27_H_23_ClN_2_O_4_: C, 68.28; H, 4.88; N, 5.90. Found: C, 68.39; H, 5.00; N, 5.81. ESI‐MS analysis for C_27_H_23_ClN_2_O_4_ [2 M+Na^+^]: Calc.: 971.2585 *m*/*z*, exp.: 971.2597 *m*/*z*. Purity: 99.46%.

##### 4.1.4.18 *(E)‐N*‐(4‐(Trifluoromethyl)phenyl)‐2‐(3,4,5‐Trimethoxystyryl)Quinoline‐4‐Carboxamide (4r)

Purified by crystallization from ethanol. Yellow solid; yield: 72%; m.p.: 268°C–269°C. ¹H NMR (300 MHz, DMSO‐d₆) *δ*: 3.72 (s, 3H, 4″‐OCH₃_styryl_), 3.88 (s, 6H, 3″/5″‐OCH₃_styryl_), 7.12 (s, 2H, 2″/6″‐CH_styryl_), 7.55–7.66 (m, 2H, 6‐CH_quin_, CH**═**CH‐Ph), 7.79–7.95 (m, 4H, 2′/6′‐CH_phenyl_, CH**═**CH‐Ph, 7‐CH_quin_), 8.04–8.14 (m, 5H, 3′/5′‐CH_phenyl_, 3/5/8‐CH_quin_), 11.20 (s, 1H, NH–C**═**O); ¹³C APT NMR (75 MHz, DMSO‐d₆) *δ*: 56.43 (3″/5″‐OCH₃_styryl_), 60.57 (4″‐OCH₃_styryl_), 105.32 (2″/6″‐CH_styryl_), 118.05 (CH‐5_quin_), 120.12 (q, *J* **=** *263.9 Hz*, CF_3_), 120.39 (2′/6′‐CH_phenyl_), 123.36 (C‐4a_quin_), 124.65 (q, *J* **=** *31.7 Hz*, 4′‐C_phenyl_‐CF_3_), 125.44 (CH‐3_quin_), 126.65 (b q, CH‐3′/5′_phenyl_), 127.59 (CH‐6_quin_), 128.04 (CH‐7_quin_), 129.59 (CH‐8_quin_), 130.79 (CH**═**CH‐Ph), 132.20 (1″‐C_styryl_), 135.85 (CH**═**CH‐Ph), 138.88 (1′‐C_phenyl_), 142.37 (4″‐C_styryl_), 142.82 (C‐4_quin_), 148.54 (C‐8a_quin_), 153.64 (3″/5″‐C_styryl_), 156.13 (C‐2_quin_), 166.36 (NH–C**═**O). Anal calcd for C_28_H_23_F_3_N_2_O_4_: C, 66.14; H, 4.56; N, 5.51. Found: C, 66.27; H, 4.67; N, 5.42. ESI‐MS analysis for [C_28_H_23_F_3_N_2_O_4_ **+** H^+^]: Calc.: 509.1683 *m*/*z*, exp.: 509.1684 *m*/*z*. Purity: 99.26%.

##### 4.1.4.19 *(E)‐N*‐(4‐Nitrophenyl)‐2‐(3,4,5‐Trimethoxystyryl)Quinoline‐4‐Carboxamide (4s)

Purified by crystallization from ethanol. Yellow solid; yield: 70%; m.p.: 288°C–289°C. ¹H NMR (300 MHz, DMSO‐d₆) *δ*: 3.71 (s, 3H, 4″‐OCH₃_styryl_), 3.87 (s, 6H, 3″/5″‐OCH₃_styryl_), 7.11 (s, 2H, 2″/6″‐CH_styryl_), 7.55–7.67 (m, 2H, 6‐CH_quin_, CH**═**CH‐Ph), 7.82–7.94 (m, 2H, CH**═**CH‐Ph, 7‐CH_quin_), 8.03–8.16 (m, 5H, 2′/3′/5′/6′‐CH_phenyl_, 3‐CH_quin_), 8.29–8.36 (m, 2H, 5/8‐CH_quin_), 11.42 (s, 1H, NH–C**═**O); ¹³C APT NMR (75 MHz, DMSO‐d₆) δ: 56.44 (3″/5″‐OCH₃_styryl_), 60.57 (4″‐OCH₃_styryl_), 105.34 (2″/6″‐CH_styryl_), 118.14 (CH‐5_quin_), 120.27 (2′/6′‐CH_phenyl_), 123.25 (C‐4a_quin_), 125.39 (CH‐3_quin_), 125.48 (3′/5′‐CH_phenyl_), 127.69 (CH‐6_quin_), 128.01 (CH‐7_quin_), 129.61 (CH‐8_quin_), 130.85 (CH**═**CH‐Ph), 132.17 (1″‐C_styryl_), 135.93 (CH**═**CH‐Ph), 138.91 (4″‐C_styryl_), 142.02 (C‐4_quin_), 143.43 (1′‐C_phenyl_), 145.32 (4′‐C_phenyl_), 148.54 (C‐8a_quin_), 153.64 (3″/5″‐C_styryl_), 156.13 (C‐2_quin_), 166.56 (NH–C**═**O). Anal calcd for C_27_H_23_N_3_O_6_: C, 66.80; H, 4.78; N, 8.66. Found: C, 66.99; H, 4.87; N, 8.58. ESI‐MS analysis for [C_29_H_28_N_2_O_4_ **+** H^+^]: Calc.: 469.2122 *m*/*z*, exp.: 469.2123 *m*/*z*. Purity: 98.48%.

##### 4.1.4.20 *(E)‐N*‐(4‐Nitro‐3‐(Trifluoromethyl)phenyl)‐2‐(3,4,5‐Trimethoxystyryl)Quinoline‐4‐Carboxamide (4t)

Purified by flash column chromatography (ethyl acetate/cyclohexane, 1:1). Yellow solid; yield: 75%; m.p.: 276°C–277°C. ¹H NMR (300 MHz, DMSO‐d₆) *δ*: 3.71 (s, 3H, 4″‐OCH₃_styryl_), 3.88 (s, 6H, 3″/5″‐OCH₃_styryl_), 7.11 (s, 2H, 2″/6″‐CH_styryl_), 7.55–7.68 (m, 2H, 6‐CH_quin_, CH**═**CH‐Ph), 7.82–7.93 (m, 2H, CH**═**CH‐Ph, 7‐CH_quin_), 8.08–8.19 (m, 3H, 6′‐CH_phenyl_, 3/8‐CH_quin_), 8.32 (b s, 2H, 2′/5′‐CH_phenyl_), 8.50 (b s, 1H, 5‐CH_quin_), 11.60 (s, 1H, NH–C**═**O); ¹³C APT NMR (75 MHz, DMSO‐d₆) *δ*: 56.44 (3″/5″‐OCH₃_styryl_), 60.49 (4″‐OCH₃_styryl_), 105.33 (2″/6″‐CH_styryl_), 118.28 (CH‐5_quin_), 118.68 (q, *J* **=** *4.5 Hz*, 2′‐CH_phenyl_), 122.51 (q, *J* **=** *271.1 Hz*, CF_3_), 123.14 (C‐4a_quin_), 123.83 (q, *J* **=** *32.9 Hz*, 3′‐C_phenyl_‐CF_3_), 123.81 (6′‐CH_phenyl_), 125.45 (CH‐3_quin_), 127.75 (5′‐CH_phenyl_), 127.97 (CH‐6_quin_), 128.19 (CH‐7_quin_), 129.61 (CH‐8_quin_), 130.92 (CH**═**CH‐Ph), 132.13 (1″‐C_styryl_), 135.97 (CH**═**CH‐Ph), 138.93 (1′‐C_phenyl_), 141.53 (4″‐C_styryl_), 142.58 (4′‐C_phenyl_), 143.79 (C‐4_quin_), 148.56 (C‐8a_quin_), 153.65 (3″/5″‐C_styryl_), 156.09 (C‐2_quin_), 166.77 (NH–C**═**O). Anal calcd for C_28_H_22_F_3_N_3_O_6_: C, 66.76; H, 4.01; N, 7.59. Found: C, 66.87; H, 4.12; N, 7.44. ESI‐MS analysis for [C_28_H_22_F_3_N_3_O_6_ **+** H^+^]: Calc.: 554.1533 *m*/*z*, exp.: 554.1532 *m*/*z*. Purity: 98.55%.

##### 4.1.4.21 *(E)‐*2‐(3‐Methylstyryl)‐*N*‐(4‐Nitro‐3‐(Trifluoromethyl)Phenyl)Quinoline‐4‐Carboxamide (4u)

Purified by flash column chromatography (ethyl acetate/cyclohexane, 1:1). White solid; yield: 67%; m.p.: 243°C–245°C. ¹H NMR (300 MHz, DMSO‐d₆) *δ*: 2.36 (s, 3H, 3″‐CH₃_styryl_), 7.19–7.37 (m, 2H, 4″/5″‐CH_styryl_), 7.54–7.67 (m, 4H, 2″/6″‐CH_styryl_, 6‐CH_quin_, CH**═**CH‐Ph), 7.82–7.95 (m, 2H, CH**═**CH‐Ph, 7‐CH_quin_), 8.08–8.48 (m, 6H, 2′/5′/6′‐CH_phenyl_, 3/5/8‐CH_quin_), 11.59 (s, 1H, NH–C**═**O); ¹³C APT NMR (75 MHz, DMSO‐d₆) *δ*: 21.35 (3″‐CH₃_styryl_), 118.34 (CH‐5_quin_), 118.62 (b q, 2′‐CH_phenyl_), 122.51 (q, *J* **=** *271.1 Hz*, CF_3_), 122.95 (q, *J* **=** *32.5 Hz*, 3′‐C_phenyl_‐CF_3_), 123.17 (C‐4a_quin_), 123.77 (6′‐CH_phenyl_), 125.13 (CH‐3_quin_), 125.45 (5′‐CH_phenyl_), 127.83 (CH‐6_quin_), 128.27 (6″‐CH_styryl_), 128.30 (CH**═**CH‐Ph), 128.40 (5″‐CH_styryl_), 129.32 (CH‐7_quin_), 129.71 (CH‐8_quin_), 130.20 (2″‐CH_styryl_), 130.92 (CH‐6_quin_), 135.83 (CH**═**CH‐Ph), 136.38 (1″‐C_styryl_), 138.55 (3″‐C_styryl_), 141.65 (C‐4_quin_), 142.48 (1′‐C_phenyl_), 143.91 (4′‐C_phenyl_), 148.47 (C‐8a_quin_), 155.97 (C‐2_quin_), 166.79 (NH–C**═**O). Anal calcd for C_26_H_18_F_3_N_3_O_3_: C, 65.41; H, 3.80; N, 8.80. Found: C, 65.53; H, 3.97; N, 8.69. ESI‐MS analysis for [C_26_H_18_F_3_N_3_O_3_ **+** H^+^]: Calc.: 478.1373 *m*/*z*, exp.: 478.1373 *m*/*z*. Purity: 99.37%.

##### 4.1.4.22 *(E)‐*2‐(4‐Methoxystyryl)‐*N*‐(4‐Nitro‐3‐(Trifluoromethyl)phenyl)Quinoline‐4‐Carboxamide (4v)

Purified by flash column chromatography (ethyl acetate/cyclohexane, 1:1). Yellow solid; yield: 57%; m.p.: 264°C–266°C. ¹H NMR (300 MHz, DMSO‐d₆) *δ*: 3.80 (s, 3H, 3″‐OCH₃_styryl_), 7.01 (d, 2H, *J* **=** *8.1 Hz*, 3″/5″‐CH_styryl_), 7.41 (d, 1H, *J* **=** *16.2 Hz*, CH** =** CH‐Ph), 7.59–7‐93 (m, 5H, 2″/6″‐CH_styryl_, 6‐CH_quin_, 6′‐CH_phenyl_, CH**═**CH‐Ph), 8.05–8.48 (m, 6H, 2′/5′‐CH_phenyl_, 3/5/7/8‐CH_quin_), 11.61 (s, 1H, NH–C**═**O); ¹³C APT NMR (75 MHz, DMSO‐d₆) *δ*: 55.71 (3″‐OCH₃_styryl_), 114.90 (3″/5″‐CH_styryl_), 118.17 (CH‐5_quin_), 118.66 (b q, 2′‐CH_phenyl_), 122.49 (q, *J* **=** *271.5 Hz*, CF_3_), 123.15 (C‐4a_quin_), 123.39 (q, *J* **=** *35.0 Hz*, 3′‐C_phenyl_‐CF_3_), 123.77 (6′‐CH_phenyl_), 125.41 (5′‐CH_phenyl_), 126.21 (CH‐3_quin_), 127.58 (CH‐6_quin_), 128.27 (CH**═**CH‐Ph), 129.08 (1″‐C_styryl_), 129.38 (2″/6″‐CH_styryl_), 129.57 (CH‐7_quin_), 130.86 (CH‐8_quin_), 135.51 (CH**═**CH‐Ph), 141.51 (C‐4_quin_), 143.87 (1′‐C_phenyl_), 148.49 (4′‐C_phenyl_), 156.29 (C‐8a_quin_), 160.51 (C‐2_quin_), 160.67 (4″‐C_styryl_), 166.83 (NH–C**═**O). Anal calcd for C_26_H_18_F_3_N_3_O_4_: C, 63.29; H, 3.68; N, 8.52. Found: C, 63.40; H, 3.77; N, 8.43. ESI‐MS analysis for [C_26_H_18_F_3_N_3_O_4_ **+** H^+^]: Calc.: 494.1322 *m*/*z*, exp.: 494.1329 *m*/*z*. Purity: 98.35%.

##### 4.1.4.23 *(E)‐*2‐(4‐Chlorostyryl)‐*N*‐(4‐Nitro‐3‐(Trifluoromethyl)Phenyl)Quinoline‐4‐Carboxamide (4w)

Purified by flash column chromatography (ethyl acetate/cyclohexane, 1:1). White solid; yield: 75%; m.p.: 250°C–252°C. ¹H NMR (600 MHz, DMSO‐d₆) *δ*: 7.49 (d t, 2H, *J* **=** *8.4/6.6 Hz*, 2″/6″‐CH_styryl_), 7.58 (d, 1H, *J* **=** *16.2 Hz*, CH**═**CH‐Ph), 7.63–7.66 (m, 1H, 6‐CH_quin_), 7.78 (d t, 2H, *J* **=** *8.4/6.6 Hz*, 3″/5″‐CH_styryl_), 7.82–7.85 (m, 1H, 7‐CH_quin_), 7.94 (d, 1H, *J* **=** *16.2 Hz*, CH**═**CH‐Ph), 8.08–8.10 (m, 1H, 6′‐CH_phenyl_), 8.13–8.14 (m, 1H, 5′‐CH_phenyl_), 8.19 (s, 1H, 3‐CH_quin_), 8.28–8.31 (m, 2H, 5/8‐CH_quin_), 8.47 (b s, 1H, 2′‐CH_phenyl_), 11.60 (s, 1H, NH–C**═**O); ¹³C decoupling NMR (100 MHz, DMSO‐d₆) *δ*: 118.43 (CH‐5_quin_), 118.71 (b q, 2′‐CH_phenyl_), 122.50 (q, *J* **=** *266.8 Hz*, CF_3_), 123.26 (C‐4a_quin_), 123.42 (q, *J* **=** *30.8 Hz*, 3′‐C_phenyl_‐CF_3_), 123.83 (6′‐CH_phenyl_), 125.46 (CH‐3_quin_), 127.98 (CH‐6_quin_), 128.20 (b q, 5′‐CH_phenyl_), 129.44 (2″/6″‐CH_styryl_, CH**═**CH‐Ph), 129.51 (3″/5″‐CH_styryl_), 129.76 (CH‐7_quin_), 130.98 (CH‐8_quin_), 133.87 (1″‐C_styryl_), 134.37 (CH**═**CH‐Ph), 135.47 (4″‐C_styryl_), 141.74 (C‐4_quin_), 142.61 (4′‐C_phenyl_), 143.79 (1′‐C_phenyl_), 148.50 (C‐8a_quin_), 155.73 (C‐2_quin_), 166.74 (NH–C**═**O). Anal calcd for C_25_H_15_ClF_3_N_3_O_3_: C, 60.31; H, 3.04; N, 8.44. Found: C, 60.43; H, 3.17; N, 8.32. ESI‐MS analysis for [C_25_H_15_ClF_3_N_3_O_3_ **+** H^+^]: Calc.: 498.0827 *m*/*z*, exp.: 498.0829 *m*/*z*. Purity: 98.02%.

##### 4.1.4.24 *(E)‐*2‐(3,4‐Dichlorostyryl)‐*N*‐(4‐Nitro‐3‐(Trifluoromethyl)Phenyl)Quinoline‐4‐Carboxamide (4x)

Purified by flash column chromatography (ethyl acetate/cyclohexane, 1:1). White solid; yield: 74%; m.p.: 295°C–297°C. ¹H NMR (300 MHz, DMSO‐d₆) *δ*: 7.65–7.79 (m, 4H, 2″/5″/6″‐CH_styryl_, CH**═**CH‐Ph), 7.83–7.96 (m, 2H, 6′‐CH_phenyl_, 6‐CH_quin_), 8.07–8.21 (m, 4H, 3/7/8‐CH_quin_, CH**═**CH‐Ph), 8.28–8.48 (m, 3H, 2′/5′‐CH_phenyl_, 5‐CH_quin_), 11.64 (s, 1H, NH–C**═**O); ¹³C APT NMR (75 MHz, DMSO‐d₆) *δ*: 118.57 (CH‐5_quin_), 118.68 (b q, 2′‐CH_phenyl_), 122.49 (q, *J* **=** *271.4 Hz*, CF_3_), 123.29 (C‐4a_quin_), 123.39 (q, *J* **=** *32.9 Hz*, 3′‐C_phenyl_‐CF_3_), 123.76 (6′‐CH_phenyl_), 125.48 (CH‐3_quin_), 127.86 (5′‐CH_phenyl_), 128.17 (CH‐6_quin_), 128.28 (6″‐CH_styryl_), 129.46 (CH**═**CH‐Ph), 129.78 (CH‐7_quin_), 130.73 (CH‐8_quin_), 131.09 (2″‐CH_styryl_), 131.50 (5″‐CH_styryl_), 132.21 (1″‐C_styryl_), 133.08 (CH**═**CH‐Ph), 137.40 (3″/4″‐C_styryl_), 141.77 (C‐4_quin_), 142.56 (4′‐C_phenyl_), 143.75 (1′‐C_phenyl_), 148.44 (C‐8a_quin_), 155.42 (C‐2_quin_), 166.67 (NH–C**═**O). Anal calcd for C_25_H_14_Cl_2_F_3_N_3_O_3_: C, 56.41; H, 2.65; N, 7.89. Found: C, 56.55; H, 2.76; N, 7.78. ESI‐MS analysis for [C_25_H_14_Cl_2_F_3_N_3_O_3_ **+** H^+^]: Calc.: 532.0437 *m*/*z*, exp.: 532.0436 *m*/*z*. Purity: 98.85%.

##### 4.1.4.25 *(E)‐*2‐(3‐Methylstyryl)‐*N*‐(4‐(Trifluoromethyl)Phenyl)Quinoline‐4‐Carboxamide (4y)

Purified by crystallization from ethanol. White solid; yield: 53%; m.p.: 260°C–262°C. ¹H NMR (600 MHz, DMSO‐d₆) *δ*: 2.35 (s, 3H, 3″‐CH₃_styryl_), 7.18 (d, 1H, *J* **=** *7.8 Hz*, 4″‐CH_styryl_), 7.32 (t, 1H, *J* **=** *7.8 Hz*, 5″‐CH_styryl_), 7.52–7.54 (m, 2H, 6″‐CH_styryl_, CH**═**CH‐Ph), 7.58 (s, 1H, 2″‐CH_styryl_), 7.62 (t, 1H, *J* **=** *7.8 Hz*, 6‐CH_quin_), 7.78–7.83 (m, 3H, 2′/6′‐CH_phenyl_, 7‐CH_quin_), 7.92 (d, 1H, *J* **=** *16.2 Hz*, CH**═**CH‐Ph), 8.03 (d, 2H, *J* **=** *8.4 Hz*, 3′/5′‐CH_phenyl_), 8.08 (d, 2H, *J* **=** *9.0 Hz*, 5/8‐CH_quin_), 8.14 (s, 1H, 3‐CH_quin_), 11.18 (s, 1H, NH–C**═**O); ¹³C decoupling NMR (151 MHz, DMSO‐d₆) *δ*: 21.60 (3″‐CH₃_styryl_), 118.11 (CH‐5_quin_), 120.32 (2′/6′‐CH_phenyl_), 123.38 (C‐4a_quin_), 124.60 (q, *J* **=** *31.6 Hz*, 3′‐C_phenyl_‐CF_3_), 124.77 (q, *J* **=** *272.4 Hz*, CF_3_), 125.09 (CH‐3_quin_), 125.39 (6″‐CH_styryl_), 126.66 (b q, 3′/5′‐CH_phenyl_), 127.67 (CH**═**CH‐Ph), 128.28 (5″‐CH_styryl_), 128.46 (CH‐7_quin_), 129.28 (4″‐CH_styryl_), 129.67 (2″‐CH_styryl_), 130.12 (CH‐6_quin_), 130.77 (CH‐8_quin_), 135.69 (CH**═**CH‐Ph), 136.45 (1″‐C_styryl_), 138.51 (3″‐C_styryl_), 142.43 (C‐4_quin_), 142.79 (1′‐C_phenyl_), 148.44 (C‐8a_quin_), 156.00 (C‐2_quin_), 166.33 (NH–C**═**O). Anal calcd for C_26_H_19_F_3_N_2_O: C, 72.21; H, 4.43; N, 6.48. Found: C, 72.33; H, 4.55; N, 6.41. ESI‐MS analysis for [C_26_H_19_F_3_N_2_O** +** H^+^]: Calc.: 433.1522 *m*/*z*, exp.: 433.1522 *m*/*z*. Purity: 99.28%.

##### 4.1.4.26 *(E)‐*2‐(4‐Chlorostyryl)‐*N*‐(4‐(Trifluoromethyl)Phenyl)Quinoline‐4‐Carboxamide (4z)

Purified by flash column chromatography (ethyl acetate/cyclohexane, 1:1). White solid; yield: 58%; m.p.: 271°C–273°C. ¹H NMR (600 MHz, DMSO‐d₆) *δ*: 7.49 (d, 2H, *J* **=** *8.4 Hz*, 2″/6″‐CH_styryl_,), 7.57 (d, 1H, *J* **=** *16.2 Hz*, CH**═**CH‐Ph), 7.63 (t, 1H, *J* **=** *7.8 Hz*, 6‐CH_quin_), 7.77–7.83 (m, 5H, 3″/5″‐CH_styryl_, 2′/6′‐CH_pheny_, 7‐CH_quin_), 7.95 (d, 1H, *J* **=** *16.2 Hz*, CH**═**CH‐Ph), 8.02 (d, 2H, *J* **=** *8.4 Hz*, 3′/5′‐CH_phenyl_), 8.08 (d, 2H, *J* **=** *9.0 Hz*, 5/8‐CH_quin_), 8.14 (s, 1H, 3‐CH_quin_), 11.19 (s, 1H, NH–C**═**O); ¹³C decoupling NMR (151 MHz, DMSO‐d₆) *δ*: 118.16 (CH‐5_quin_), 120.32 (2′/6′‐CH_phenyl_), 123.44 (C‐4a_quin_), 124.64 (q, *J* **=** *31.9 Hz*, 4′‐C_phenyl_‐CF_3_), 124.77 (q, *J* **=** *271.8 Hz*, CF_3_), 125.41 (CH‐3_quin_), 126.66 (b q, 3′/5′‐CH_phenyl_), 127.82 (CH‐6_quin_), 129.40 (2″/6″‐CH_styryl_), 129.44 (CH**═**CH‐Ph), 129.47 (3″/5″‐CH_styryl_), 129.70 (CH‐7_quin_), 130.84 (CH‐8_quin_), 133.78 (1″‐C_styryl_), 134.22 (CH**═**CH‐Ph), 135.49 (4″‐C_styryl_), 142.53 (1′‐C_phenyl_), 142.77 (C‐4_quin_), 148.42 (C‐8a_quin_), 155.74 (C‐2_quin_), 166.29 (NH–C**═**O). Anal calcd for C_25_H_16_ClF_3_N_2_O: C, 66.31; H, 3.56; N, 6.19. Found: C, 66.43; H, 3.67; N, 6.11. ESI‐MS analysis for [C_25_H_16_ClF_3_N_2_O** +** H^+^]: Calc.: 453.0976 *m*/*z*, exp.: 453.0975 *m*/*z*. Purity: 98.78%.

##### 4.1.4.27 *(E)‐*2‐(4‐Bromostyryl)‐*N*‐(4‐(Trifluoromethyl)Phenyl)Quinoline‐4‐Carboxamide (4aa)

Purified by flash column chromatography (ethyl acetate/cyclohexane, 1:1). Yellow solid; yield: 61%; m.p.: 267°C–269°C. ¹H NMR (600 MHz, DMSO‐d₆) *δ*: 7.57–7.63 (m, 4H, 2″/6″‐CH_styryl_, CH**═**CH‐Ph, 6‐CH_quin_), 7.71 (d, 2H, 3″/5″‐CH_styryl_), 7.78–7.83 (m, 3H, 2′/6′‐CH_phenyl_, 7‐CH_quin_), 7.93 (d, 1H, *J* **=** *16.8 Hz*, CH**═**CH‐Ph), 8.02 (d, 2H, *J* **=** *8.4 Hz*, 3′/5′‐CH_phenyl_), 8.08 (d, 2H, *J* **=** *8.4 Hz*, 5/8‐CH_quin_), 8.14 (s, 1H, 3‐CH_quin_), 11.18 (s, 1H, NH–C**═**O); ¹³C decoupling NMR (151 MHz, DMSO‐d₆) *δ*: 118.17 (CH‐5_quin_), 120.32 (2′/6′‐CH_phenyl_), 120.94 (q, *J* **=** *260.6 Hz*, CF_3_), 121.41 (q, *J* **=** *31.9 Hz*, 4′‐C_phenyl_‐CF_3_), 122.49 (C‐4a_quin_), 123.44 (CH‐3_quin_), 125.41 (CH‐6_quin_), 126.66 (b q, 3′/5′‐CH_phenyl_), 127.83 (CH**═**CH‐Ph), 129.49 (CH‐7_quin_), 129.74 (2″/6″‐CH_styryl_, CH‐8_quin_), 130.85 (4″‐C_styryl_), 132.32 (3″/5″‐CH_styryl_), 134.30 (CH**═**CH‐Ph), 135.83 (1″‐C_styryl_), 142.53 (1′‐C_phenyl_), 142.76 (C‐4_quin_), 148.43 (C‐8a_quin_), 155.73 (C‐2_quin_), 166.28 (NH–C**═**O). Anal calcd for C_25_H_16_BrF_3_N_2_O: C, 60.38; H, 3.24; N, 5.63. Found: C, 60.51; H, 3.35; N, 5.52. ESI‐MS analysis for [C_25_H_16_BrF_3_N_2_O** +** H^+^]: Calc.: 499.0454 *m*/*z*, exp.: 499.0456 *m*/*z*. Purity: 98.34%.

##### 4.1.4.28 *(E)‐*2‐(3,4‐Dichlorostyryl)‐*N*‐(4‐(Trifluoromethyl)Phenyl)Quinoline‐4‐Carboxamide (4ab)

Purified by flash column chromatography (ethyl acetate/cyclohexane, 1:1). White solid; yield: 49%; m.p.: 276°C–278°C. ¹H NMR (600 MHz, DMSO‐d₆) *δ*: 7.63–7.69 (m, 3H, 2″/5″/6″‐CH_styryl_), 7.75–7.84 (m, 4H, 2′/6′‐CH_phenyl_, CH**═**CH‐Ph, 6‐CH_quin_), 7.93 (d, 1H, *J* **=** *16.2 Hz*, CH**═**CH‐Ph), 8.01–8.11 (m, 6H, 3′/5′‐CH_phenyl_, 3/5/7/8‐CH_quin_), 11.20 (s, 1H, NH–C**═**O); ¹³C decoupling NMR (151 MHz, DMSO‐d₆) *δ*: 118.32 (CH‐5_quin_), 120.31 (2′/6′‐CH_phenyl_), 123.51 (C‐4a_quin_), 124.64 (q, *J* **=** *31.9 Hz*, 4′‐C_phenyl_‐CF_3_), 124.76 (q, *J* **=** *271.5 Hz*, CF_3_), 125.43 (CH‐3_quin_), 125.66 (b q, 3′/5′‐CH_phenyl_), 127.79 (CH‐6_quin_), 127.99 (6″‐CH_styryl_), 129.44 (CH**═**CH‐Ph), 129.74 (CH‐7_quin_), 130.81 (CH‐8_quin_), 130.92 (2″‐CH_styryl_), 131.46 (1″/5″‐CH_styryl_), 132.18 (4″‐C_styryl_), 132.94 (CH**═**CH‐Ph), 137.46 (3″‐C_styryl_), 142.60 (1′‐C_phenyl_), 142.74 (C‐4_quin_), 148.41 (C‐8a_quin_), 155.44 (C‐2_quin_), 166.64 (NH–C**═**O). Anal calcd for C_25_H_15_Cl_2_F_3_N_2_O: C, 61.62; H, 3.10; N, 5.75. Found: C, 61.74; H, 3.21; N, 5.63. ESI‐MS analysis for [C_25_H_15_Cl_2_F_3_N_2_O** +** H^+^]: Calc.: 487.0586 *m*/*z*, exp.: 487.0588 *m*/*z*. Purity: 99.50%.

##### 4.1.4.29 *(E)‐*2‐(2‐Nitrostyryl)‐*N*‐(4‐(Trifluoromethyl)Phenyl)Quinoline‐4‐Carboxamide (4ac)

Purified by flash column chromatography (ethyl acetate/cyclohexane, 1:1). Yellow solid; yield: 44%; m.p.: 265°C–267°C. ¹H NMR (600 MHz, DMSO‐d₆) *δ*: 7.57–7.63 (m, 2H, CH**═**CH‐Ph, 4″‐CH_styryl_), 7.66 (t, 1H, *J* **=** *7.2 Hz*, 6‐CH_quin_), 7.77–7.81 (m, 3H, 2′/6′‐CH_phenyl_, 5″‐CH_styryl_), 7.84 (t, 1H, *J* **=** *7.2 Hz*, 7‐CH_quin_), 8.01–8.05 (m, 3H, 3′/5′‐CH_phenyl_, 6″‐CH_styryl_), 8.07–8.13 (m, 4H, 3″‐CH_styryl_, 3/5/8‐CH_quin_), 8.13 (d, 1H, *J* **=** *16.2 Hz*, CH**═**CH‐Ph), 11.21 (s, 1H, NH–C**═**O); ¹³C decoupling NMR (151 MHz, DMSO‐d₆) *δ*: 118.65 (CH‐5_quin_), 120.33 (2′/6′‐CH_phenyl_), 123.66 (C‐4a_quin_), 124.63 (q, *J* **=** *32.2 Hz*, 4′‐C_phenyl_‐CF_3_), 124.76 (q, *J* **=** *272.0 Hz*, CF_3_), 125.06 (CH‐3_quin_), 125.43 (3″‐CH_styryl_), 126.66 (b q, 3′/5′‐CH_phenyl_), 128.19 (CH‐6_quin_), 129.02 (CH‐7_quin_), 129.90 (CH‐8_quin_), 129.96 (1″‐C_styryl_), 130.15 (4″‐CH_styryl_), 130.98 (6″‐CH_styryl_), 131.34 (5″‐CH_styryl_), 133.09 (CH**═**CH‐Ph), 134.12 (CH**═**CH‐Ph), 142.74 (1′‐C_phenyl_), 142.85 (C‐4_quin_), 148.35 (2″‐C_styryl_), 148.83 (C‐8a_quin_), 155.07 (C‐2_quin_), 166.18 (NH–C**═**O). Anal calcd for C_25_H_16_F_3_N_3_O_3_: C, 64.80; H, 3.48; N, 9.07. Found: C, 64.92; H, 3.60; N, 8.99. ESI‐MS analysis for [C_25_H_16_F_3_N_3_O_3_ **+** H^+^]: Calc.: 464.1217 *m*/*z*, exp.: 464.1217 *m*/*z*. Purity: 98.29%.

##### 4.1.4.30 *(E)‐*2‐(3‐Nitrostyryl)‐*N*‐(4‐(Trifluoromethyl)Phenyl)Quinoline‐4‐Carboxamide (4ad)

Purified by flash column chromatography (ethyl acetate/cyclohexane, 1:1). Yellow solid; yield: 82%; m.p.: 261°C–263°C. ¹H NMR (600 MHz, DMSO‐d₆) *δ*: 7.65–7.48 (m, 6H, 2′/3′/5′/6′‐CH_phenyl_, 5″‐CH_styryl_, 6‐CH_quin_), 8.02‐8.24 (m, 8H, 2″/4″/6″‐CH_styryl_, 5/7/8‐CH_quin_, CH**═**CH‐Ph, CH**═**CH‐Ph), 8.55 (b s, 1H, 3‐CH_quin_), 11.21 (s, 1H, NH–C**═**O); ¹³C decoupling NMR (151 MHz, DMSO‐d₆) *δ*: 118.37 (CH‐5_quin_), 120.32 (2′/6′‐CH_phenyl_), 122.28 (2″‐CH_styryl_), 123.57 (C‐4a_quin_), 123.61 (CH‐3_quin_), 124.64 (q, *J* **=** *31.6 Hz*, 4′‐C_phenyl_‐CF_3_), 124.77 (q, *J* **=** *272.6 Hz*, CF_3_), 125.45 (4″‐CH_styryl_), 126.67 (b q, 3′/5′‐CH_phenyl_), 128.06 (CH‐6_quin_), 129.79 (CH**═**CH‐Ph), 130.93 (CH‐7_quin_), 131.38 (CH‐8_quin_), 133.27 (5″‐CH_styryl_), 133.67 (CH**═**CH‐Ph), 138.43 (1″‐C_styryl_), 142.63 (1′‐C_phenyl_), 142.75 (C‐4_quin_), 148.41 (3″‐C_styryl_), 148.86 (C‐8a_quin_), 155.39 (C‐2_quin_), 166.25 (NH–C**═**O). Anal calcd for C_25_H_16_F_3_N_3_O_3_: C, 64.80; H, 3.48; N, 9.07. Found: C, 64.93; H, 3.59; N, 9.00. ESI‐MS analysis for [C_25_H_16_F_3_N_3_O_3_ **+** H^+^]: Calc.: 464.1217 *m*/*z*, exp.: 464.1219 *m*/*z*. Purity: 99.09%.

##### 4.1.4.31 *(E)‐N*‐(4‐Chlorophenyl)‐2‐(4‐Methoxystyryl)Quinoline‐4‐Carboxamide (4ae)

Purified by crystallization from ethanol. White solid; yield: 53%; m.p.: 262°C–264°C. ¹H NMR (400 MHz, DMSO‐d₆) *δ*: 3.80 (s, 3H, 4″‐OCH₃_styryl_), 7.01 (d, 2H, *J* **=** *8.8 Hz*, 3″/5″‐CH_styryl_), 7.41 (d, 1H, *J* **=** *16.4 Hz*, CH**═**CH‐Ph), 7.48 (d, 2H, *J* **=** *8.8 Hz*, 3′/5′‐CH_phenyl_), 7.60 (t, 1H, *J* **=** *7.6 Hz*, 6‐CH_quin_), 7.71 (d, 2H, *J* **=** *8.8 Hz*, 2″/6″‐CH_styryl_), 7.80 (t, 1H, *J* **=** *7.6 Hz*, 7‐CH_quin_), 7.84–7.93 (m, 3H, 2′/6′‐CH_phenyl_, CH**═**CH‐Ph), 8.04–8.08 (m, 3H, 3/5/8‐CH_quin_), 10.95 (s, 1H, NH–C**═**O); ¹³C decoupling NMR (100 MHz, DMSO‐d₆) *δ*: 55.73 (4″‐OCH₃_styryl_), 114.91 (3″/5″‐CH_styryl_), 117.91 (CH‐5_quin_), 121.98 (2′/6′‐CH_phenyl_), 123.37 (C‐4a_quin_), 125.47 (CH‐3_quin_), 126.37 (CH‐6_quin_), 127.35 (CH‐7_quin_), 128.25 (1″‐C_styryl_), 129.26 (3′/5′‐CH_phenyl_, CH‐8_quin_), 129.35 (2″/6″‐CH_styryl_), 129.55 (4′‐C_phenyl_), 130.66 (CH**═**CH‐Ph), 135.33 (CH**═**CH‐Ph), 138.26 (1′‐C_phenyl_), 142.61 (C‐4_quin_), 148.52 (C‐8a_quin_), 156.33 (C‐2_quin_), 160.50 (4″‐C_styryl_), 165.99 (NH–C**═**O). Anal calcd for C_25_H_19_ClN_2_O_2_: C, 72.37; H, 4.62; N, 6.75. Found: C, 72.50; H, 4.75; N, 6.68. ESI‐MS analysis for [C_25_H_19_ClN_2_O_2_ **+** Na^+^]: Calc.: 437.1027 *m*/*z*, exp.: 437.1026 *m*/*z*. Purity: 99.32%.

##### 4.1.4.32 *(E)‐N*‐(4‐Chlorophenyl)‐2‐(4‐Chlorostyryl)Quinoline‐4‐Carboxamide (4af)

Purified by flash column chromatography (ethyl acetate/cyclohexane, 1:1). White solid; yield: 54%; m.p.: 274°C–276°C. ¹H NMR (600 MHz, DMSO‐d₆) δ: 7.46–7.50 (m, 4H, 2″/3″/5″/6″‐CH_styryl_), 7.57 (d, 1H, *J* **=** *16.2 Hz*, CH**═**CH‐Ph), 7.62 (t, 1H, *J* **=** *7.2 Hz*, 6‐CH_quin_), 7.77–7.85 (m, 5H, 2′/3′/5′/6′‐CH_phenyl_, 7‐CH_quin_), 7.95 (d, 1H, *J* **=** *16.2 Hz*, CH** =** CH‐Ph), 8.07–8.11 (m, 3H, 3/5/8‐CH_quin_), 10.99 (s, 1H, NH–C**═**O); ¹³C decoupling NMR (151 MHz, DMSO‐d₆) *δ*: 118.08 (CH‐5_quin_), 121.91 (2′/6′‐CH_phenyl_), 123.53 (C‐4a_quin_), 125.48 (CH‐3_quin_), 127.74 (CH‐6_quin_), 128.22 (1″‐C_styryl_), 129.25 (3′/5′‐CH_phenyl_, CH‐8_quin_), 129.40 (2″/6″‐CH_styryl_), 129.46 (3″/5″‐CH_styryl_), 129.67 (CH**═**CH‐Ph), 130.78 (CH‐7_quin_), 133.76 (4′‐C_phenyl_), 134.16 (CH**═**CH‐Ph), 135.51 (4″‐C_styryl_), 138.20 (1′‐C_phenyl_), 142.76 (C‐4_quin_), 148.42 (C‐8a_quin_), 155.74 (C‐2_quin_), 165.86 (NH–C**═**O). Anal calcd for C_24_H_16_Cl_2_N_2_O: C, 68.75; H, 3.85; N, 6.68. Found: C, 68.87; H, 3.98; N, 6.59. ESI‐MS analysis for [C_24_H_16_Cl_2_N_2_O** +** H^+^]: Calc.: 419.0712 *m*/*z*, exp.: 419.0714 *m*/*z*. Purity: 99.51%.

##### 4.1.4.33 *(E)‐N*‐(4‐Chlorophenyl)‐2‐(3,4‐Dichlorostyryl)Quinoline‐4‐Carboxamide (4ag)

Purified by flash column chromatography (ethyl acetate/cyclohexane, 1:1). White solid; yield: 42%; m.p.: 271°C–273°C. ¹H NMR (600 MHz, DMSO‐d₆) *δ*: 7.45–7.48 (m, 2H, 5″/6″‐CH_styryl_), 7.62–7.69 (m, 3H, 2″‐CH_styryl_, 3′/5′‐CH_phenyl_), 7.74–7.85 (m, 4H, 2′/6′‐CH_phenyl_, 6‐CH_quin_, CH**═**CH‐Ph), 7.92 (d, 1H, *J* **=** *16.2 Hz*, CH**═**CH‐Ph), 8.04–8.08 (m, 4H, 3/5/7/8‐CH_quin_), 10.97 (s, 1H, NH–C**═**O); ¹³C decoupling NMR (151 MHz, DMSO‐d₆) *δ*: 118.23 (CH‐5_quin_), 121.89 (2′/6′‐CH_phenyl_), 123.60 (C‐4a_quin_), 125.50 (CH‐3_quin_), 127.78 (CH‐6_quin_), 127.91 (6″‐CH_styryl_), 128.25 (1″‐C_styryl_), 129.27 (3′/5′‐CH_phenyl_), 129.44 (CH**═**CH‐Ph), 129.72 (4′‐C_phenyl_), 130.85 (CH‐7_quin_), 130.87 (CH‐8_quin_), 131.42 (2″‐CH_styryl_), 131.46 (5″‐CH_styryl_), 132.18 (3″‐C_styryl_), 132.89 (CH**═**CH‐Ph), 137.48 (4″‐C_styryl_), 138.17 (1′‐C_phenyl_), 142.84 (C‐4_quin_), 148.48 (C‐8a_quin_), 155.44 (C‐2_quin_), 165.81 (NH–C**═**O). Anal calcd for C_24_H_15_Cl_3_N_2_O: C, 63.53; H, 3.33; N, 6.17. Found: C, 63.64; H, 3.45; N, 6.05. ESI‐MS analysis for [C_24_H_15_Cl_3_N_2_O** +** H^+^]: Calc.: 453.0323 *m*/*z*, exp.: 453.0324 *m*/*z*. Purity: 99.86%.

##### 4.1.4.34 *(E)‐N*‐(4‐Chlorophenyl)‐2‐(2‐Nitrostyryl)Quinoline‐4‐Carboxamide (4ah)

Purified by flash column chromatography (ethyl acetate/cyclohexane, 1:1). Yellow solid; yield: 83%; m.p.: 254°C–256°C. ¹H NMR (600 MHz, DMSO‐d₆) *δ*: 7.45–7.47 (m, 2H, 3′/5′‐CH_phenyl_), 7.57–7.67 (m, 3H, 2′/6′‐CH_phenyl_, 4″‐CH_styryl_), 7.78–7.84 (m, 4H, 5″/6″‐CH_styryl_, 6/7‐CH_quin_), 8.03–8.11 (m, 5H, CH**═**CH‐Ph, 3″‐CH_styryl_, 3/5/8‐CH_quin_), 8.12 (d, 1H, *J* **=** *16.2 Hz*, CH**═**CH‐Ph), 10.99 (s, 1H, NH–C**═**O); ¹³C decoupling NMR (151 MHz, DMSO‐d₆) *δ*: 118.59 (CH‐5_quin_), 121.91 (2′/6′‐CH_phenyl_), 123.74 (C‐4a_quin_), 125.06 (CH‐3_quin_), 125.50 (3″‐CH_styryl_), 128.12 (CH‐6_quin_), 128.24 (CH‐7_quin_), 129.02 (CH‐8_quin_), 129.26 (3′/5′‐CH_phenyl_), 129.87 (4′‐C_phenyl_), 129.91 (1″‐C_styryl_), 130.13 (4″‐CH_styryl_), 130.93 (6″‐CH_styryl_), 131.36 (5″‐CH_styryl_), 133.11 (CH** =** CH‐Ph), 134.12 (CH**═**CH‐Ph), 138.16 (1′‐C_phenyl_), 143.09 (C‐4_quin_), 148.34 (2″‐C_styryl_), 148.84 (C‐8a_quin_), 155.07 (C‐2_quin_), 165.75 (NH–C**═**O). Anal calcd for C_24_H_16_ClN_3_O_3_: C, 67.06; H, 3.75; N, 9.78. Found: C, 67.20; H, 3.87; N, 9.65. ESI‐MS analysis for [C_24_H_16_ClN_3_O_3_ **+** H^+^]: Calc.: 430.0953 *m*/*z*, exp.: 430.0956 *m*/*z*. Purity: 98.35%.

### 4.2 Biological Assays

#### 4.2.1 Cell Culture

To test the newly synthesized compounds, various human cell lines were employed. HD‐MB03 cells were purchased from DSMZ (Braunschweig, Germany), while A549, MDA‐MB‐231, RPMI‐8420, SU‐DHL‐8, TOLEDO, VL51, SU‐DHL‐1, SU‐DHL‐18, and KM‐H2 were obtained from ATCC (Manassas, VA, USA). Cells were cultured in DMEM (for MDA‐MB‐231 and A549) or RPMI‐1640 (for RPMI‐8420, SU‐DHL‐8, TOLEDO, VL51, SU‐DHL‐1, SU‐DHL‐18, KM‐H2, and HD‐MB03) media (Gibco, Milan, Italy). Both media were supplemented with 115 units/mL penicillin G (Gibco, Milan, Italy), 115 µg/mL streptomycin (Invitrogen, Milan, Italy), and 10% fetal bovine serum (Invitrogen, Milan, Italy). Peripheral blood mononuclear cells (PBMCs) were isolated from healthy donors as previously described [37]. For cytotoxicity assays, PBMCs were resuspended at 5 × 10⁵ cells/mL in complete RPMI medium with or without 2.5 µg/mL phytohemagglutinin (PHA) (Irvine Scientific) to stimulate T‐cell activation and proliferation. All cell lines were maintained at 37°C in a humidified atmosphere containing 5% CO₂. All compounds were dissolved in DMSO at a 10 mM stock concentration. For dose–response assays, cells were seeded in 96‐well plates at densities optimized for each cell line: HD‐MB03 at 10,000 cells/well, A549 at 4000 cells/well, and MDA‐MB‐231 at 7000 cells/well. All other cell lines were seeded at 20,000 cells/well in a final volume of 100 µL per well. After 24 h, cells were treated with a 6‐point serial dilution of compounds starting from 10 µM, in triplicate for statistical analysis. Seventy‐2 h posttreatment, 10 µL of resazurin solution (100 µg/mL) was added to each well, and cells were incubated for an additional 3–6 h. Fluorescence was measured using a Spark 10 M microplate reader (Tecan Group Ltd., Männedorf, Switzerland) with excitation at 535 nm and emission at 600 nm. The IC₅₀ was defined as the concentration of compound required to inhibit cell proliferation by 50% compared to cells treated with the highest DMSO concentration.

#### 4.2.2 Apoptosis Assay

For apoptosis evaluation, cells were plated in 12‐well plates at 0.2 × 10⁶ cells/mL in 1 mL of complete medium. After 24 h, cells were treated with the indicated compound concentrations. After 48 h, cells were harvested, centrifuged, and stained with Annexin V‐FITC and propidium iodide (PI) using the Annexin‐V Fluos kit (Roche Diagnostics), following the manufacturer's instructions. Fluorescence was analyzed using a Coulter Cytomics FC500 flow cytometer (Beckman Coulter) with detection in the FL1 and FL3 channels, respectively.

#### 4.2.3 Cell‐Cycle Analysis

Cells were plated in 12‐well plates at 0.2 × 10⁶ cells/mL in 1 mL of complete medium. After 24 h, cells were treated with the specified compound concentrations. Following an additional 24 h, cells were harvested, centrifuged, and fixed in 70% ice‐cold ethanol. Cells were then stained and processed as previously described [38]. Data acquisition was performed using a Coulter Cytomics FC500 flow cytometer (Beckman Coulter).

#### 4.2.4 EdU Incorporation Assay

For evaluation of DNA synthesis, cells were plated in 12‐well plates at 0.2 × 10⁶ cells/mL in 1 mL of complete medium. After 24 h, cells were treated with the indicated compound concentrations. EdU (5‐ethynyl‐2‐deoxyuridine) incorporation was performed according to the Baseclick EdU Flow Cytometry Kit instructions (Sigma Aldrich, St. Louis, MO, USA) as previously described [39]. EdU‐FITC incorporation was detected using a CytoFLEX flow cytometer (Beckman Coulter, Brea, CA, USA) and analyzed with FlowJo software v.10.7.1 (BD Biosciences).

#### 4.2.5 Mitochondrial Membrane Potential Analysis (TMRE)

Cells were plated in 12‐well plates at 0.2 × 10⁶ cells/mL in 1 mL of complete medium. After 24 h, cells were treated with the indicated compound concentrations. Following a further 24‐h incubation, cells were stained with TMRE (Invitrogen, US) [40] at 1 µM for 20 min. After washing with 1X PBS, fluorescence was detected using a CytoFLEX flow cytometer (Beckman Coulter) and analyzed with FlowJo software v.10.7.1.

#### 4.2.6 Mitochondrial ROS Detection (MitoSOX)

Cells were plated in 12‐well plates at 0.2 × 10⁶ cells/mL in 1 mL of complete medium. After 24 h, cells were treated with the indicated compound concentrations. Following a 24‐h treatment, cells were stained with 0.5 µM MitoSOX (Invitrogen, US) [40] for 20 min. After washing with PBS, fluorescence was measured using a CytoFLEX flow cytometer (Beckman Coulter) and analyzed with FlowJo software v.10.7.1.

#### 4.2.7 Statistical Analysis

All statistical analyses were conducted using GraphPad Prism 10 software (GraphPad, La Jolla, California). The data shown in bar graphs are expressed as the mean ± SEM. Asterisks above the bars indicate statistical significance relative to control cells or specific groups (specified in brackets, if applicable). The significance thresholds were defined as follows: *p < 0.05, **p < 0.01, ***p < 0.001, ****p < 0.0001.

## Conflicts of Interest

The authors declare no conflicts of interest.

## Supporting information

ArchPharm SupplMat QN REVISED.

ArchPharm SupplMat InChI 2020 QN.

## Data Availability

The data that support the findings of this study are available in the supporting materials of this article.
