# Supplementary material for: Microwave‐Enhanced Synthesis of 2‐Styrylquinoline‐4‐Carboxamides With Promising Anti‐Lymphoma Activity
Source: Arch Pharm (Weinheim). 2025 Nov 24;358(11):e70148. doi: 10.1002/ardp.70148 (PMC12645083; doi:10.1002/ardp.70148)
Supplement: Supplementary file 1 — ArchPharm SupplMat QN REVISED. [file ARDP-358-e70148-s001.doc]

**Supplemental Material: Novel Compounds and Chemical Characterizations**

**Microwave-Enhanced Synthesis of 2-Styrylquinoline-4-carboxamides with Promising Anti-Lymphoma Activity**

Sardo, Ignazio^1,§^, Manfreda, Lorenzo^2,§^, Titone, Giulia Maria^1^, Barreca, Marilia^1^, Bivacqua, Roberta^1^, Spanò, Virginia^1^, Amata, Sara^1^, Zanolli, Arianna^2,3^, Bortolozzi, Roberta^3,4*^ Raimondi, Maria Valeria^1,*^, Viola, Giampietro^2,3^, Barraja, Paola^1^, Montalbano, Alessandra^1^

1 Department of Biological, Chemical and Pharmaceutical Sciences and Technologies (STEBICEF), University of Palermo, Palermo, Italy

2 Department of Woman’s and Child’s Health, University of Padova, Padova, Italy

3 Istituto di Ricerca Pediatrica IRP, Fondazione Città della Speranza, Padova, Italy

4 Department of Pharmaceutical and Pharmacological Sciences, University of Padova, Italy

* Correspondence:

Prof Maria Valeria Raimondi, Department of Biological, Chemical and Pharmaceutical Sciences and Technologies (STEBICEF), University of Palermo, Via Archirafi 32, 90123, Palermo, Italy

E-mail: [mariavaleria.raimondi@unipa.it](mailto:mariavaleria.raimondi@unipa.it)

Dr Roberta Bortolozzi, Department of Pharmaceutical and Pharmacological Sciences, University of Padova, Via Marzolo 5, 35131, Padova, Italy

E-mail: [roberta.bortolozzi@unipd.it](mailto:roberta.bortolozzi@unipd.it)

^§^ co-first authors.

**Figure S1**. ^1^H NMR (DMSO-d_6_) of 2-Methylquinoline-4-carboxylic acid (**2**).


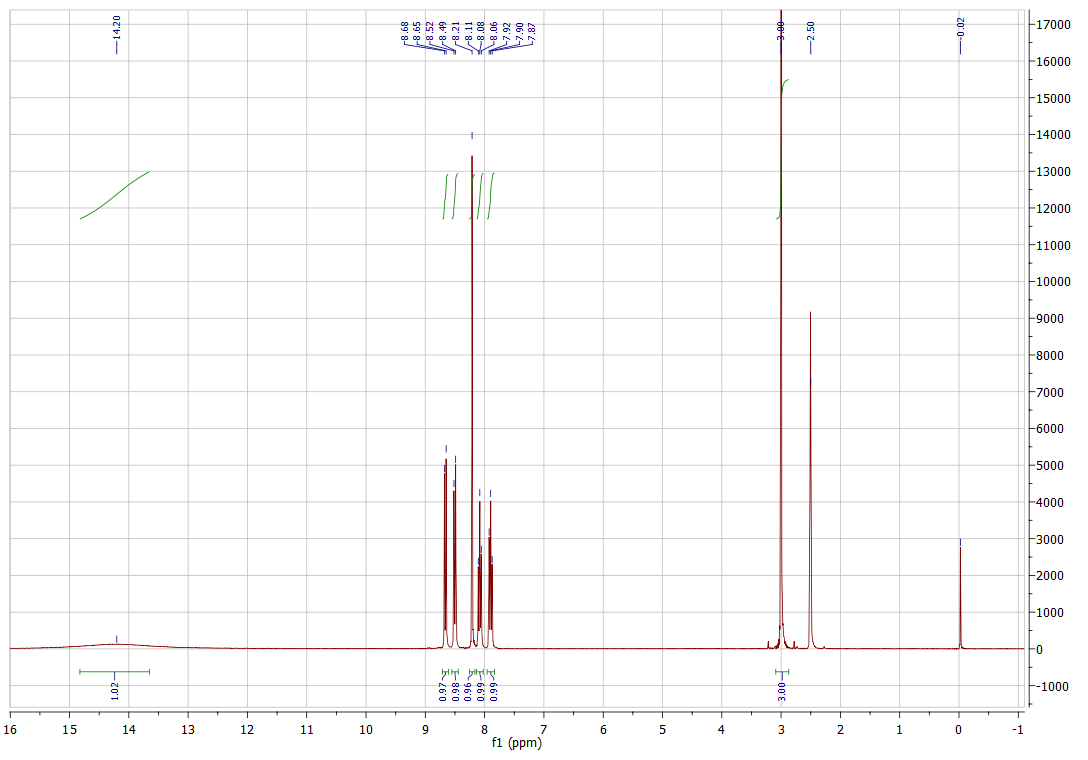


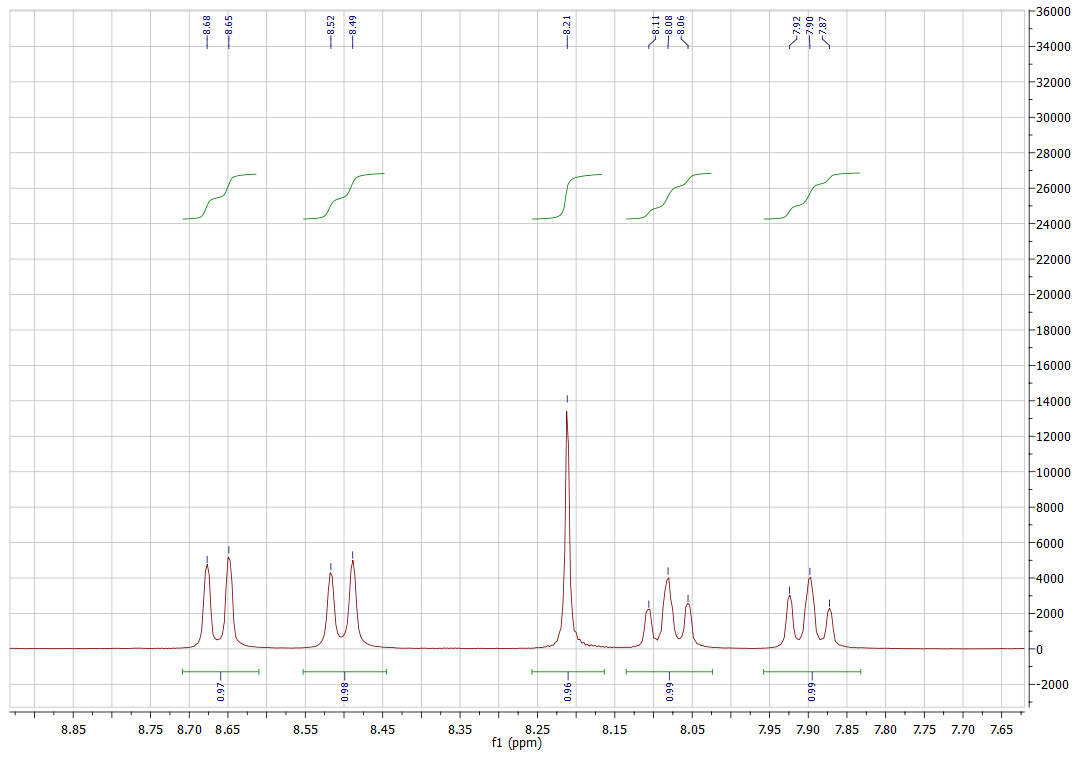


**Figure S2**. ^13^C APT NMR (DMSO-d_6_) of 2-Methylquinoline-4-carboxylic acid (**2**).


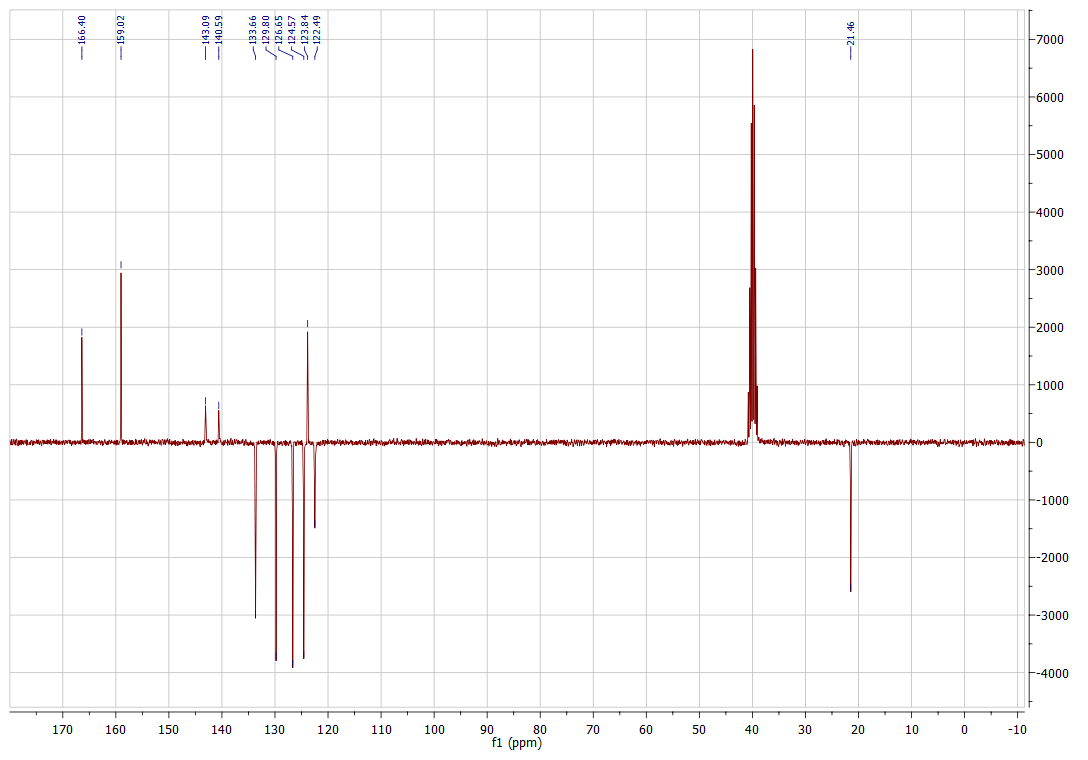


**Figure S3**. ^1^H NMR (DMSO-d_6_) of 2-Methyl-*N*-phenylquinoline-4-carboxamide (**3a**)


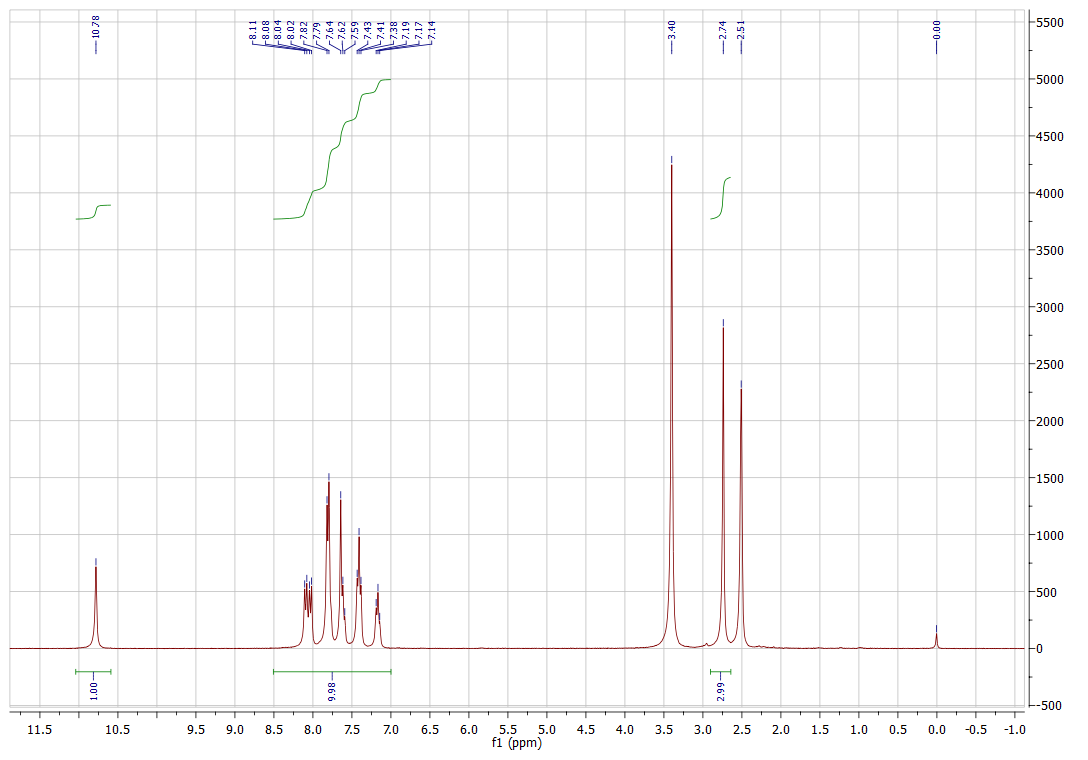


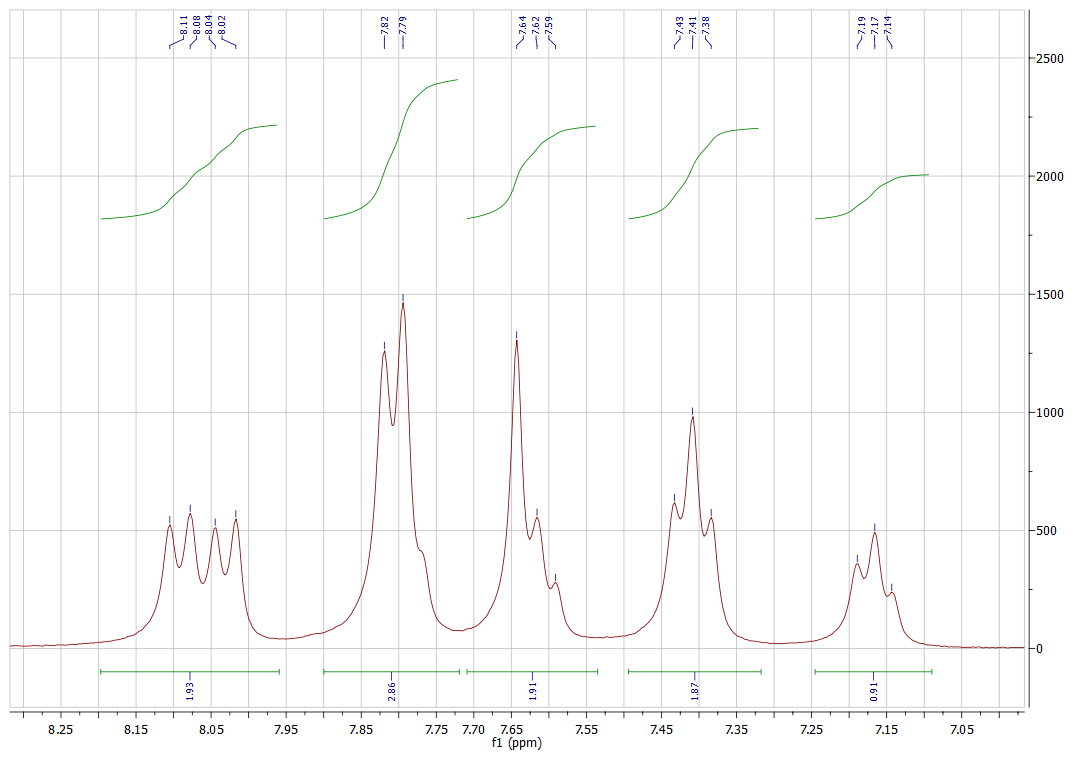


**Figure S4**. ^13^C APT NMR (DMSO-d_6_) of 2-Methyl-*N*-phenylquinoline-4-carboxamide (**3a**)


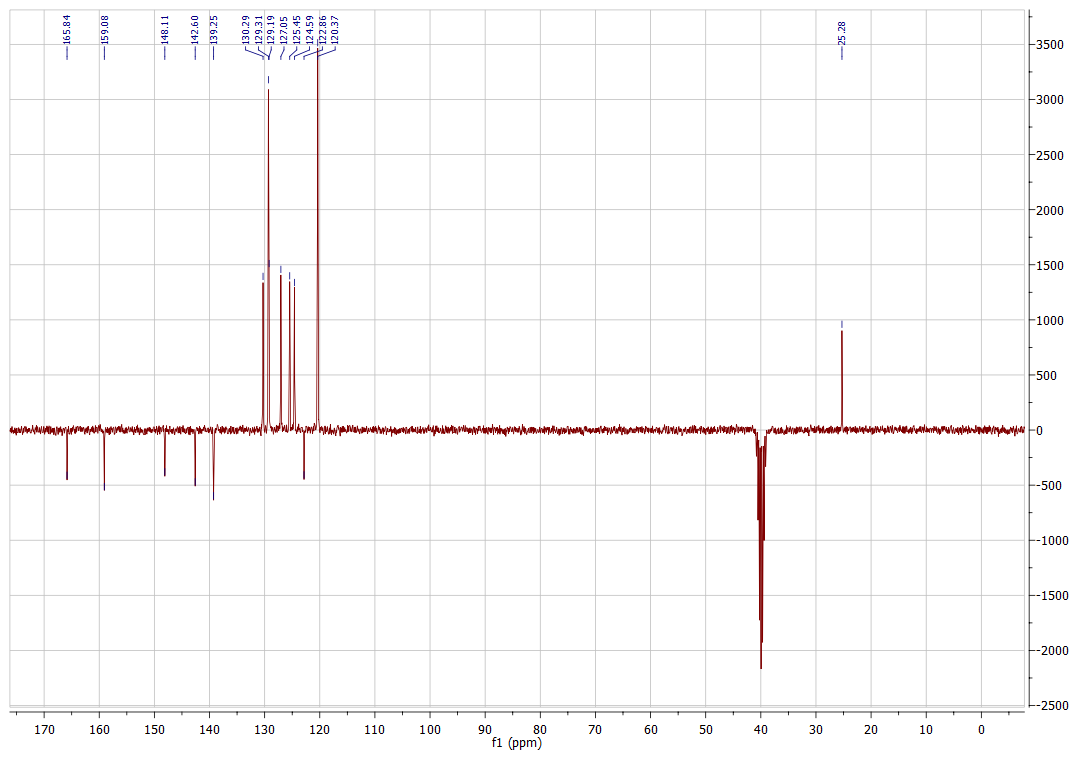


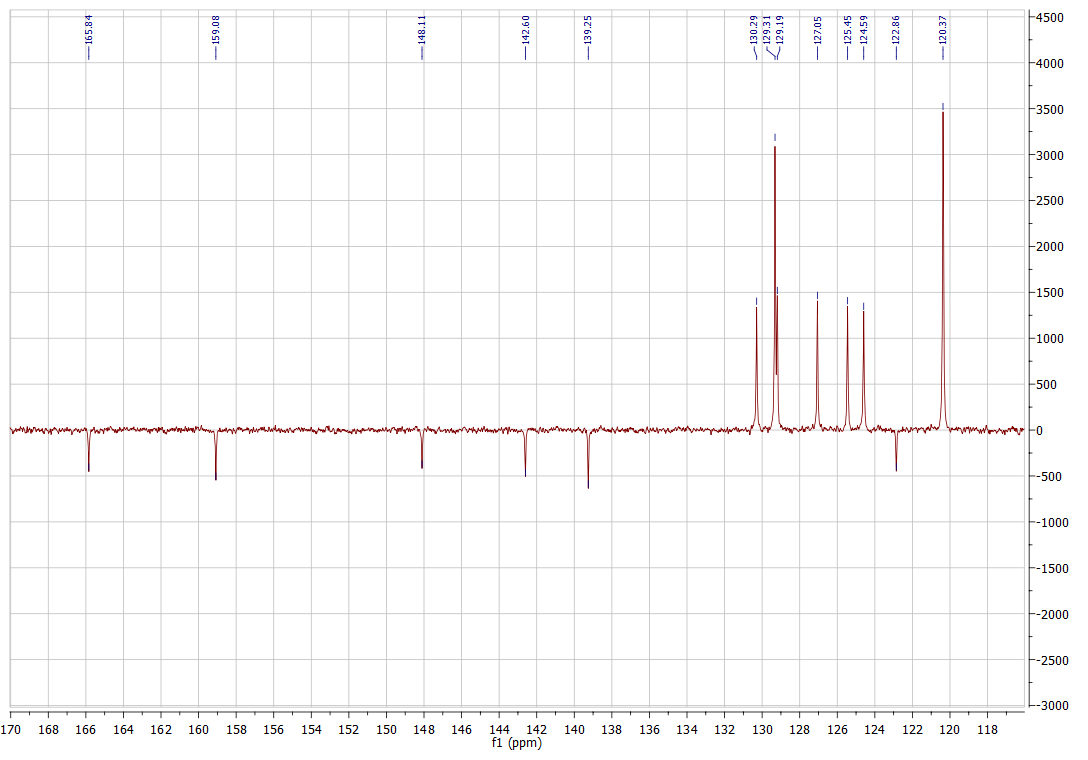


**Figure S5**. ^1^H NMR (DMSO-d_6_) of 2-Methyl-*N*-(3-methylphenyl)quinoline-4-carboxamide (**3b**)


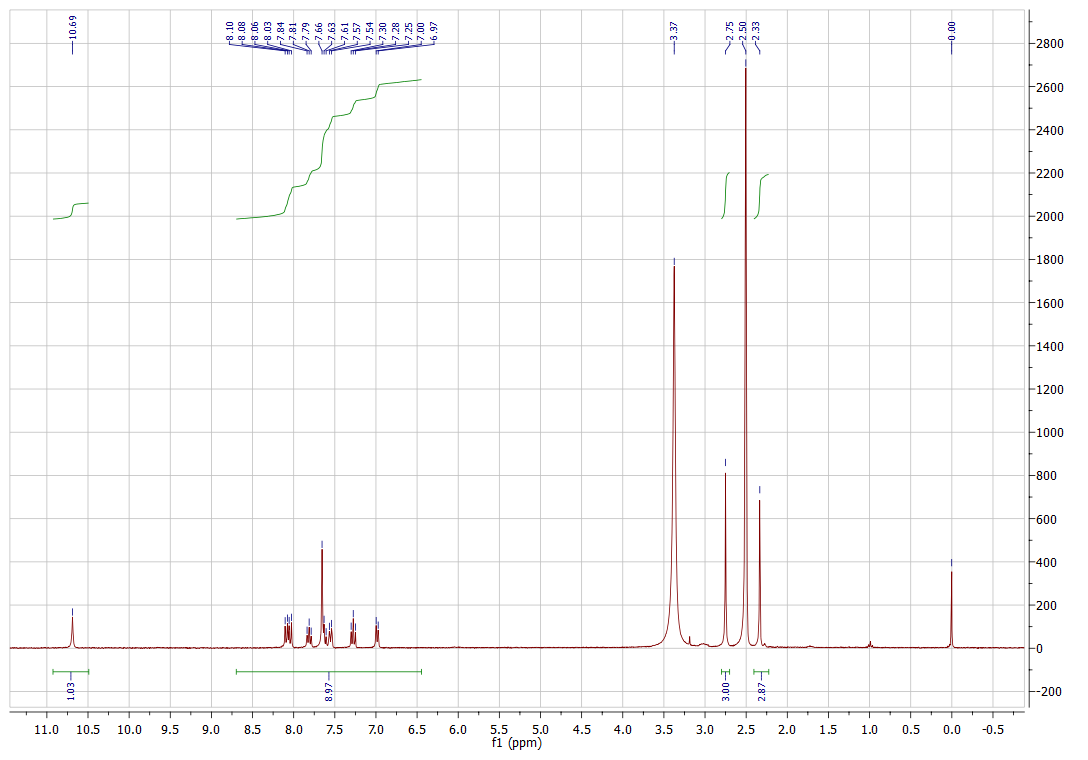


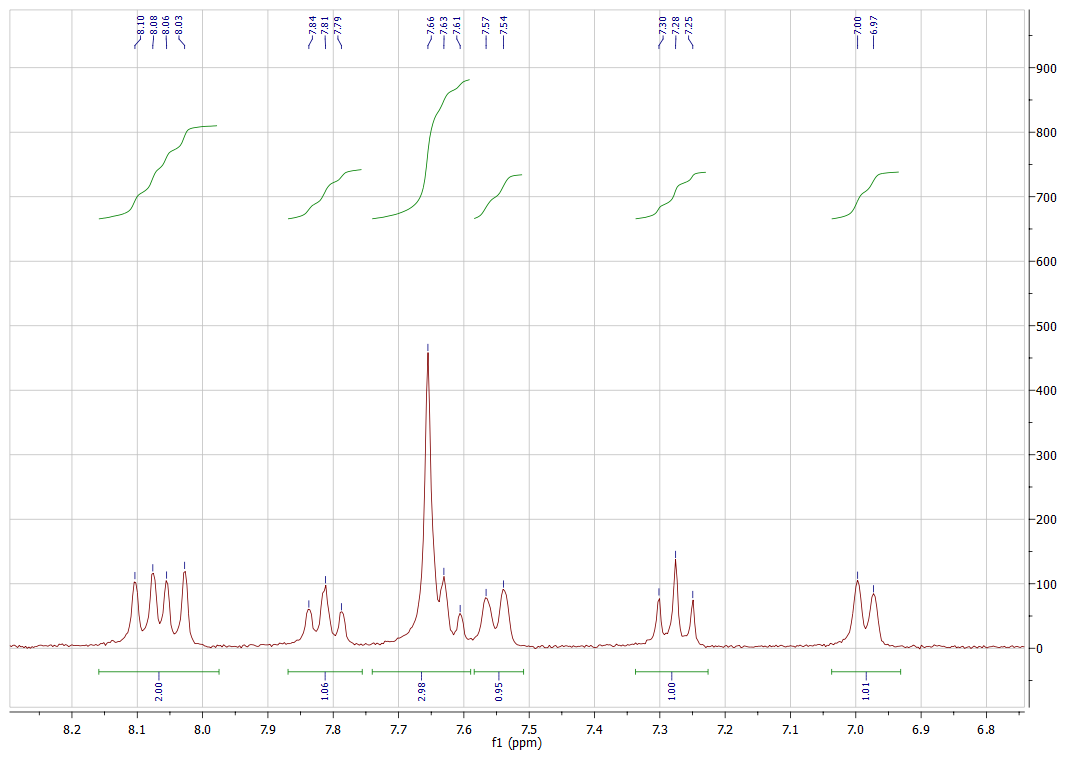


**Figure S6**. ^13^C APT NMR (DMSO-d_6_) of 2-Methyl-*N*-(3-methylphenyl)quinoline-4-carboxamide (**3b**)


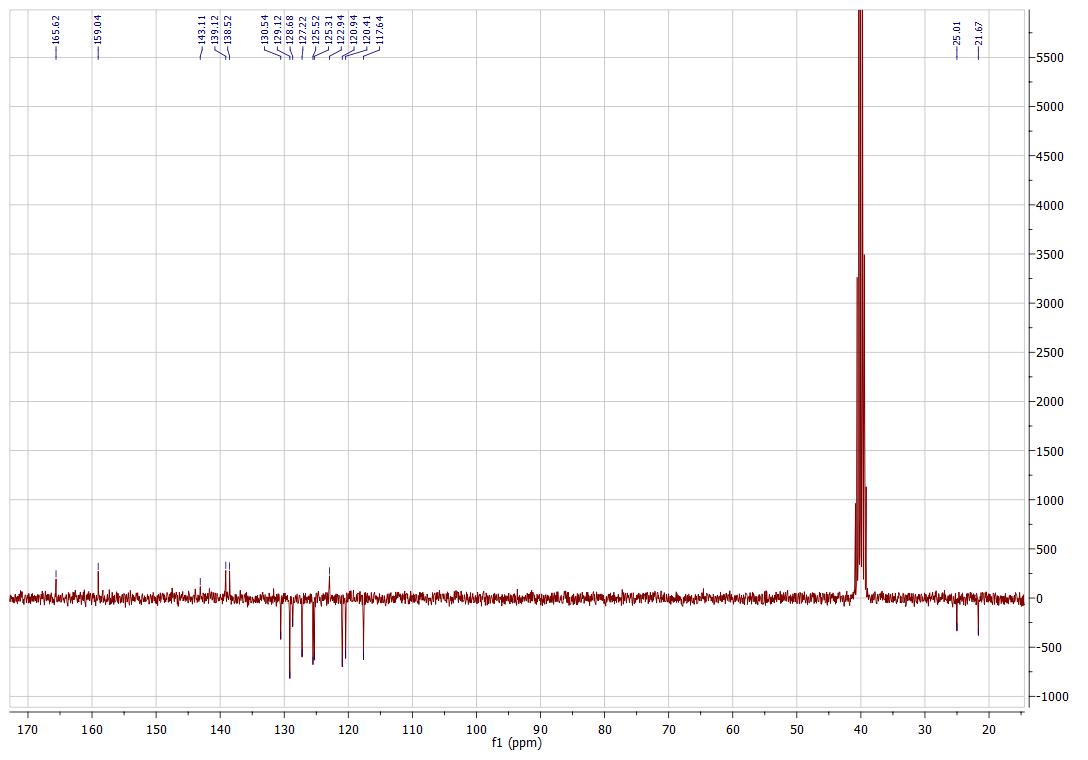


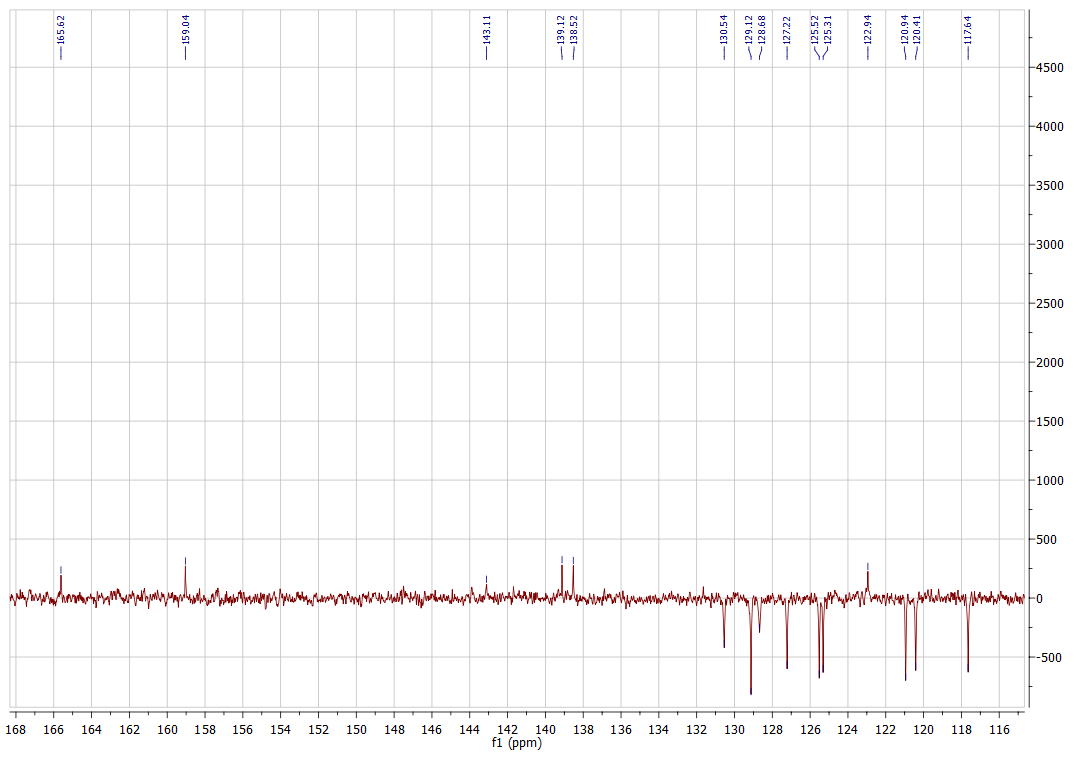


**Figure S7**. ^1^H NMR (DMSO-d_6_) of 2-Methyl-*N*-(4-methylphenyl)quinoline-4-carboxamide (**3c**)


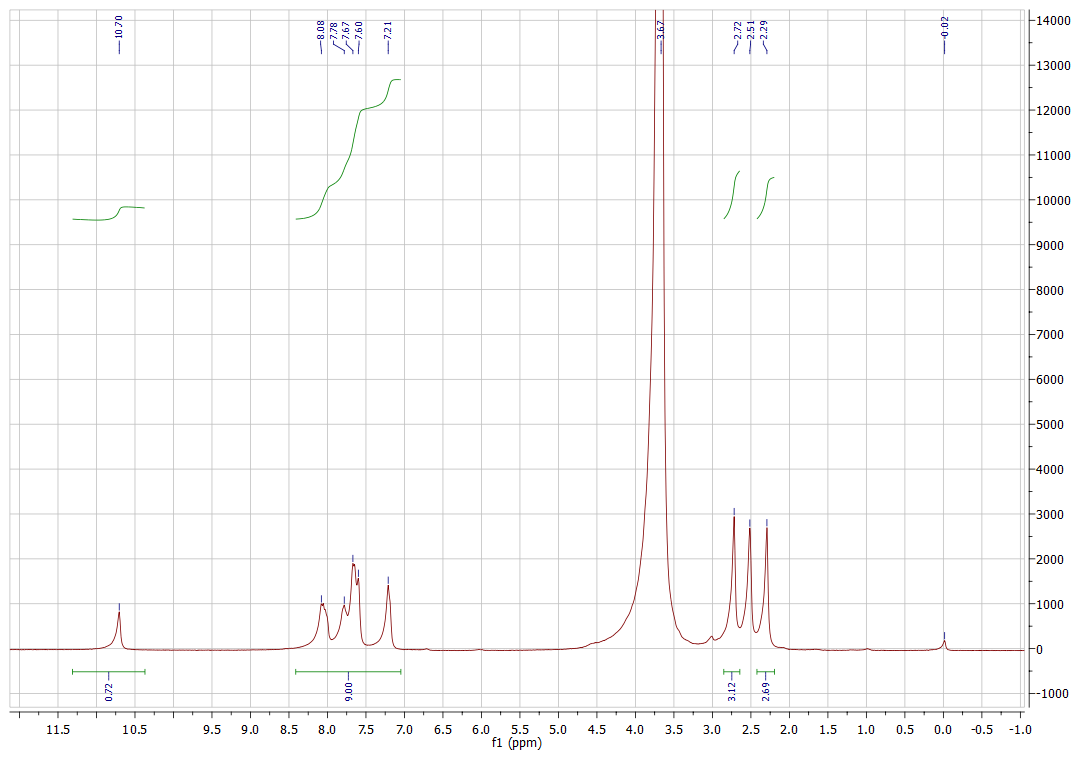


**Figure S8**. ^13^C APT NMR (DMSO-d_6_) of 2-Methyl-*N*-(4-methylphenyl)quinoline-4-carboxamide (**3c**)


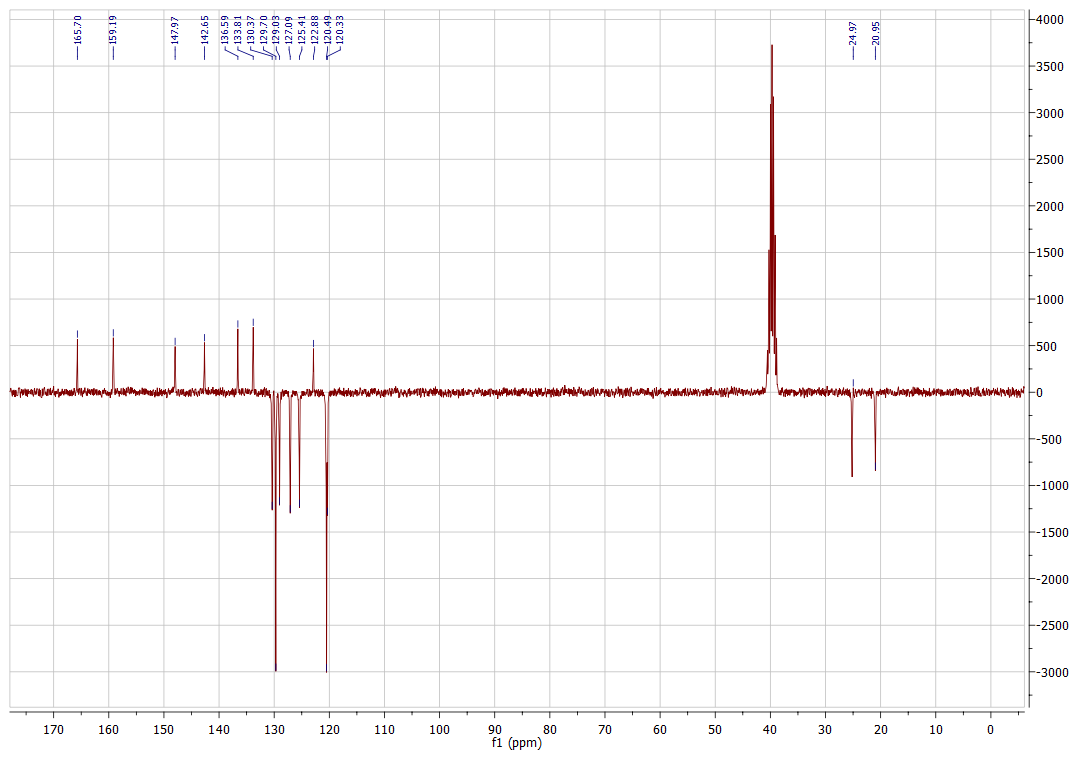


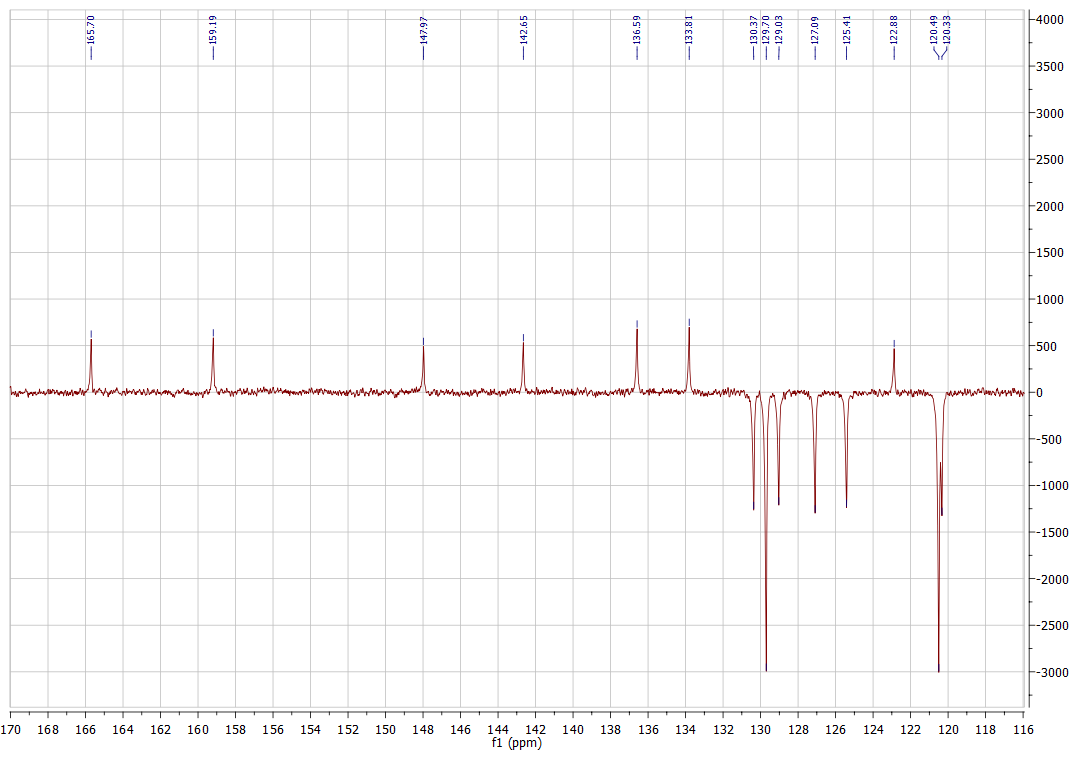


**Figure S9**. ^1^H NMR (DMSO-d_6_) of *N*-(2,6-Dimethylphenyl)-2-methylquinoline-4-carboxamide (**3d**)


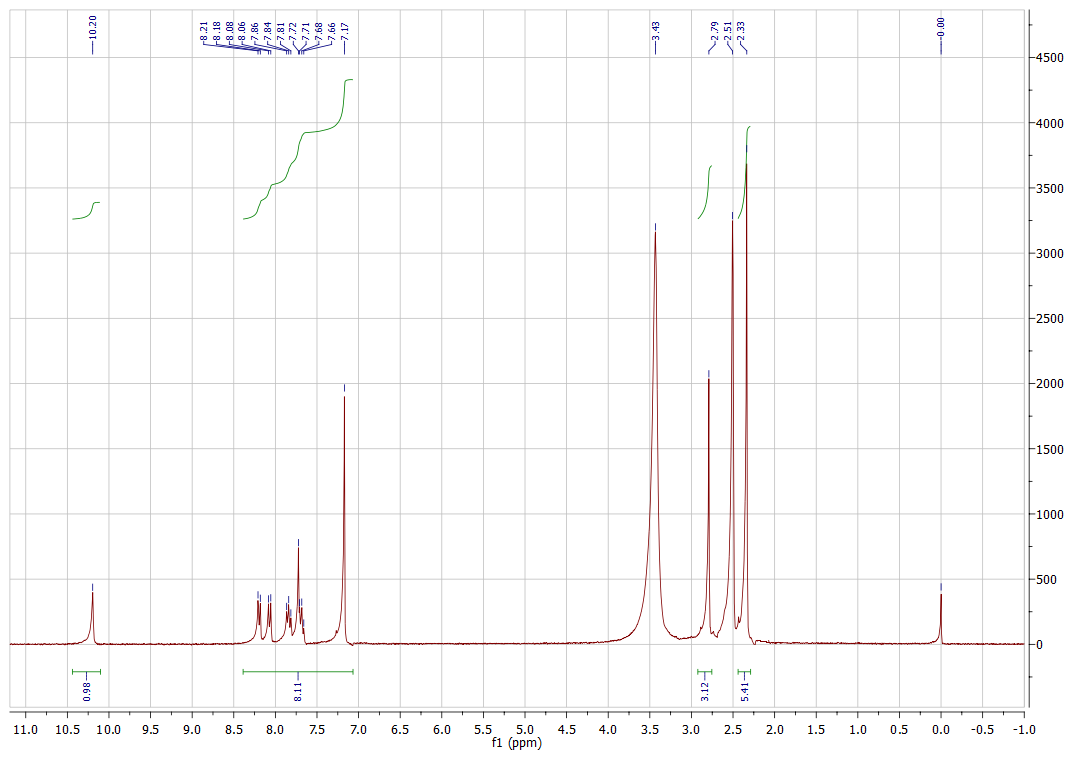


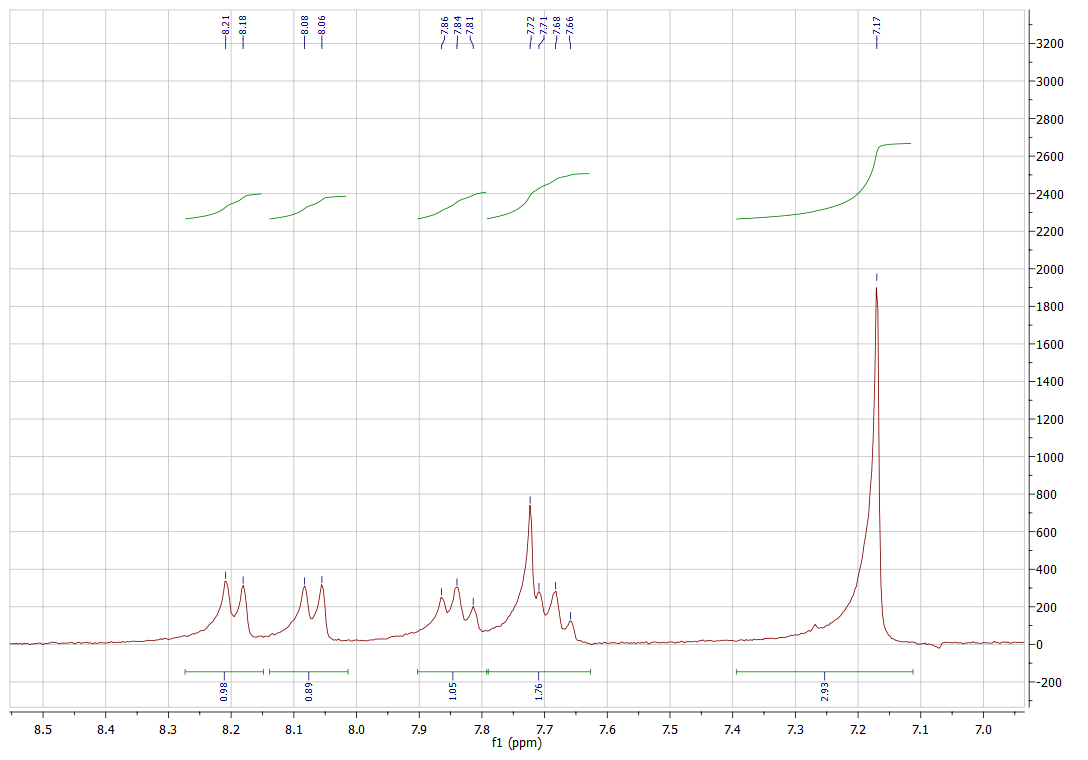


**Figure S10**. ^13^C APT NMR (DMSO-d_6_) of *N*-(2,6-Dimethylphenyl)-2-methylquinoline-4-carboxamide (**3d**)


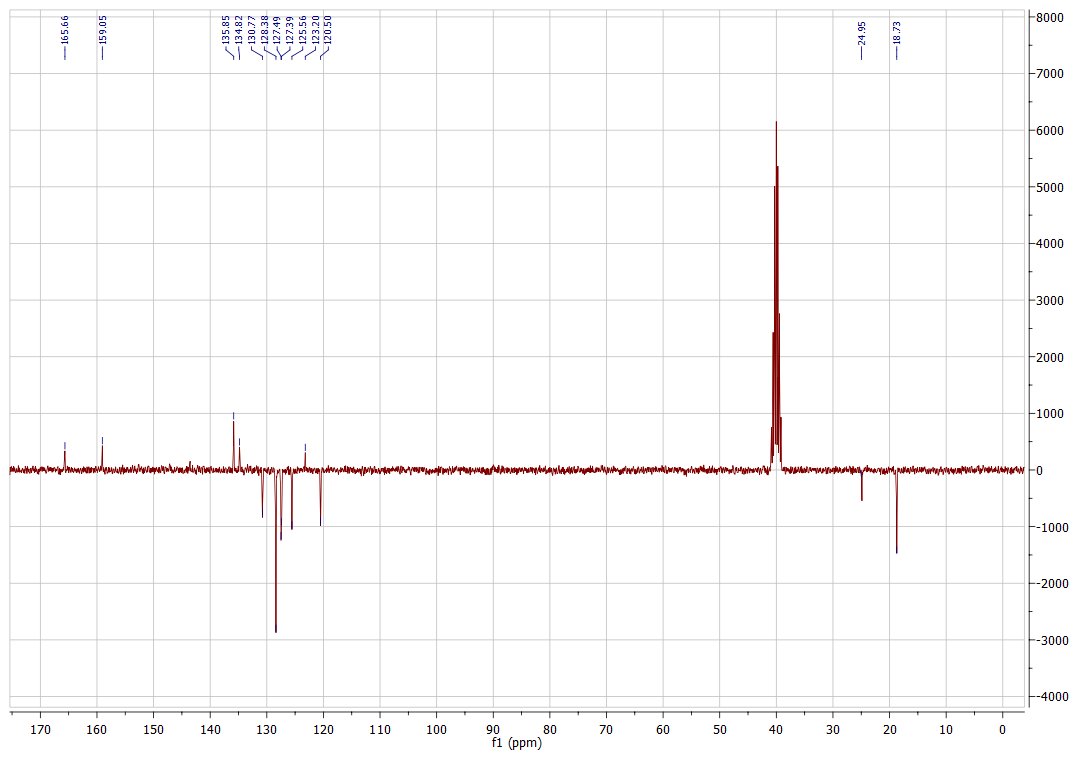


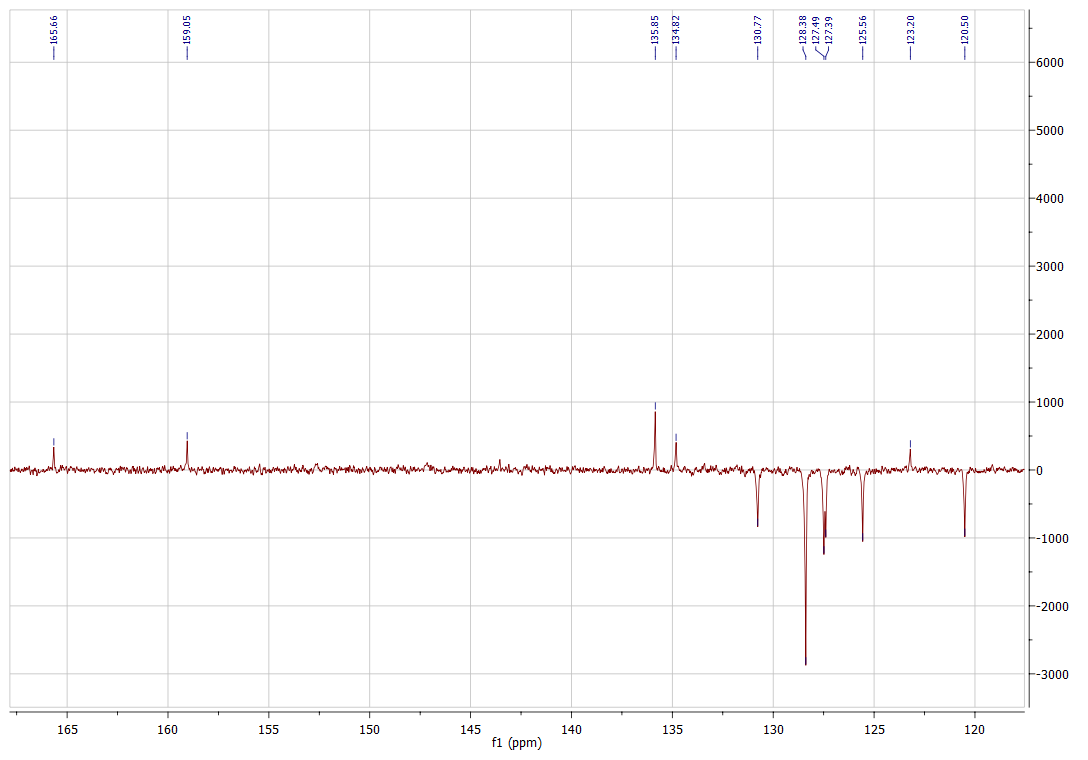


**Figure S11**. ^1^H NMR (DMSO-d_6_) of 2-Methyl-*N*-(3,4,5-trimethoxyphenyl)quinoline-4-carboxamide (**3e**)


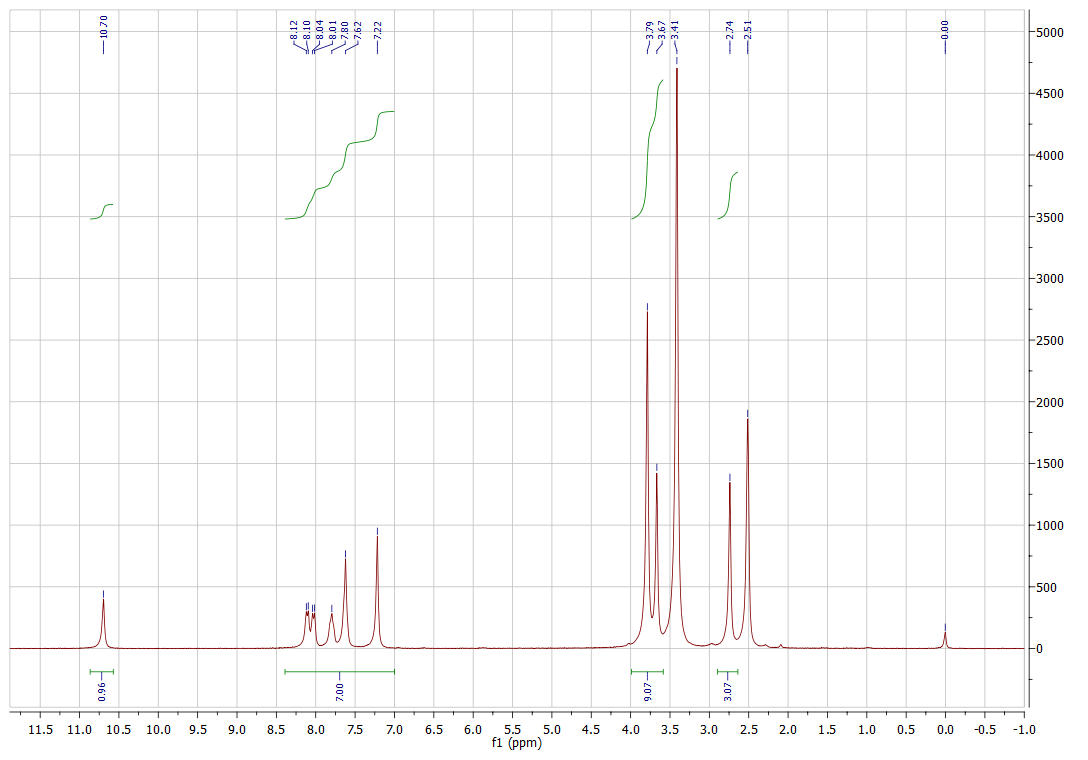


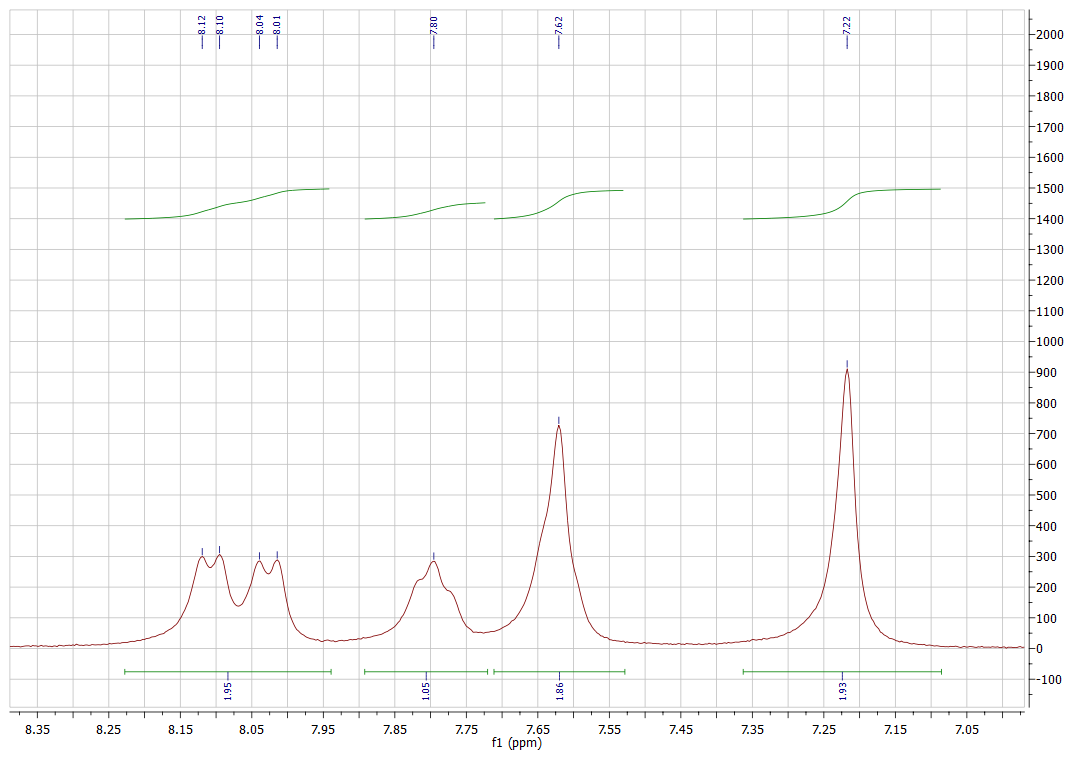


**Figure S12**. ^13^C APT NMR (DMSO-d_6_) of 2-Methyl-*N*-(3,4,5-trimethoxyphenyl)quinoline-4-carboxamide (**3e**)


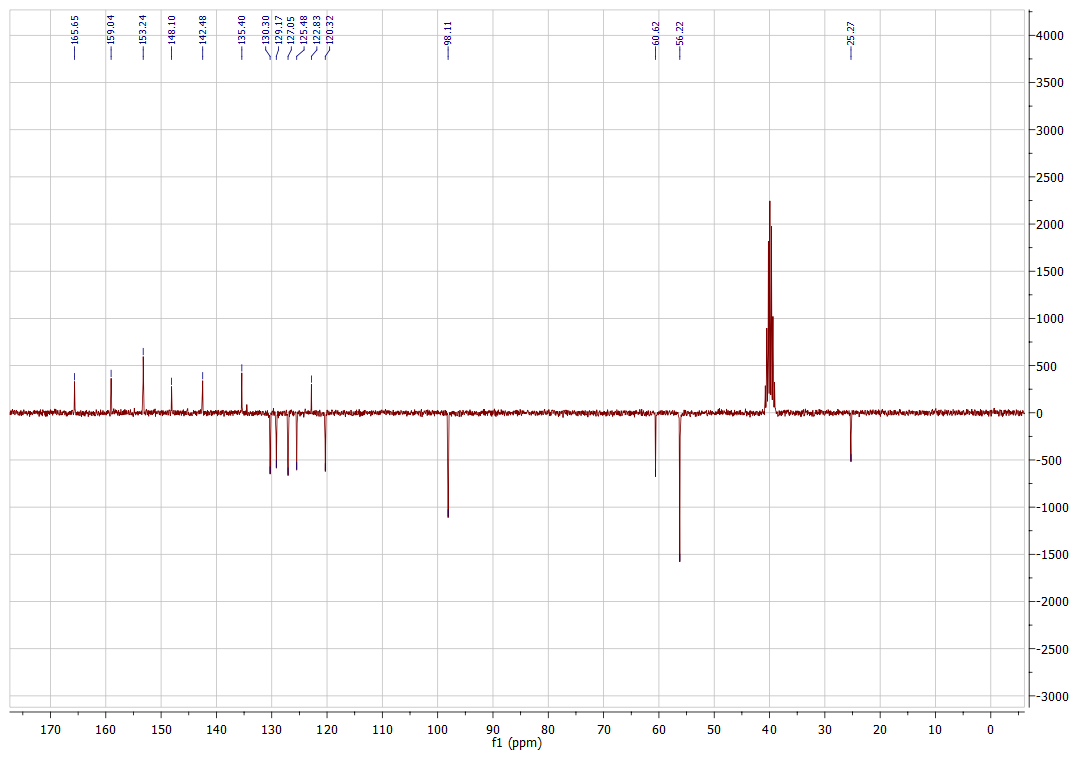


**Figure S13**. ^1^H NMR (DMSO-d_6_) of *N*-(3-Chlorophenyl)-2-methylquinoline-4-carboxamide (**3f**)


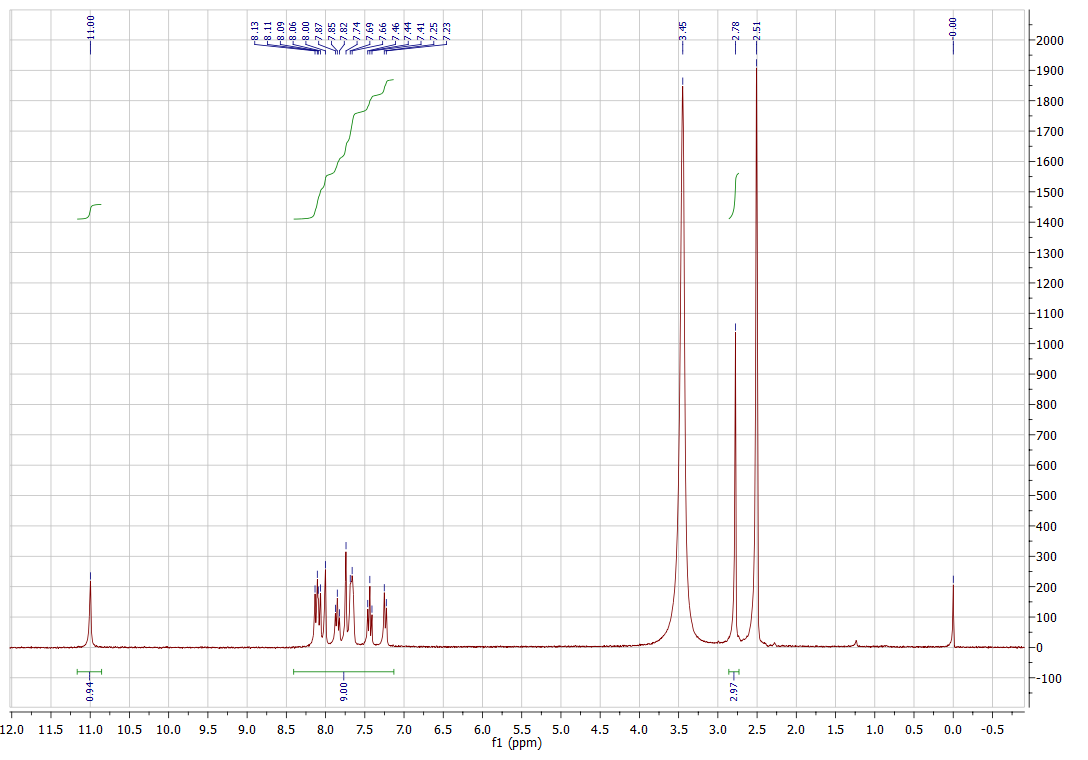


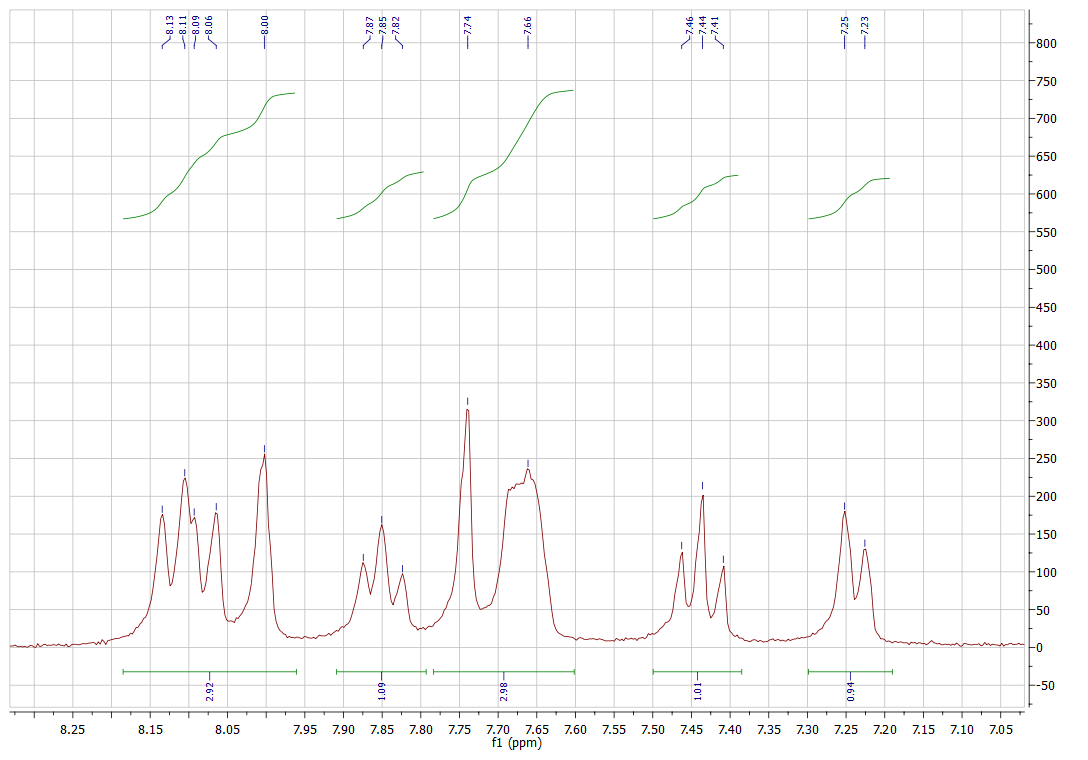


**Figure S14**. ^13^C APT NMR (DMSO-d_6_) of *N*-(3-Chlorophenyl)-2-methylquinoline-4-carboxamide (**3f**)


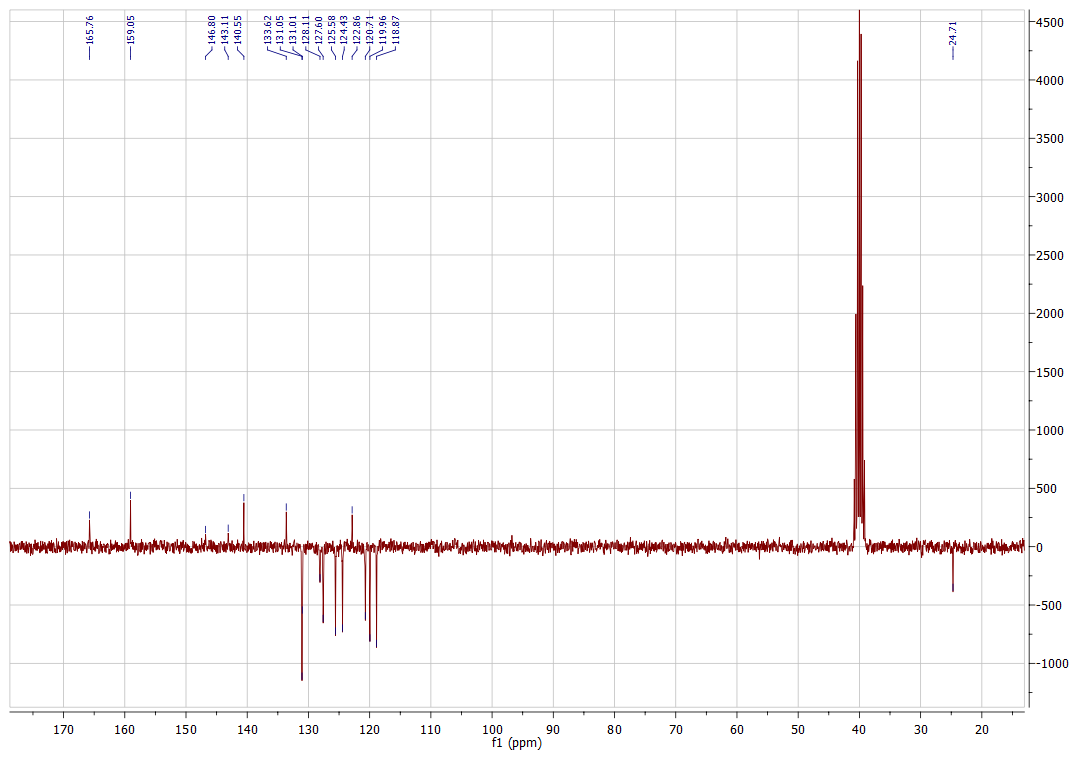


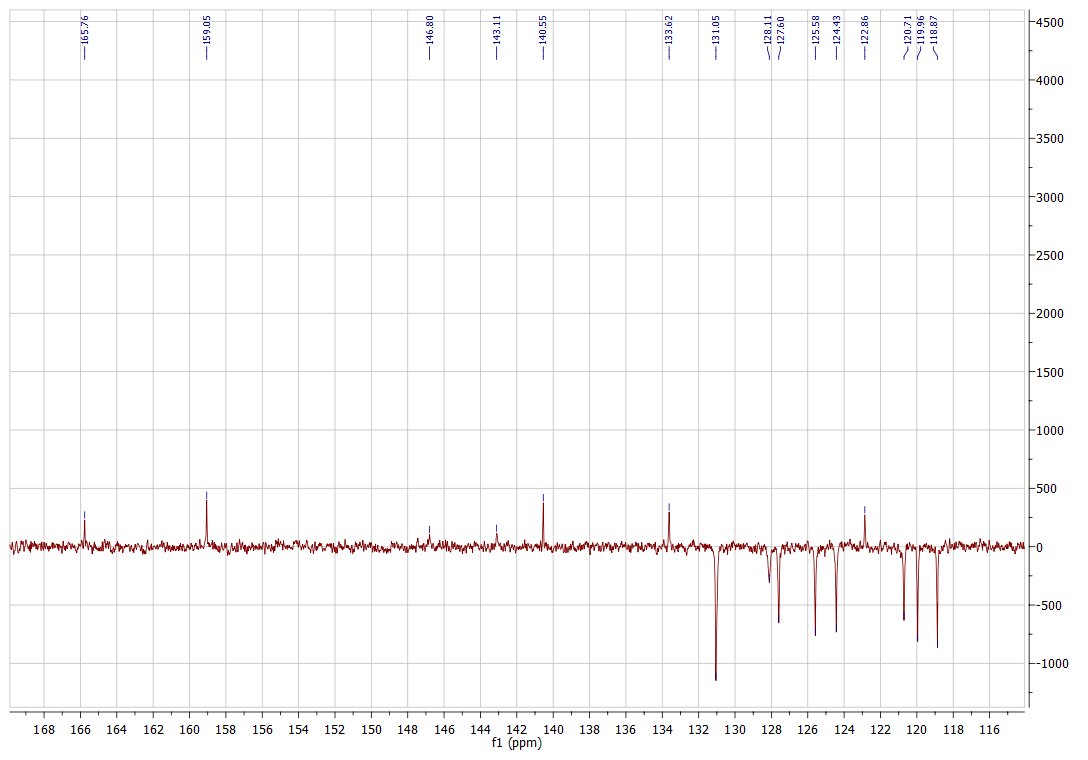


**Figure S15**. ^1^H NMR (DMSO-d_6_) of *N*-(4-Chlorophenyl)-2-methylquinoline-4-carboxamide (**3g**)


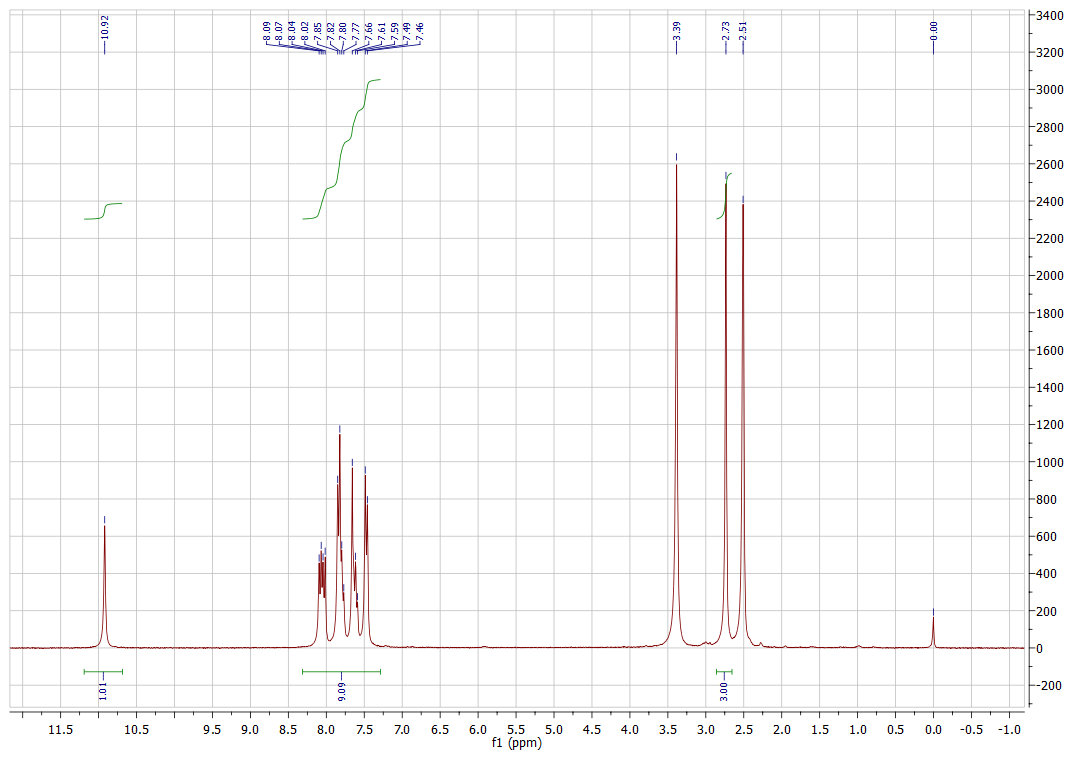


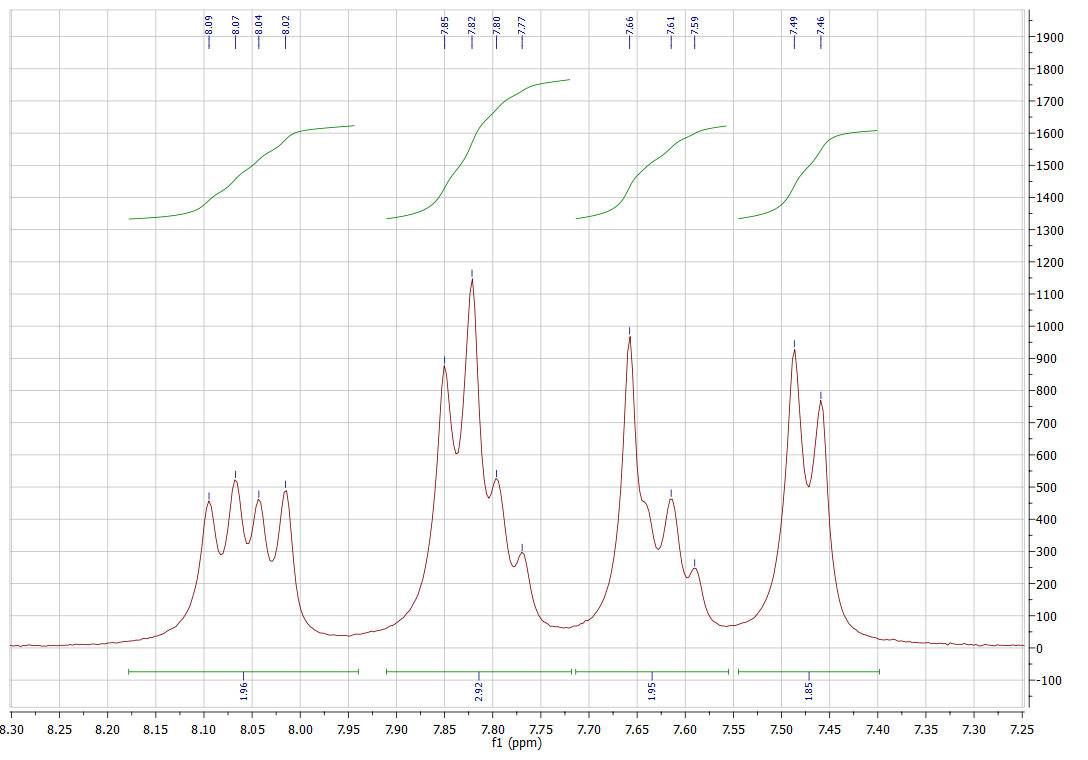


**Figure S16**. ^13^C APT NMR (DMSO-d_6_) of *N*-(4-Chlorophenyl)-2-methylquinoline-4-carboxamide (**3g**)


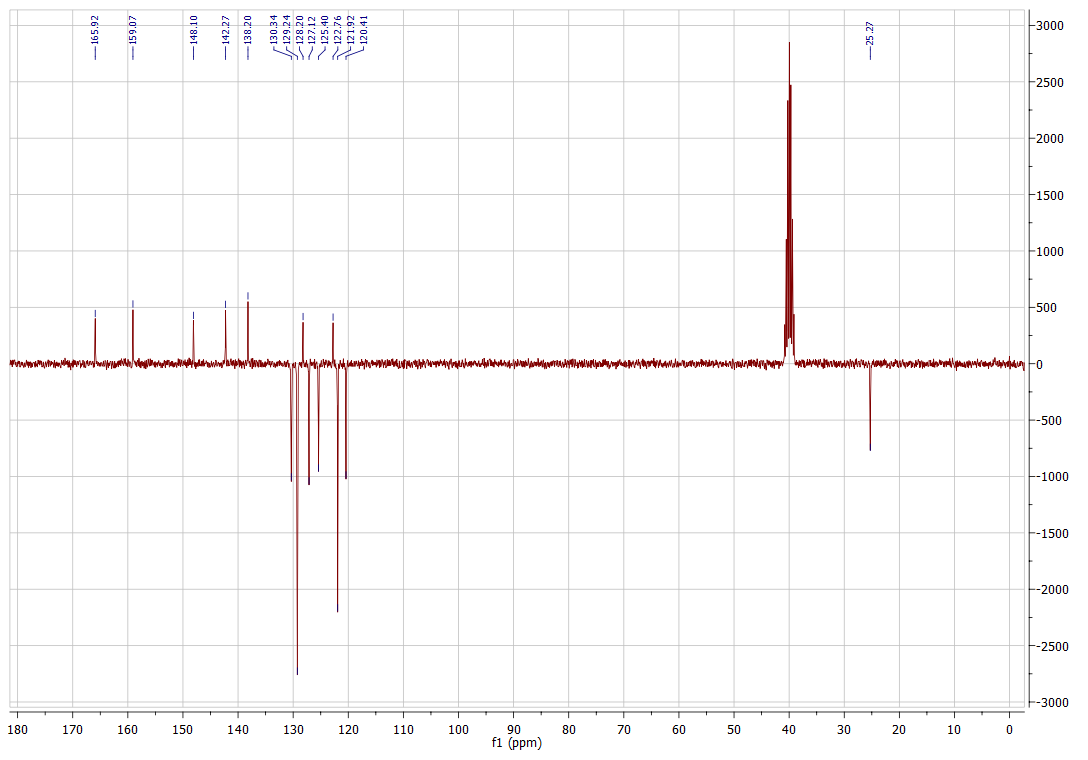


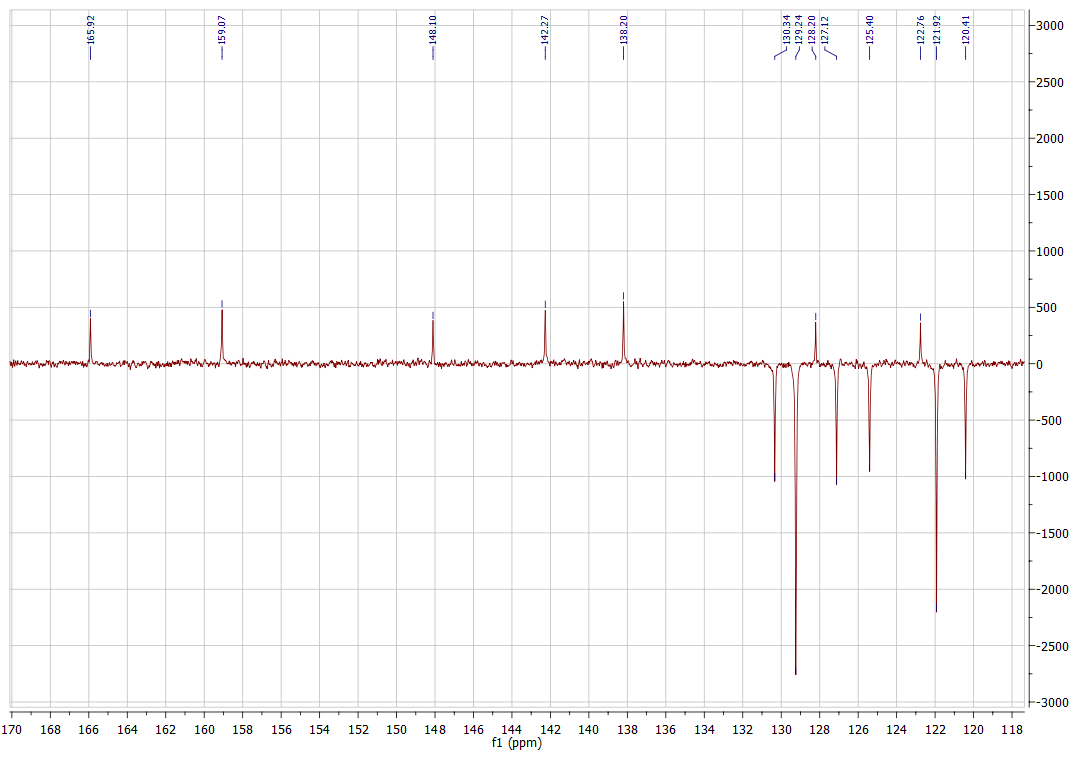


**Figure S17**. ^1^H NMR (DMSO-d_6_) of 2-Methyl-*N*-[4-(trifluoromethyl)phenyl]quinoline-4-carboxamide (**3h**)


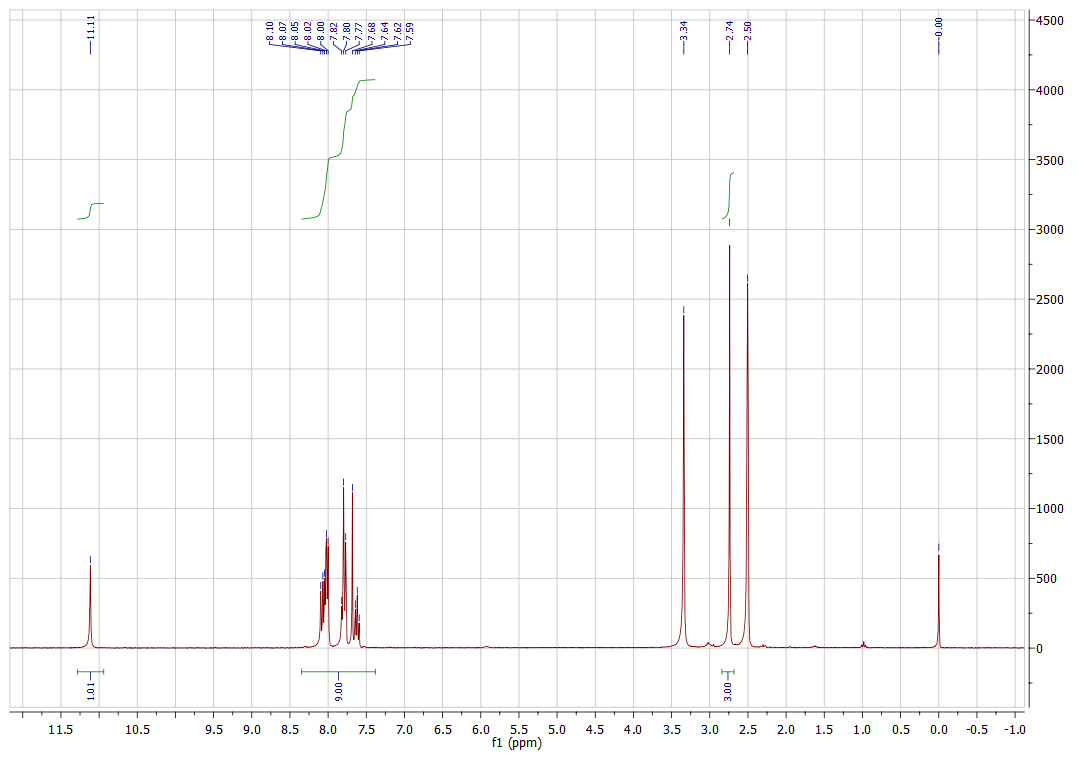


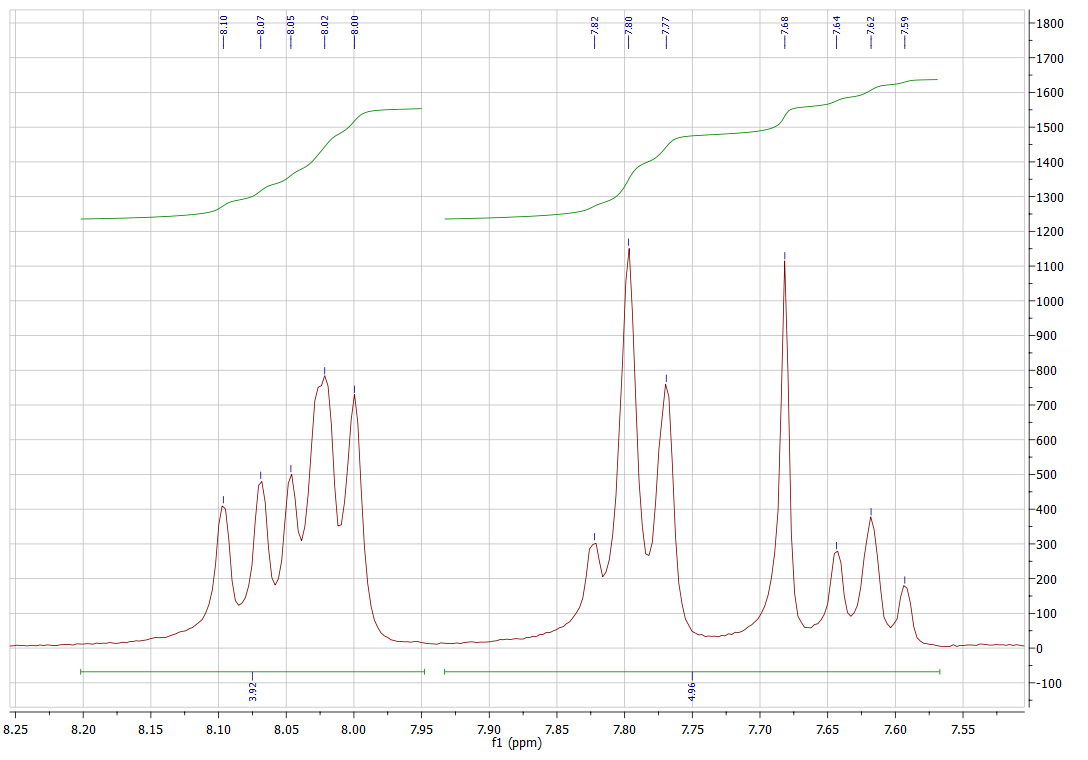


**Figure S18**. ^13^C APT NMR (DMSO-d_6_) of 2-Methyl-*N*-[4-(trifluoromethyl)phenyl]quinoline-4-carboxamide (**3h**)


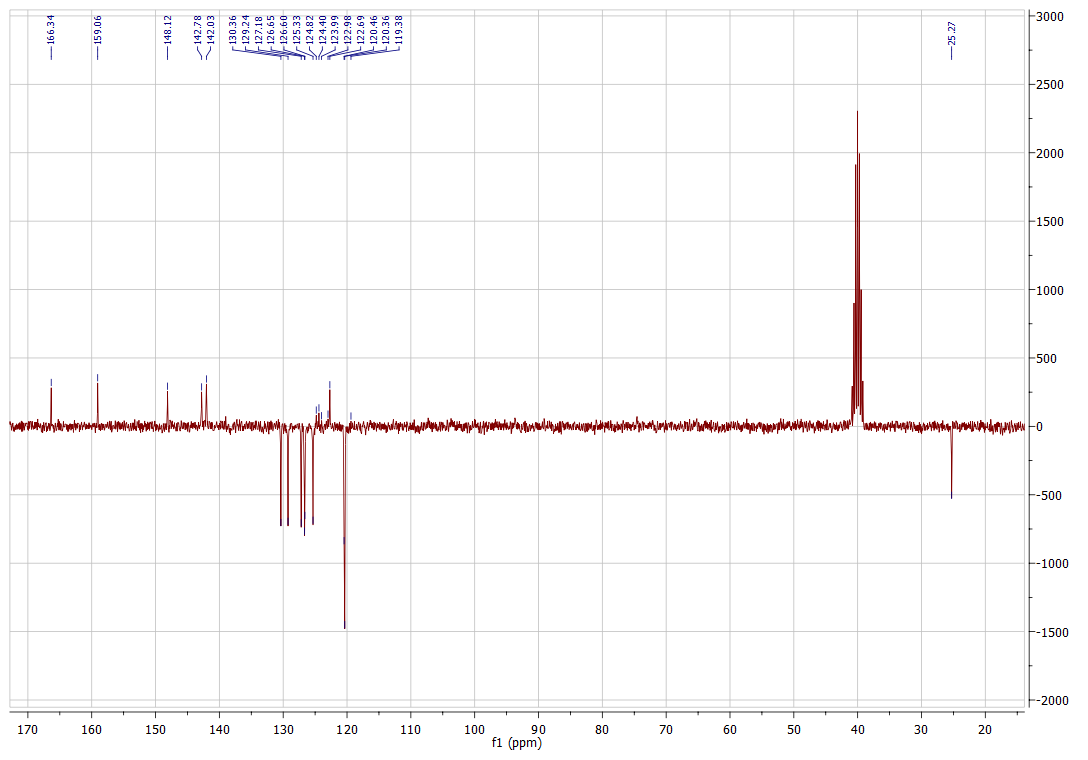


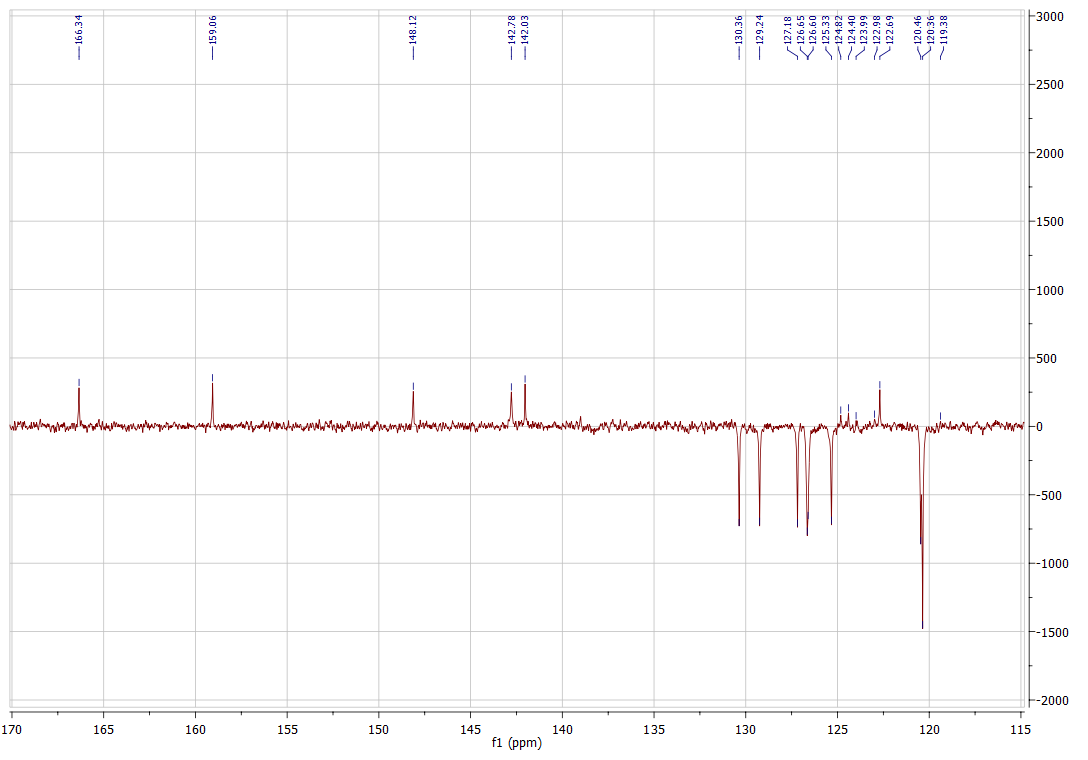


**Figure S19**. ^1^H NMR (DMSO-d_6_) of 2-Methyl-*N*-(4-nitrophenyl)quinoline-4-carboxamide (**3i**)


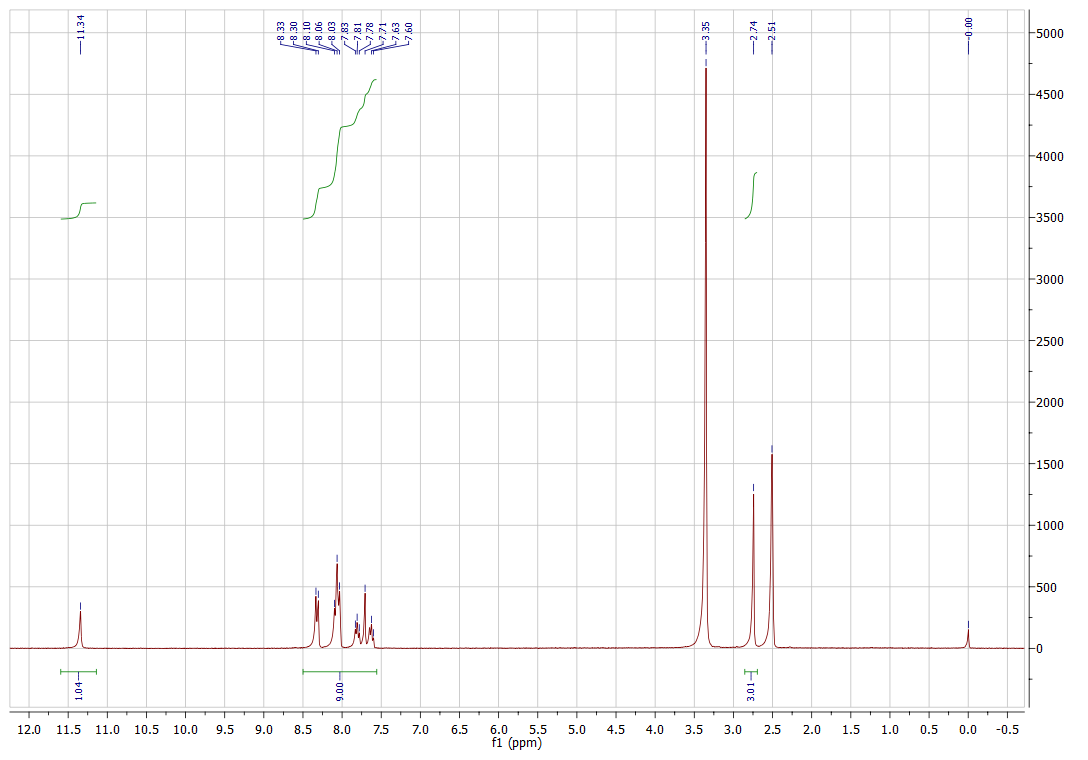


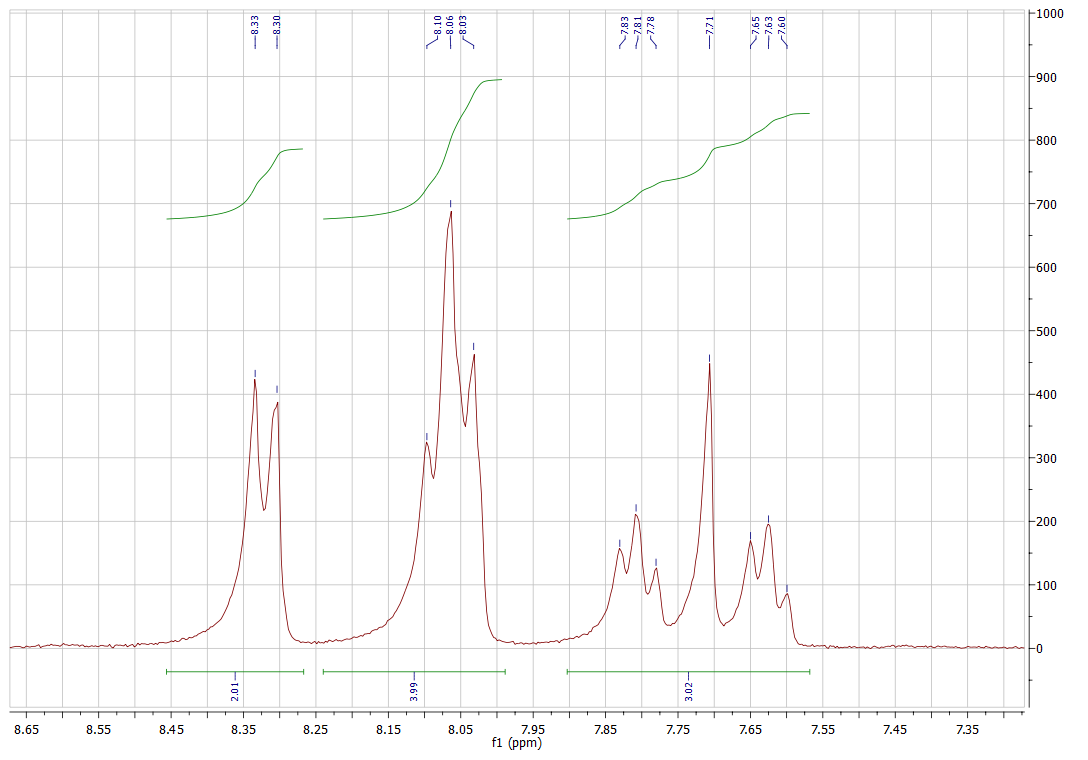


**Figure S20**. ^13^C APT NMR (DMSO-d_6_) of 2-Methyl-*N*-(4-nitrophenyl)quinoline-4-carboxamide (**3i**)


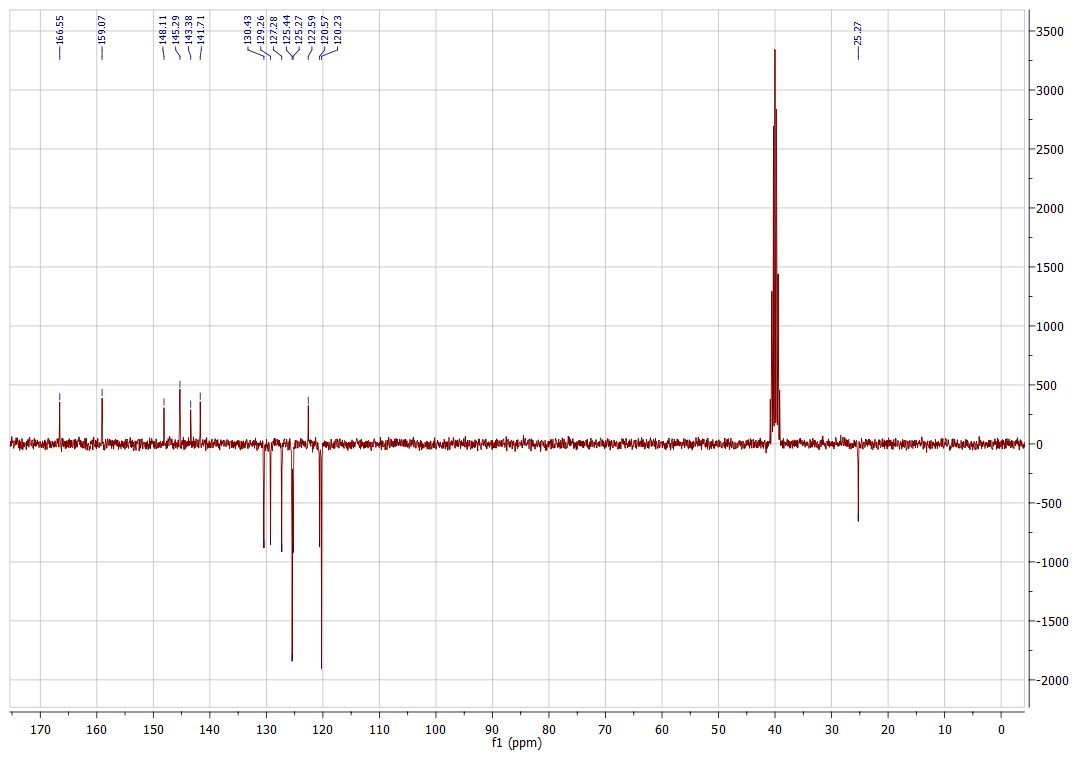


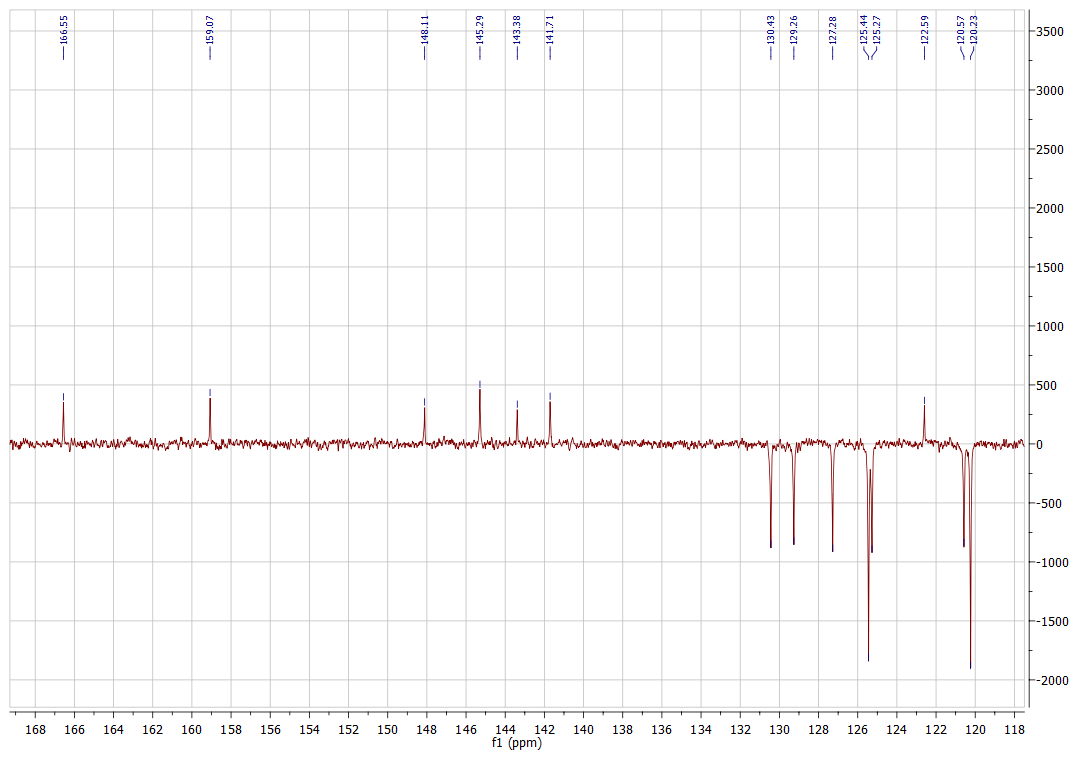


**Figure S21**. ^1^H NMR (DMSO-d_6_) of 2-Methyl-*N*-[4-nitro-3-(trifluoromethyl)phenyl]quinoline-4-carboxamide (**3j**)


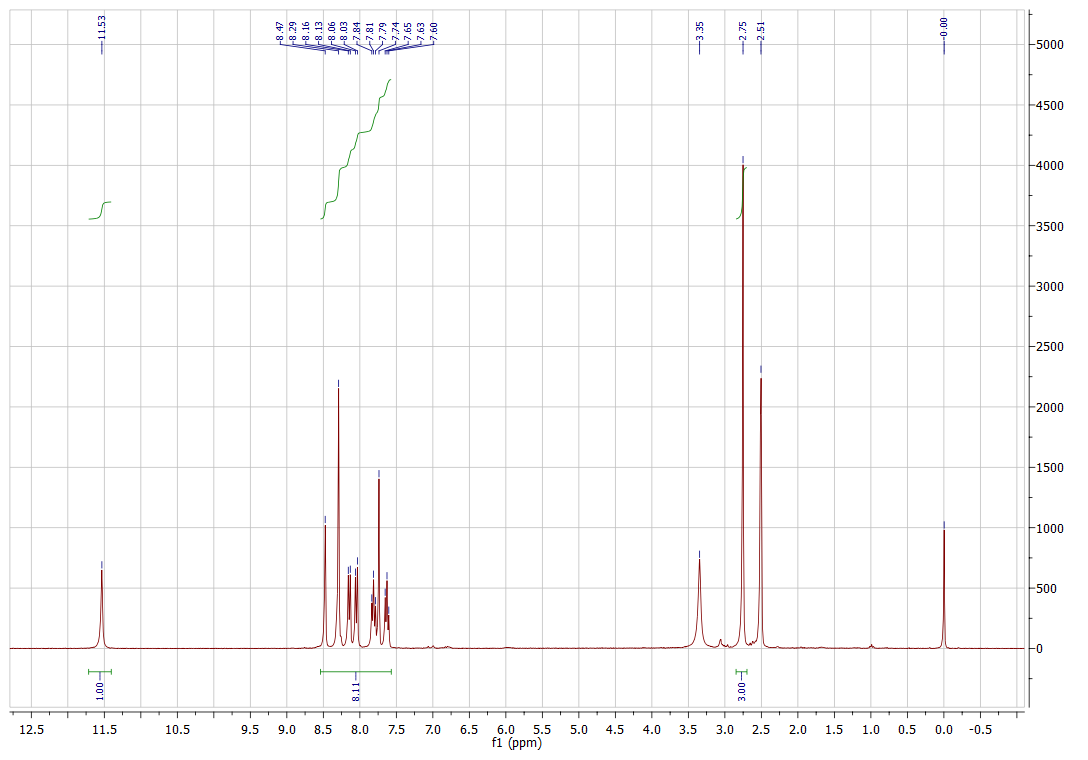


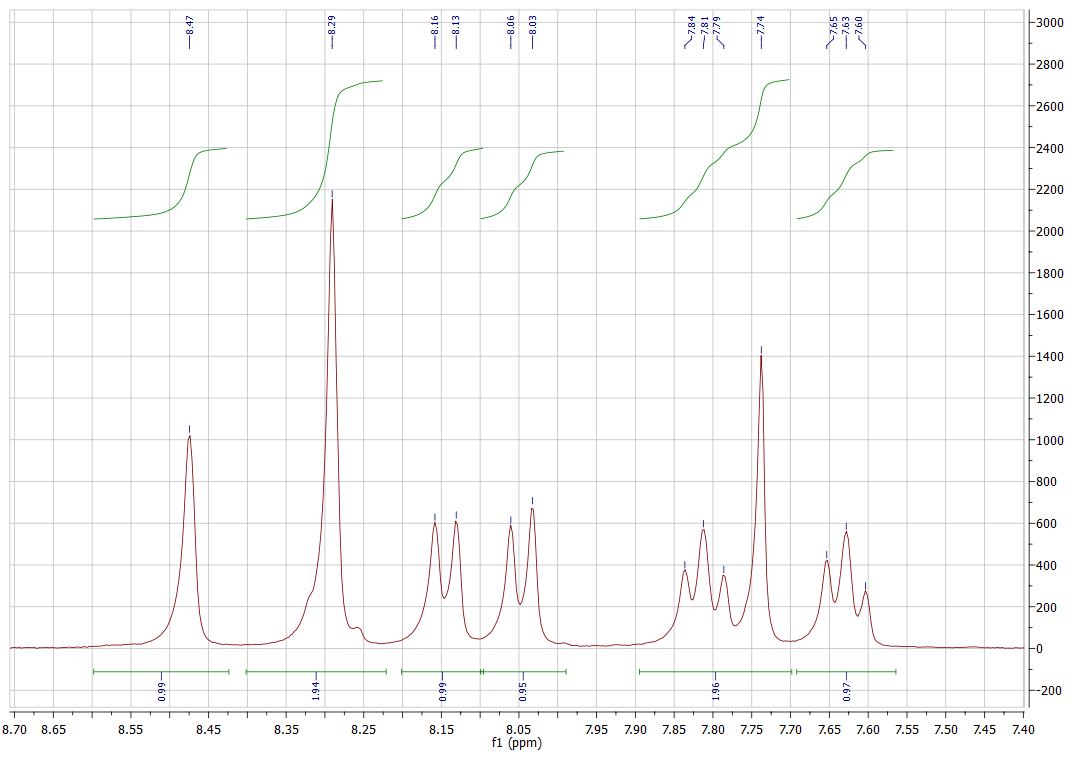


**Figure S22**. ^13^C APT NMR (DMSO-d_6_) of 2-Methyl-*N*-[4-nitro-3-(trifluoromethyl)phenyl]quinoline-4-carboxamide (**3j**)


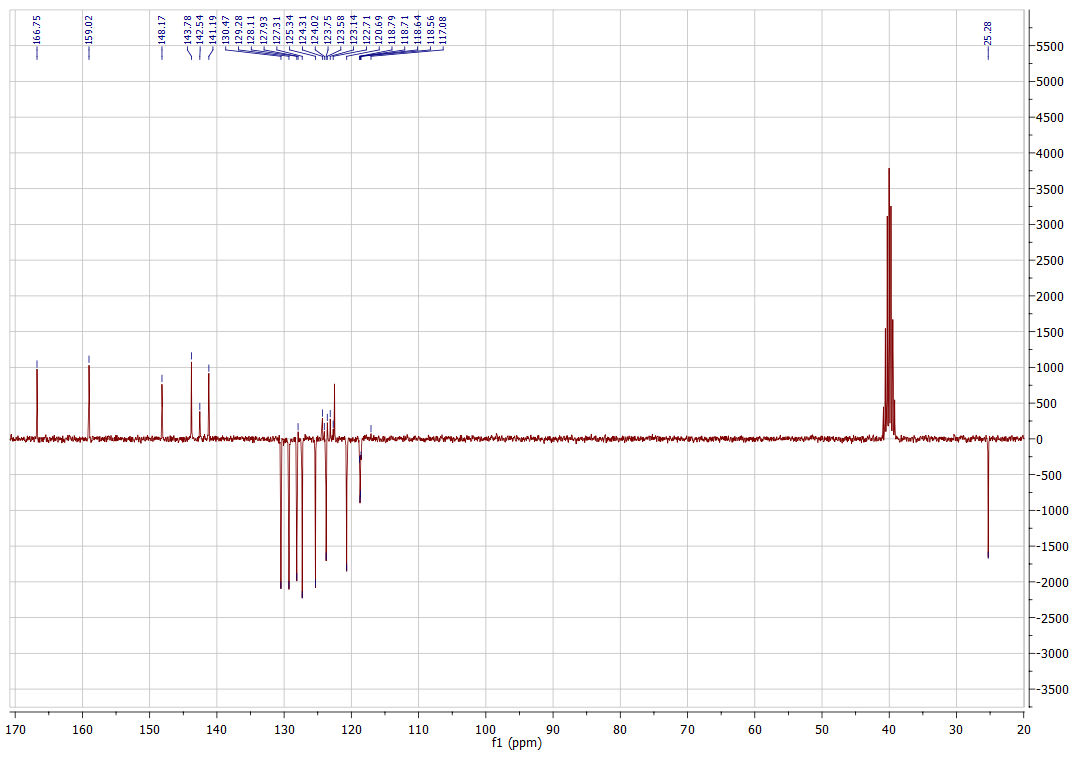


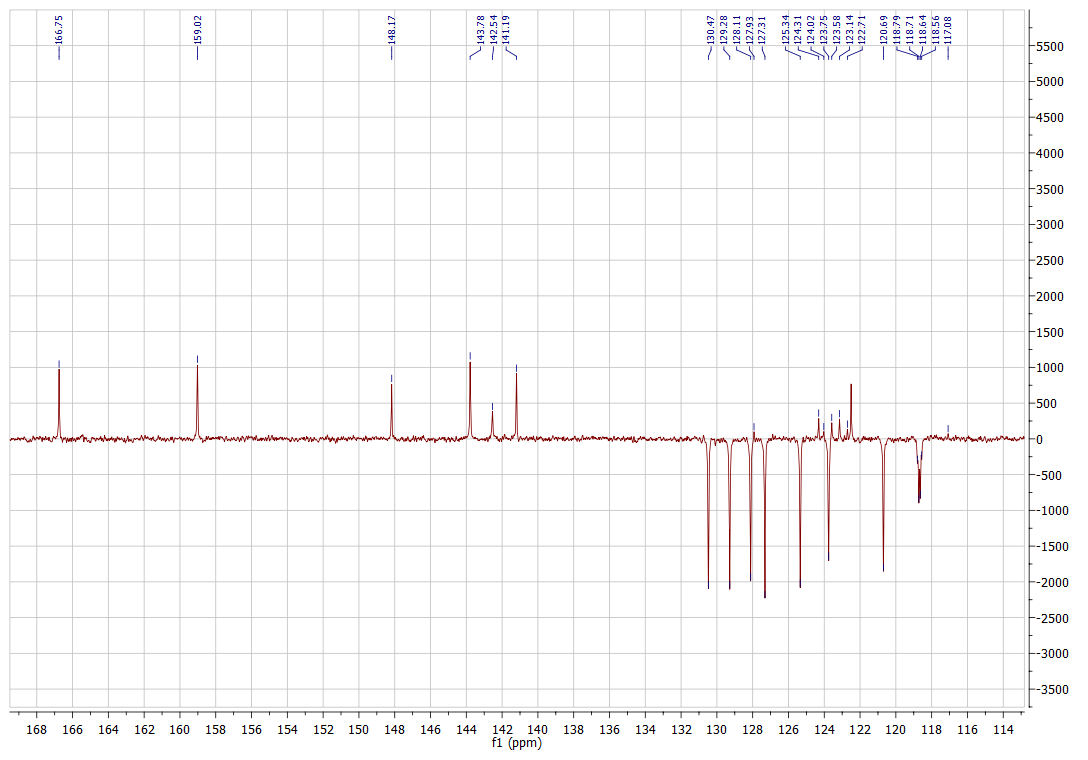


**Figure S23**. ^1^H NMR (DMSO-d_6_) of *(E)-N*-phenyl-2-styrylquinoline-4-carboxamide (**4a**)


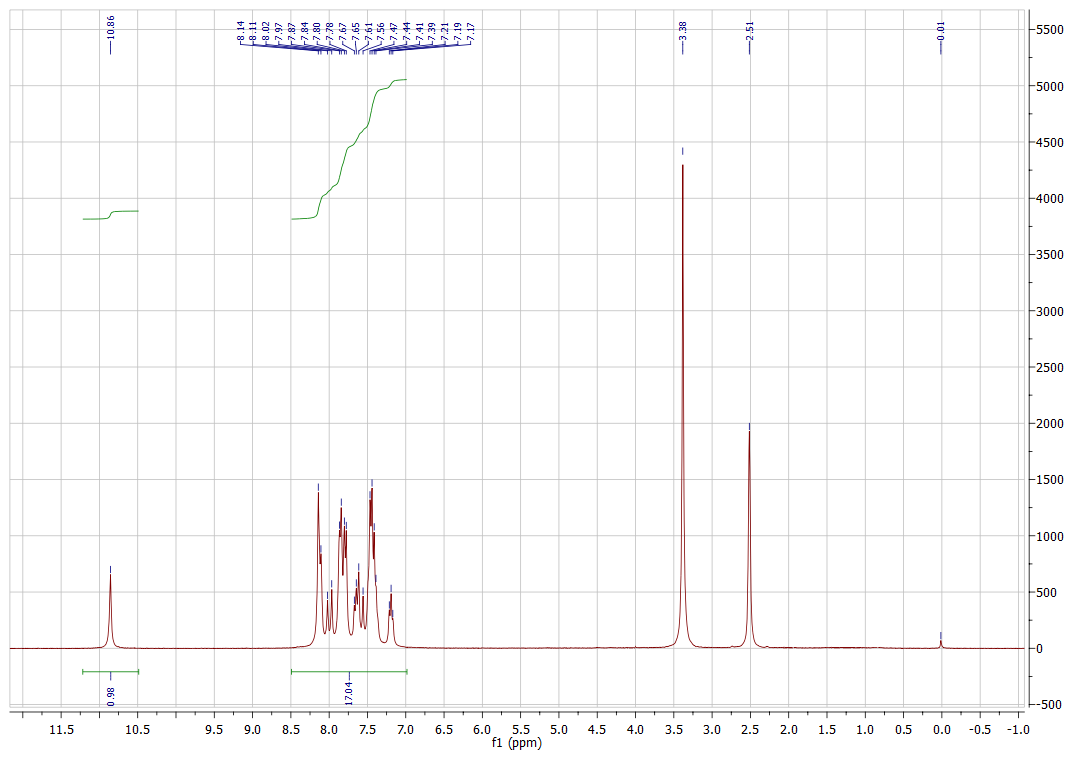


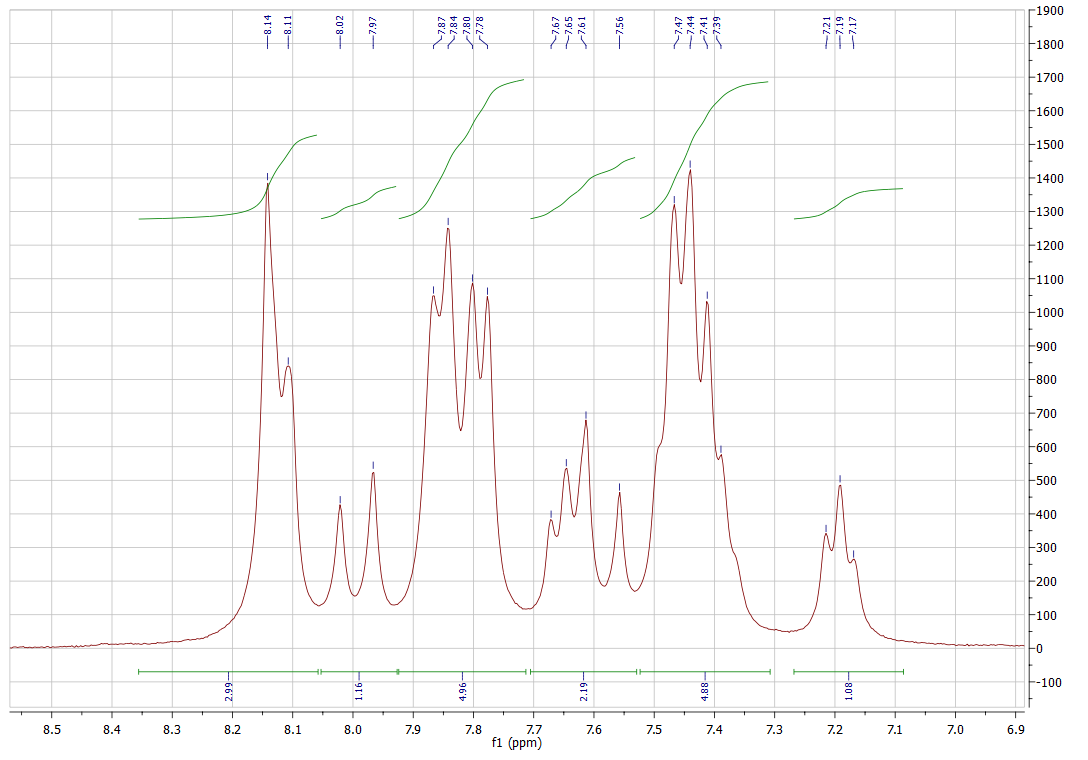


**Figure S24**. ^13^C APT NMR (DMSO-d_6_) of *(E)-N*-phenyl-2-styrylquinoline-4-carboxamide (**4a**)


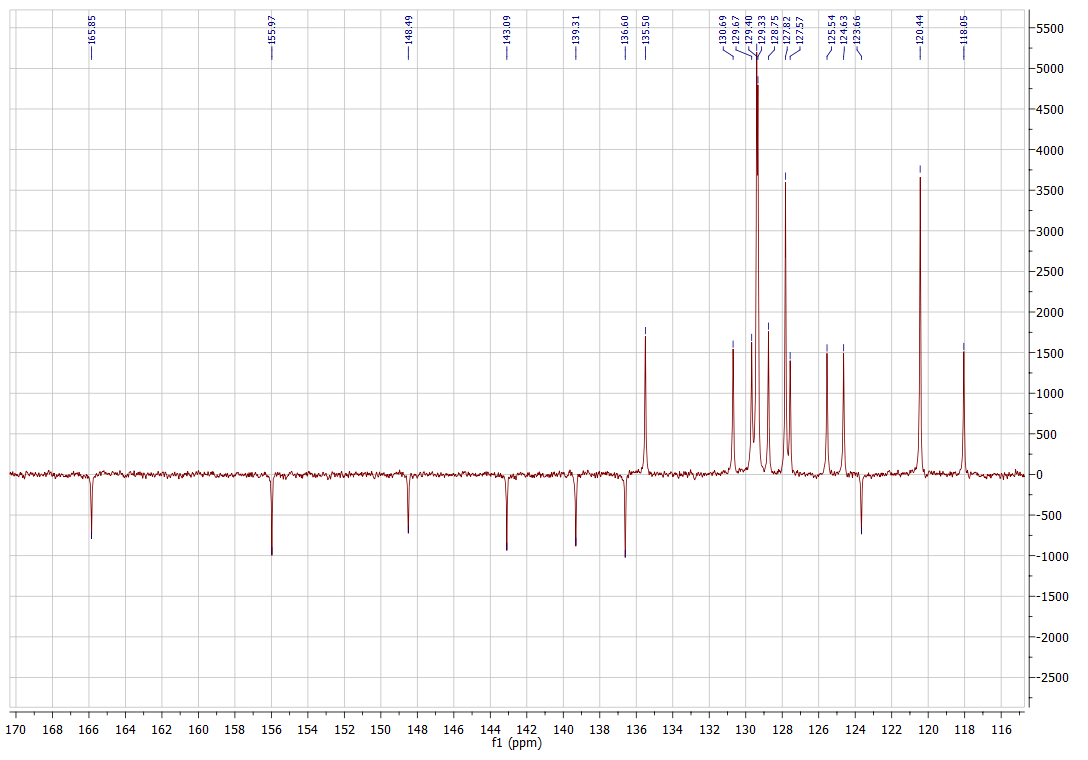


**Figure S25**. HPLC chromatogram and mass spectra of *(E)-N*-phenyl-2-styrylquinoline-4-carboxamide (**4a**)

| **Peak** | **RT** | **Area Sum %** | **Area** |
| --- | --- | --- | --- |
| 1 | 8.1743 | 0.17 | 1.99 |
| 2 | 8.4376 | 99.58 | 1151.82 |
| 3 | 8.9043 | 0.14 | 1.67 |
| 4 | 9.3843 | 0.05 | 0.56 |
| 5 | 9.6143 | 0.05 | 0.61 |

ESI-MS analysis for [C_24_H_18_N_2_O+H^+^]: Calc.: 351.1492 *m*/*z*, exp.: 351.1494 *m*/*z*.

**Figure S26**. ^1^H NMR (DMSO-d_6_) of *(E)-*2-styryl-*N*-(m-tolyl)quinoline-4-carboxamide (**4b**)


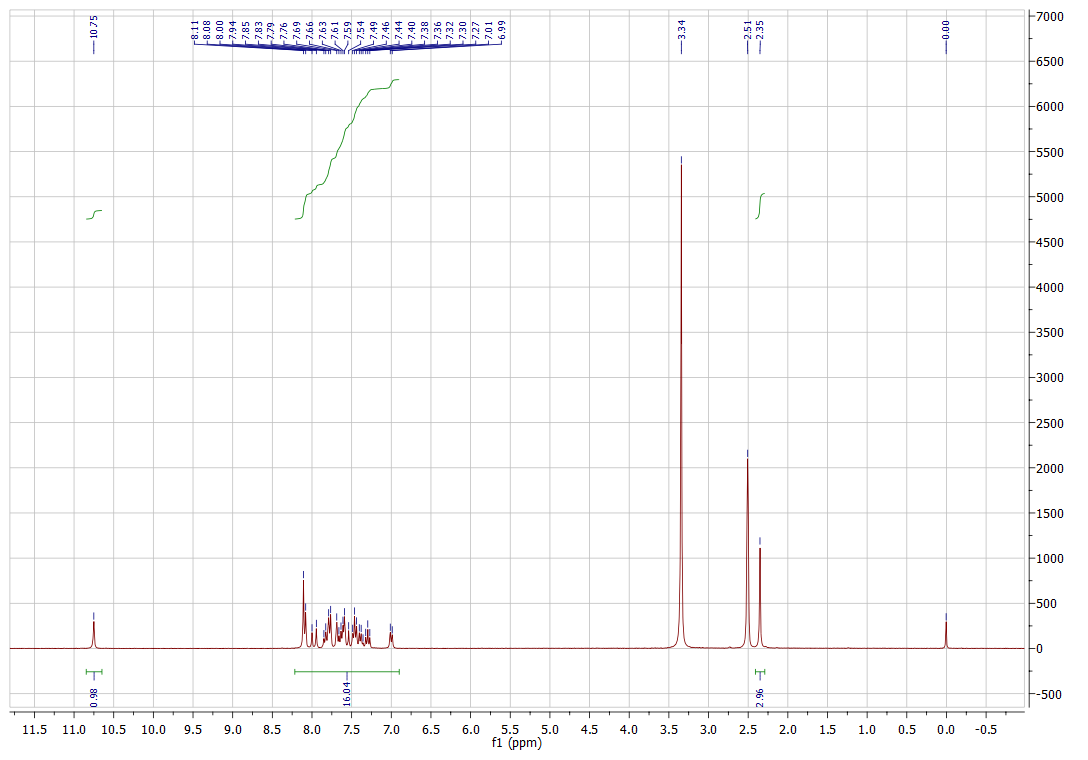


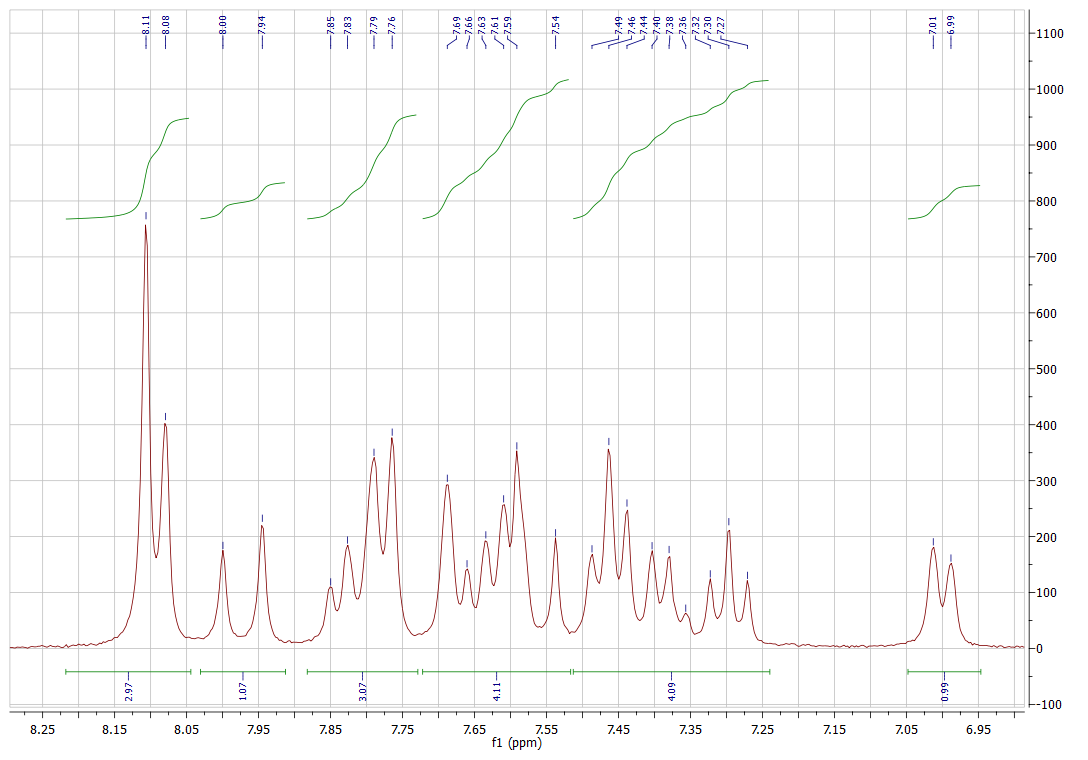


**Figure S27**. ^13^C APT NMR (DMSO-d_6_) of *(E)-*2-styryl-*N*-(m-tolyl)quinoline-4-carboxamide (**4b**)


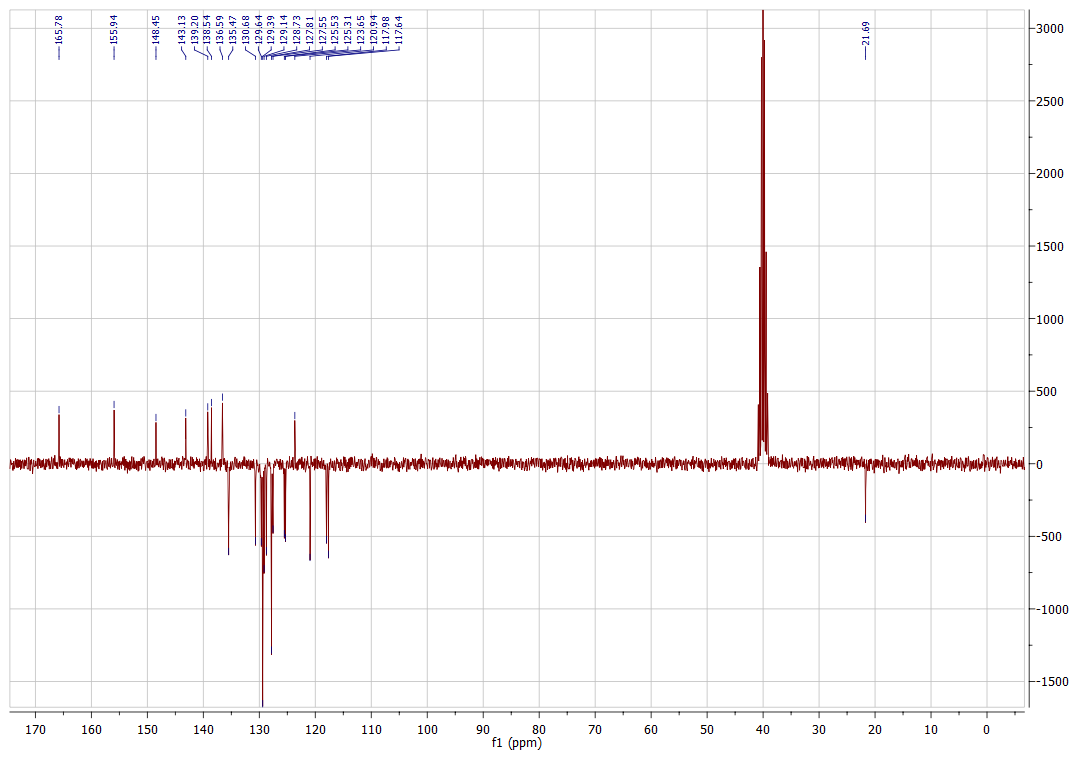


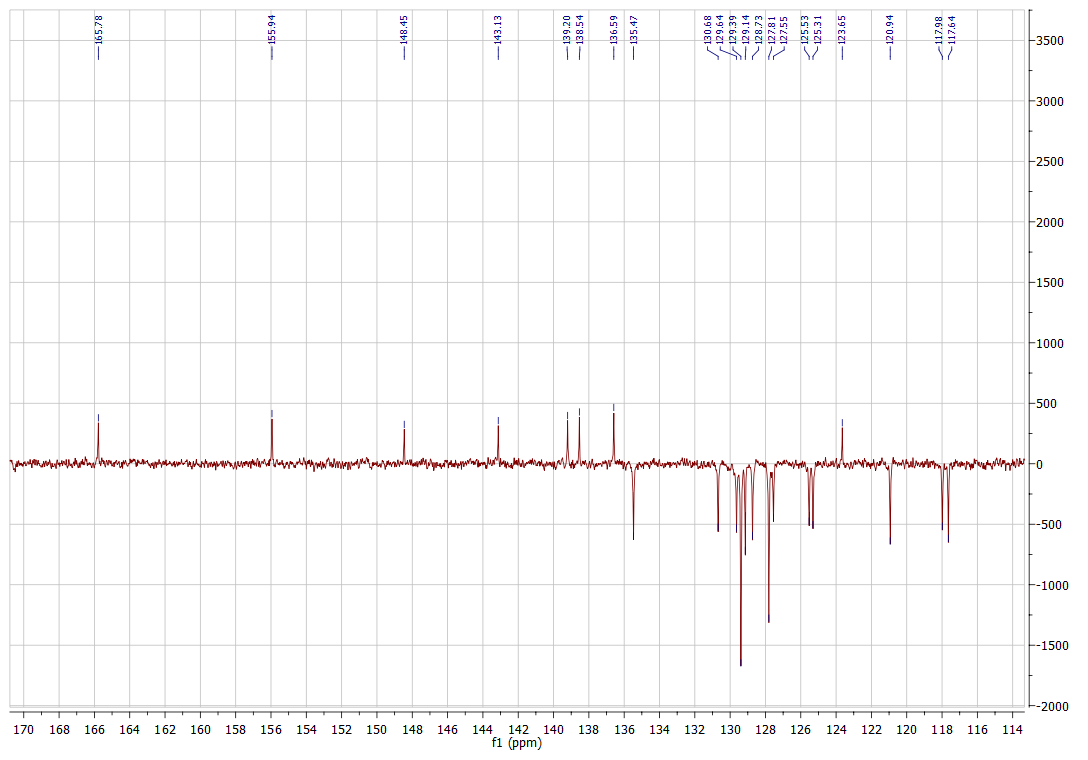


**Figure S28**. HPLC chromatogram and mass spectra of *(E)-*2-styryl-*N*-(m-tolyl)quinoline-4-carboxamide (**4b**)

| **Peak** | **RT** | **Area Sum %** | **Area** |
| --- | --- | --- | --- |
| 1 | 6.6945 | 0.69 | 4.27 |
| 2 | 7.2045 | 0.29 | 1.77 |
| 3 | 8.8678 | 98.21 | 607.65 |
| 4 | 9.5878 | 0.81 | 5.03 |

ESI-MS analysis for [C_25_H_20_N_2_O+Na^+^]: Calc.: 387.146 *m*/*z*, exp.: 387.1467 *m*/*z.*

**Figure S29**. ^1^H NMR (DMSO-d_6_) of *(E)-*2-styryl-*N*-(p-tolyl)quinoline-4-carboxamide (**4c**)


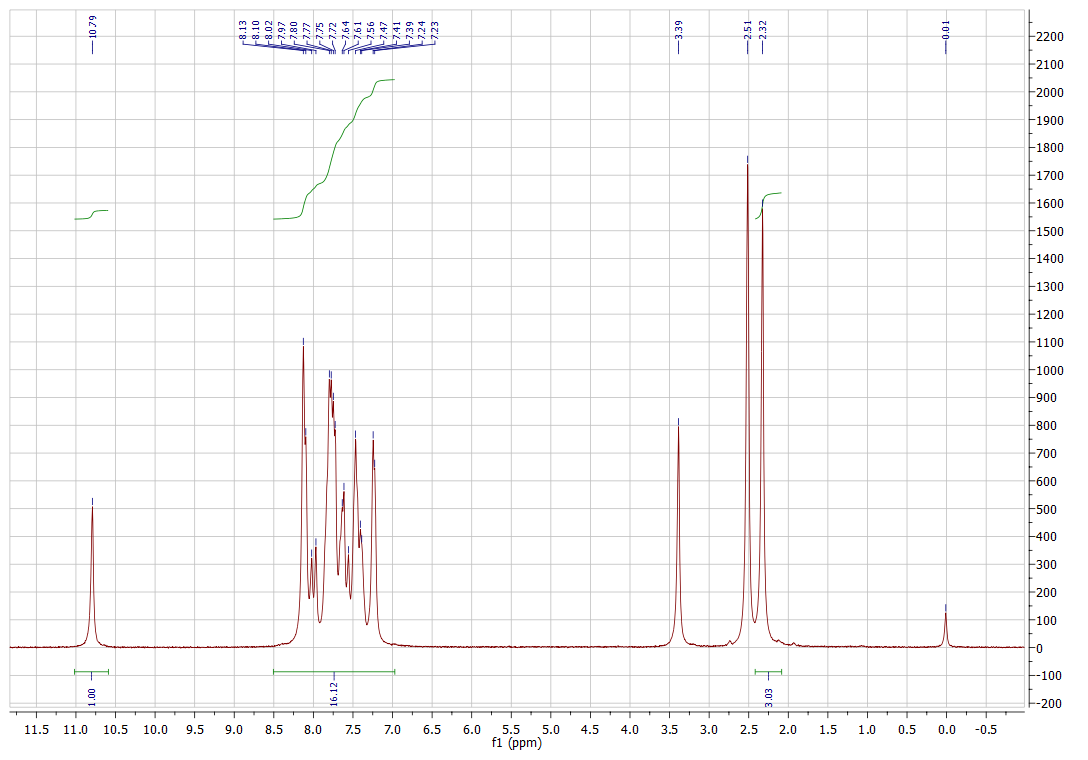


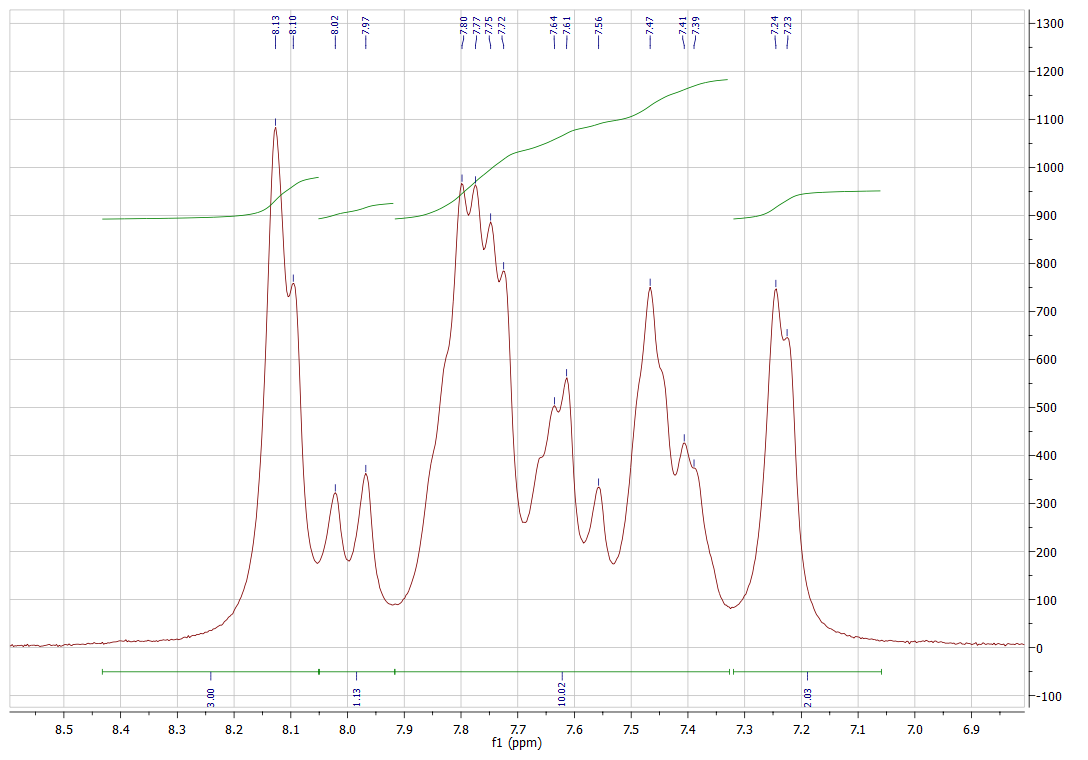


**Figure S30**. ^13^C APT NMR (DMSO-d_6_) of *(E)-*2-styryl-*N*-(p-tolyl)quinoline-4-carboxamide (**4c**)


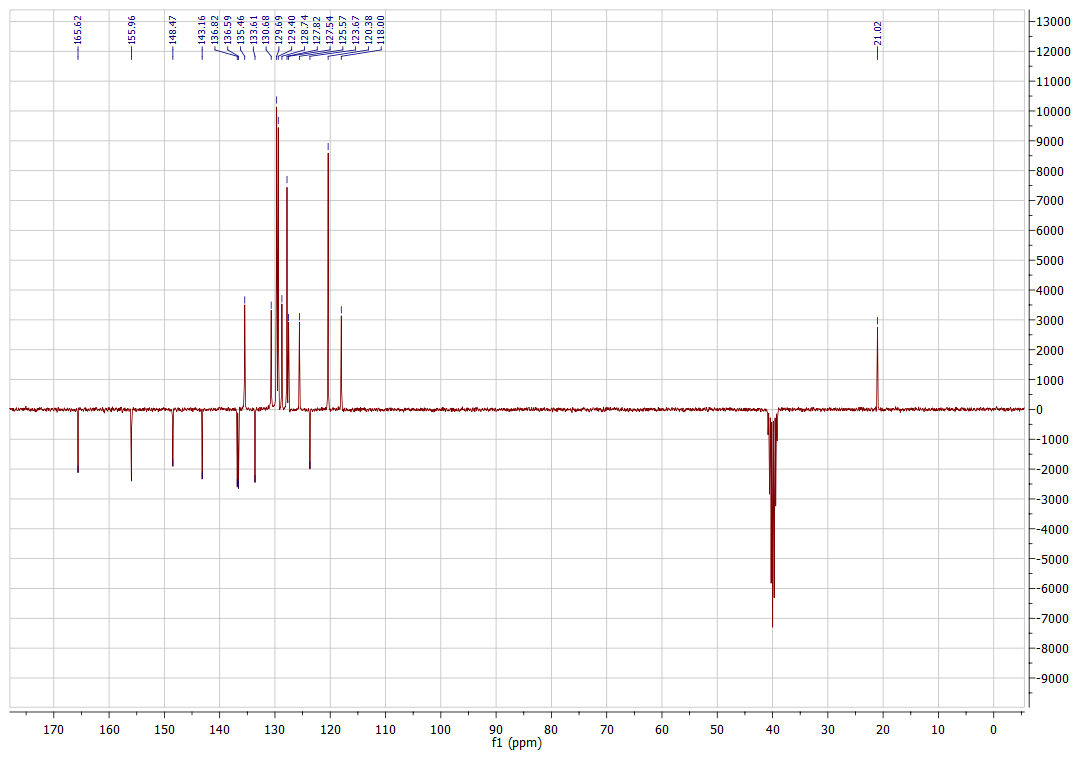


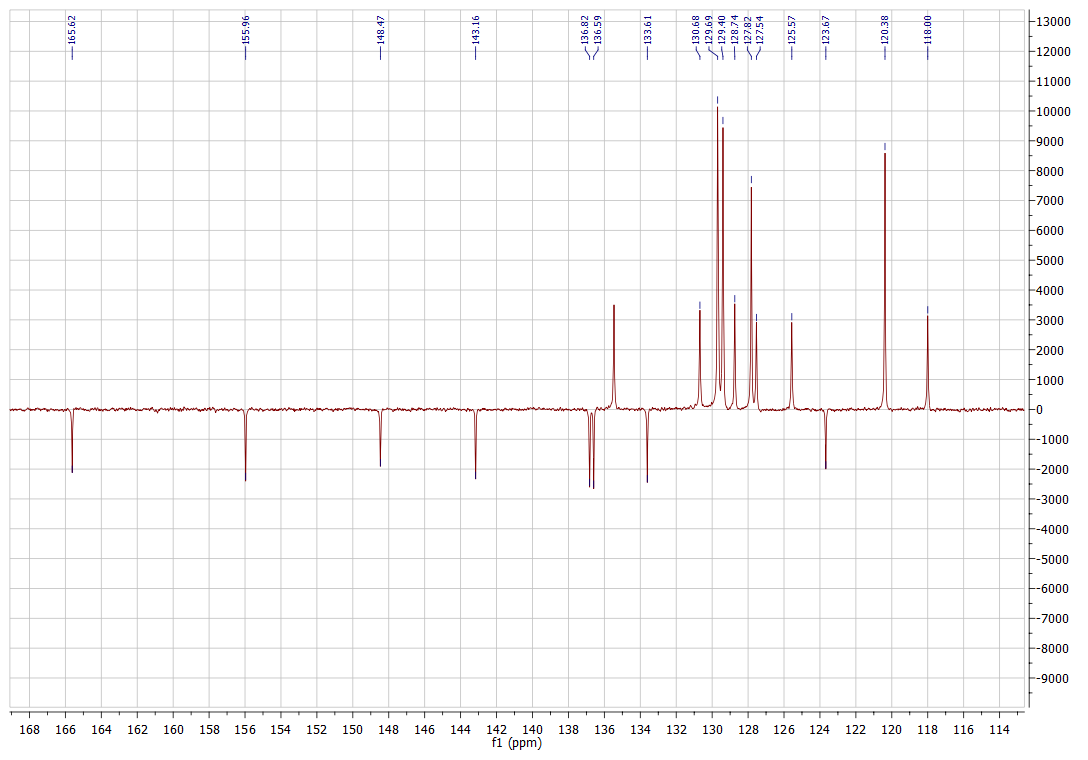


**Figure S31**. HPLC chromatogram and mass spectra of *(E)-*2-styryl-*N*-(p-tolyl)quinoline-4-carboxamide (**4c**)

| **Peak** | **RT** | **Area Sum %** | **Area** |
| --- | --- | --- | --- |
| 1 | 7.4480 | 0.31 | 8.08 |
| 2 | 8.2280 | 0.1 | 2.45 |
| 3 | 8.8380 | 99.17 | 2542.78 |
| 4 | 9.5347 | 0.42 | 10.69 |

ESI-MS analysis for [C_25_H_20_N_2_O+H^+^]: Calc.: 365.1648 *m*/*z*, exp.: 365.1647 *m*/*z.*

**Figure S32**. ^1^H NMR (DMSO-d_6_) of *(E)-N*-(2,6-dimethylphenyl)-2-styrylquinoline-4-carboxamide (**4d**)


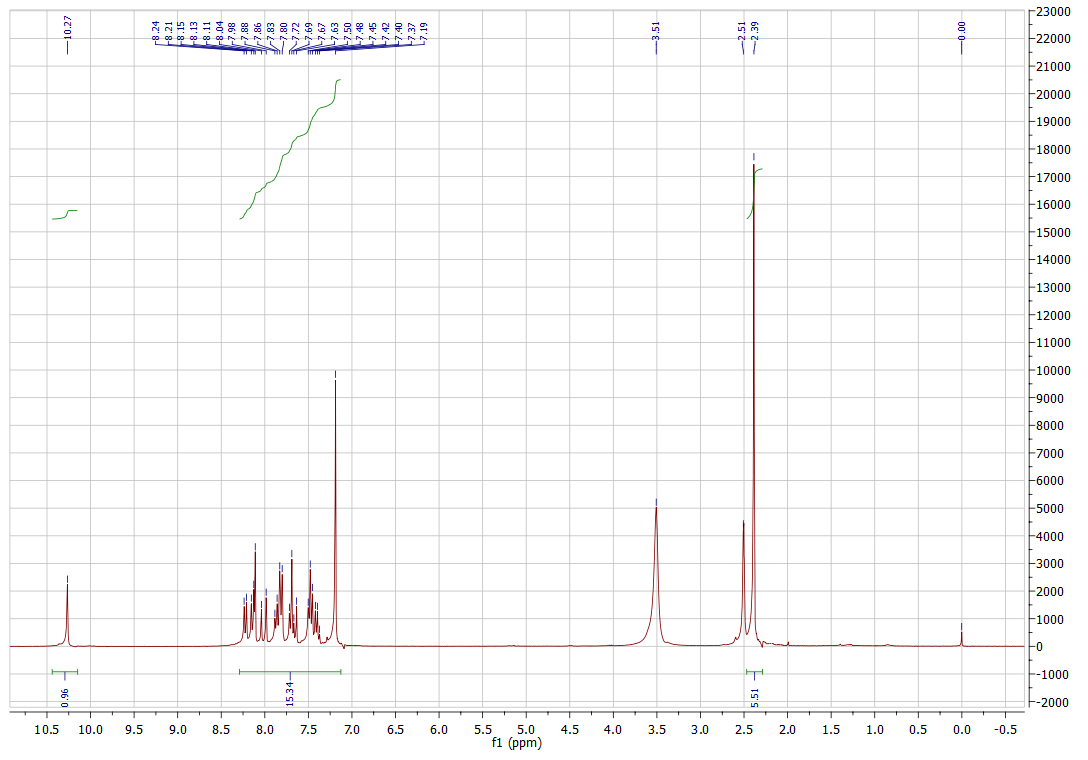


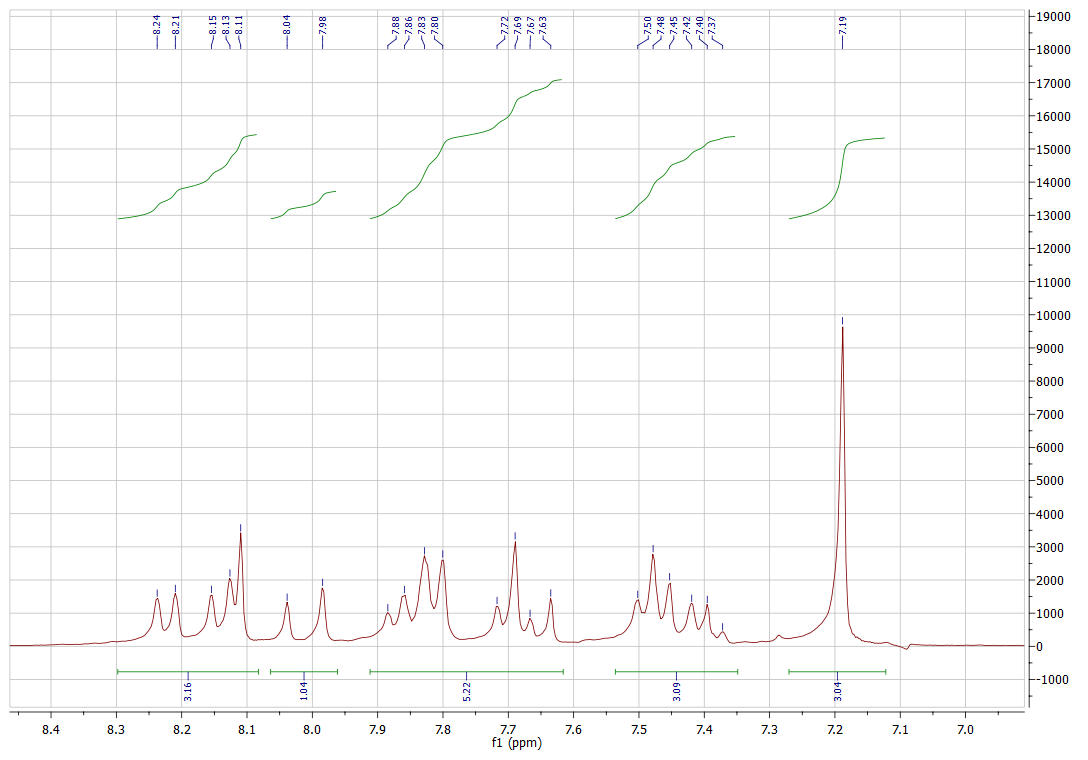


**Figure S33**. ^13^C APT NMR (DMSO-d_6_) of *(E)-N*-(2,6-dimethylphenyl)-2-styrylquinoline-4-carboxamide (**4d**)


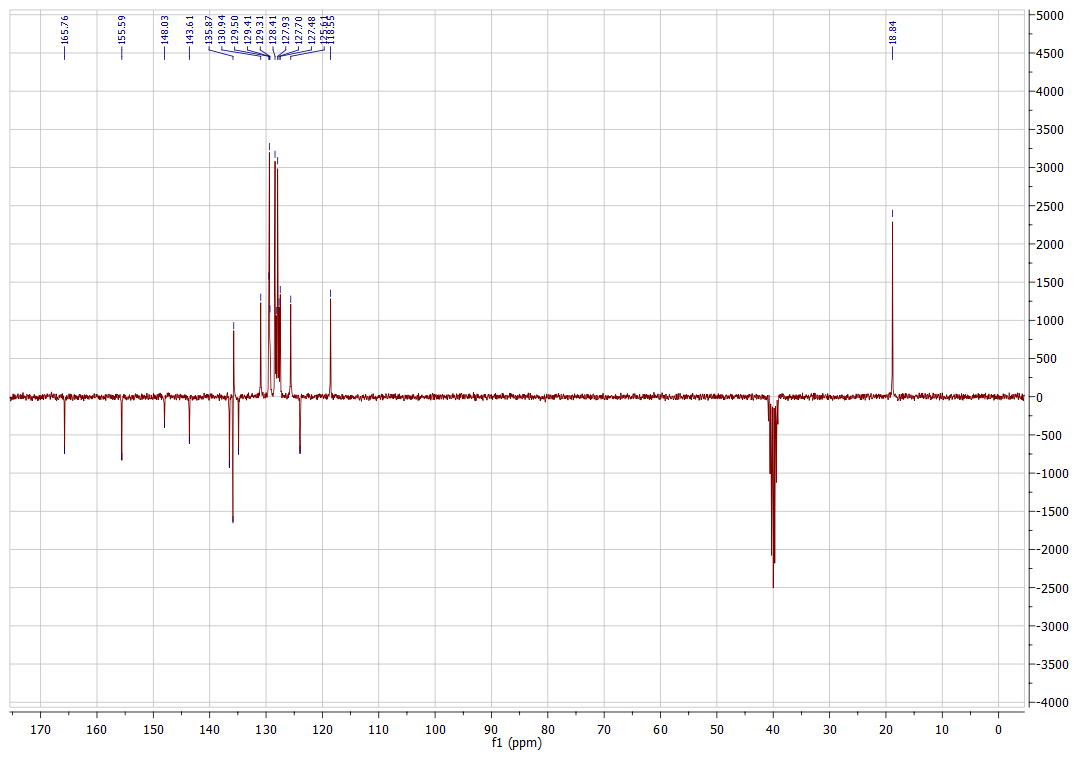


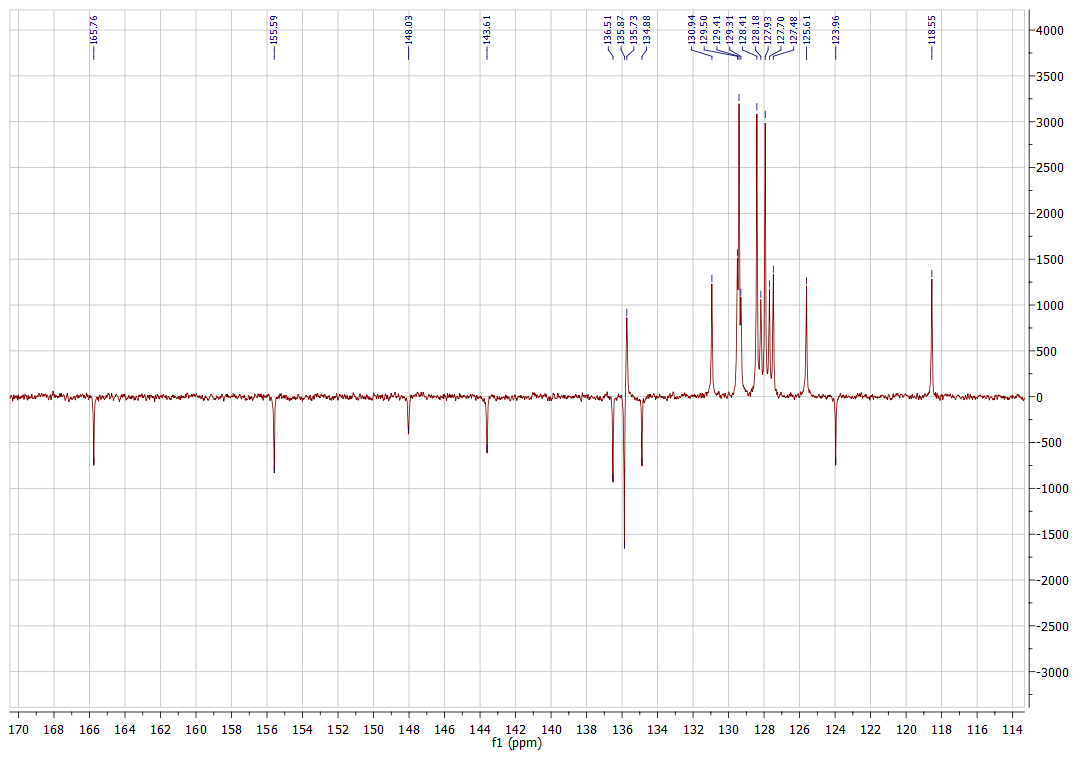


**Figure S34**. HPLC chromatogram and mass spectra of *(E)-N*-(2,6-dimethylphenyl)-2-styrylquinoline-4-carboxamide (**4d**)

| **Peak** | **RT** | **Area Sum %** | **Area** |
| --- | --- | --- | --- |
| 1 | 8.2316 | 1.18 | 3.2 |
| 2 | 8.5882 | 98.35 | 267.64 |
| 3 | 8.8516 | 0.47 | 1.29 |

ESI-MS analysis for [C_26_H_22_N_2_O+H^+^]: Calc.: 379.1805 *m*/*z*, exp.: 379.1805 *m*/*z.*

**Figure S35**. ^1^H NMR (DMSO-d_6_) of *(E)-*2-styryl-*N*-(3,4,5-trimethoxyphenyl)quinoline-4-carboxamide (**4e**)


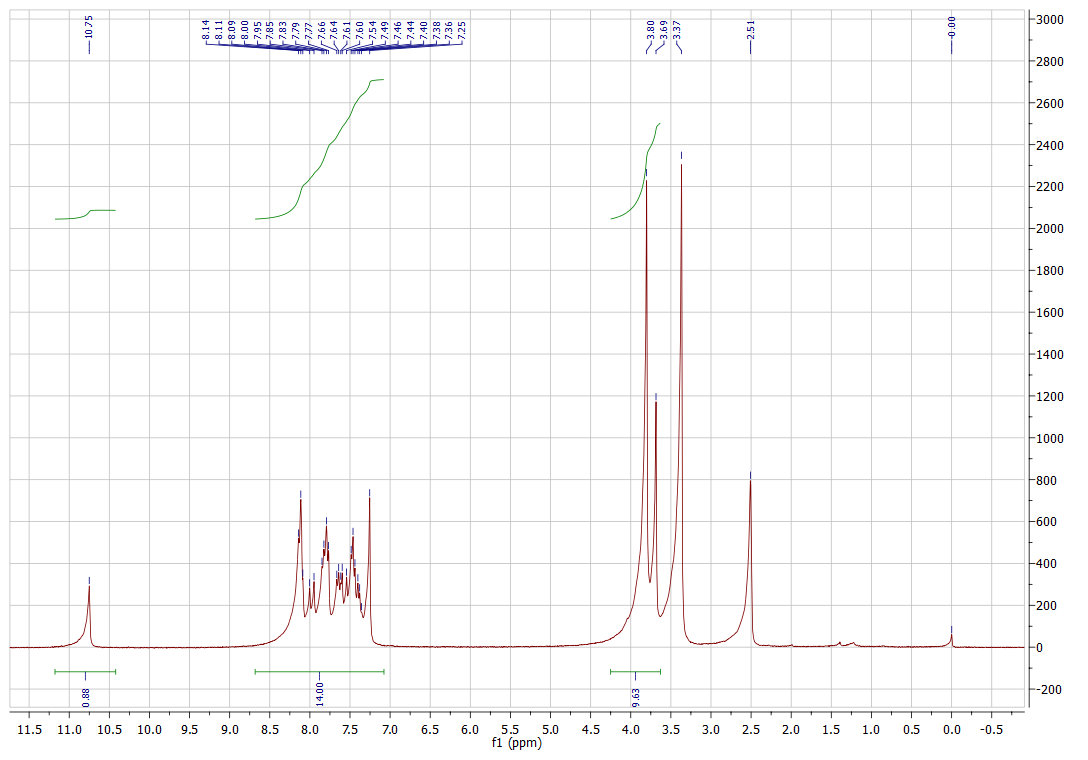


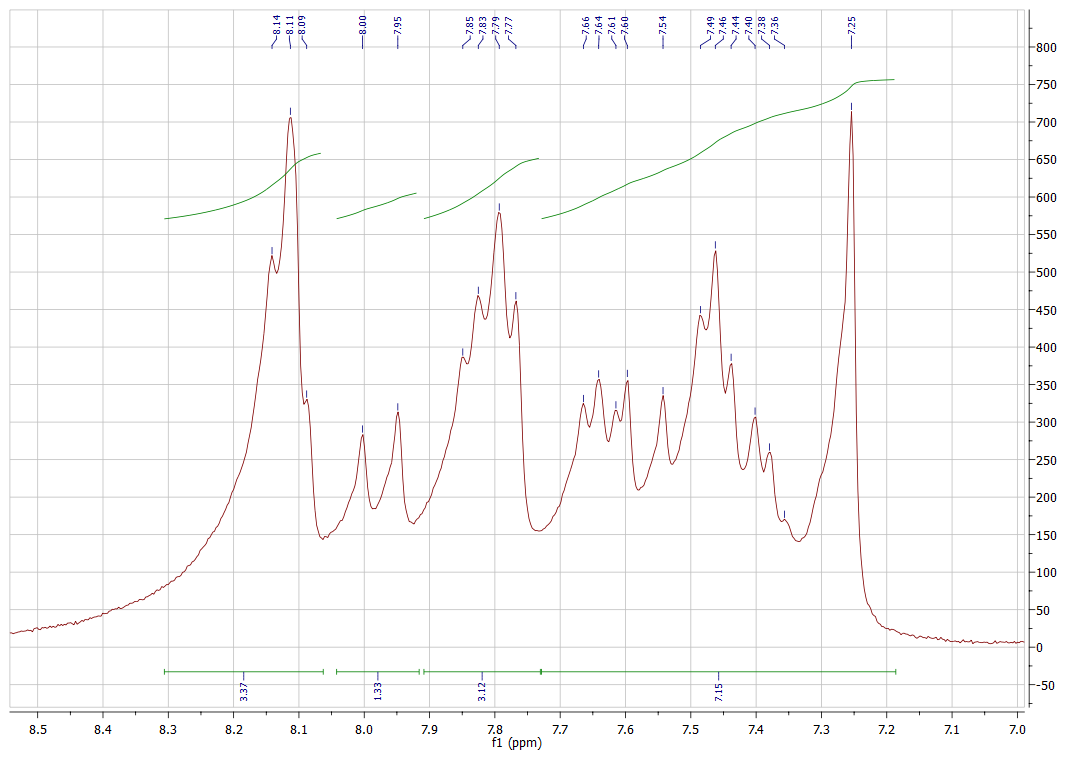


**Figure S36**. ^13^C APT NMR (DMSO-d_6_) of *(E)-*2-styryl-*N*-(3,4,5-trimethoxyphenyl)quinoline-4-carboxamide (**4e**)


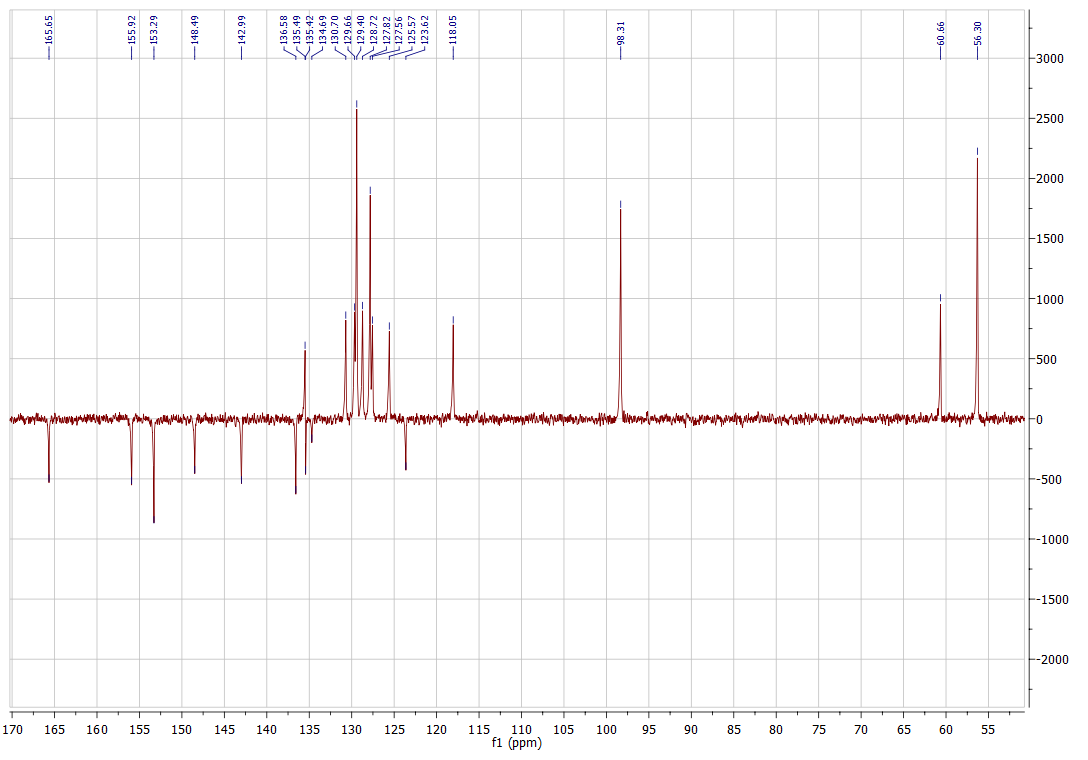


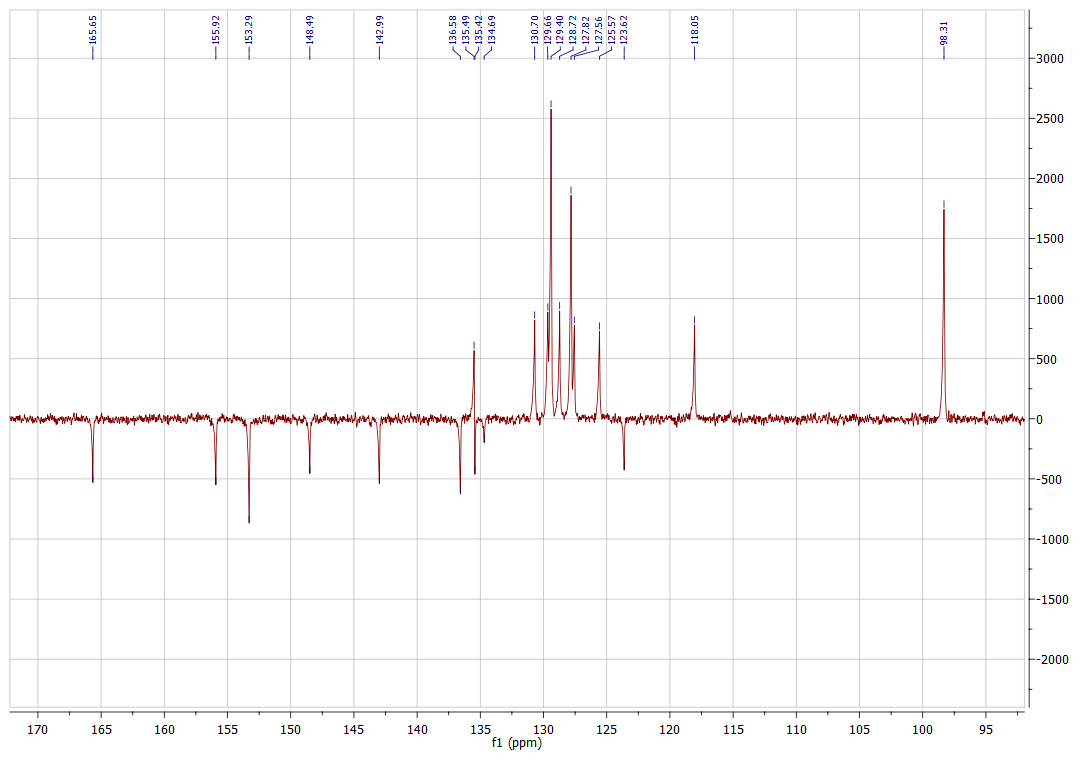


**Figure S37**. HPLC chromatogram and mass spectra of *(E)-*2-styryl-*N*-(3,4,5-trimethoxyphenyl)quinoline-4-carboxamide (**4e**)

| **Peak** | **RT** | **Area Sum %** | **Area** |
| --- | --- | --- | --- |
| 1 | 7.2134 | 0.2 | 1.93 |
| 2 | 8.1268 | 99.74 | 986.65 |
| 3 | 9.0768 | 0.06 | 0.64 |

ESI-MS analysis for [C_27_H_24_N_2_O_4_+Na^+^]: Calc.: 463.1623 *m*/*z*, exp.: *m*/*z;* 463.1622 *m*/*z.*

**Figure S38**. ^1^H NMR (DMSO-d_6_) of *(E)-N*-(3-chlorophenyl)-2-styrylquinoline-4-carboxamide (**4f**)


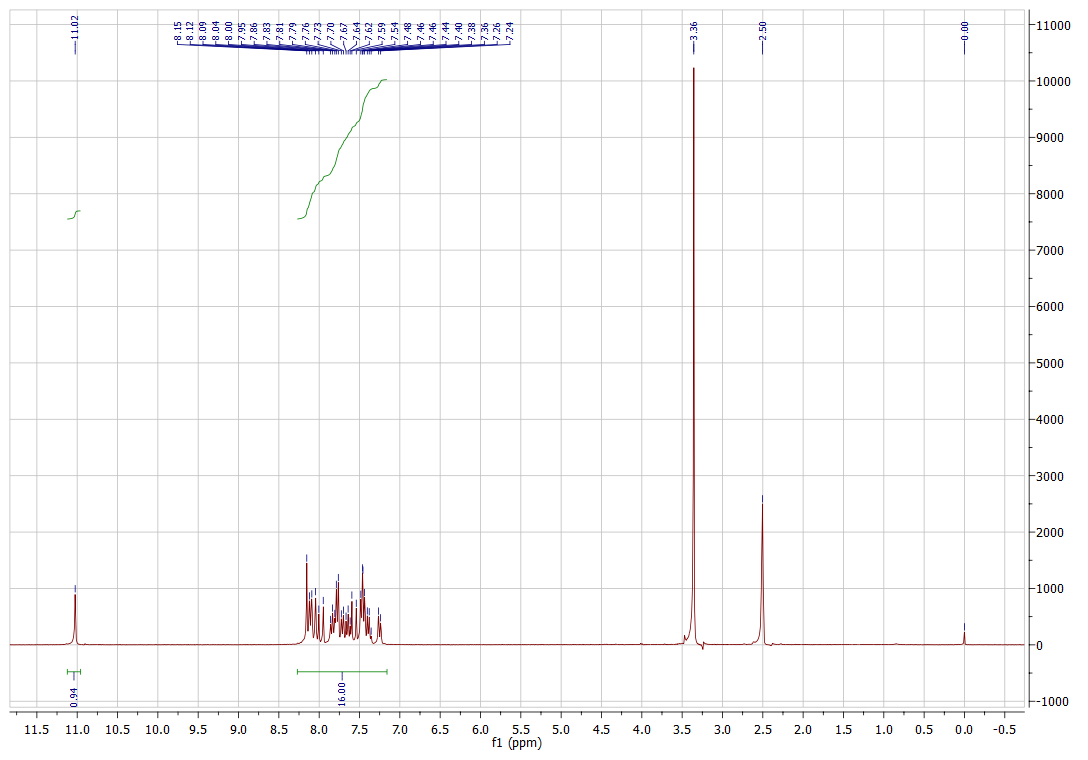


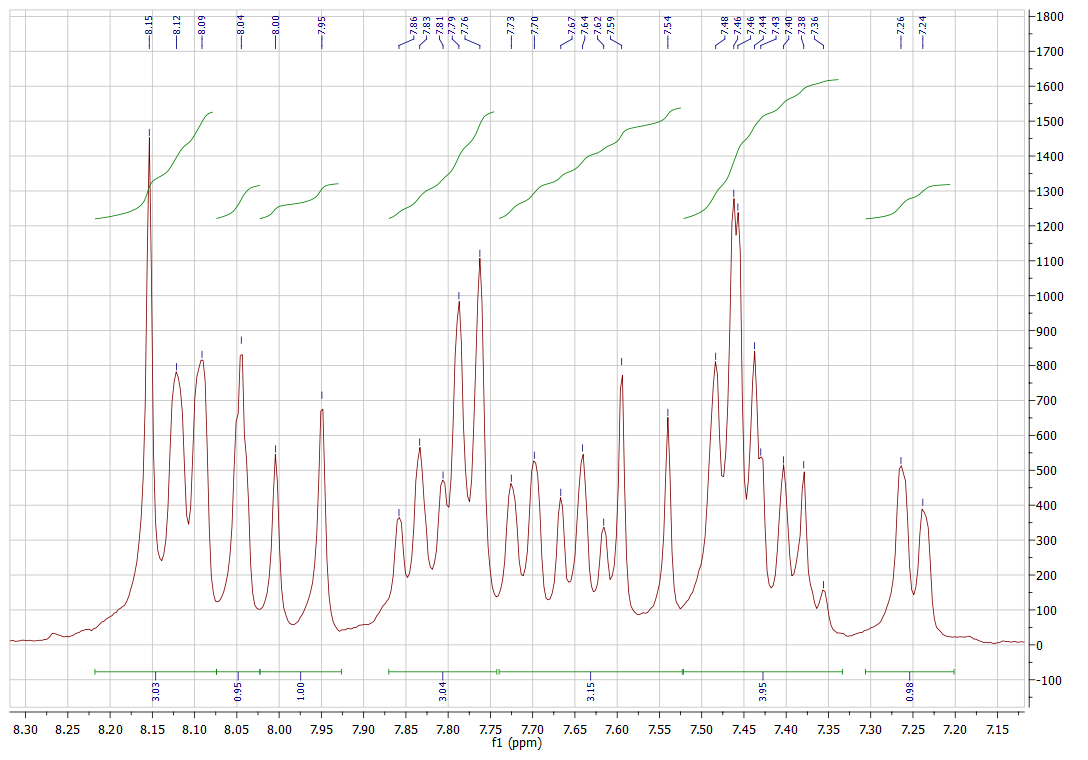


**Figure S39**. ^13^C APT NMR (DMSO-d_6_) of *(E)-N*-(3-chlorophenyl)-2-styrylquinoline-4-carboxamide (**4f**)


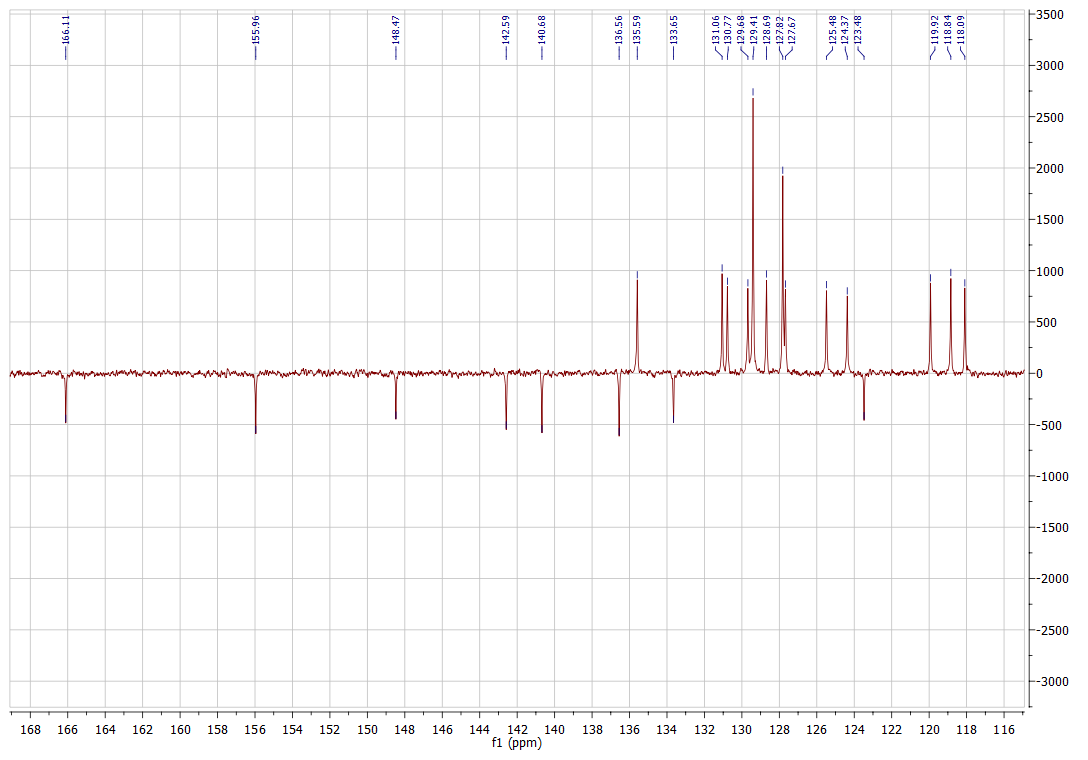


**Figure S40**. HPLC chromatogram and mass spectra of *(E)-N*-(3-chlorophenyl)-2-styrylquinoline-4-carboxamide (**4f**)

| **Peak** | **RT** | **Area Sum %** | **Area** |
| --- | --- | --- | --- |
| 1 | 6.7136 | 0.6 | 2.44 |
| 2 | 7.2203 | 0.47 | 1.92 |
| 3 | 9.0870 | 0.44 | 1.8 |
| 4 | 9.3403 | 98.49 | 401.97 |

ESI-MS analysis for [C_24_H_17_ClN_2_O+H^+^]: Calc.: 385.1102 *m*/*z*, exp.: 385.1102 *m*/*z.*

**Figure S41**. ^1^H NMR (DMSO-d_6_) of *(E)-N*-(4-chlorophenyl)-2-styrylquinoline-4-carboxamide (**4g**)


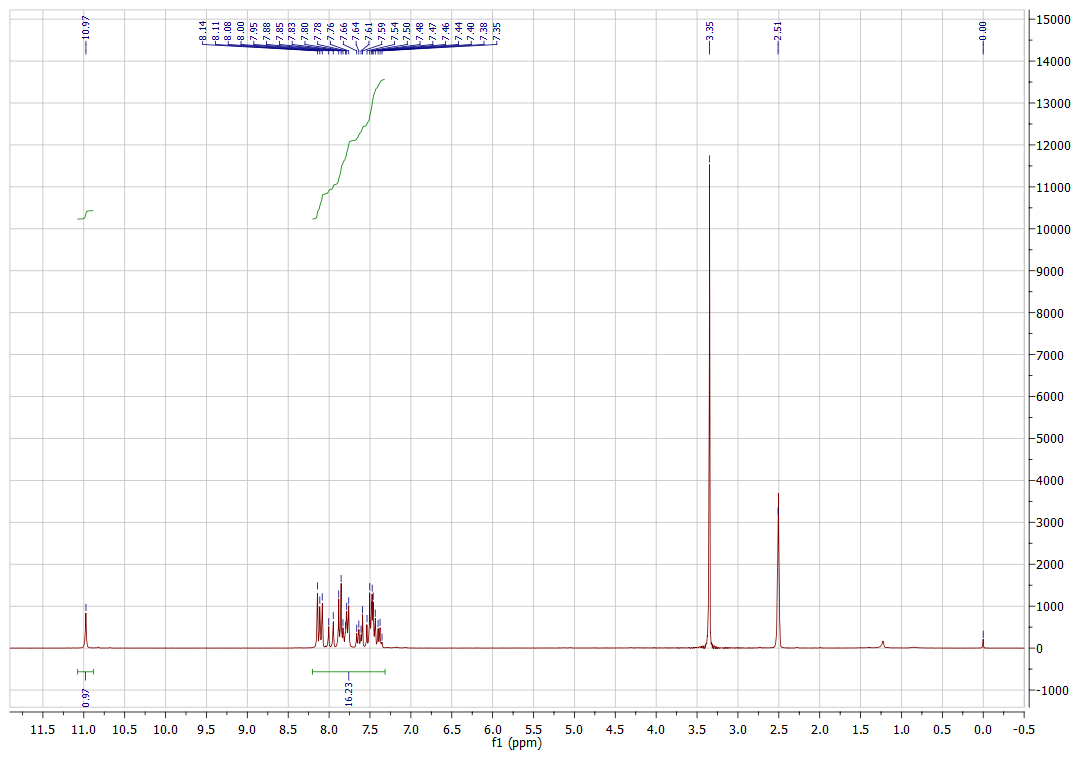


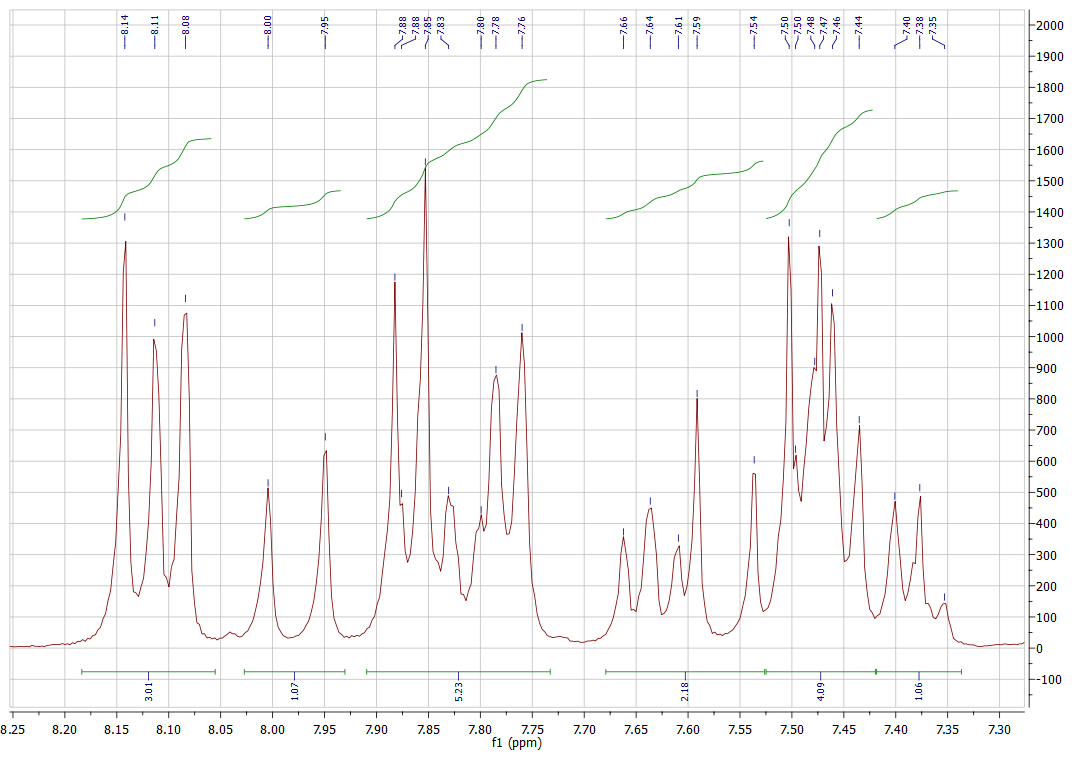


**Figure S42**. ^13^C APT NMR (DMSO-d_6_) of *(E)-N*-(4-chlorophenyl)-2-styrylquinoline-4-carboxamide (**4g**)


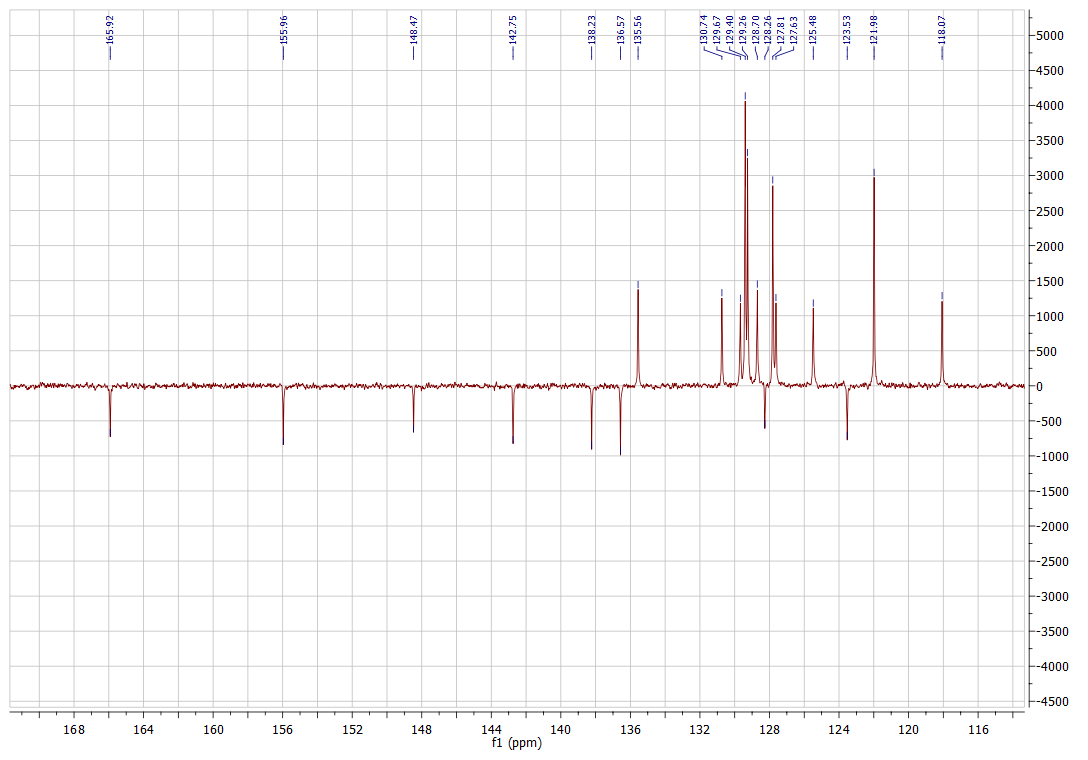


**Figure S43**. HPLC chromatogram and mass spectra of *(E)-N*-(4-chlorophenyl)-2-styrylquinoline-4-carboxamide (**4g**)

| **Peak** | **RT** | **Area Sum %** | **Area** |
| --- | --- | --- | --- |
| 1 | 7.2420 | 0.34 | 1.76 |
| 2 | 8.4853 | 0.12 | 0.62 |
| 3 | 9.2853 | 98.32 | 509.9 |
| 4 | 10.2186 | 1.22 | 6.32 |

ESI-MS analysis for [C_24_H_17_ClN_2_O+H^+^]: Calc.: 385.1102 *m*/*z*, exp.: 385.1102 *m*/*z.*

**Figure S44**. ^1^H NMR (DMSO-d_6_) of *(E)-*2-styryl-*N*-(4-(trifluoromethyl)phenyl)quinoline-4-carboxamide (**4h**)


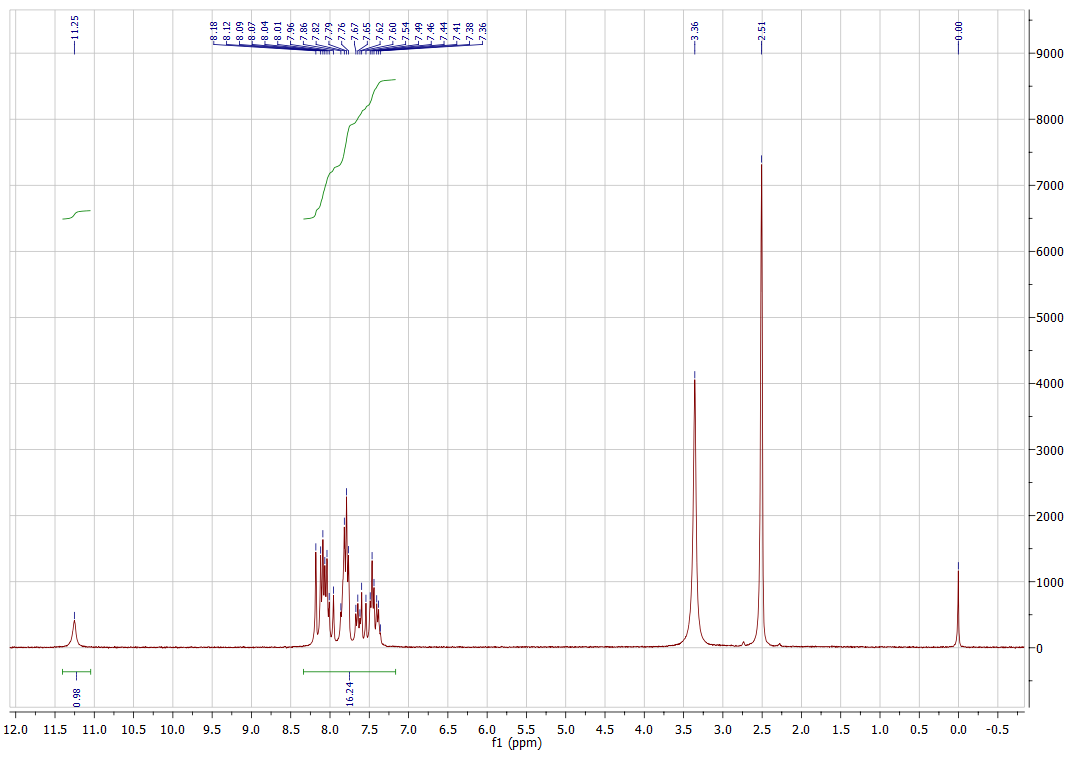


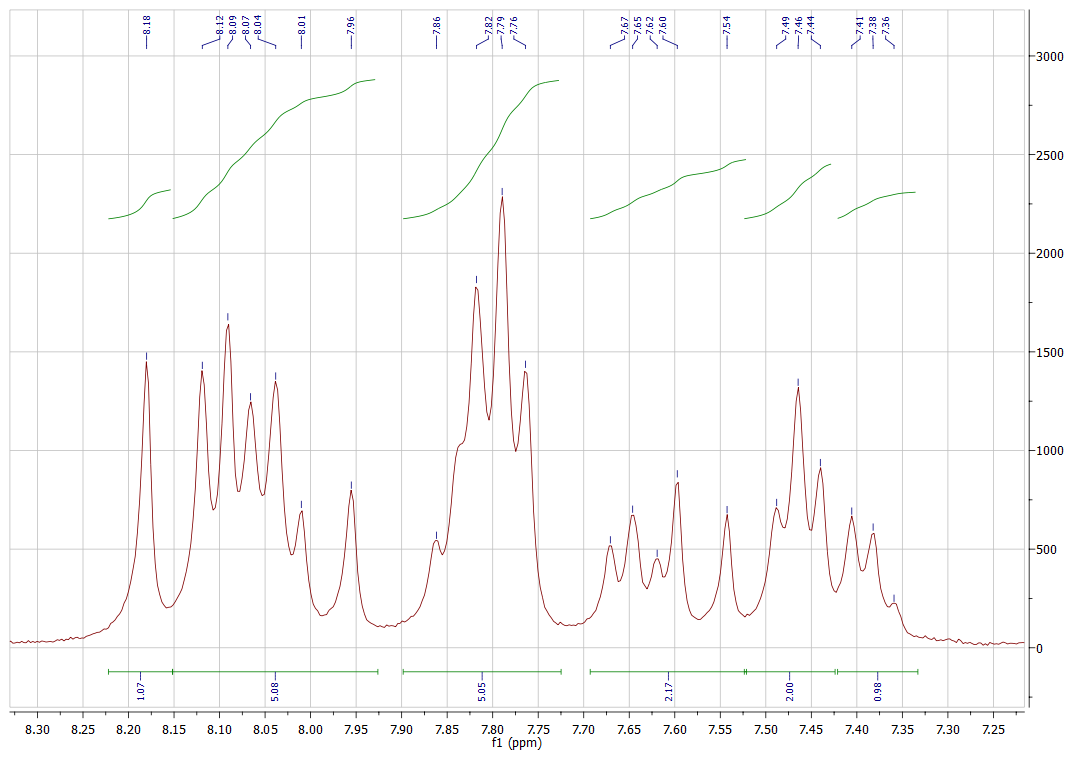


**Figure S45**. ^13^C APT NMR (DMSO-d_6_) of *(E)-*2-styryl-*N*-(4-(trifluoromethyl)phenyl)quinoline-4-carboxamide (**4h**)


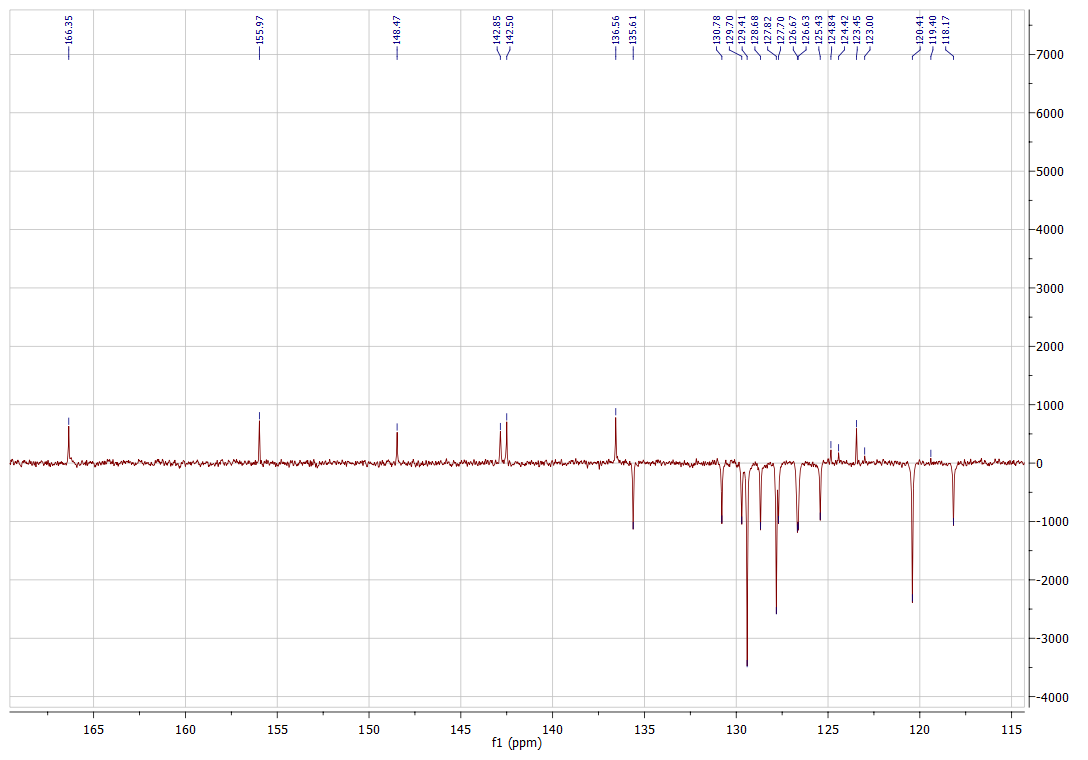


**Figure S46**. HPLC chromatogram and mass spectra of *(E)-*2-styryl-*N*-(4-(trifluoromethyl)phenyl)quinoline-4-carboxamide (**4h**)

| **Peak** | **RT** | **Area Sum %** | **Area** |
| --- | --- | --- | --- |
| 1 | 8.8672 | 0.36 | 1.92 |
| 2 | 9.3905 | 0.46 | 2.42 |
| 3 | 9.6372 | 98.78 | 525.01 |
| 4 | 10.5839 | 0.4 | 2.14 |

ESI-MS analysis for [C_25_H_17_F_3_N_2_O+H^+^]: Calc.: 419.1366 *m*/*z*, exp.: *m*/*z;* 419.1366 *m*/*z*.

**Figure S47**. ^1^H NMR (DMSO-d_6_) of *(E)-N*-(4-nitrophenyl)-2-styrylquinoline-4-carboxamide (**4i**)


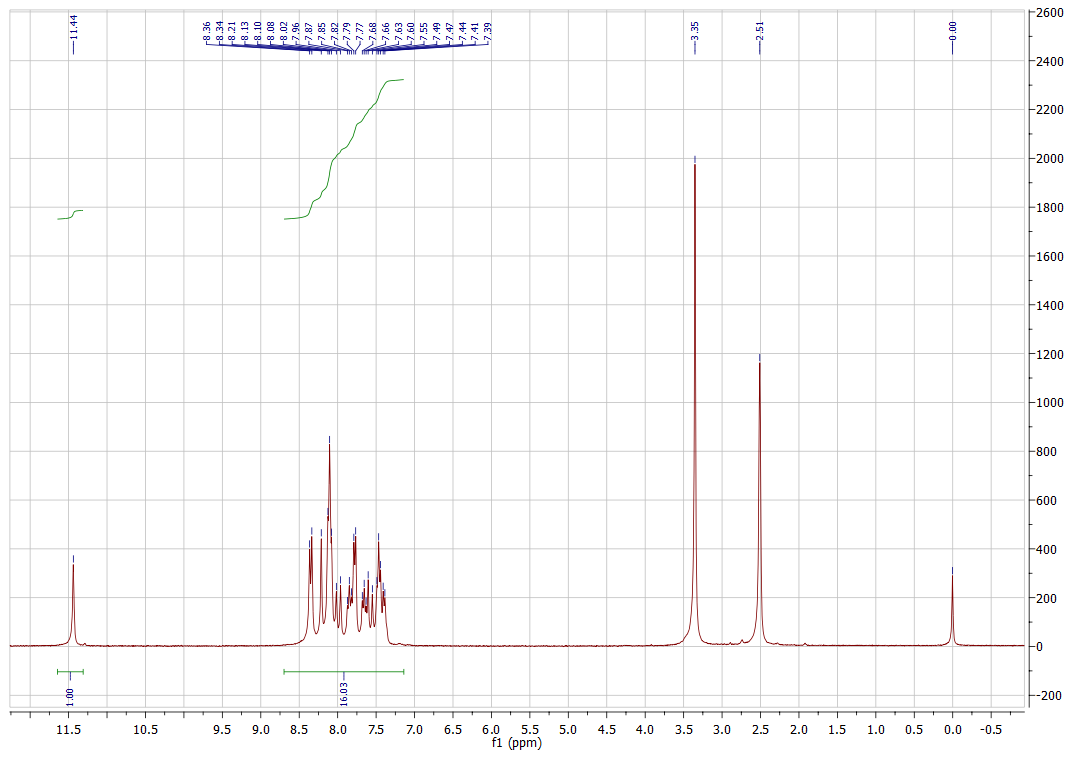


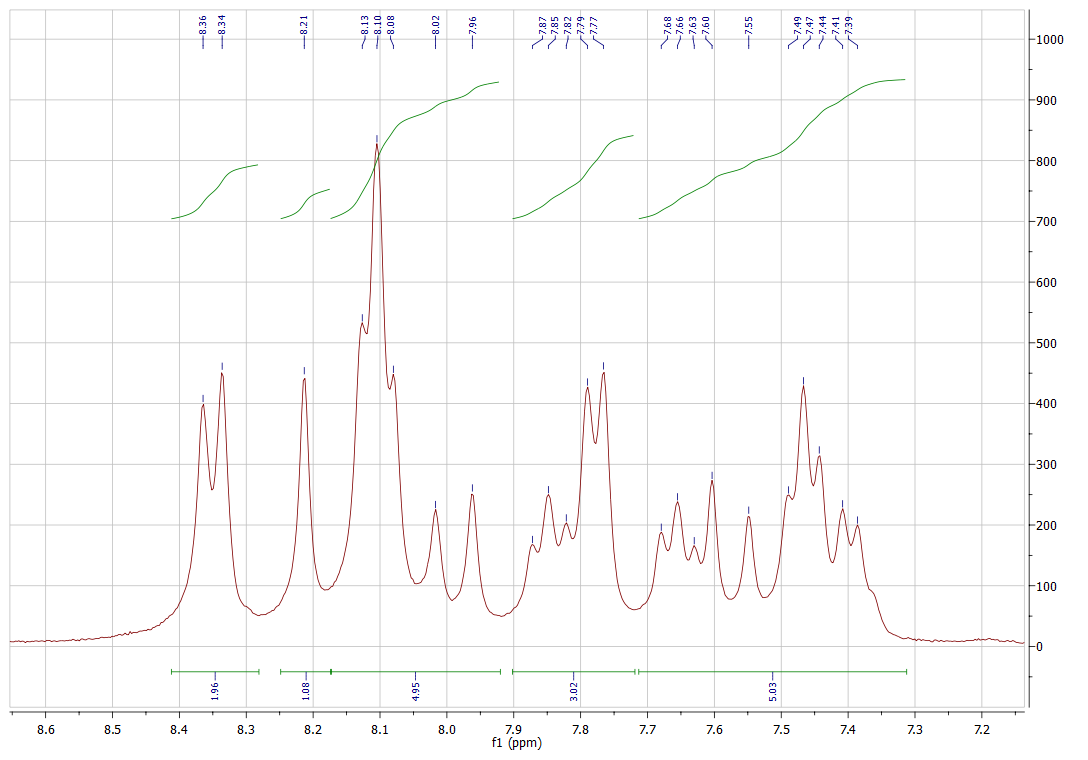


**Figure S48**. ^13^C APT NMR (DMSO-d_6_) of *(E)-N*-(4-nitrophenyl)-2-styrylquinoline-4-carboxamide (**4i**)


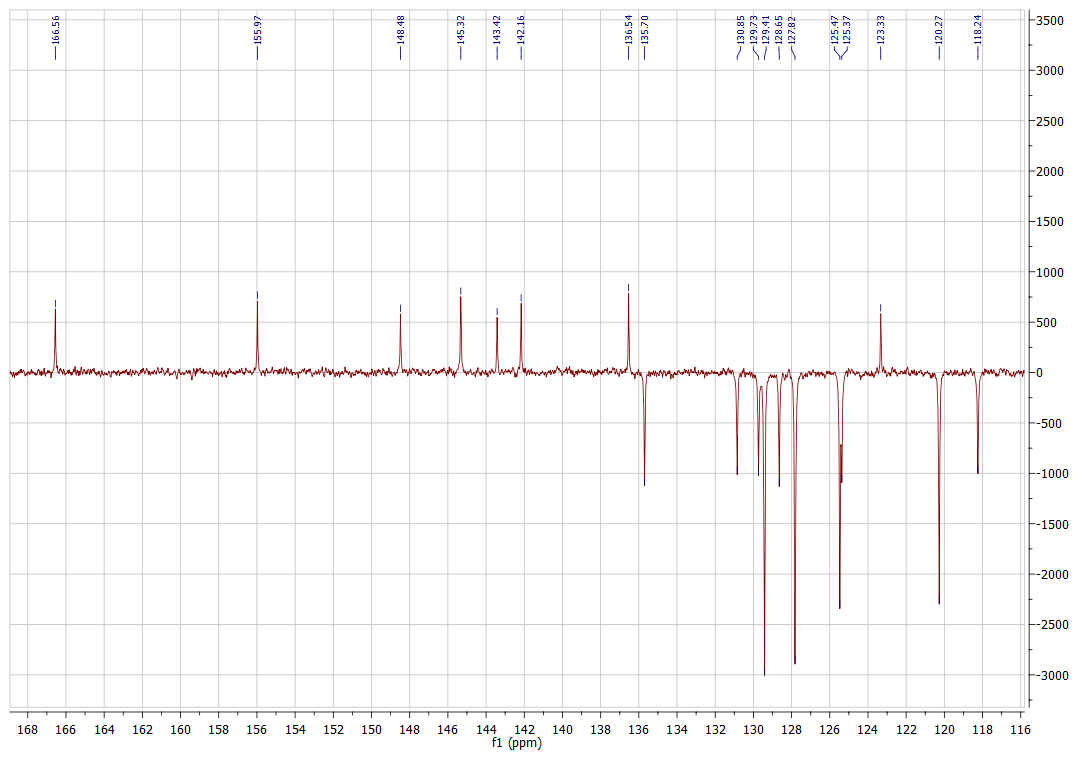


**Figure S49**. HPLC chromatogram and mass spectra of *(E)-N*-(4-nitrophenyl)-2-styrylquinoline-4-carboxamide (**4i**)

| **Peak** | **RT** | **Area Sum %** | **Area** |
| --- | --- | --- | --- |
| 1 | 8.416 | 0.77 | 1.96 |
| 2 | 8.733 | 98.41 | 249.71 |
| 3 | 9.350 | 0.82 | 2.07 |


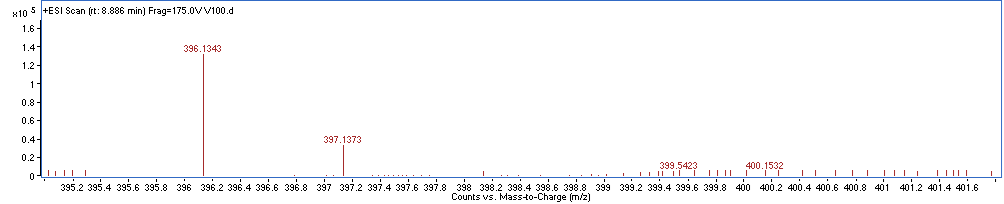


ESI-MS analysis for [C_24_H_17_N_3_O_3_+H^+^]: Calc.: 396.134 *m*/*z*, exp.: 396.1343 *m*/*z*.

**Figure S50**. ^1^H NMR (DMSO-d_6_) of *(E)-N-*(4-nitro-3-(trifluoromethyl)phenyl)-2-styrylquinoline-4-carboxamide (**4j**)


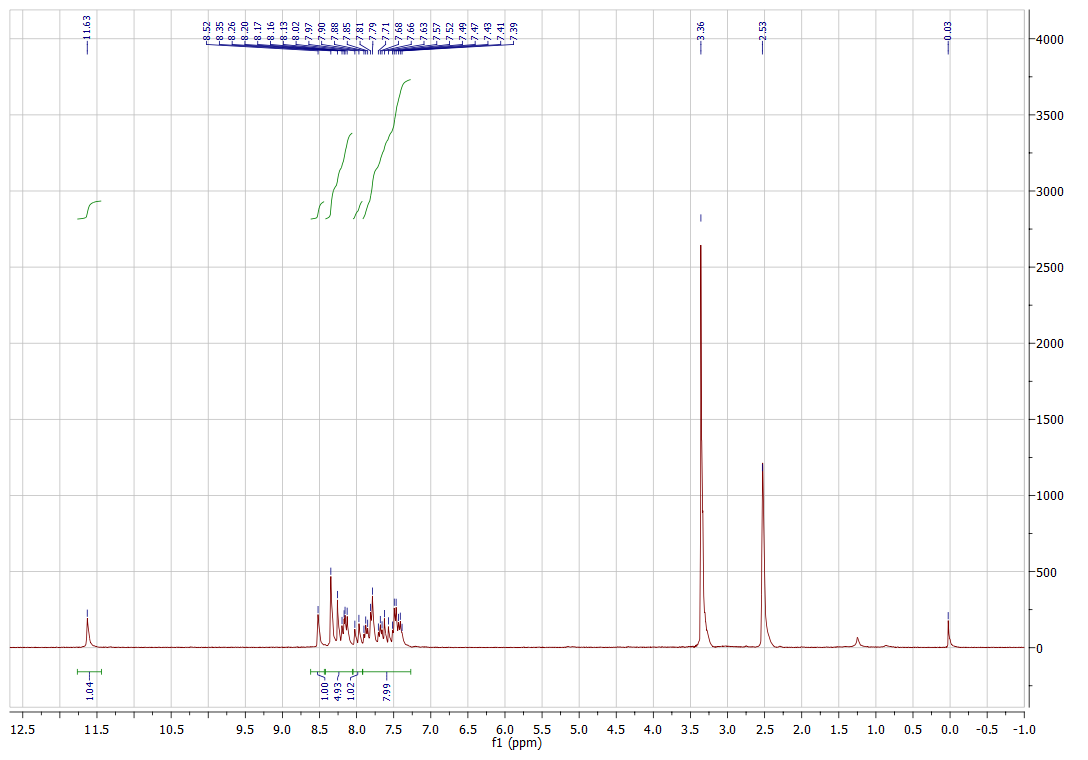


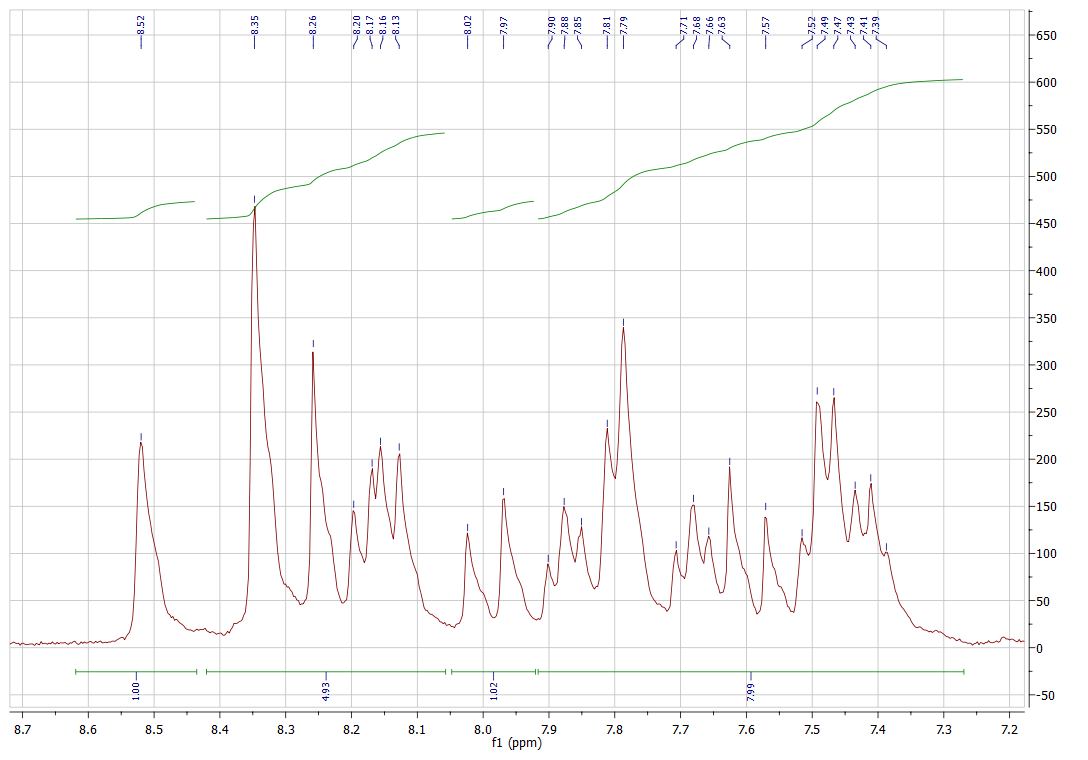


**Figure S51**. ^13^C APT NMR (DMSO-d_6_) of *(E)-N-*(4-nitro-3-(trifluoromethyl)phenyl)-2-styrylquinoline-4-carboxamide (**4j**)


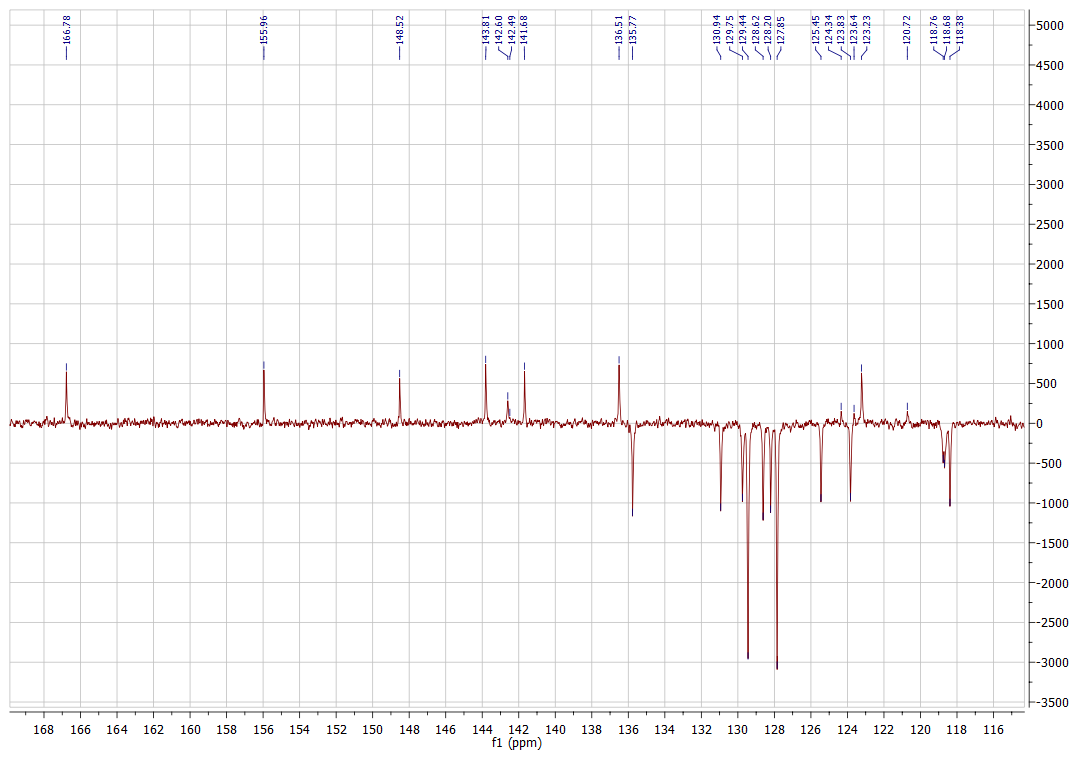


**Figure S52**. HPLC chromatogram and mass spectra of *(E)-N-*(4-nitro-3-(trifluoromethyl)phenyl)-2-styrylquinoline-4-carboxamide (**4j**)

| **Peak** | **RT** | **Area Sum %** | **Area** |
| --- | --- | --- | --- |
| 1 | 8.3274 | 0.57 | 7.24 |
| 2 | 9.4107 | 0.43 | 5.5 |
| 3 | 9.7141 | 98.85 | 1250.67 |
| 4 | 10.2907 | 0.14 | 1.76 |

ESI-MS analysis for [C_25_H_16_F_3_N_3_O_3_+Na^+^]: Calc.: 486.1036 *m*/*z*, exp.: 486.1034 *m*/*z*.

**Figure S53**. ^1^H NMR (DMSO-d_6_) of *(E)-N*-phenyl-2-(3,4,5-trimethoxystyryl)quinoline-4-carboxamide (**4k**)


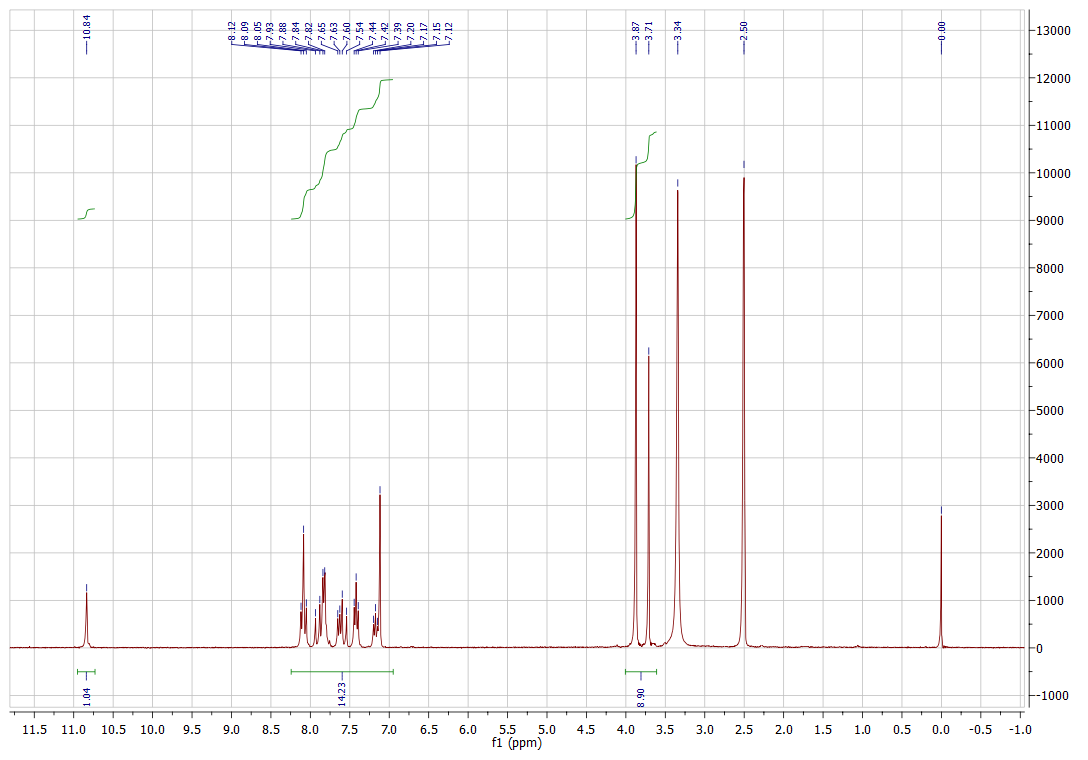


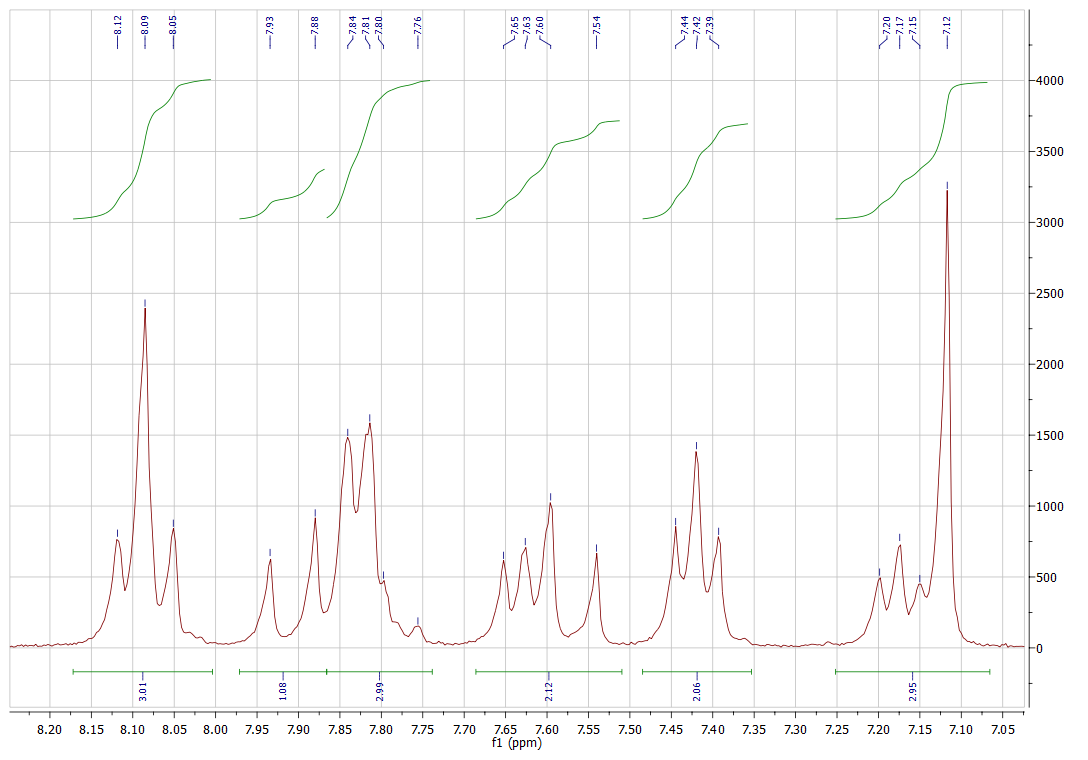


**Figure S54**. ^13^C APT NMR (DMSO-d_6_) of *(E)-N*-phenyl-2-(3,4,5-trimethoxystyryl)quinoline-4-carboxamide (**4k**)


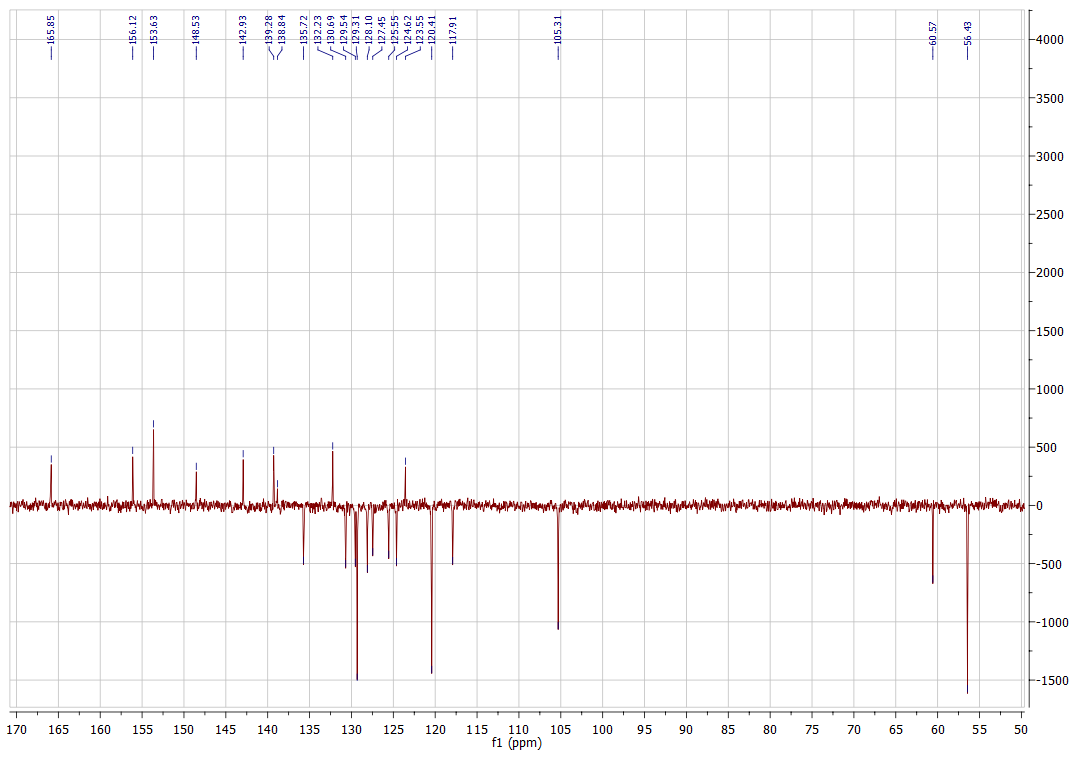


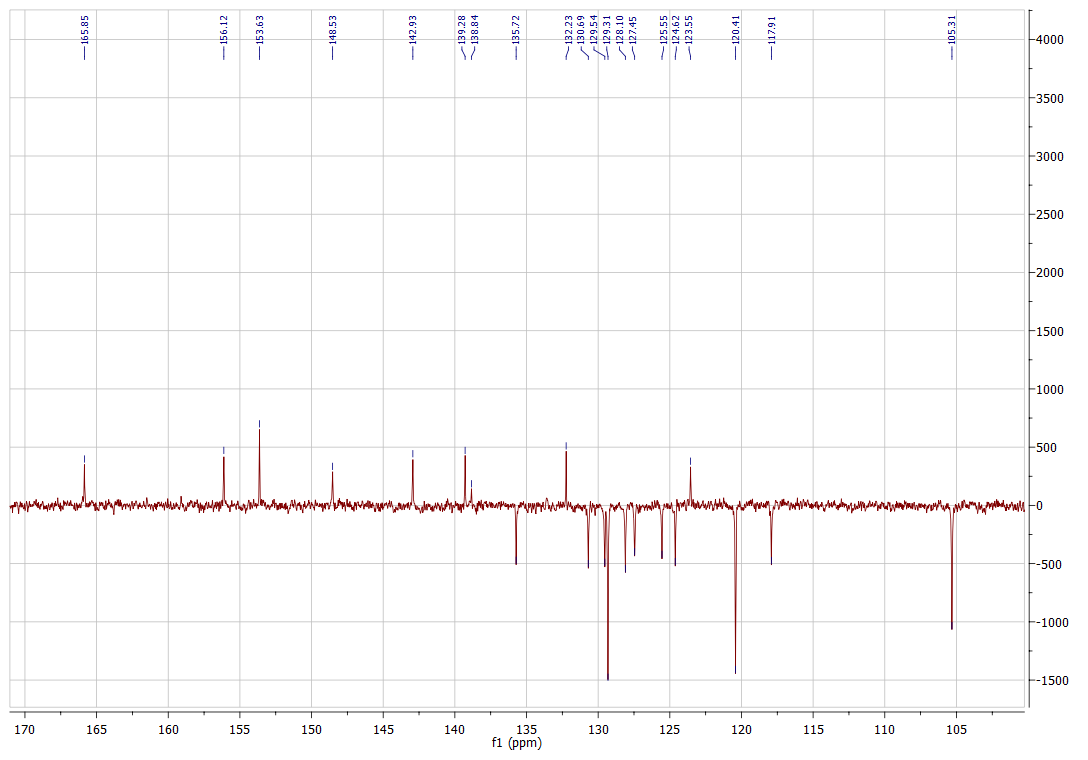


**Figure S55**. HPLC chromatogram and mass spectra of *(E)-N*-phenyl-2-(3,4,5-trimethoxystyryl)quinoline-4-carboxamide (**4k**)

| **Peak** | **RT** | **Area Sum %** | **Area** |
| --- | --- | --- | --- |
| 1 | 8.1009 | 98.03 | 2050.51 |
| 2 | 8.5409 | 1.97 | 41.42 |

ESI-MS analysis for [C_27_H_24_N_2_O_4_+Na^+^]: Calc.: 463.1628 *m*/*z*, exp.: 463.1632 *m*/*z.*

**Figure S56**. ^1^H NMR (DMSO-d_6_) of *(E)-N*-(m-tolyl)-2-(3,4,5-trimethoxystyryl)quinoline-4-carboxamide (**4l**)


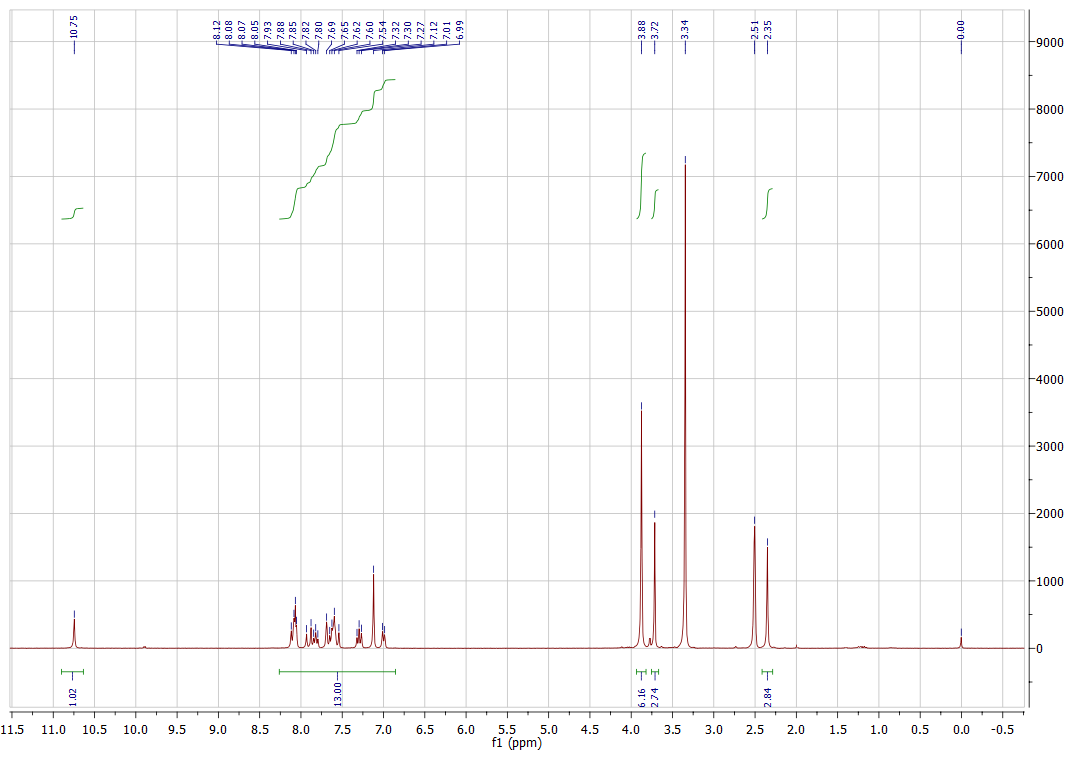


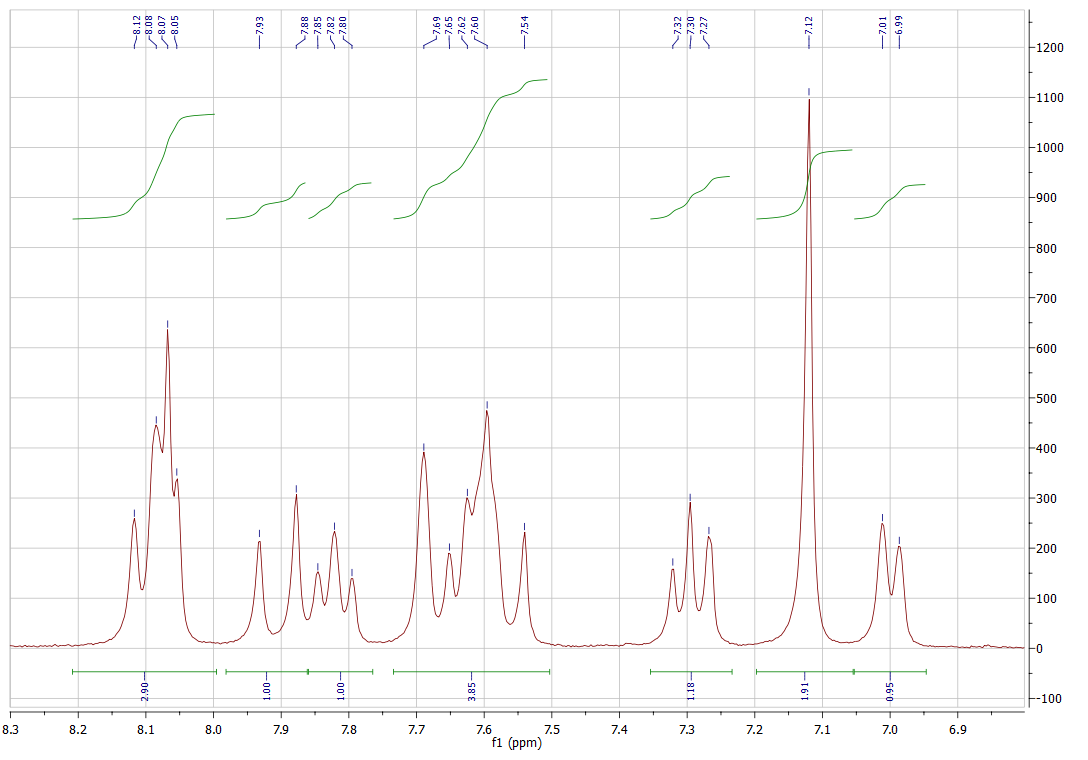


**Figure S57**. ^13^C APT NMR (DMSO-d_6_) of *(E)-N*-(m-tolyl)-2-(3,4,5-trimethoxystyryl)quinoline-4-carboxamide (**4l**)


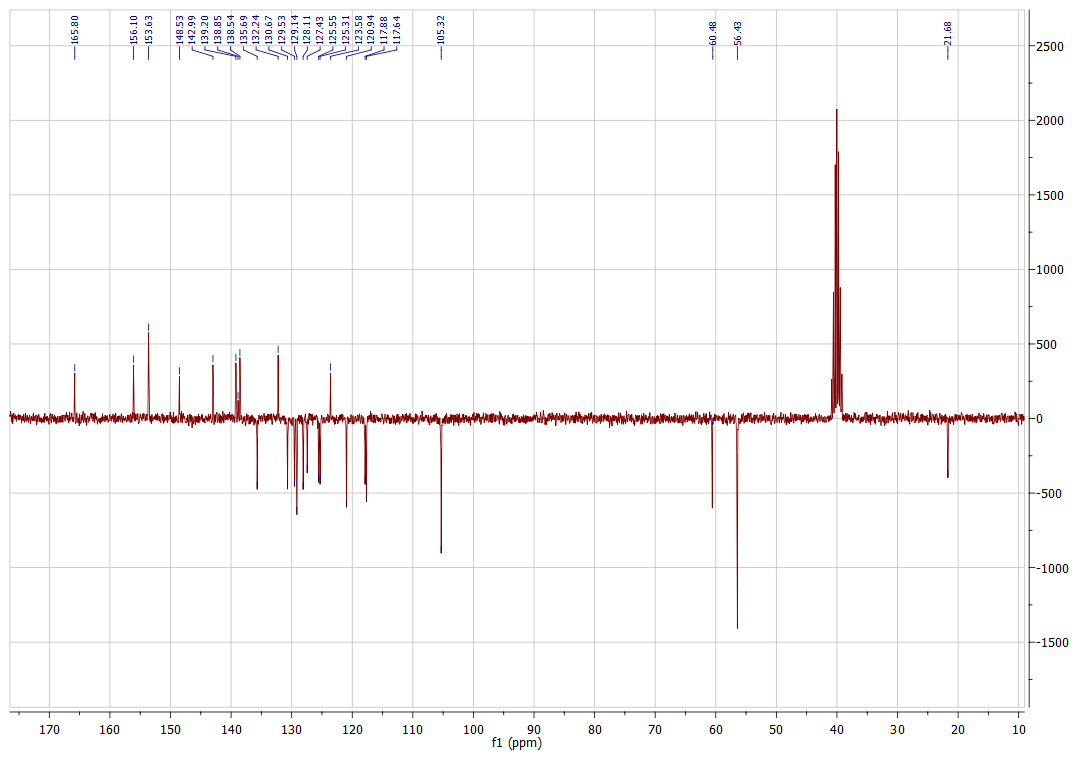


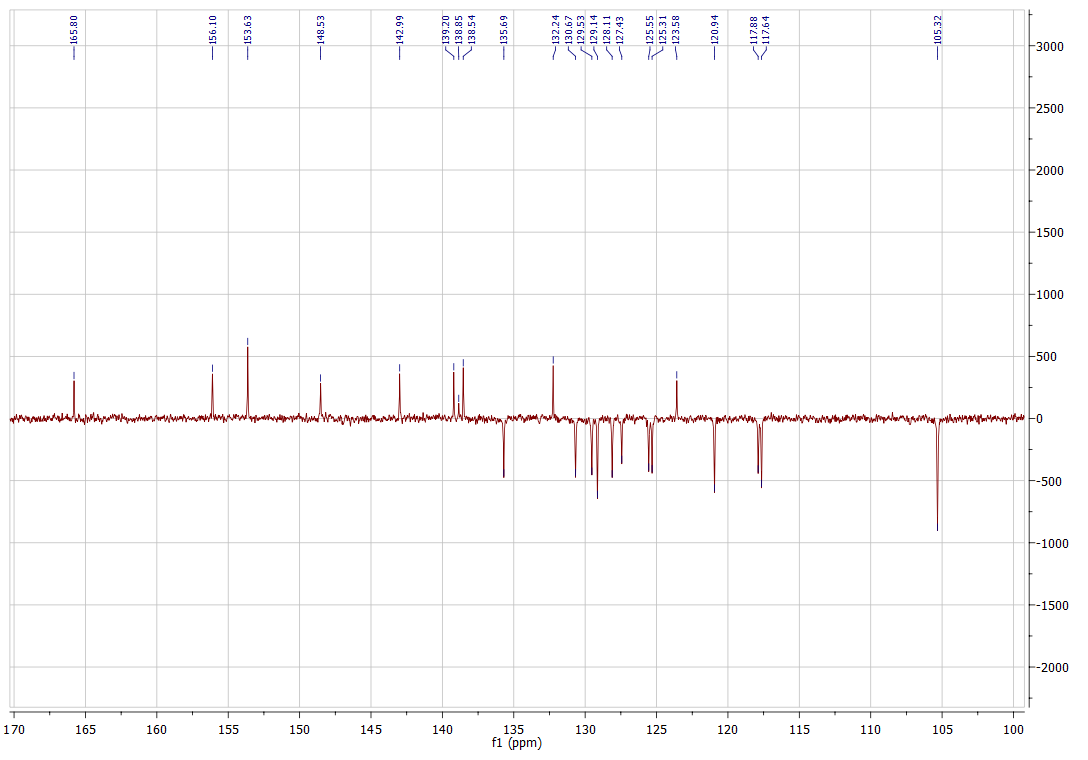


**Figure S58**. HPLC chromatogram and mass spectra of *(E)-N*-(m-tolyl)-2-(3,4,5-trimethoxystyryl)quinoline-4-carboxamide (**4l**)

| **Peak** | **RT** | **Area Sum %** | **Area** |
| --- | --- | --- | --- |
| 1 | 5.7228 | 0.27 | 1.77 |
| 2 | 6.7103 | 0.63 | 4.19 |
| 3 | 7.2228 | 0.23 | 1.55 |
| 4 | 8.5228 | 98.87 | 656.7 |

ESI-MS analysis for C_28_H_26_N_2_O_4_ [2M+Na^+^]: Calc.: 931.3677 *m*/*z*, exp.: 931.3676 *m*/*z.*

**Figure S59**. ^1^H NMR (DMSO-d_6_) of *(E)-N*-(p-tolyl)-2-(3,4,5-trimethoxystyryl)quinoline-4-carboxamide (**4m**)


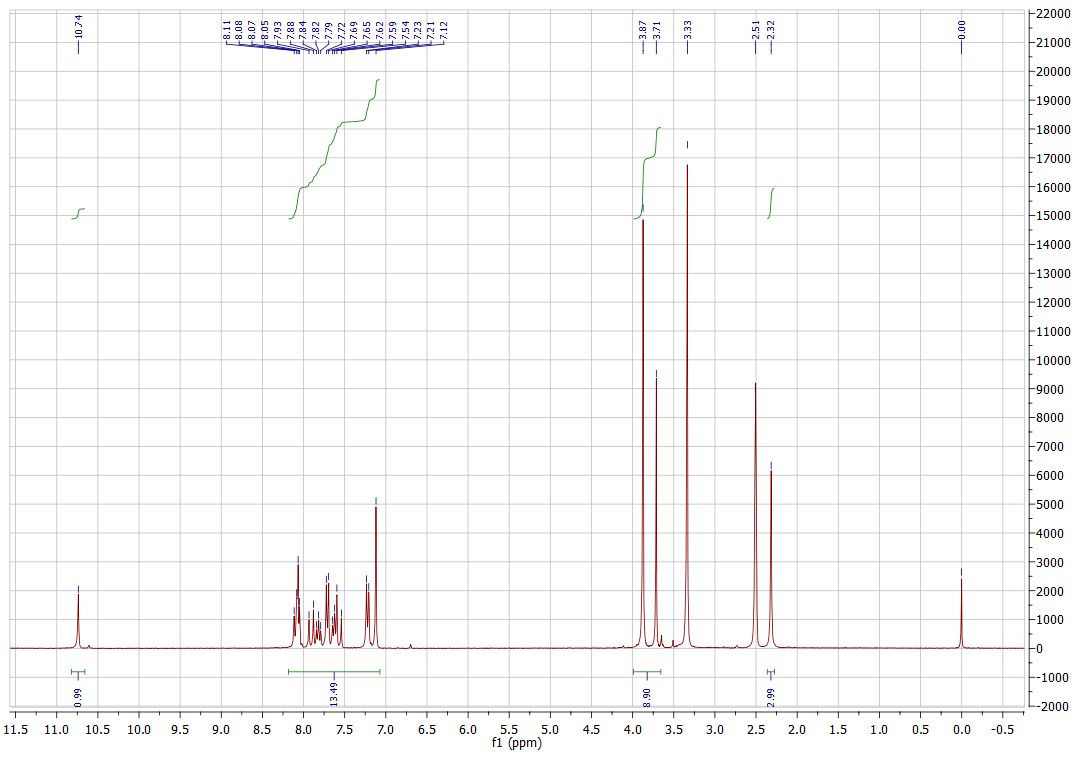


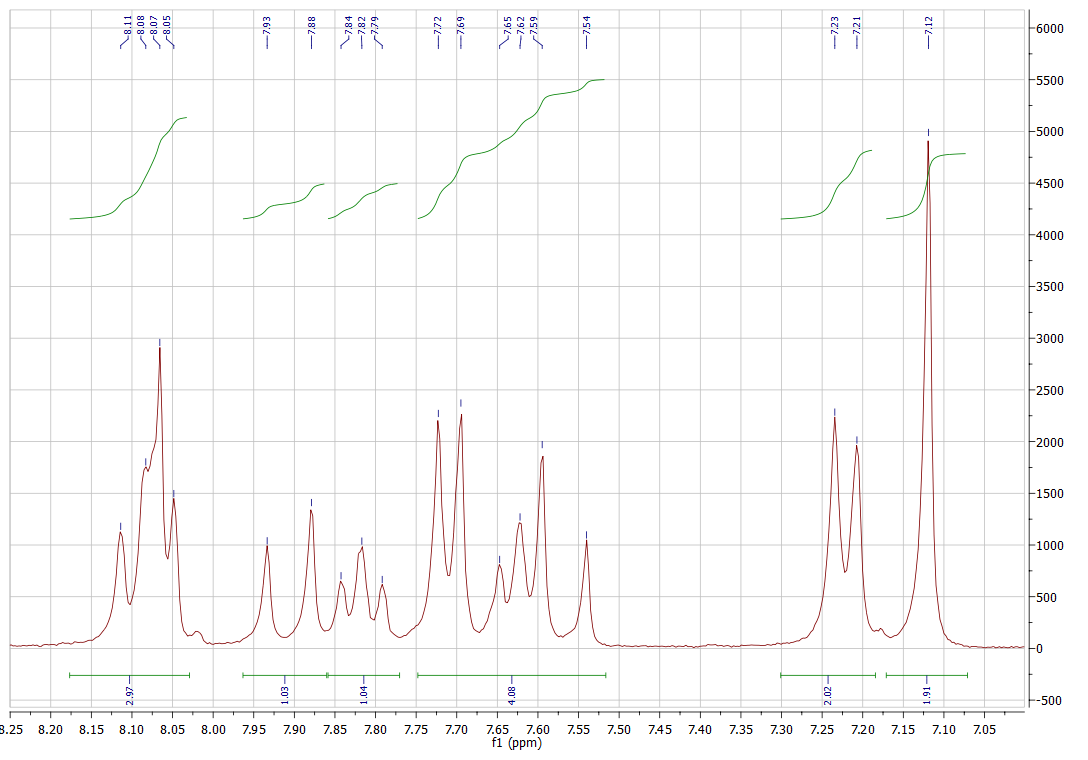


**Figure S60**. ^13^C APT NMR (DMSO-d_6_) of *(E)-N*-(p-tolyl)-2-(3,4,5-trimethoxystyryl)quinoline-4-carboxamide (**4m**)


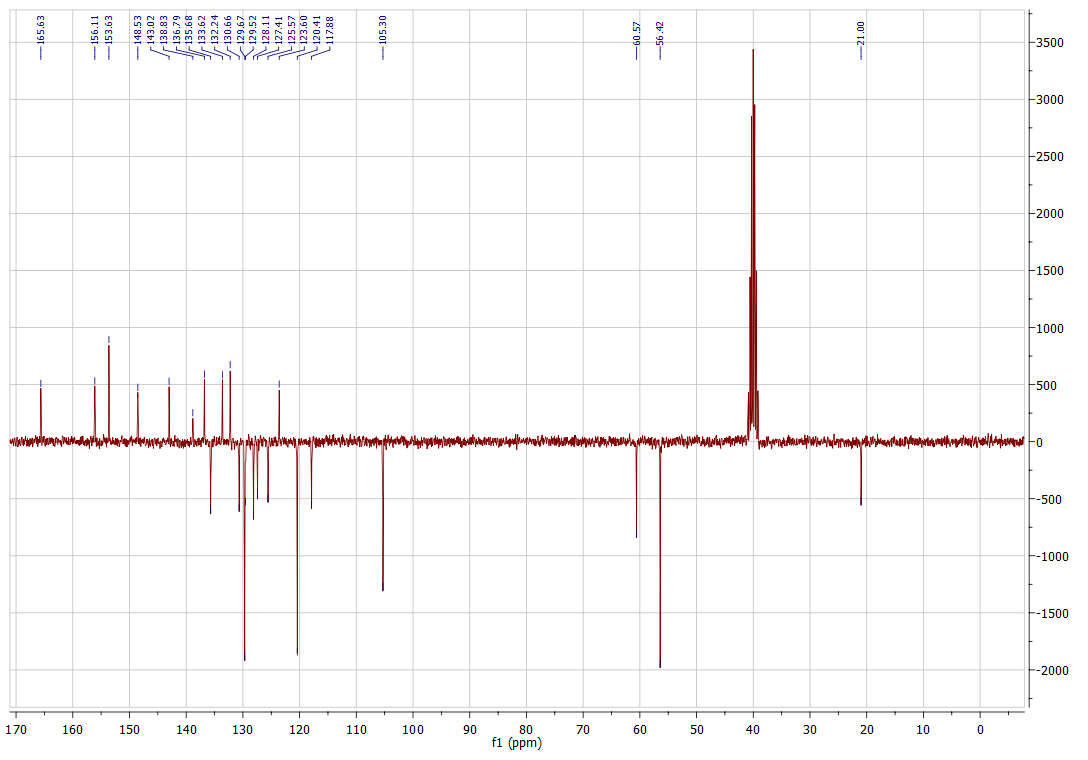


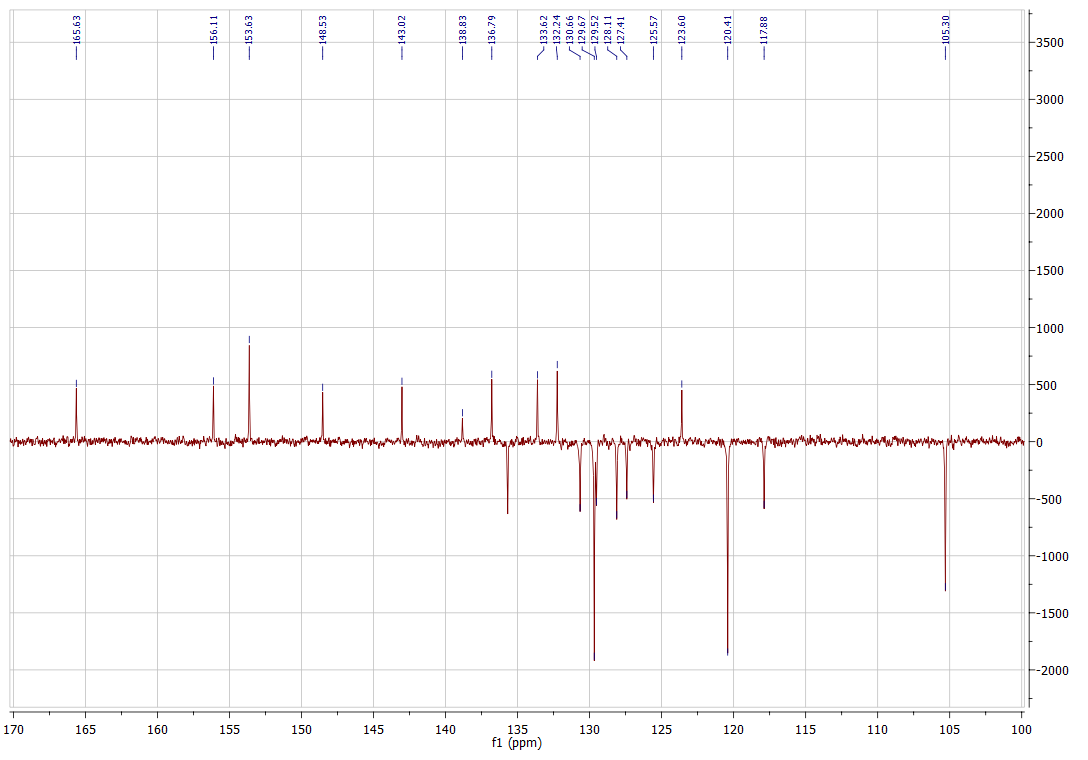


**Figure S61**. HPLC chromatogram and mass spectra of *(E)-N*-(p-tolyl)-2-(3,4,5-trimethoxystyryl)quinoline-4-carboxamide (**4m**)

| **Peak** | **RT** | **Area Sum %** | **Area** |
| --- | --- | --- | --- |
| 1 | 8.4911 | 98.00 | 1691.04 |
| 2 | 9.1678 | 2.00 | 34.59 |

ESI-MS analysis for [C_28_H_26_N_2_O_4_+H^+^]: Calc.: 455.1965 *m*/*z*, exp.: 455.1965 *m*/*z.*

**Figure S62**. ^1^H NMR (DMSO-d_6_) of *(E)-N*-(2,6-dimethylphenyl)-2-(3,4,5-trimethoxystyryl)quinoline-4-carboxamide (**4n**)


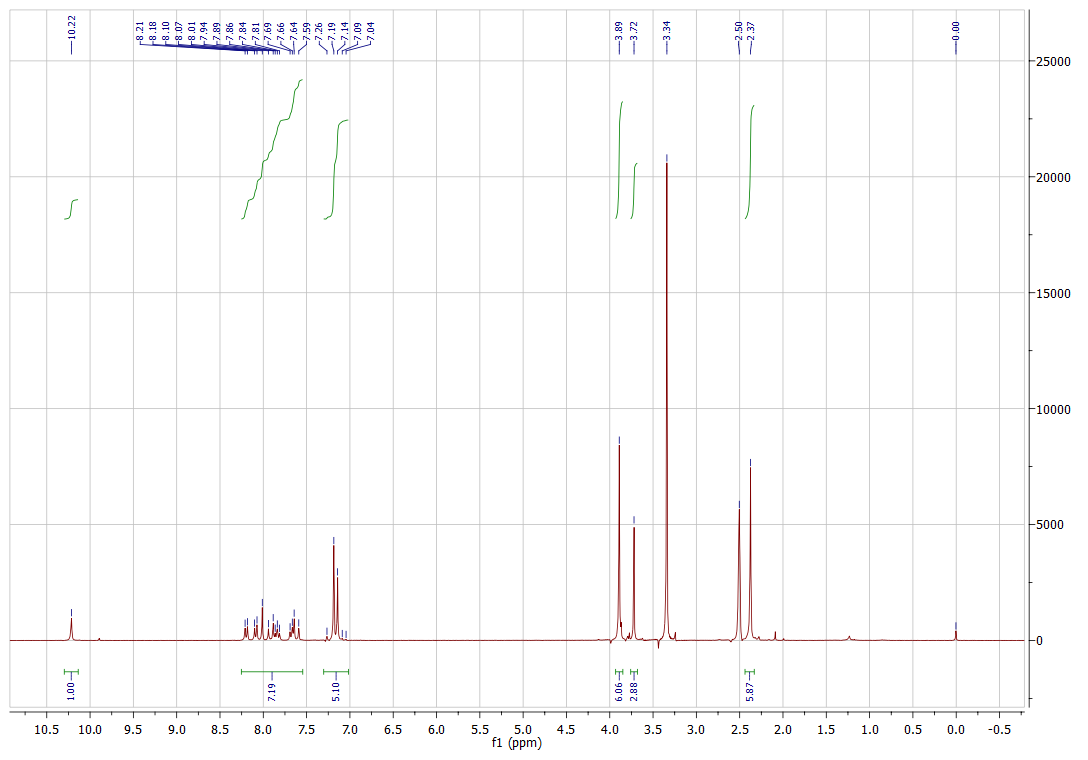


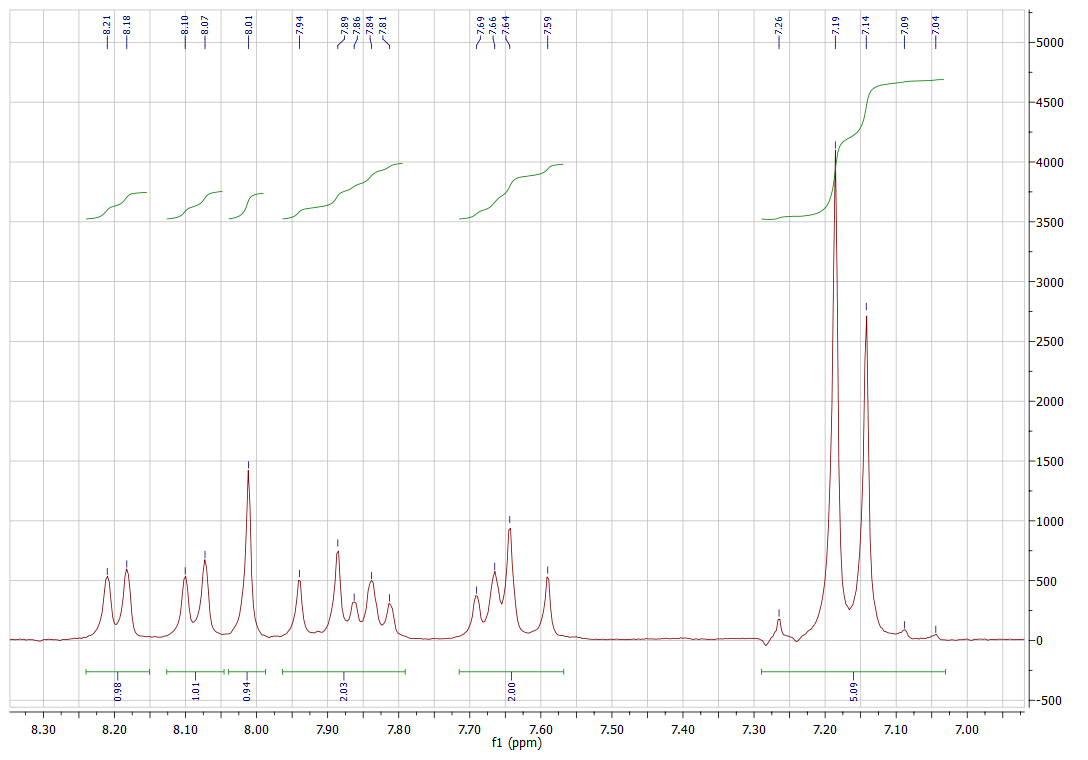


**Figure S63**. ^13^C APT NMR (DMSO-d_6_) of *(E)-N*-(2,6-dimethylphenyl)-2-(3,4,5-trimethoxystyryl)quinoline-4-carboxamide (**4n**)


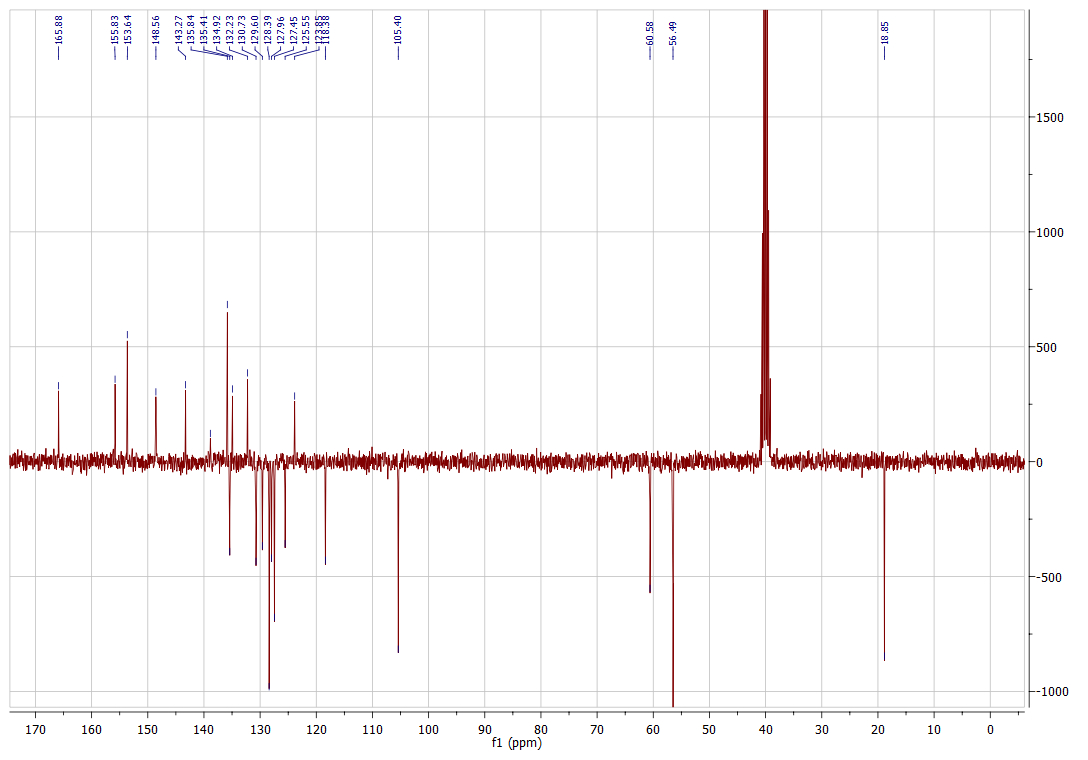


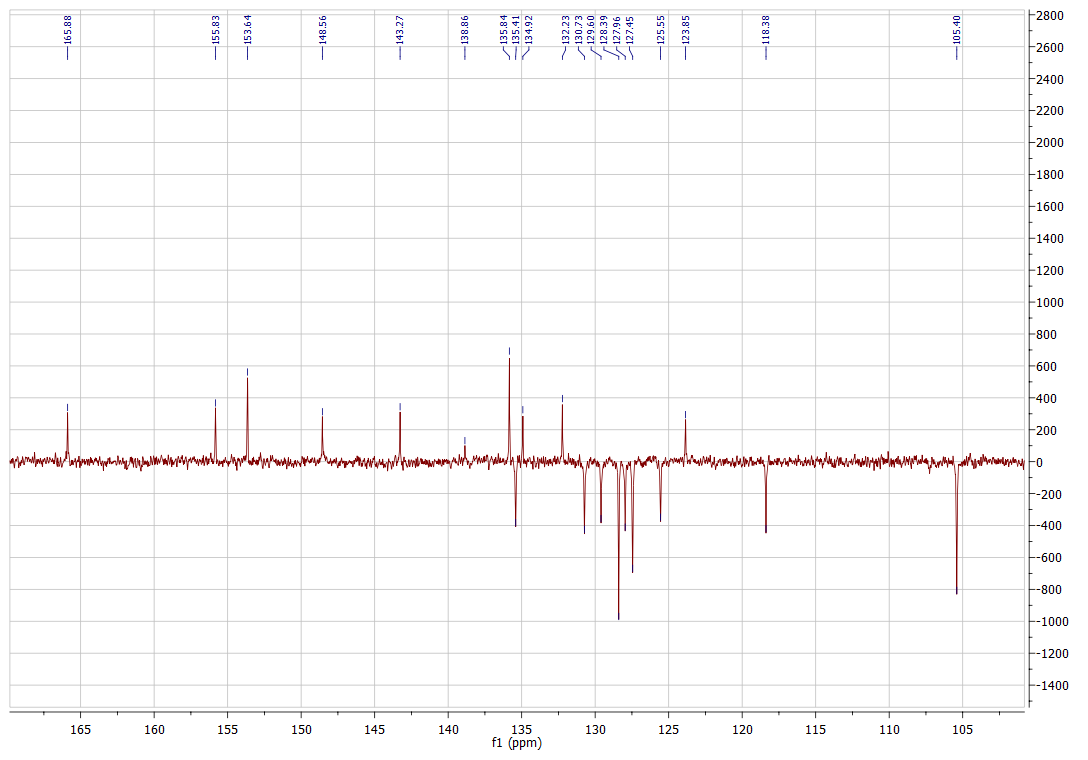


**Figure S64**. HPLC chromatogram and mass spectra of *(E)-N*-(2,6-dimethylphenyl)-2-(3,4,5-trimethoxystyryl)quinoline-4-carboxamide (**4n**)

| **Peak** | **RT** | **Area Sum %** | **Area** |
| --- | --- | --- | --- |
| 1 | 7.947 | 99.75 | 352.86 |
| 2 | 8.220 | 0.25 | 0.9 |

**
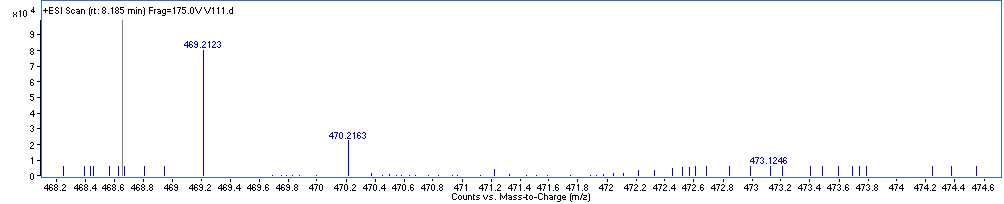
**

ESI-MS analysis for [C_29_H_28_N_2_O_4_+H^+^]: Calc.: 469.2122 *m*/*z*, exp.: 469.2123 *m*/*z*.

**Figure S65** ^1^H NMR (DMSO-d_6_) of *(E)-N*-(3,4,5-trimethoxyphenyl)-2-(3,4,5-trimethoxystyryl)quinoline-4-carboxamide (**4o**)


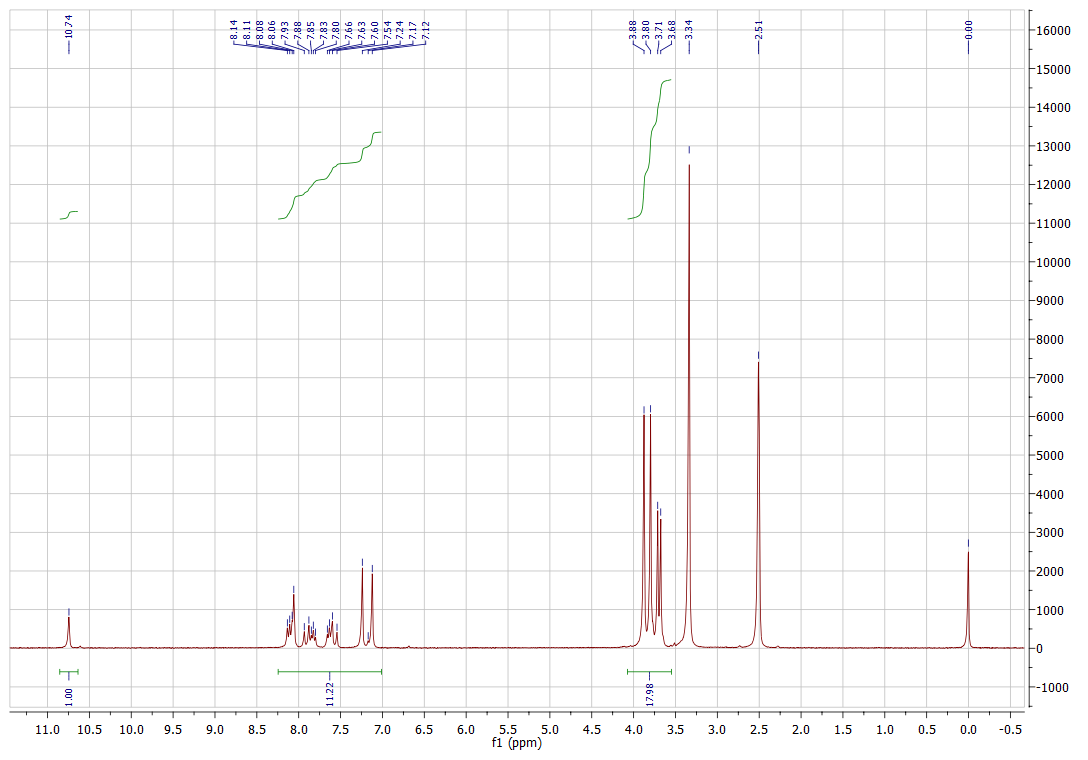


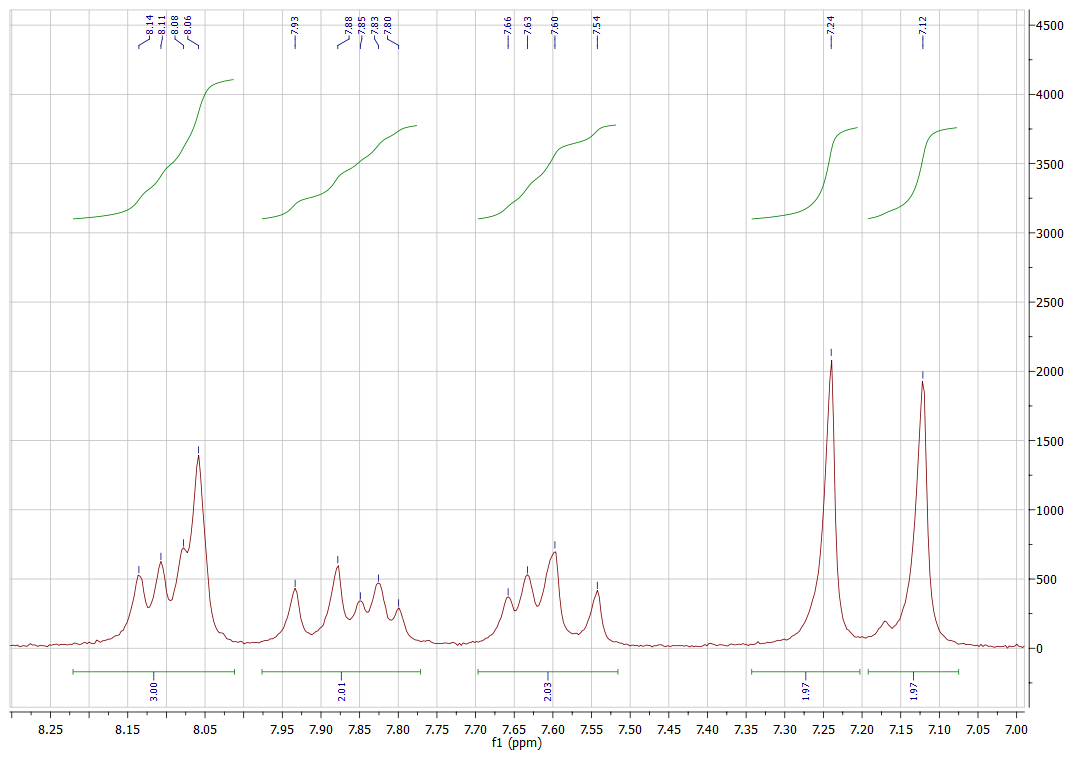


**Figure S66**. ^13^C APT NMR (DMSO-d_6_) of *(E)-N*-(3,4,5-trimethoxyphenyl)-2-(3,4,5-trimethoxystyryl)quinoline-4-carboxamide (**4o**)


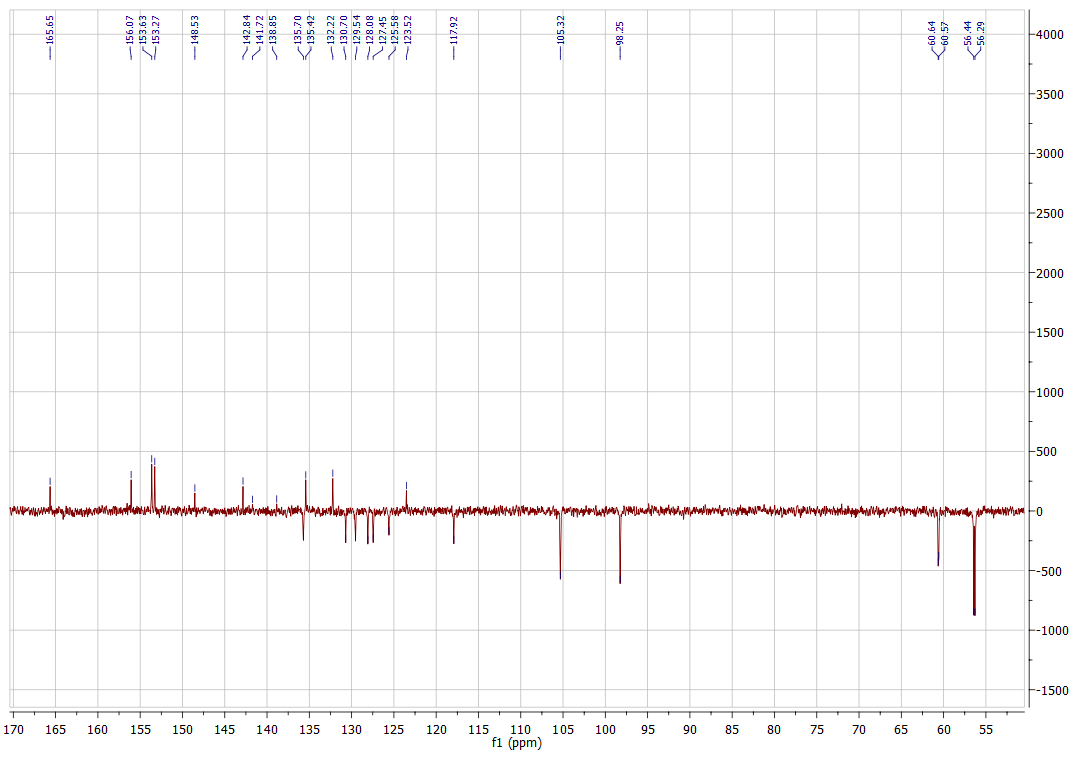


**Figure S67**. HPLC chromatogram and mass spectra of *(E)-N*-(3,4,5-trimethoxyphenyl)-2-(3,4,5-trimethoxystyryl)quinoline-4-carboxamide (**4o**)

| **Peak** | **RT** | **Area Sum %** | **Area** |
| --- | --- | --- | --- |
| 1 | 7.7714 | 98.18 | 996.04 |
| 2 | 7.9180 | 1.82 | 18.47 |

ESI-MS analysis for [C_30_H_30_N_2_O_7_+H^+^]: Calc.: 531.2126 *m*/*z*, exp.: 531.2126 *m*/*z*.

**Figure S68**. ^1^H NMR (DMSO-d_6_) of *(E)-N*-(3-chlorophenyl)-2-(3,4,5-trimethoxystyryl)quinoline-4-carboxamide (**4p**)


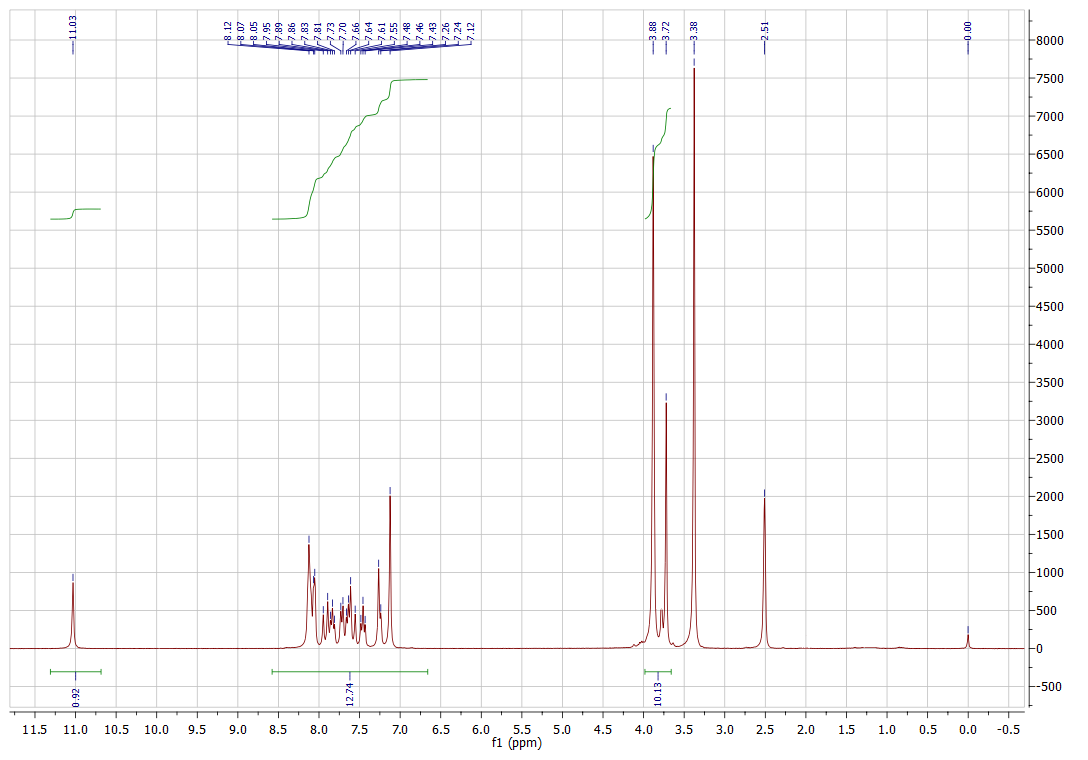


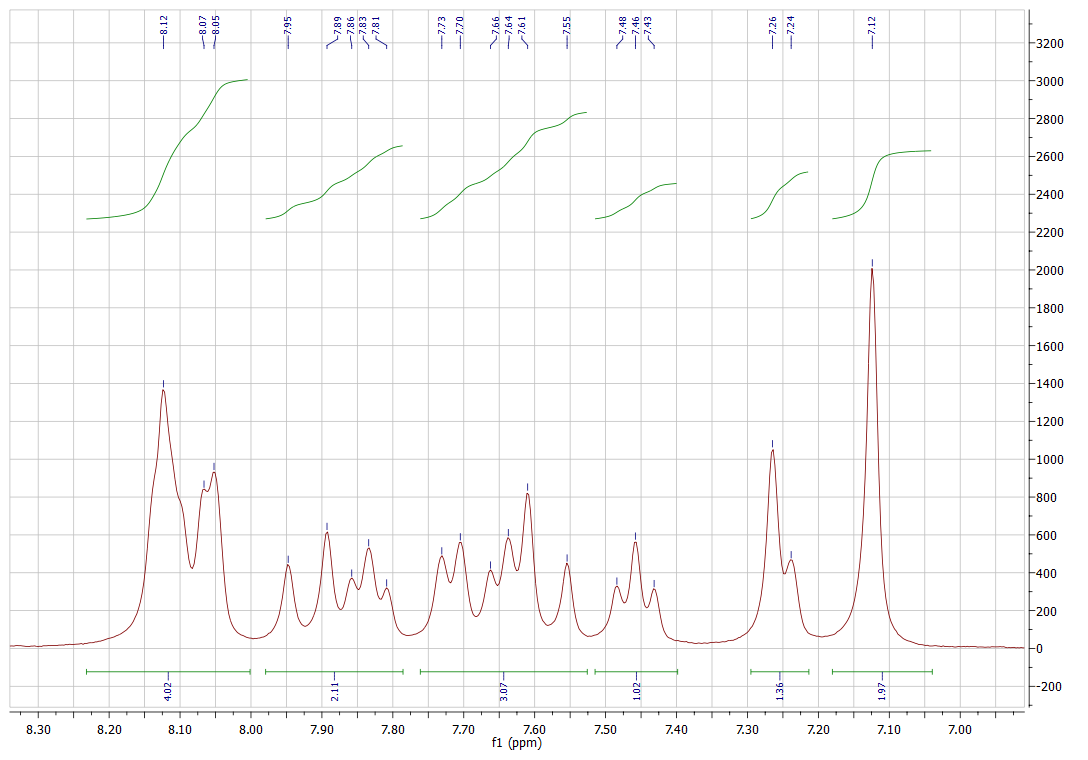


**Figure S69**. ^13^C APT NMR (DMSO-d_6_) of *(E)-N*-(3-chlorophenyl)-2-(3,4,5-trimethoxystyryl)quinoline-4-carboxamide (**4p**)

**
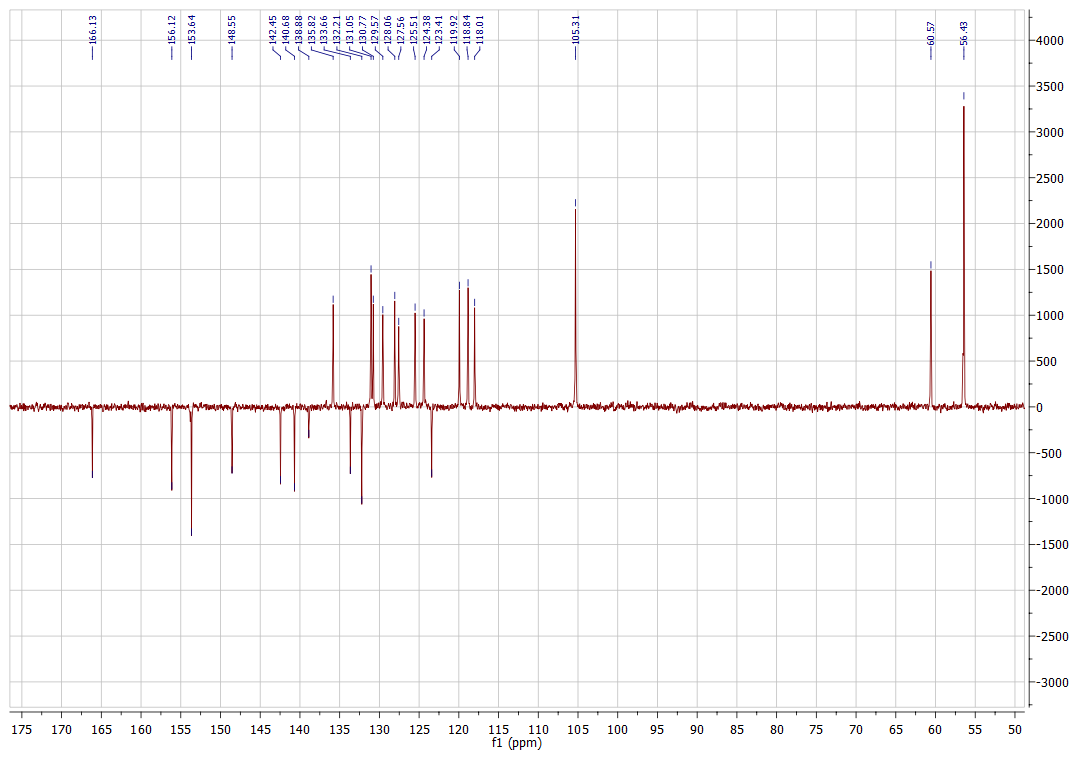
**


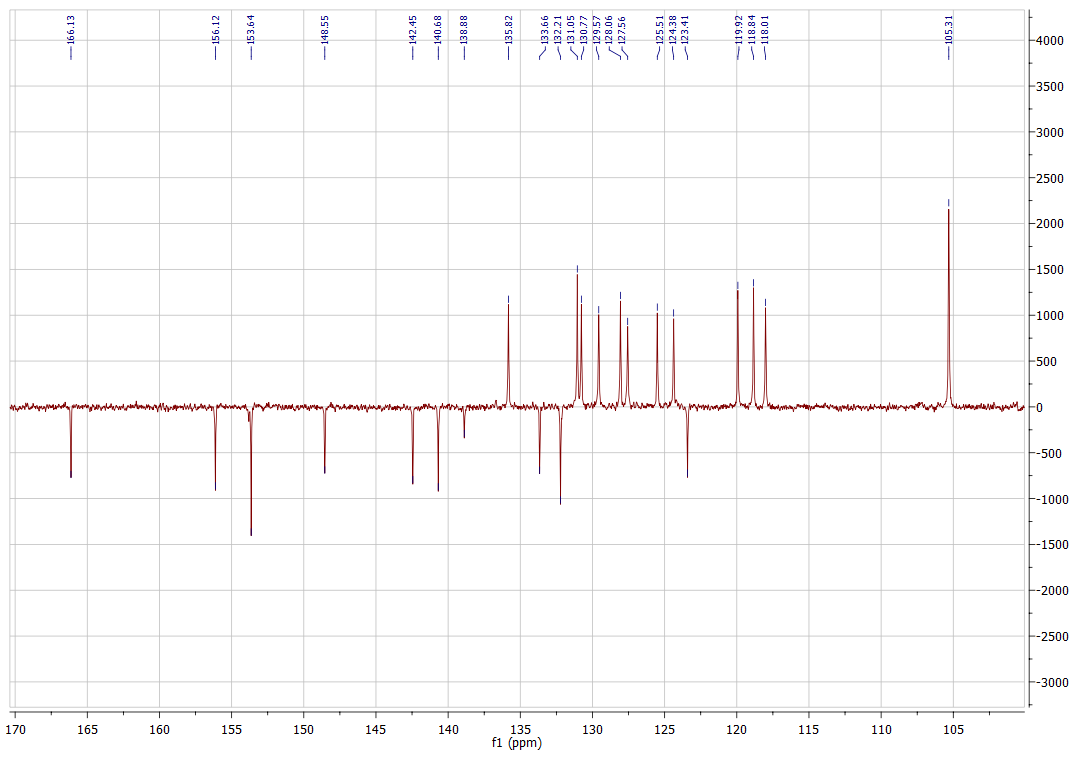


**Figure S70**. HPLC chromatogram and mass spectra of *(E)-N*-(3-chlorophenyl)-2-(3,4,5-trimethoxystyryl)quinoline-4-carboxamide (**4p**)

| **Peak** | **RT** | **Area Sum %** | **Area** |
| --- | --- | --- | --- |
| 1 | 8.47 | 0.37 | 28.16 |
| 2 | 8.92 | 99.25 | 7657.95 |
| 3 | 11.52 | 0.39 | 29.98 |

ESI-MS analysis for C_27_H_23_ClN_2_O_4_ [M+H^+^]: Calc.: 475.1419 *m*/*z*, exp.: 475.1419 *m*/*z*.

**Figure S71**. ^1^H NMR (DMSO-d_6_) of *(E)-N*-(4-chlorophenyl)-2-(3,4,5-trimethoxystyryl)quinoline-4-carboxamide (**4q**)

**Figure S72**. ^13^C APT NMR (DMSO-d_6_) of *(E)-N*-(4-chlorophenyl)-2-(3,4,5-trimethoxystyryl)quinoline-4-carboxamide (**4q**)

**Figure S73**. HPLC chromatogram and mass spectra of *(E)-N*-(4-chlorophenyl)-2-(3,4,5-trimethoxystyryl)quinoline-4-carboxamide (**4q**)

**<**

| **Peak** | **RT** | **Area Sum %** | **Area** |
| --- | --- | --- | --- |
| 1 | 7.2284 | 0.23 | 1.66 |
| 2 | 8.5151 | 0.24 | 1.73 |
| 3 | 8.9018 | 99.46 | 722.33 |
| 4 | 9.6484 | 0.07 | 0.56 |

ESI-MS analysis for C_27_H_23_ClN_2_O_4_ [2M+Na^+^]: Calc.: 971.2585 *m*/*z*, exp.: 971.2597 *m*/*z*.

**Figure S74**. ^1^H NMR (DMSO-d_6_) of *(E)-N*-(4-(trifluoromethyl)phenyl)-2-(3,4,5-trimethoxystyryl)quinoline-4-carboxamide (**4r**)

**Figure S75**. ^13^C APT NMR (DMSO-d_6_) of *(E)-N*-(4-(trifluoromethyl)phenyl)-2-(3,4,5-trimethoxystyryl)quinoline-4-carboxamide (**4r**)

**Figure S76**. HPLC chromatogram and mass spectra of *(E)-N*-(4-(trifluoromethyl)phenyl)-2-(3,4,5-trimethoxystyryl)quinoline-4-carboxamide (**4r**)

| **Peak** | **RT** | **Area Sum %** | **Area** |
| --- | --- | --- | --- |
| 1 | 7.2270 | 0.05 | 0.59 |
| 2 | 8.4903 | 0.23 | 2.96 |
| 3 | 9.2836 | 99.26 | 1282.13 |
| 4 | 10.2236 | 0.46 | 6.00 |

ESI-MS analysis for [C_28_H_23_F_3_N_2_O_4_+H^+^]: Calc.: 509.1683 *m*/*z*, exp.: 509.1684 *m*/*z*.

**Figure S77**. ^1^H NMR (DMSO-d_6_) of *(E)-N*-(4-nitrophenyl)-2-(3,4,5-trimethoxystyryl)quinoline-4-carboxamide (**4s**)

**Figure S78**. ^13^C APT NMR (DMSO-d_6_) of *(E)-N*-(4-nitrophenyl)-2-(3,4,5-trimethoxystyryl)quinoline-4-carboxamide (**4s**)

**Figure S79**. HPLC chromatogram and mass spectra of *(E)-N*-(4-nitrophenyl)-2-(3,4,5-trimethoxystyryl)quinoline-4-carboxamide (**4s**)

| **Peak** | **RT** | **Area Sum %** | **Area** |
| --- | --- | --- | --- |
| 1 | 8.383 | 98.48 | 67.77 |
| 2 | 8.953 | 1.52 | 1.05 |

ESI-MS analysis for [C_29_H_28_N_2_O_4_+H^+^]: Calc.: 469.2122 *m*/*z*, exp.: 469.2123 *m*/*z*.

**Figure S80**. ^1^H NMR (DMSO-d_6_) of *(E)-N*-(4-nitro-3-(trifluoromethyl)phenyl)-2-(3,4,5-trimethoxystyryl)quinoline-4-carboxamide (**4t**)

ù

**Figure S81**. ^13^C APT NMR (DMSO-d_6_) of *(E)-N*-(4-nitro-3-(trifluoromethyl)phenyl)-2-(3,4,5-trimethoxystyryl)quinoline-4-carboxamide (**4t**)

**Figure S82**. HPLC chromatogram and mass spectra of *(E)-N*-(4-nitro-3-(trifluoromethyl)phenyl)-2-(3,4,5-trimethoxystyryl)quinoline-4-carboxamide (**4t**)

| **Peak** | **RT** | **Area Sum %** | **Area** |
| --- | --- | --- | --- |
| 1 | 7.9720 | 0.59 | 13.09 |
| 2 | 8.9286 | 0.34 | 7.58 |
| 3 | 9.3820 | 98.55 | 2172.30 |
| 4 | 10.8153 | 0.51 | 11.20 |

ESI-MS analysis for [C_28_H_22_F_3_N_3_O_6_+H^+^]: Calc.: 554.1533 *m*/*z*, exp.: 554.1532 *m*/*z*.

**Figure S83**. ^1^H NMR (DMSO-d_6_) of *(E)-*2-(3-methylstyryl)-*N*-(4-nitro-3-(trifluoromethyl)phenyl)quinoline-4-carboxamide (**4u**)

**Figure S84**. ^13^C APT NMR (DMSO-d_6_) of *(E)-*2-(3-methylstyryl)-*N*-(4-nitro-3-(trifluoromethyl)phenyl)quinoline-4-carboxamide (**4u**)

**Figure S85**. HPLC chromatogram and mass spectra of *(E)-*2-(3-methylstyryl)-*N*-(4-nitro-3-(trifluoromethyl)phenyl)quinoline-4-carboxamide (**4u**)

| **Peak** | **RT** | **Area Sum %** | **Area** |
| --- | --- | --- | --- |
| 1 | 8.7772 | 0.15 | 3.48 |
| 2 | 9.7839 | 0.48 | 11.25 |
| 3 | 10.1039 | 99.37 | 2326.18 |

ESI-MS analysis for [C_26_H_18_F_3_N_3_O_3_+H^+^]: Calc.: 478.1373 *m*/*z*, exp.: 478.1373 *m*/*z*.

**Figure S86**. ^1^H NMR (DMSO-d_6_) of *(E)-*2-(4-methoxystyryl)-*N*-(4-nitro-3-(trifluoromethyl)phenyl)quinoline-4-carboxamide (**4v**)

**Figure S87**. ^13^C APT NMR (DMSO-d_6_) of *(E)-*2-(4-methoxystyryl)-*N*-(4-nitro-3-(trifluoromethyl)phenyl)quinoline-4-carboxamide (**4v**)

**Figure S88**. HPLC chromatogram and mass spectra of *(E)-*2-(4-methoxystyryl)-*N*-(4-nitro-3-(trifluoromethyl)phenyl)quinoline-4-carboxamide (**4v**)

| **Peak** | **RT** | **Area Sum %** | **Area** |
| --- | --- | --- | --- |
| 1 | 9.4407 | 98.35 | 339.81 |
| 2 | 10.4741 | 1.65 | 6.69 |

ESI-MS analysis for [C_26_H_18_F_3_N_3_O_4_+H^+^]: Calc.: 494.1322 *m*/*z*, exp.: 494.1329 *m*/*z*.

**Figure S89**. ^1^H NMR (DMSO-d_6_) of *(E)-*2-(4-chlorostyryl)-*N*-(4-nitro-3-(trifluoromethyl)phenyl)quinoline-4-carboxamide (**4w**)

**Figure S90**. ^13^C decoupling NMR (DMSO-d_6_) of *(E)-*2-(4-chlorostyryl)-*N*-(4-nitro-3-(trifluoromethyl)phenyl)quinoline-4-carboxamide (**4w**)

**Figure S91**. HPLC chromatogram and mass spectra of *(E)-*2-(4-chlorostyryl)-*N*-(4-nitro-3-(trifluoromethyl)phenyl)quinoline-4-carboxamide (**4w**)

| **Peak** | **RT** | **Area Sum %** | **Area** |
| --- | --- | --- | --- |
| 1 | 9.2509 | 0.81 | 1.38 |
| 2 | 9.6703 | 0.31 | 0.53 |
| 3 | 9.9043 | 0.86 | 1.48 |
| 4 | 10.2376 | 98.02 | 168.48 |

ESI-MS analysis for [C_25_H_15_ClF_3_N_3_O_3_+H^+^]: Calc.: 498.0827 *m*/*z*, exp.: 498.0829 *m*/*z*.

**Figure S92**. ^1^H NMR (DMSO-d_6_) of *(E)-*2-(3,4-dichlorostyryl)-*N*-(4-nitro-3-(trifluoromethyl)phenyl)quinoline-4-carboxamide (**4x**)

**Figure S93**. ^13^C APT NMR (DMSO-d_6_) of *(E)-*2-(3,4-dichlorostyryl)-*N*-(4-nitro-3-(trifluoromethyl)phenyl)quinoline-4-carboxamide (**4x**)

**Figure S94**. HPLC chromatogram and mass spectra of *(E)-*2-(3,4-dichlorostyryl)-*N*-(4-nitro-3-(trifluoromethyl)phenyl)quinoline-4-carboxamide (**4x**)

| **Peak** | **RT** | **Area Sum %** | **Area** |
| --- | --- | --- | --- |
| 1 | 9.7345 | 0.38 | 4.72 |
| 2 | 10.4078 | 0.77 | 9.64 |
| 3 | 10.7145 | 98.85 | 1235.01 |

ESI-MS analysis for [C_25_H_14_Cl_2_F_3_N_3_O_3_+H^+^]: Calc.: 532.0437 *m*/*z*, exp.: 532.0436 *m*/*z*.

**Figure S95**. ^1^H NMR (DMSO-d_6_) of *(E)-*2-(3-methylstyryl)-*N*-(4-(trifluoromethyl)phenyl)quinoline-4-carboxamide (**4y**)

**Figure S96**. ^13^C decoupling NMR (DMSO-d_6_) of *(E)-*2-(3-methylstyryl)-*N*-(4-(trifluoromethyl)phenyl)quinoline-4-carboxamide (**4y**)

**Figure S97**. HPLC chromatogram and mass spectra of *(E)-*2-(3-methylstyryl)-*N*-(4-(trifluoromethyl)phenyl)quinoline-4-carboxamide (**4y**)

| **Peak** | **RT** | **Area Sum %** | **Area** |
| --- | --- | --- | --- |
| 1 | 9.7745 | 0.58 | 1.54 |
| 2 | 10.0245 | 99.28 | 263.04 |
| 3 | 10.7445 | 0.14 | 0.36 |

ESI-MS analysis for [C_26_H_19_F_3_N_2_O+H^+^]: Calc.: 433.1522 *m*/*z*, exp.: 433.1522 *m*/*z*.

**Figure S98**. ^1^H NMR (DMSO-d_6_) of *(E)-*2-(4-chlorostyryl)-*N*-(4-(trifluoromethyl)phenyl)quinoline-4-carboxamide (**4z**)

**Figure S99**. ^13^C decoupling NMR (DMSO-d_6_) of *(E)-*2-(4-chlorostyryl)-*N*-(4-(trifluoromethyl)phenyl)quinoline-4-carboxamide (**4z**)

**Figure S100**. HPLC chromatogram and mass spectra of *(E)-*2-(4-chlorostyryl)-*N*-(4-(trifluoromethyl)phenyl)quinoline-4-carboxamide (**4z**)

| **Peak** | **RT** | **Area Sum %** | **Area** |
| --- | --- | --- | --- |
| 1 | 9.5797 | 0.41 | 5.64 |
| 2 | 9.9230 | 0.55 | 7.56 |
| 3 | 10.1997 | 98.78 | 1347.19 |
| 4 | 10.8930 | 0.26 | 3.49 |

ESI-MS analysis for [C_25_H_16_ClF_3_N_2_O +H^+^]: Calc.: 453.0976 *m*/*z*, exp.: 453.0975 *m*/*z*.

**Figure S101**. ^1^H NMR (DMSO-d_6_) of *(E)-*2-(4-bromostyryl)-*N*-(4-(trifluoromethyl)phenyl)quinoline-4-carboxamide (**4aa**)

**Figure S102**. ^13^C decoupling NMR (DMSO-d_6_) of *(E)-*2-(4-bromostyryl)-*N*-(4-(trifluoromethyl)phenyl)quinoline-4-carboxamide (**4aa**)

**Figure S103**. HPLC chromatogram and mass spectra of *(E)-*2-(4-bromostyryl)-*N*-(4-(trifluoromethyl)phenyl)quinoline-4-carboxamide (**4aa**)

| **Peak** | **RT** | **Area Sum %** | **Area** |
| --- | --- | --- | --- |
| 1 | 9.7301 | 0.43 | 4.84 |
| 2 | 10.0399 | 0.83 | 9.25 |
| 3 | 10.1901 | 0.29 | 3.3 |
| 4 | 10.3299 | 98.34 | 1102.32 |
| 5 | 10.9766 | 0.1 | 1.17 |

ESI-MS analysis for [C_25_H_16_BrF_3_N_2_O +H^+^]: Calc.: 499.0454 *m*/*z*, exp.: 499.0456 *m*/*z*.

**Figure S104**. ^1^H NMR (DMSO-d_6_) of *(E)-*2-(3,4-dichlorostyryl)-*N*-(4-(trifluoromethyl)phenyl)quinoline-4-carboxamide (**4ab**)

**Figure S105**. ^13^C decoupling NMR (DMSO-d_6_) of *(E)-*2-(3,4-dichlorostyryl)-*N*-(4-(trifluoromethyl)phenyl)quinoline-4-carboxamide (**4ab**)

**Figure S106**. HPLC chromatogram and mass spectra of *(E)-*2-(3,4-dichlorostyryl)-*N*-(4-(trifluoromethyl)phenyl)quinoline-4-carboxamide (**4ab**)

| **Peak** | **RT** | **Area Sum %** | **Area** |
| --- | --- | --- | --- |
| 1 | 10.2101 | 0.26 | 2.02 |
| 2 | 10.4501 | 0.25 | 1.94 |
| 3 | 10.7101 | 99.50 | 781.16 |

ESI-MS analysis for [C_25_H_15_Cl_2_F_3_N_2_O +H^+^]: Calc.: 487.0586 *m*/*z*, exp.: 487.0588 *m*/*z*.

**Figure S107**. ^1^H NMR (DMSO-d_6_) of *(E)-*2-(2-nitrostyryl)-*N*-(4-(trifluoromethyl)phenyl)quinoline-4-carboxamide (**4ac**)

**Figure S108**. ^13^C decoupling NMR (DMSO-d_6_) of *(E)-*2-(2-nitrostyryl)-*N*-(4-(trifluoromethyl)phenyl)quinoline-4-carboxamide (**4ac**)

**Figure S109**. HPLC chromatogram and mass spectra of *(E)-*2-(2-nitrostyryl)-*N*-(4-(trifluoromethyl)phenyl)quinoline-4-carboxamide (**4ac**)

| **Peak** | **RT** | **Area Sum %** | **Area** |
| --- | --- | --- | --- |
| 1 | 8.9686 | 0.65 | 4.99 |
| 2 | 9.3686 | 0.83 | 6.35 |
| 3 | 9.5886 | 98.29 | 751.55 |
| 4 | 10.3085 | 0.23 | 1.75 |

ESI-MS analysis for [C_25_H_16_F_3_N_3_O_3_+H^+^]: Calc.: 464.1217 *m*/*z*, exp.: 464.1217 *m*/*z*.

**Figure S110**. ^1^H NMR (DMSO-d_6_) of *(E)-*2-(3-nitrostyryl)-*N*-(4-(trifluoromethyl)phenyl)quinoline-4-carboxamide (**4ad**)

**Figure S111**. ^13^C decoupling NMR (DMSO-d_6_) of *(E)-*2-(3-nitrostyryl)-*N*-(4-(trifluoromethyl)phenyl)quinoline-4-carboxamide (**4ad**)

**Figure S112**. HPLC chromatogram and mass spectra of *(E)-*2-(3-nitrostyryl)-*N*-(4-(trifluoromethyl)phenyl)quinoline-4-carboxamide (**4ad**)

| **Peak** | **RT** | **Area Sum %** | **Area** |
| --- | --- | --- | --- |
| 1 | 9.0936 | 0.34 | 4.74 |
| 2 | 9.4836 | 0.57 | 8.09 |
| 3 | 9.7170 | 99.09 | 1401.07 |

ESI-MS analysis for [C_25_H_16_F_3_N_3_O_3_+H^+^]: Calc.: 464.1217 *m*/*z*, exp.: 464.1219 *m*/*z*.

**Figure S113**. ^1^H NMR (DMSO-d_6_) of *(E)-N*-(4-chlorophenyl)-2-(4-methoxystyryl)quinoline-4-carboxamide (**4ae**)

**Figure S114**. ^13^C decoupling NMR (DMSO-d_6_) of *(E)-N*-(4-chlorophenyl)-2-(4-methoxystyryl)quinoline-4-carboxamide (**4ae**)

**Figure S115**. HPLC chromatogram and mass spectra of *(E)-N*-(4-chlorophenyl)-2-(4-methoxystyryl)quinoline-4-carboxamide (**4ae**)

| **Peak** | **RT** | **Area Sum %** | **Area** |
| --- | --- | --- | --- |
| 1 | 6.2305 | 0.29 | 4.14 |
| 2 | 6.7539 | 0.17 | 2.48 |
| 3 | 7.2305 | 0.12 | 1.78 |
| 4 | 8.8405 | 99.32 | 1433.4 |
| 5 | 10.0872 | 0.1 | 1.39 |

ESI-MS analysis for [C_25_H_19_ClN_2_O_2_+Na^+^]: Calc.: 437.1027 *m*/*z*, exp.: 437.1026 *m*/*z*.

**Figure S116**. ^1^H NMR (DMSO-d_6_) of *(E)-N*-(4-chlorophenyl)-2-(4-chlorostyryl)quinoline-4-carboxamide (**4af**)

**Figure S117**. ^13^C decoupling NMR (DMSO-d_6_) of *(E)-N*-(4-chlorophenyl)-2-(4-chlorostyryl)quinoline-4-carboxamide (**4af**)

**Figure S118**. HPLC chromatogram and mass spectra of *(E)-N*-(4-chlorophenyl)-2-(4-chlorostyryl)quinoline-4-carboxamide (**4af**)

| **Peak** | **RT** | **Area Sum %** | **Area** |
| --- | --- | --- | --- |
| 1 | 8.8404 | 0.05 | 0.78 |
| 2 | 9.2257 | 0.12 | 1.93 |
| 3 | 9.9157 | 99.51 | 1540.54 |
| 4 | 10.5924 | 0.32 | 4.9 |

ESI-MS analysis for [C_24_H_16_Cl_2_N_2_O +H^+^]: Calc.: 419.0712 *m*/*z*, exp.: 419.0714 *m*/*z*.

**Figure S119**. ^1^H NMR (DMSO-d_6_) of *(E)-N*-(4-chlorophenyl)-2-(3,4-dichlorostyryl)quinoline-4-carboxamide (**4ag**)

**Figure S120**. ^13^C decoupling NMR (DMSO-d_6_) of *(E)-N*-(4-chlorophenyl)-2-(3,4-dichlorostyryl)quinoline-4-carboxamide (**4ag**)

**Figure S121**. HPLC chromatogram and mass spectra of *(E)-N*-(4-chlorophenyl)-2-(3,4-dichlorostyryl)quinoline-4-carboxamide (**4ag**)

| **Peak** | **RT** | **Area Sum %** | **Area** |
| --- | --- | --- | --- |
| 1 | 9.6001 | 0.06 | 0.54 |
| 2 | 9.9009 | 0.04 | 0.38 |
| 3 | 10.0309 | 0.03 | 0.29 |
| 4 | 10.4909 a | 99.86 | 847.33 |

ESI-MS analysis for [C_24_H_15_Cl_3_N_2_O +H^+^]: Calc.: 453.0323 *m*/*z*, exp.: 453.0324*m*/*z*.

**Figure S122**. ^1^H NMR (DMSO-d_6_) of *(E)-N*-(4-chlorophenyl)-2-(2-nitrostyryl)quinoline-4-carboxamide (**4ah**)

**Figure S123**. ^13^C decoupling NMR (DMSO-d_6_) of *(E)-N*-(4-chlorophenyl)-2-(2-nitrostyryl)quinoline-4-carboxamide (**4ah**)

**Figure S124**. HPLC chromatogram and mass spectra of *(E)-N*-(4-chlorophenyl)-2-(2-nitrostyryl)quinoline-4-carboxamide (**4ah**)

| **Peak** | **RT** | **Area Sum %** | **Area** |
| --- | --- | --- | --- |
| 1 | 8.845 | 1.65 | 4.17 |
| 2 | 9.082 | 98.35 | 248.1 |

ESI-MS analysis for [C_24_H_16_ClN_3_O_3_+H^+^]: Calc.: 430.0953 *m*/*z*, exp.: 430.0956 *m*/*z*.
