# Supplementary material for: Microwave‐Enhanced Synthesis of 2‐Styrylquinoline‐4‐Carboxamides With Promising Anti‐Lymphoma Activity
Source: Arch Pharm (Weinheim). 2025 Nov 24;358(11):e70148. doi: 10.1002/ardp.70148 (PMC12645083; doi:10.1002/ardp.70148)
Supplement: Supplementary file 2 — ArchPharm SupplMat InChI 2020 QN. [file ARDP-358-e70148-s002.doc]

**Supplemental Material: Novel Compounds and Biological Screening Results**

**Microwave-Enhanced Synthesis of 2-Styrylquinoline-4-carboxamides with Promising Anti-Lymphoma Activity**

Sardo, Ignazio^1,§^, Manfreda, Lorenzo^2,§^, Titone, Giulia Maria^1^, Barreca, Marilia^1^, Bivacqua, Roberta^1^, Spanò, Virginia^1^, Amata, Sara^1^, Zanolli, Arianna^2,3^, Bortolozzi, Roberta^3,4*^ Raimondi, Maria Valeria^1,*^, Viola, Giampietro^2,3^, Barraja, Paola^1^, Montalbano, Alessandra^1^

1 Department of Biological, Chemical and Pharmaceutical Sciences and Technologies (STEBICEF), University of Palermo, Palermo, Italy

2 Department of Woman’s and Child’s Health, University of Padova, Padova, Italy

3 Istituto di Ricerca Pediatrica IRP, Fondazione Città della Speranza, Padova, Italy

4 Department of Pharmaceutical and Pharmacological Sciences, University of Padova, Italy

* Correspondence:

Prof Maria Valeria Raimondi, Department of Biological, Chemical and Pharmaceutical Sciences and Technologies (STEBICEF), University of Palermo, Via Archirafi 32, 90123, Palermo, Italy

E-mail: [mariavaleria.raimondi@unipa.it](mailto:mariavaleria.raimondi@unipa.it)

Dr Roberta Bortolozzi, Department of Pharmaceutical and Pharmacological Sciences, University of Padova, Via Marzolo 5, 35131, Padova, Italy

E-mail: [roberta.bortolozzi@unipd.it](mailto:roberta.bortolozzi@unipd.it)

^§^ co-first authors.

**Table 1.** *In vitro* antiproliferative activity (IC₅₀, µM) of quinoline derivatives **4a–z** and **4aa–ah** against five human cancer cell lines.

|  |  | **Biological Activity (IC_50_. µM)^a^** | | | | |
| --- | --- | --- | --- | --- | --- | --- |
| **Cpd No.** | **InChl** | **HD-MB03** | **RPMI-8402** | **SU-DHL-8** | **A549** | **MDA-MB-231** |
| **4a** | InChI=1S/C24H18N2O/c27-24(26-19-11-5-2-6-12-19)22-17-20(16-15-18-9-3-1-4-10-18)25-23-14-8-7-13-21(22)23/h1-17H,(H,26,27)/b16-15+ | >10 | >10 | >10 | >10 | >10 |
| **4b** | InChI=1S/C25H20N2O/c1-18-8-7-11-20(16-18)27-25(28)23-17-21(15-14-19-9-3-2-4-10-19)26-24-13-6-5-12-22(23)24/h2-17H,1H3,(H,27,28)/b15-14+ | >10 | >10 | 5.05±0.58 | >10 | >10 |
| **4c** | InChI=1S/C25H20N2O/c1-18-11-14-20(15-12-18)27-25(28)23-17-21(16-13-19-7-3-2-4-8-19)26-24-10-6-5-9-22(23)24/h2-17H,1H3,(H,27,28)/b16-13+ | >10 | >10 | >10 | >10 | >10 |
| **4d** | InChI=1S/C26H22N2O/c1-18-9-8-10-19(2)25(18)28-26(29)23-17-21(16-15-20-11-4-3-5-12-20)27-24-14-7-6-13-22(23)24/h3-17H,1-2H3,(H,28,29)/b16-15+ | >10 | >10 | 6.53±1.49 | >10 | 5.60±0.79 |
| **4e** | InChI=1S/C27H24N2O4/c1-31-24-16-20(17-25(32-2)26(24)33-3)29-27(30)22-15-19(14-13-18-9-5-4-6-10-18)28-23-12-8-7-11-21(22)23/h4-17H,1-3H3,(H,29,30)/b14-13+ | 7.10±1.15 | >10 | 6.61±0.76 | >10 | >10 |
| **4f** | InChI=1S/C24H17ClN2O/c25-18-9-6-10-19(15-18)27-24(28)22-16-20(14-13-17-7-2-1-3-8-17)26-23-12-5-4-11-21(22)23/h1-16H,(H,27,28)/b14-13+ | >10 | >10 | 8.27±0.72 | >10 | >10 |
| **4g** | InChI=1S/C24H17ClN2O/c25-18-11-14-19(15-12-18)27-24(28)22-16-20(13-10-17-6-2-1-3-7-17)26-23-9-5-4-8-21(22)23/h1-16H,(H,27,28)/b13-10+ | >10 | 6.31±0.36 | 7.80±1.30 | >10 | >10 |
| **4h** | InChI=1S/C25H17F3N2O/c26-25(27,28)18-11-14-19(15-12-18)30-24(31)22-16-20(13-10-17-6-2-1-3-7-17)29-23-9-5-4-8-21(22)23/h1-16H,(H,30,31)/b13-10+ | >10 | 8.97±0.75 | 7.09±0.25 | >10 | >10 |
| **4i** | InChI=1S/C24H17N3O3/c28-24(26-18-12-14-20(15-13-18)27(29)30)22-16-19(11-10-17-6-2-1-3-7-17)25-23-9-5-4-8-21(22)23/h1-16H,(H,26,28)/b11-10+ | >10 | 1.14±0.12 | 1.64±0.25 | >10 | >10 |
| **4j** | InChI=1S/C25H16F3N3O3/c26-25(27,28)21-15-18(12-13-23(21)31(33)34)30-24(32)20-14-17(11-10-16-6-2-1-3-7-16)29-22-9-5-4-8-19(20)22/h1-15H,(H,30,32)/b11-10+ | 8.23±0.47 | >10 | 3.86±0.44 | >10 | >10 |
| **4k** | InChI=1S/C27H24N2O4/c1-31-24-15-18(16-25(32-2)26(24)33-3)13-14-20-17-22(21-11-7-8-12-23(21)28-20)27(30)29-19-9-5-4-6-10-19/h4-17H,1-3H3,(H,29,30)/b14-13+ | >10 | >10 | 5.33±0.52 | >10 | >10 |
| **4l** | InChI=1S/C28H26N2O4/c1-18-8-7-9-20(14-18)30-28(31)23-17-21(29-24-11-6-5-10-22(23)24)13-12-19-15-25(32-2)27(34-4)26(16-19)33-3/h5-17H,1-4H3,(H,30,31)/b13-12+ | >10 | 3.75±0.18 | 2.94±0.08 | 9.34±0.18 | 5.95±0.66 |
| **4m** | InChI=1S/C28H26N2O4/c1-18-9-12-20(13-10-18)30-28(31)23-17-21(29-24-8-6-5-7-22(23)24)14-11-19-15-25(32-2)27(34-4)26(16-19)33-3/h5-17H,1-4H3,(H,30,31)/b14-11+ | >10 | >10 | >10 | >10 | >10 |
| **4n** | InChI=1S/C28H26N2O4/c1-18-9-12-20(13-10-18)30-28(31)23-17-21(29-24-8-6-5-7-22(23)24)14-11-19-15-25(32-2)27(34-4)26(16-19)33-3/h5-17H,1-4H3,(H,30,31)/b14-11+ | >10 | 0.79±0.07 | 0.46±0.05 | >10 | >10 |
| **4o** | InChI=1S/C30H30N2O7/c1-34-24-13-18(14-25(35-2)28(24)38-5)11-12-19-15-22(21-9-7-8-10-23(21)31-19)30(33)32-20-16-26(36-3)29(39-6)27(17-20)37-4/h7-17H,1-6H3,(H,32,33)/b12-11+ | >10 | 4.55±0.42 | 2.09±0.14 | >10 | 5.95±0.89 |
| **4p** | InChI=1S/C27H23ClN2O4/c1-32-24-13-17(14-25(33-2)26(24)34-3)11-12-20-16-22(21-9-4-5-10-23(21)29-20)27(31)30-19-8-6-7-18(28)15-19/h4-16H,1-3H3,(H,30,31)/b12-11+ | 8.31±0.64 | 9.06±0.59 | 4.46±0.37 | >10 | 5.70±0.10 |
| **4q** | InChI=1S/C27H23ClN2O4/c1-32-24-14-17(15-25(33-2)26(24)34-3)8-11-20-16-22(21-6-4-5-7-23(21)29-20)27(31)30-19-12-9-18(28)10-13-19/h4-16H,1-3H3,(H,30,31)/b11-8+ | >10 | >10 | 4.61±0.84 | >10 | >10 |
| **4r** | InChI=1S/C28H23F3N2O4/c1-35-24-14-17(15-25(36-2)26(24)37-3)8-11-20-16-22(21-6-4-5-7-23(21)32-20)27(34)33-19-12-9-18(10-13-19)28(29,30)31/h4-16H,1-3H3,(H,33,34)/b11-8+ | >10 | >10 | 2.13±0.08 | >10 | >10 |
| **4s** | InChI=1S/C27H23N3O6/c1-34-24-14-17(15-25(35-2)26(24)36-3)8-9-19-16-22(21-6-4-5-7-23(21)28-19)27(31)29-18-10-12-20(13-11-18)30(32)33/h4-16H,1-3H3,(H,29,31)/b9-8+ | >10 | >10 | 1.37±0.14 | >10 | >10 |
| **4t** | InChI=1S/C28H22F3N3O6/c1-38-24-12-16(13-25(39-2)26(24)40-3)8-9-17-14-20(19-6-4-5-7-22(19)32-17)27(35)33-18-10-11-23(34(36)37)21(15-18)28(29,30)31/h4-15H,1-3H3,(H,33,35)/b9-8+ | >10 | >10 | >10 | >10 | >10 |
| **4u** | InChI=1S/C26H18F3N3O3/c1-16-5-4-6-17(13-16)9-10-18-14-21(20-7-2-3-8-23(20)30-18)25(33)31-19-11-12-24(32(34)35)22(15-19)26(27,28)29/h2-15H,1H3,(H,31,33)/b10-9+ | >10 | >10 | >10 | >10 | >10 |
| **4v** | InChI=1S/C26H18F3N3O4/c1-36-19-11-7-16(8-12-19)6-9-17-14-21(20-4-2-3-5-23(20)30-17)25(33)31-18-10-13-24(32(34)35)22(15-18)26(27,28)29/h2-15H,1H3,(H,31,33)/b9-6+ | >10 | >10 | >10 | >10 | >10 |
| **4w** | InChI=1S/C25H15ClF3N3O3/c26-16-8-5-15(6-9-16)7-10-17-13-20(19-3-1-2-4-22(19)30-17)24(33)31-18-11-12-23(32(34)35)21(14-18)25(27,28)29/h1-14H,(H,31,33)/b10-7+ | >10 | 3.56±0.44 | 8.25±0.67 | >10 | >10 |
| **4x** | InChI=1S/C25H14Cl2F3N3O3/c26-20-9-6-14(11-21(20)27)5-7-15-12-18(17-3-1-2-4-22(17)31-15)24(34)32-16-8-10-23(33(35)36)19(13-16)25(28,29)30/h1-13H,(H,32,34)/b7-5+ | >10 | >10 | >10 | >10 | >10 |
| **4y** | InChI=1S/C26H19F3N2O/c1-17-5-4-6-18(15-17)9-12-21-16-23(22-7-2-3-8-24(22)30-21)25(32)31-20-13-10-19(11-14-20)26(27,28)29/h2-16H,1H3,(H,31,32)/b12-9+ | 3.84±0.22 | 3.2±0.19 | 3.25±0.23 | 8.73±0.74 | 7.84±0.65 |
| **4z** | InChI=1S/C25H16ClF3N2O/c26-18-10-5-16(6-11-18)7-12-20-15-22(21-3-1-2-4-23(21)30-20)24(32)31-19-13-8-17(9-14-19)25(27,28)29/h1-15H,(H,31,32)/b12-7+ | 6.73±1.15 | >10 | 4.77±0.32 | >10 | >10 |
| **4aa** | InChI=1S/C25H16BrF3N2O/c26-18-10-5-16(6-11-18)7-12-20-15-22(21-3-1-2-4-23(21)30-20)24(32)31-19-13-8-17(9-14-19)25(27,28)29/h1-15H,(H,31,32)/b12-7+ | >10 | >10 | >10 | >10 | >10 |
| **4ab** | InChI=1S/C25H15Cl2F3N2O/c26-21-12-6-15(13-22(21)27)5-9-18-14-20(19-3-1-2-4-23(19)31-18)24(33)32-17-10-7-16(8-11-17)25(28,29)30/h1-14H,(H,32,33)/b9-5+ | 7.83±0.98 | >10 | 6.54±0.44 | >10 | >10 |
| **4ac** | InChI=1S/C25H16F3N3O3/c26-25(27,28)17-10-13-18(14-11-17)30-24(32)21-15-19(29-22-7-3-2-6-20(21)22)12-9-16-5-1-4-8-23(16)31(33)34/h1-15H,(H,30,32)/b12-9+ | >10 | >10 | >10 | >10 | >10 |
| **4ad** | InChI=1S/C25H16F3N3O3/c26-25(27,28)17-9-12-18(13-10-17)30-24(32)22-15-19(29-23-7-2-1-6-21(22)23)11-8-16-4-3-5-20(14-16)31(33)34/h1-15H,(H,30,32)/b11-8+ | >10 | >10 | >10 | >10 | >10 |
| **4ae** | InChI=1S/C25H19ClN2O2/c1-30-21-14-7-17(8-15-21)6-11-20-16-23(22-4-2-3-5-24(22)27-20)25(29)28-19-12-9-18(26)10-13-19/h2-16H,1H3,(H,28,29)/b11-6+ | >10 | 6.41±1.16 | 4.67±0.14 | >10 | >10 |
| **4af** | InChI=1S/C24H16Cl2N2O/c25-17-8-5-16(6-9-17)7-12-20-15-22(21-3-1-2-4-23(21)27-20)24(29)28-19-13-10-18(26)11-14-19/h1-15H,(H,28,29)/b12-7+ | >10 | 4.61±0.24 | 4.45±0.53 | >10 | >10 |
| **4ag** | InChI=1S/C24H15Cl3N2O/c25-16-7-10-17(11-8-16)29-24(30)20-14-18(28-23-4-2-1-3-19(20)23)9-5-15-6-12-21(26)22(27)13-15/h1-14H,(H,29,30)/b9-5+ | 6.93±0.06 | 6.42±0.43 | 3,49±0,11 | 7.71±0.56 | 5.52±0.22 |
| **4ah** | InChI=1S/C24H16ClN3O3/c25-17-10-13-18(14-11-17)27-24(29)21-15-19(26-22-7-3-2-6-20(21)22)12-9-16-5-1-4-8-23(16)28(30)31/h1-15H,(H,27,29)/b12-9+ | >10 | 1.33±0.02 | 2.15±0.45 | 6.92±0.68 | 4.99±0.35 |

Cell viability was evaluated after 72 h of treatment. Data are represented as mean +/- SEM of at least three independent experiments.

**Table 2.** IC₅₀ values (µM) of selected quinoline derivatives (**4i, 4n, 4s, 4ah**) against lymphoma-derived cell lines.

|  |  | **Biological Activity (IC_50_. µM)^a^** | | | | |
| --- | --- | --- | --- | --- | --- | --- |
| **Cpd No.** | **InChl** | **VL51** | **TOLEDO** | **SU-DHL-18** | **SU-DHL-1** | **KM-H2** |
| **4i** | InChI=1S/C24H17N3O3/c28-24(26-18-12-14-20(15-13-18)27(29)30)22-16-19(11-10-17-6-2-1-3-7-17)25-23-9-5-4-8-21(22)23/h1-16H,(H,26,28)/b11-10+ | 1.03±0.02 | 1.05±0.09 | 7.60±2.80 | 2.32±1.46 | 2.8±0.21 |
| **4n** | InChI=1S/C28H26N2O4/c1-18-9-12-20(13-10-18)30-28(31)23-17-21(29-24-8-6-5-7-22(23)24)14-11-19-15-25(32-2)27(34-4)26(16-19)33-3/h5-17H,1-4H3,(H,30,31)/b14-11+ | >10 | >10 | >10 | >10 | >10 |
| **4s** | InChI=1S/C27H23N3O6/c1-34-24-14-17(15-25(35-2)26(24)36-3)8-9-19-16-22(21-6-4-5-7-23(21)28-19)27(31)29-18-10-12-20(13-11-18)30(32)33/h4-16H,1-3H3,(H,29,31)/b9-8+ | >10 | >10 | >10 | >10 | >10 |
| **4ah** | InChI=1S/C24H16ClN3O3/c25-17-10-13-18(14-11-17)27-24(29)21-15-19(26-22-7-3-2-6-20(21)22)12-9-16-5-1-4-8-23(16)28(30)31/h1-15H,(H,27,29)/b12-9+ | >10 | 4**.**93±0**.**62 | 3**.**79±0**.**51 | 4**.**90±0**.**31 | 6**.**74±2**.**12 |

Cell viability was evaluated after 72 h of treatment. Data are represented as mean +/- SEM of at least three independent experiments.

^a^ Biological activity studies.

- 1. **Cell growth inhibitory effects**

*Cell Culture*

To test the newly synthesized compounds, various human cell lines were employed. HD-MB03 cells were purchased from DSMZ (Braunschweig, Germany), while A549, MDA-MB-231, RPMI-8420, SU-DHL-8, TOLEDO, VL51, SU-DHL-1, SU-DHL-18, and KM-H2 were obtained from ATCC (Manassas, VA, USA). Cells were cultured in DMEM (for MDA-MB-231 and A549) or RPMI-1640 (for RPMI-8420, SU-DHL-8, TOLEDO, VL51, SU-DHL-1, SU-DHL-18, KM-H2, and HD-MB03) media (Gibco, Milan, Italy). Both media were supplemented with 115 units/mL penicillin G (Gibco, Milan, Italy), 115 µg/mL streptomycin (Invitrogen, Milan, Italy), and 10% fetal bovine serum (Invitrogen, Milan, Italy). Peripheral blood mononuclear cells (PBMCs) were isolated from healthy donors as previously described. ^[1]^ For cytotoxicity assays, PBMCs were resuspended at 5 × 10⁵ cells/mL in complete RPMI medium with or without 2.5 µg/mL phytohemagglutinin (PHA) (Irvine Scientific) to stimulate T cell activation and proliferation. All cell lines were maintained at 37 °C in a humidified atmosphere containing 5% CO₂. All compounds were dissolved in DMSO at a 10 mM stock concentration. For dose–response assays, cells were seeded in 96-well plates at densities optimized for each cell line: HD-MB03 at 10,000 cells/well, A549 at 4,000 cells/well, and MDA-MB-231 at 7,000 cells/well. All other cell lines were seeded at 20,000 cells/well in a final volume of 100 µL per well. After 24 hours, cells were treated with a 6-point serial dilution of compounds starting from 10 µM, in triplicate for statistical analysis. Seventy-two hours post-treatment, 10 µL of resazurin solution (100 µg/mL) was added to each well, and cells were incubated for an additional 3–6 hours. Fluorescence was measured using a Spark 10M microplate reader (Tecan Group Ltd., Männedorf, Switzerland) with excitation at 535 nm and emission at 600 nm. The IC₅₀ was defined as the concentration of compound required to inhibit cell proliferation by 50% compared to cells treated with the highest DMSO concentration.

**1.2 Statistical analysis**

All statistical analyses were conducted using GraphPad Prism 10 software (GraphPad, La Jolla, California). The data shown in bar graphs are expressed as the mean ± SEM. Asterisks above the bars indicate statistical significance relative to control cells or specific groups (specified in brackets, if applicable). The significance thresholds were defined as follows: * p < 0.05, ** p < 0.01, *** p < 0.001, **** p < 0.0001.

Reference

[1] Romagnoli, R. et al. (2017). Synthesis and Biological Evaluation of 2-Methyl-4,5-Disubstituted Oxazoles as a Novel Class of Highly Potent Antitubulin Agents. Scientific Reports. https://doi.org/10.1038/srep46356.
